# Tumor collagens predict genetic features and patient outcomes

**DOI:** 10.1038/s41525-023-00358-9

**Published:** 2023-07-06

**Authors:** Kevin S. Guo, Alexander S. Brodsky

**Affiliations:** Department of Pathology and Laboratory Medicine, Rhode Island Hospital, Warren Alpert Medical School, Brown University, Providence, RI USA

**Keywords:** Cancer microenvironment, Biomarkers, Data mining, Tumour biomarkers

## Abstract

The extracellular matrix (ECM) is a critical determinant of tumor fate that reflects the output from myriad cell types in the tumor. Collagens constitute the principal components of the tumor ECM. The changing collagen composition in tumors along with their impact on patient outcomes and possible biomarkers remains largely unknown. The RNA expression of the 43 collagen genes from solid tumors in The Cancer Genome Atlas (TCGA) was clustered to classify tumors. PanCancer analysis revealed how collagens by themselves can identify the tissue of origin. Clustering by collagens in each cancer type demonstrated strong associations with survival, specific immunoenvironments, somatic gene mutations, copy number variations, and aneuploidy. We developed a machine learning classifier that predicts aneuploidy, and chromosome arm copy number alteration (CNA) status based on collagen expression alone with high accuracy in many cancer types with somatic mutations, suggesting a strong relationship between the collagen ECM context and specific molecular alterations. These findings have broad implications in defining the relationship between cancer-related genetic defects and the tumor microenvironment to improve prognosis and therapeutic targeting for patient care, opening new avenues of investigation to define tumor ecosystems.

## Introduction

Successful personalized medicine requires tumor classification that predicts patient responses with high accuracy^1^. Molecular targeting has not typically considered the tumor extracellular matrix (ECM) when considering therapy options. The ECM is a collection of structural proteins and enzymes that provides a cohesive scaffold for cells and tissues. Identifying ECM composition and the functional roles of constituent proteins in healthy and diseased states is at a relatively early stage of characterization. ECM environments in malignant neoplasias can influence tumor growth, metastasis, and overall disease outcomes, in part through regulation of known cancer hallmarks^2^. The tumor microenvironment is increasingly being shown to impact cell states, responses to therapy, patient outcomes, and potential for novel biomarkers^3^.

Collagens constitute up to 30% of the total protein in the body and are the major components of the ECM. High expression of collagens in tumors has long been associated with poor outcomes as part of stromal expression signatures in many, but not all, malignancies^4,5^. These stroma, or mesenchymal, groups are enriched for collagens, yet the expression of collagens in tumors of various cancer types has not been evaluated.

Other studies have evaluated aspects of the matrisome in TCGA suggesting that an organized transcription factor network specifies the ECM^6^. Proteomics is revealing the complexity of the matrisome originating from multiple cell types^7,8^. Individual collagens such as collagen types IV^9^, collagen type X^10^, and XI^11^ have been proposed as biomarkers. Together, these findings underscore the importance of the matrisome and collagens in forming the tumor ecosystem. Because collagens and the matrisome proteins are secreted from multiple cell types, the composition of a site-specific ECM reflects the output of myriad cell types and pathways acting in concert to influence disease progression.

Collagens are a large complex family of proteins with a wide range of structures and tissue-specific expression (Supplemental Table 1). Minor collagens are informally defined as any collagen at lower expression levels compared to the major structural collagens, Types I, II, and III, found in high abundance in many tissues. Fibrillar collagens constitute a subgroup of collagens and include type I and many of the collagens that interact with type I including types V, XI, XII, XIV, and XVII (Supplemental Table 1)^12,13^. Dysregulation leads to expression of many tissue-specific collagens in a range of cancer types.

Cellular pathways and molecular alterations impart context-dependent impacts, complicating therapeutic decision-making and incurring variable treatment responses. We hypothesized that tumors can be classified by ECM composition, revealing connections among functional pathways and the microenvironment. We find that classifying tumors solely based on expression of the 43 human collagen genes captures discriminating features between cancer types, compared to results from hundreds of genes representing the matrisome, and simplifies analysis to demonstrate specificity. Collagen-defined classification in multiple cancer types identified strong associations with overall survival, pathways, molecular alterations, histology, and tissue of origin. Collagen clustering classified tumors with high aneuploidy into distinct groups associated with survival in multiple cancer types. We developed a machine-learning model to predict aneuploidy and CNAs from collagen expression alone.

Enrichment of specific somatic mutations in tumor groups classified by collagen expression implies that the combination of genetics and constituent collagens present in the tumor microenvironment may be exploited to improve therapeutic targeting. Due to the pan-cancer nature of this study, the following sections will report patterns across tumor types and highlight specific findings made possible by classifying samples by collagen expression. Many detailed results are summarized in the supplemental data and in the supplementary information describing the findings for each cancer type. Together, these observations highlight the importance of tumor ECM composition in mediating the impact of molecular alterations and the immunoenvironment to guide cancer therapies.

## Results

### Collagen clustering identifies tissue of origin

**Fig. 1:**
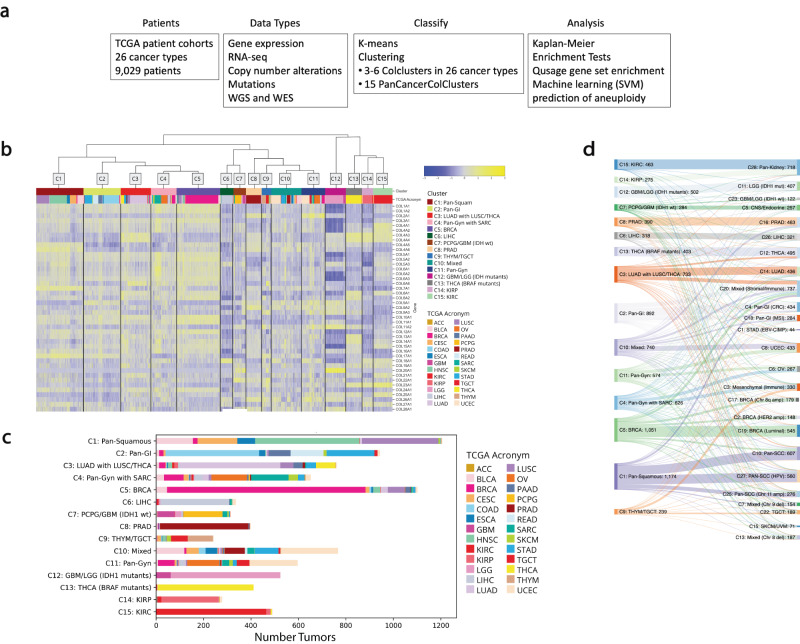
PanCan clustering of 9029 TCGA solid tumors by collagen mRNA expression. **a** Flow chart showing the analysis workflow and data used in this study. **b** Heat map of collagen expression across all TCGA cancer types clustered into 15 groups via k-means with Pearson correlation distance defines PanColClusters. **c** The homogeneity and heterogeneity of the PanColClusters. The number of tumors from each cancer type in each PanColCluster is plotted. **d** Sankey diagram showing correspondence between PanColClusters (left) and Hoadley et. al. PanCan iClusters^19^ (right).

Individual collagens have been utilized as biomarkers for specific cell types and cell states including COL17A1 marking skin stem cells^14,15^, COL10A1 as a hypertrophic chondrocyte differentiation marker^16^, and COL22A1 as a chondrocyte differentiation marker^17^. These findings suggested that collagens could distinguish cancer types by their tissues of origin. We hypothesized that considering all collagens together would define specific cancer types and associations with molecular features. Figure 1a summarizes the analysis approach to test collagen defining tumor groups including the key feature: evaluation of the relationship between collagen-defined tumor groups with cell state and cell genetics. To test this idea, we started by evaluating collagen clusters across the TCGA RNAseq data from 9029 solid tumors all together (Fig. 1b). The available data used from TCGA for each cancer type is summarized in Supplemental Table 2. The abbreviations for each cancer type from TCGA are listed in Supplemental Table 2. 15 PanCancer collagen-defined k-means clusters, named PanColClusters, was optimal using gap statistics^18^. 7 PanColClusters were homogeneous while the other 8 were relatively heterogeneous (Fig. 1c). The PanColClusters were highly concordant with the 28 iClusters defined by multi-omics by Hoadley et al. (Fig. 1d)^19^. The cluster for each tumor ID is recorded in Supplemental Table 3. These observations suggest that collagen expression classified cancer types by their tissues of origin resulting in the same seminal observations as other approaches. These findings suggest that the collagen centered ECM characteristics of tumors maintains the features of the tissue of the origin.

### Collagen expression classifies tumors

**Fig. 2:**
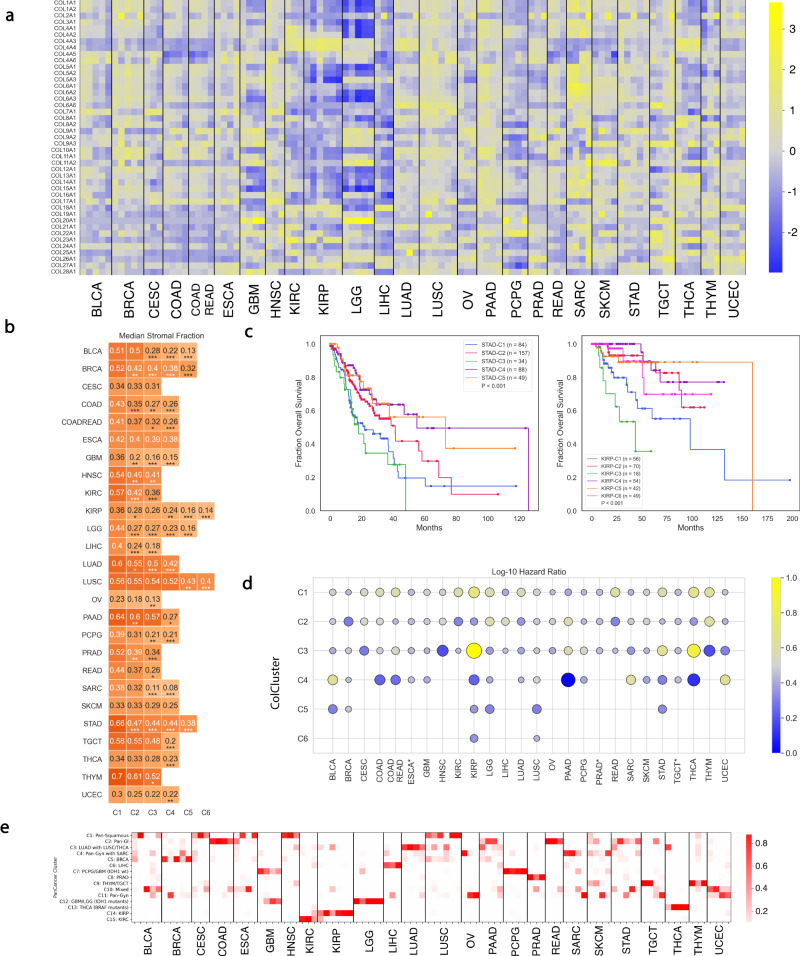
Clustering of each tumor in each cancer type in TCGA by collagen mRNA expression creating the ColClusters. **a** Heat map of median collagen expression in each ColCluster. Z-scored in each tumor type. **b** Heat map showing the median stromal fraction for each ColCluster. *’s indicate *p* value by Kolmogorov-Smirnov (KS) test relative to ColCluster-1 for each cancer type. **c** Representative Kaplan–Meier curves for stomach adenocarcinoma (STAD) and renal papillary cell carcinoma (KIRP) with accompanying log-rank *p* values and relative Hazard Ratio (HR) in each ColCluster. Kaplan–Meir curves for each cancer type are in Supplemental Fig. 4. **d** Bubble plot of the negative log−10 Hazard Ratios (HRs) for each ColCluster. *’s indicate poor model fit. Numbers for each cancer type from the univariate and multivariate Cox proportional hazards model are listed in Tables S4 and S5. **e** Heat map highlighted proportion of tumors in each ColCluster represented in each PanColCluster.

We used k-means clustering to classify each of the 25 TCGA solid tumor cancer types, plus the combination of COADREAD, with ≥100 cases independently (Fig. 2a). Silhouette and gap statistic analysis identified the optimal number of clusters for each tumor type (Supplemental Fig. 1). Between 3–6 well-defined clusters were identified for each cancer type. We named these k-means defined clusters, collagen clusters (ColClusters). The ColCluster for each tumor ID is recorded in Supplemental Table 3. The stroma fraction estimates the non-tumor cellular component from calculations done by Thorsson et al.^20^.

Because of the relationship with stroma fraction for COL1A1 and fibrillar collagens, ColClusters were ordered by stroma fraction with ColCluster 1 having the highest median stroma fraction in each tumor type (Fig. 2b). The difference in stroma fraction across ColClusters was not significant between ColClusters 1 and 2 in 14/26 cancer types examined (Fig. 2b), with 8/26 cancer types having a similar stroma fraction in ColCluster-2 compared to ColCluster 1, and only 3/26 ColCluster 3’s had similar stroma fraction compared to their respective ColCluster 1’s. ColClusters with similar levels of high fibrillar collagen expression were defined by distinct and strong differences in specific collagens, often minor collagens (Fig. 2a, Supplemental Fig. 3). ColCluster 1’s with high stroma fraction were not always the cluster with the highest expression of fibrillar collagens (Fig. 2a). For example, the Esophageal Carcinoma (ESCA), ColCluster-C4 (ESCA-C4) had highly expressed fibrillar collagens and yet had similar stroma fraction compared to the other ColClusters.

Collagen mRNA expression in bulk tumor samples is a result of a complicated contribution from multiple cell types including fibroblasts, macrophages, and tumor cells^7,21^. We evaluated the relationship between the stroma fraction, the ColClusters, and collagen expression to test if collagen composition was correlated with stroma fraction. The relationship between collagens and stroma fraction varies in each tumor setting. As collagen type I is the dominant collagen, often highly secreted by fibroblasts and stroma cells, COL1A1 is positively correlated with the stroma fraction in all but 3 of the cancer types (Supplemental Fig. 2). Stroma and collagen expression were also strongly positively correlated for many of the other fibrillar collagens including types III, V, XI, and XIV, regulators of collagen type I fiber width and structure (Supplemental Table 1)^12^. However, it is notable that even in ColClusters with similar stroma fraction (Fig. 2b), significant collagen expression differences were observed suggesting that the total collagen composition and stroma fraction are distinct characteristics. Moreover, many of the non-fibrillar collagens including collagen types VII, VIII, IX, COL4A5, COL4A6, and others, were only modestly correlated with stroma fraction (Supplemental Fig. 2). This observation, along with the other findings in this study, highlights the distinct features of collagen composition differing from stroma fraction in characterizing tumors.

Many collagens have ≥10 fold dynamic range across the ColClusters and cancer types, suggesting clear definition of the ColClusters (Supplemental Fig. 3). In particular, minor collagens such as COL7A1, COL10A1, COL17A1, and collagen type IX have large dynamic ranges. These collagens have very specific expression in normal tissue, and yet exhibited dysregulated expression in many cancer types, though often in only a fraction of tumors in each cancer type (Fig. 2a) and (Supplemental Fig. 3). Notably, COL25A1 is dysregulated and expressed in KIRC, LUAD, SARC, THCA, and UCEC cancer types (Supplemental Fig. 3). Other high dynamic range collagens including collagen type IX and COL4A5/6 marked many specific ColClusters (Fig. 2a). Some brain specific collagens help define ColClusters. The brain specific collagen, COL20A1, was only significantly expressed in neuronal lineage tumors (GBM, LGG, PCPG, and TGCT) (Supplemental Fig. 3). COL25A1 is a transmembrane collagen normally expressed in brain tissue and developing myoblasts^22^.

6 genes express collagen type IV which is the major component of the basement membrane^23^. Each pair of collagen type IV’s (COL4A1/A2, COL4A3/A4, and COL4A5/A6) are co-regulated from shared divergent promoters^24,25^. Collagen type IV shows a large dynamic range of expression both across and within cancer types (Supplemental Fig. 3), defining both PanColClustersand in the ColClusters. 26 of the 104 ColClusters were defined by high expression of one of the COL4 pairs, including all cancer types except Prostate Adenocarcinoma (PRAD). Mutations in COL4A1/A2 and COL4A3/A4 generate distinct mice phenotypes^26^ and these observations suggest differential functions in these tumors. The roles and relationships among these COL4 genes in tumors remains poorly defined. These observations suggest a complex relationship among the dysregulated expressed COL4 genes.

### Overall survival

Individual collagens and the ECM have been linked with overall survival in many cancers^10^. Survival associations of the groups defined by ColClusters were evaluated, and many distinct patterns were identified. In 13/26 of the cancer types, ColClusters were significantly associated with overall survival with p values ≤0.05 by Kaplan–Meier analysis. Kaplan–Meier curves for all the cancer types are shown in Supplemental Fig. 4. Univariate Cox Proportional Hazards derived hazard ratios in each cluster are summarized in (Fig. 2c) (Supplemental Table 4). Representative Kaplan–Meier curves are shown for KIRP and STAD highlighting the significant separation of high and low risk patients (Fig. 2d). ColClusters with relatively high stroma fractions were often biased to lower overall survival in the Kaplan–Meier Analysis (Supplemental Fig. 4). Of the 13 cancer types with significant ColCluster separation, C1 was the highest, or among the highest, risk in 10 cancer types. Notably, COL1A1 was expressed the most highly in C1 of the ColClusters in 19/26 cancer types (Supplemental Fig. 3 and Supplemental Fig. 2). Multivariate cox proportional hazards analysis showed that ColClusters were independent of stroma fraction and staging in many cancer types (Supplemental Table 5). All together, these observations suggest that the specific composition of collagen-defined tumor ECM was associated with overall survival in multiple cancer types, independent from the total stroma fraction and staging.

### PanCancer mapping to ColClusters

Combining PanColClusters and ColClusters helps define and identify the tumors’ unique collagen features. For the tumor types in a range of heterogeneous PanColClusters, specific ColClusters were commonly associated with different tissue origins highlighting a range of ECM, collagen and phenotypes (Fig. 2e).

Squamous is a feature of many BLCA, ESCA, and LUSC tumors. PanCan-C1 is the pan-squamous group (Fig. 1c). This group was distinguished by expression of minor collagens including COL4A5/COL4A6, COL7A1 and COL17A1. COL17A1 has been reported to be a squamous marker^27^. Both COL17A1 and COL7A1 are involved in Epidermolysis Bullosa. Although most LUSC tumors were in PanCan-C1:Squamous, LUSC-C4 is a group of LUSC tumors, characterized by high expression of COL4A3/COL4A4, that resembles LUAD and mapped to the PanCan-C3:LUAD group. Bladder Adenocarcinoma (BLCA) was distributed into both the C1:Pan-squamous and the C10:mixed cluster. All tumors in the BLCA-2 ColCluster were in PanCan-C1 while C10:mixed mapped to BLCA-C3, C4, and C5. Thus, collagen expression distinguished histology features in BLCA. C). These same tumors mapped to different iClusters in Hoadley et al.^19^ (Supplemental Table 3).

Ovarian tumors (OV) were split into 2 PanColClusters, PanCan-C4 and PanCan-C11. Although OV ColClusters were not associated with overall survival, these findings suggest that the high collagen type I, fibrillar collagen, and high stroma OV-C1 group is similar to many Sarcoma (SARC) tumors that have relatively longer overall survival,while OV-C2 and OV-C3, clustered with SARC-C4, the SARC group with shorter overall survival, defined by minor collagens COL2A2 and COL4A5/A6. Because the sarcomas in TCGA are a diverse collection of tumors, we found that collagen clustering identified the tissue of origin and histologies of the range of TCGA sarcomas (Supplemental Fig. 16). Together, these findings highlight how collagen expression identified tumors with similar environments, even with different tissue origins. PanColCluster-C4 is also marked by relatively high expression of other fibrillar collagens, similar to PanColCluster-C5. C4 and C5 are distinguished by differences in a few minor collagens with C4 having lower expression of COL4A5/COL4A6 and COL12A1, with higher expression of COL18A1.

### Collagen expression classified tumors similarly to the whole matrisome gene set

Collagens are the most abundant component of the matrisome. A number of groups have investigated classifications defined by large sets of matrisome genes^6,28^. We compared how collagen only clustering corresponded to classifications using 890 matrisome genes (Supplemental Fig. 5). These observations suggest that collagen expression alone captured the seminal features of classifying tumors based on ECM features. Characterizing tumors with smaller gene sets, such as the 43 collagens compared to all the matrisome genes, improves the likelihood of biomarker utility with patients.

### Mutation Rate and MSI Status

**Fig. 3:**
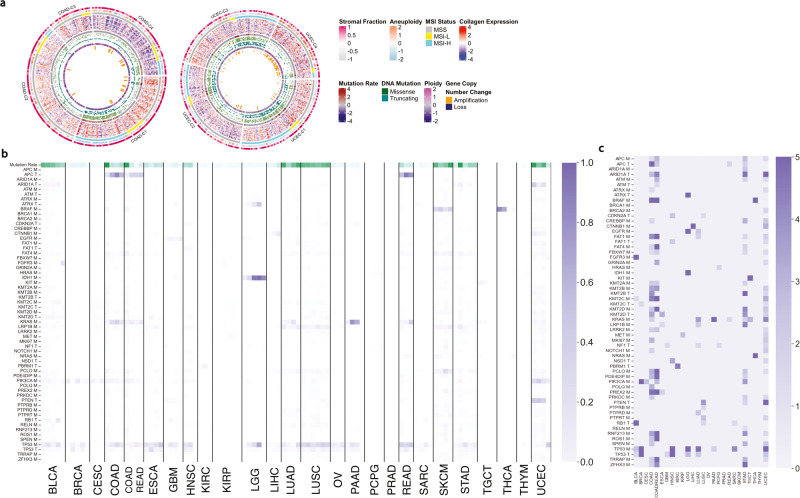
Mutation rate and somatic mutations enriched in specific ColClusters. **a** Circular diagrams showing the distribution of molecular alterations, mutation rate, and MSI status in colon adenocarcinoma (COAD) and endometrial carcinoma (UCEC) tumors. The molecular alterations and ECM are distinguished in these tumors. The circular diagrams for the other cancer types are shown in Supplemental Fig. 17. **b** Fraction of tumors in each ColCluster with the mutated gene with relative non-silent mutation rate as first row. **c** Heat map shows the log *p* values to highlight the somatic mutations with significantly biased distribution in the ColClusters for each cancer type as determined by chi-squared.

We evaluated the relationship between overall mutation rates and microsatellite instability (MSIH) with the ColClusters in stomach adenocarcinoma (STAD), colon adenocarcinoma (COAD), and uterine corpus endometrial carcinoma (UCEC). MSIH tumors were localized to the high stroma fraction and fibrillar collagen clusters, COAD-C1, STAD-C2, and UCEC-C1 (Supplemental Fig. 7). Notably, a subset of STAD MSS tumors were placed in STAD-C2, with MSIH tumors, because they had similar collagen composition, despite vastly different mutation signatures (Fig. 3a), suggesting convergence on ECM phenotypes originating from distinct genotypes.

A group of COAD MSS tumors was identified with similar collagen composition to MSIH COAD tumors in COAD-C1 and COADREAD-C1. The MSS and MSIH tumors in COAD-C1 and COADREAD-C1 had similar phenotypic characteristics and yet very different genotypes (Supplemental Fig. 14). Some MSIH tumors were grouped into other COAD and COADREAD ColClusters with MSS tumors based on their collagen composition.

### Somatic Mutations

Targeting tumors based on molecular alterations is subject to variable responses with often unclear reasons from patient to patient^1^. We hypothesized that collagens could indicate contextual differences of the impact of molecular alterations on the tumor. To test these ideas, we evaluated if ColClusters were enriched for the top 50 most frequently mutated genes, as listed in cBioPortal for the 26 cancer types in this study^29^. We also included variants in ABL1, AKT1, AKT2, ALK1, BRCA1, EGFR, ERBB2, FGFR1, FGFR3, FLT3, HRAS, JAK2, KIT, MET, NRAS, PDGFRA, and RET, known critical drivers in some contexts. Figure 3b, c shows many mutated genes significantly biased in ColClusters.

There were two general types of patterns observed with gene variants: 1) One ColCluster with strong positive or negative enrichment for a specific molecular alteration relative to the other ColClusters, suggesting a link between a specific ECM and a specific molecular alteration. 2) Multiple ColClusters had similar genetic profiles of candidate drivers or suppressors suggesting that the genotypes were associated with a diverse collagen composition in these settings.

We describe enrichment in specific ColClusters for a few representative examples. TP53 is the most frequently mutated gene and has been linked to remodeling the ECM^30^. TP53 showed distinct and significantly biased patterns across the ColClusters in BLCA, BRCA, GBM, HNSC, LGG, LUAD, SARC, and UCEC (Fig. 3b). These observations highlight how collagen composition can vary greatly with similar or distinct molecular alterations. The specific molecular alteration is distinguishable relative to collagen expression patterns.

We highlight examples of pattern 1, where specific molecular alterations were localized to one or two ColClusters, except UCEC-C4, which was enriched for P53 missense variants (Fig. 3 and Supplemental Fig. 14). These patterns highlight the connections between genetic features with specific collagen compositions.

PTEN truncations were enriched in all of the UCEC ColClusters (Fig. 3), except UCEC-C4, which was enriched for P53 missense variants (Fig. 3 and Supplemental Fig. 14). Wnt signaling in liver tumors is often activated by CNNTB1 mutations^31^. Tumors with CTNNB1 mutations were significantly less frequent in LIHC-C1 compared to LIHC-C2 and LIHC-C3 (p<0.001), even with similar overall mutation rates. LIHC-C1 is marked by higher fibrillar collagen expression compared to LIHC-C2 and LIHC-C3.

The 7 IDH1 mutation tumors were in GBM-C3. LGG-C1 and LGG-C2 were enriched for IDH1 wild-type tumors and associated with shorter overall survival. These findings highlight connections between the collagen environment and IDH1/2 mutation status in brain tumors.

One of the striking differences between LGG and GBM is the variation in collagen type IV composition, which is associated with vessel formation in the brain environment^32^. LGG tumors had lower COL4A1/2 expression compared to GBM. LGG tumors with relatively higher COL4A1/2 expression compared to other LGG tumors, and also enriched for mutant IDH1/2, may have a distinct vasculature compared to wild-type IDH tumors with lower levels of COL4A1/2 expression^33,34^. These findings link vasculature diversity with collagen composition diversity.

Collagen clustering identified a set of tumors with FGFR3 mutations (Fig. 3). Collagen clustering in BLCA tumors exemplify pattern 1 for FGFR3 mutations. Mutations in FGFR3 have been associated with less aggressive bladder tumors^35^ and were localized to BLCA-C5, marked by high expression of COL4A5/COL4A6 and COL10A1, with relatively low expression of fibrillar collagens, and the lowest HR among the 5 BLCA ColClusters (Supplemental Fig. 4).

The distribution of variants in the BRCA ColClusters exemplifies both patterns (Supplemental Figs. 14, 17). Collagen clustering separated tumors into PIK3CA (BRCA-C1 and BRCA-C3), and TP53 mutation groups (BRCA-C2 and BRCA-C4). BRCA-C1, C3 and C5 were enriched for hormone positive tumors, while BRCA-C2 and C4 were enriched for Triple Negative Breast Cancers (TNBC). BRCA-C2 AND C4 have similar collagen type IV levels, but differential expression of collagen type IX and COL2A1. This is an example of Pattern 2, where similar molecular alterations have distinct tumor ECM composition. Also noteworthy is that many TNBC tumors were classified with hormone positive BRCA tumors because of their common collagen environments (Supplemental Fig. 14 and Supplemental Fig. 17).

Genes mutated at a high rate in specific cancer types were distributed in distinct patterns across ColClusters exemplifying pattern 2. ARID1A in UCEC, KRAS in COAD, and TP53 were localized to multiple ColClusters (Fig. 3b). These ColClusters with similar putative drivers have distinct collagen environments, and different relationships with long and short overall survival (Fig. 2d).

Variants in tumor suppressors also showed significant bias. Tumors with RB1 truncations were the majority of tumors or biased towards BLCA-4, LUSC-C2/C3, and SARC-C3. RB1 loss in these tumors was linked to distinctive collagen environments relative to the other tumors in each cancer type. RB1 is reported to mediatethe cell cycle, adhesion and the tumor microenvironment^36^.

PAAD-C1 had a lower mutation rate, including lower fraction of tumors with mutated KRAS, but this is likely because of the high stroma fraction and lower overall tumor cell percentage in these cases. Re-evaluation of the rate of KRAS mutation in TCGA showed the expected high rate of KRAS mutations that were missed in TCGA sequencing analysis^37^. It is of note that PAAD-C1, defined by high fibrillar collagen expression, had only a modest difference in stroma fraction compared to the other ColClusters (Fig. 2b and Supplemental Fig. 6).

### Gene copy number aberrations

**Fig. 4:**
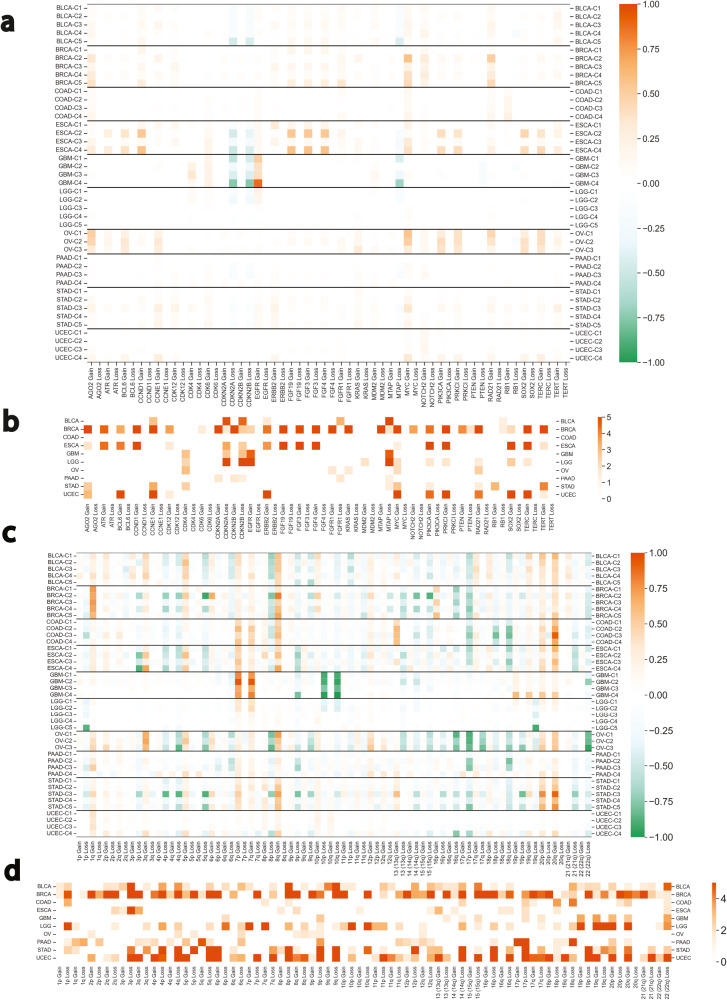
Copy number alterations (CNAs) at the gene and chromosome arm level were enriched in specific ColClusters. Some cancer types are shown here and all others are in (Supplemental Fig. 8). **a** Fraction of tumors in each ColCluster with gene copy number amplifications (orange) or deletions (green). **b** Gene copy number changes that were significantly biased across the ColClusters as determined by Chi-squared tests. Heat map indicates negative log p values. **c** Fraction of tumors in each ColCluster with chromosome arm amplification (orange) or deletion (green). All chromosome arm CNAs and cancer types tested are shown in (Supplemental Fig. 9). **d** Chromosome arm copy number changes that were significantly biased across the ColClusters as determined by Chi-squared tests. Heat map indicates negative log *p* values.

We evaluated the most common gene Copy Number Aberrations (CNAs) observed in the 26 cancer types for bias across the ColClusters using the copy number calls provided by TCGA. We chose the top 50 genes with the most CNAs according to cBioPortal^29^. Gene level CNAs showed distinct distributions among the ColClusters in all cancer types except COAD (Supplemental Fig. 8). Figure 4 shows the gene CNAs for some cancer types enriched in ColClusters.

Some examples are highlighted. Amplifications of Myc showed a biased distribution in 10 cancer types. Notably, Myc amplifications were not enriched in most ColCluster-1’s, except for LIHC and OV. In BRCA-C2 and BRCA-C4, amplification of MYC and RAD21 were enriched. 86% of TGCT tumors showed copy gains for KRAS, and yet, KRAS copy gain was negatively enriched in TGCT-C1. EGFR copy gains were significantly biased in 9 cancer types including GBM. OV tumors have high levels of CNAs with relatively low mutation rates^38^. Notably, even though the three OV ColClusters have similar overall aneuploidy (Supplemental Fig. 10). However, collagen classification of OV tumors identified specific tumor groups linking CNAs with ECM context. Specific CNAs were distinct in OV-C1 and OV-C2 compared to OV-C3. OV-C3 was enriched for SOX2 copy gains, while OV-C1 was enriched for AGO2, MYC and RAD21 copy gains. OV-C1 and OV-C2 were significantly enriched for gains in MYC, while OV-C3 was enriched for CDK4 and KRAS. Distinct CNAs in OV and other cancer types highlight relationships between the local tumor microenvironment and molecular genetics.

Tumor suppressors such as the cell cycle regulators, CDNK2A and MTAP, showed copy number losses in specific ColClusters including GBM-C1 and C4, ESCA-C2 and C4, and BLCA-C5. SARC-C1 was enriched for MDM2, CCNE1, and CDK4 gains. These findings reveal connections between molecular alterations controlling the cell cycle and the collagen environment.

### Chromosome arm copy number aberrations

We evaluated chromosome arm CNAs with at least 10 CNAs in the cancer type. The distribution of many chromosome arm CNAs was significantly biased across ColClusters in many tumor settings (Supplemental Fig. 9). Selection of cancer types are shown in (Fig. 4c). ColClusters enriched for ≥3 copy number changes across multiple chromosomes were observed in BRCA, ESCA, HNSC, KIRC, KIRP, STAD, THYM, and UCEC (Fig. 4). Some ColClusters high levels of both gains and losses including: COAD-C3, LIHC-C2, LUAD-C3, STAD-C3, STAD-C5, THYM-C3, and UCEC-C4. Others were biased towards gains or losses including BRCA-C2 and C4, KIRP-C3, and PAAD-C4.

Chromosome arm level CNAs were localized to a specific ColCluster in many cancer types including CESC (1q gain), COAD (1p loss), GBM (9p loss), HNSC (11q loss), LGG (1q gain, 19q loss), PAAD (17p, 18q gains), PCPG (3p loss) and SARC (10q loss). Some chromosome arm-level CNAs were strongly biased across the ColClusters 3p loss in multiple cancer types including BRCA, BLCA, ESCA, HNSC, LUSC, and STAD. 90% of KIRC tumors have 3p loss, but those that do not are almost all in KIRC-C3 (Fig. 4). ESCA-C2 was enriched for 8p gains, while ESCA-C1 and ESCA-C3 were enriched for 18q losses.). 10p loss was enriched in LGG-C1 and LGG-C2 while 19q loss was enriched only in LGG-C5. These findings suggest the existence of specific relationships between collagen expression and chromosome arm CNAs linking the cancer genome with the tumor ECM.

### Collagen expression predicts CNAs

**Fig. 5:**
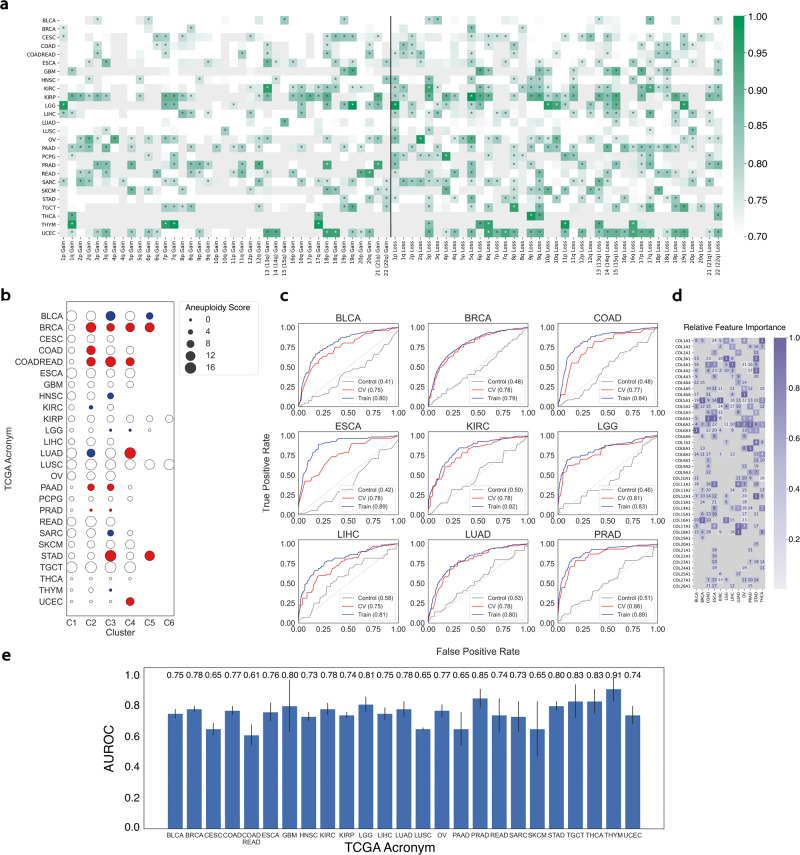
Aneuploidy is predicted by collagen mRNA expression by support vector machine (SVM) models. **a** Heat map of AUROC scores from SVM prediction of chromosome arm copy number from collagen expression. All predictions with mean AUC ≥0.75 are highlighted. White indicates AUC ≤0.5, gray indicates not computed due to insufficient number of cases. **b** Bubble plot of median aneuploidy scores in each collagen cluster normalized relative to ColCluster-1 (C1)for each cancer type. Colored bubbles indicate statistical significance at the <0.01 level compared to C1 by a Kolmogorov-Smirnov test. Red indicates higher median aneuploidy score compared to the C1 for each cancer type, and blue indicates lower median aneuploidy score compared to C1. **c** Representative ROC plots for tumor types with mean cross-validation AUC ≥0.80. **d** Relative importance of each collagen gene in the models with mean AUC ≥0.75 with ranking annotated for each weight. **e** Summary of mean AUC scores from SVM model cross-validation for each tumor type. Error bars represent the standard deviation of the cross-validation folds.

To test for specific relationships between chromosome arm copy number aberrations and collagen expression, we implemented a Support-Vector Machine (SVM) model to predict chromosome arm CNA status based solely on collagen mRNA expression. Inclusion of the stroma fraction in the model had only a modest improvement in the model predictions. We tested the quality of the model by 5-fold cross-validation in each cancer type with ≥10 cases with the chromosome arm CNA. We used the area under the curve (AUC) of the receiver operating characteristic (ROC) to evaluate the model performance in each tumor setting (Fig. 5a). As an example, the SVM model predicted 3p loss in 59% of the cancer types with at least 10 cases with 3p loss (AUC>0.75). This suggests that collagen composition is strongly linked to 3p loss in multiple cancer settings. 5q and 9q losses were predicted very well in multiple cancer types as well. These connections suggest potential genetic adaptations required to thrive in specific collagen-defined ECM environments.

### Collagen clusters associated with aneuploidy

These observations of the CNAs suggest there may also be associations between ploidy, genome doublings, and aneuploidy in the ColClusters. Aneuploidy has been associated with a range of treatment responses and patient survival risk depending on contexts^39,40^. We evaluated the relationship between aneuploidy and the collagen-defined clusters. 12 cancer types showed significantly altered distributions across the ColClusters as assessed by a Kolmogorov-Smirnov test (Fig. 5b). Some cancer types including BLCA, COAD, LUAD, STAD, and UCEC showed very strong biases with the majority of high or low aneuploid tumors grouped into 1 or 2 ColClusters. Notably, many of these cancer types had high aneuploid tumors in the ColCluster with relatively lower expression of fibrillar collagens. To emphasize this finding, ColCluster aneuploidy levels were normalized to ColCluster 1 (Fig. 5b).

Specific ColCluster aneuploidy distribution patterns are quite striking in some cancer types. In STAD, two ColClusters, STAD-C3 and STAD-C5, with relatively high aneuploidy were identified, but with strikingly different overall survival and collagen expression patterns (Fig. 2e). The median overall survival for the high aneuploidy tumors in STAD-C3 is 14.4 months compared to 37.5 months for the high aneuploidy tumors in STAD-C5. Similarly, UCEC-C4, with the shortest overall survival (Supplemental Fig. 4), is enriched for high aneuploidy tumors, yet many other high aneuploidy tumors were distributed across the other 3 UCEC ColClusters. These observations suggest that the high aneuploidy tumors in UCEC-C4 are a distinct set of aggressive high aneuploidy tumors with different collagen composition (Fig. 2d). These observations suggest that the combination of aneuploid and collagen composition may explain some of the confounding observations where aneuploidy is not always associated with worse outcomes^41^.

### Collagen expression patterns predict aneuploidy levels

To explore the relationship between collagen expression and aneuploidy further, we used a SVM model to test if collagen expression can predict aneuploidy levels in tumors. We modeled the aneuploidy scores with Gaussians to partition the scores into high and low categories (Supplemental Table 3). The SVM predicted the aneuploidy status of 9 of the cancer types with area under the curves (AUC) ≥0.8 by Receive Operator Characteristic (ROC) analysis (Fig. 5c). Many AUCs for other cancer types were very close (Fig. 5e) and Supplemental Fig. 11). Evaluation of the weights for each collagen reveal that each cancer type has specific collagen expression patterns (Fig. 5d).

We compared the SVM predictions of aneuploid levels from collagen expression to the ColCluster-aneuploidy enrichments. Some cancer types, including ESCA, LIHC, and OV, did not show biased distribution of aneuploidy scores in the ColClusters, and yet, the SVM accurately predicted aneuploidy levels (Fig. 5e), suggesting a relationship between collagen expression and aneuploidy. Other cancer types such as SARC and UCEC showed ColCluster enrichments with reasonable SVM predictions with AUCs of 0.73 and 0.74, respectively (Fig. 5e and Supplemental Fig. 11). Similar performance of SVM models were observed for related metrics, genome doublings and ploidy (Supplemental Fig. 12).

Together, these observations strongly support a relationship between the cancer genome and collagen expression. They further imply that not all aneuploid tumors have similar features. It is the combination of aneuploidy and the ECM that should be considered to understand tumor progression and therapeutic options.

### Immune cell infiltration varies with collagen environment

The tumor ECM is a critical regulator of immune cell infiltration through myriad mechanisms including mechanical blockage^42^, angiogenesis by basement membrane collagens^43^, or stimulation of specific signaling pathways^42^. Enrichment of immune cell expression signatures derived from Tamborero et al.^44^ were determined by QuSAGE to identify the ColClusters enriched for each cell type compared to the other ColClusters (Supplemental Fig. 13)^45^. Regulatory T cells and macrophages were enriched in many of the high stroma ColCluster 1’s. 9/26 ColCluster 1’s were highest for T-regs compared to the other ColClusters, suggesting connections between these immunosuppressive cells and tumors with high expression of fibrillar collagens. These observations identify the tumor collagen composition to be a critical feature linking immune cell infiltration with tumors and overall survival.

BLCA and STAD highlight the relationship between collagen expression and immune cell infiltration. BLCA-C1 and BLCA-C2 have similar levels of stroma fraction, as well as expression of many of the fibrillar collagens, and yet showed distinct immune cell infiltration patterns. BLCA-C1 was enriched for activated CD8 T cells, B cells and regulatory T cells while BLCA-C2 was enriched for aDC cells. These observations connect specific collagen-defined tumor classes with immune cell infiltration patterns. STAD-C1 and C2 have similar stroma fractions (Fig. 2b), but significantly different immunoenvironments. STAD-C1 may be more immunosuppressive with higher T-reg infiltration, while STAD-C2 may be more immune activated with enrichment for activated dendritic cells (aDCs) and higher expression of inflammatory gene signatures (Supplemental Fig. 13), consistent with STAD-C2 associated with longer overall survival (Fig. 2c).

**Fig. 6:**
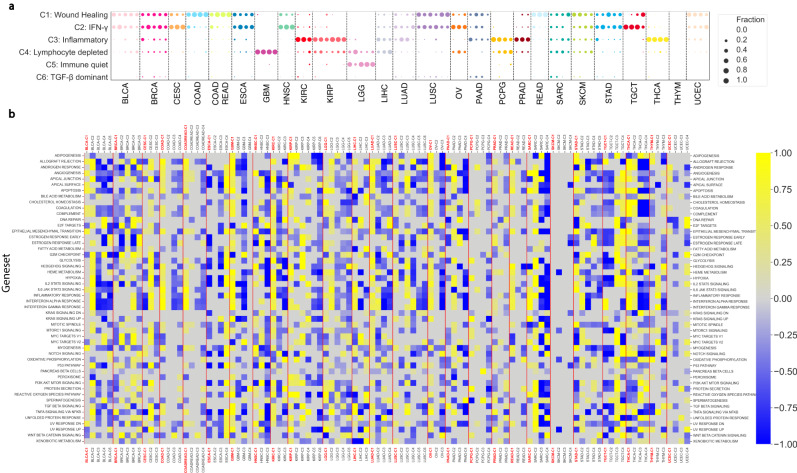
Immune environments and hallmarks characterizing the ColClusters. **a** Bubble plot showing bias in immune sub-types, as defined by Thorsson et al.^20^, across ColClusters. The bubble color indicates the cancer type and the bubble size indicates the fraction of the ColCluster with the designated immune subtype. **b** Heat map indicating relative enrichment of the 50 MsigDB hallmark gene sets between ColClusters as computed via QuSAGE. Each cancer type was evaluated separately.

To assess the global immunoenvironment in each ColCluster, we identified significant biased distributions for the 6 immunotypes defined by Thorsson et al. in all but 2 cancer settings (Fig. 6 and Supplemental Table 6) BRCA-C2 and C4 were enriched for the “IGFN-*γ*" immune group, similar to all 3 OV ColClusters, and UCEC-C4. These groups have high levels of structural variations with high aneuploidy levels (Fig. 4). LGG-C2 had a more GBM-like immunoenvironment as it is enriched for “C4-lymphocyte depleted" compared to the large majority of tumors in the other 4 LGG ColClusters in immunotype-C5, “immunologically quiet". LUAD-C3 and C4 were enriched for immunotype-C3, “Inflammatory", while the other LUAD ColClusters were enriched for immunotypes C1 and C2. LUSC-C4 was biased to immunotype C2, while the others were divided between immunotypes C1 and C2. UCEC showed a distinct pattern with immunotype C2, “IFN-g dominant", strongly enriched in the high aneuploidy UCEC ColCluster-4, while the other 3 UCEC ColClusters were biased towards immunotype-C1, “Wound Healing". ColClusters for LIHC and SKCM had a distinct difference in immunotypes. In some cancer types, the same immunotype was observed in multiple ColClusters including COAD, COADREAD, GBM, LGG, PRAD, and THCA. In other cancer types, including BLCA and BRCA, the distribution of multiple immunotypes was similar across all the ColClusters with only subtle biases observed. The high aneuploidy ColClusters, including STAD-C3 and UCEC-C4 were enriched for distinct immunotypes relative to the other STAD and UCEC ColClusters. These observations suggest that collagen composition was associated with specific immunoenvironments.

### Collagen clusters associated with cancer hallmarks

To assess the biological features enriched in each ColCluster, the 50 Molecular Signature Database (MSigDB) cancer hallmark gene sets were evaluated using Qusage (Fig. 6b)^46^. Qusage identified the ColClusters where each gene set is most enriched relative to the other ColClusters. Of the ColClusters with high expression of fibrillar collagens, 19 were enriched for the highest number of hallmark gene sets in their respective cancer type.

TGF*β* and EMT, in particular, have been associated with expression of fibrillar collagens and high stroma ColClusters. in myriad models^47^. We examined the relationship between the high stroma fraction ColCluster-1’s and these hallmarks. 13/26 ColCluster-1’s were highest in TGF*β* signaling. EMT was highest in ColCluster-1’s including BLCA, CESC, COAD, COADREAD, GBM, HNSC, KIRC, KIRP, LIHC, LUAD, LUSC, OV, READ, STAD, THCA, and UCEC. ESCA-C4, LGG-C2, PAAD-C2, PCPG-C2, SARC-C2, TCGT-C4, THYM-C3, were relatively high in EMT and fibrillar collagen gene expression in these cancer types (Fig. 6b). The angiogenesis hallmark gene set was associated with the high collagen type I and fibrillar collagen expression ColClusters in 19 cancer types.

Not all hallmark gene sets were associated with high fibrillar collagen expression as many showed specific patterns across the ColClusters and were enriched in other ColClusters. Bile acids may decrease adhesion to collagens^48^. Bile acid metabolism with the highest QuSAGE values in most cancer types was enriched in ColClusters other than the high fibrillar collagen ColClusters, except for BRCA-C3, KIRP-C1, and TGCT-C4. Another example is Myc regulated expression. ColClusters including BRCA-C2, BRCA-C4, STAD-C3 had relatively high expression of the Myc target gene set, consistent with Myc amplification in these clusters. These observations connect distinct pathways with lower fibrillar collagen environment ColClusters.

### Aneuploidy tumors in context

**Fig. 7:**
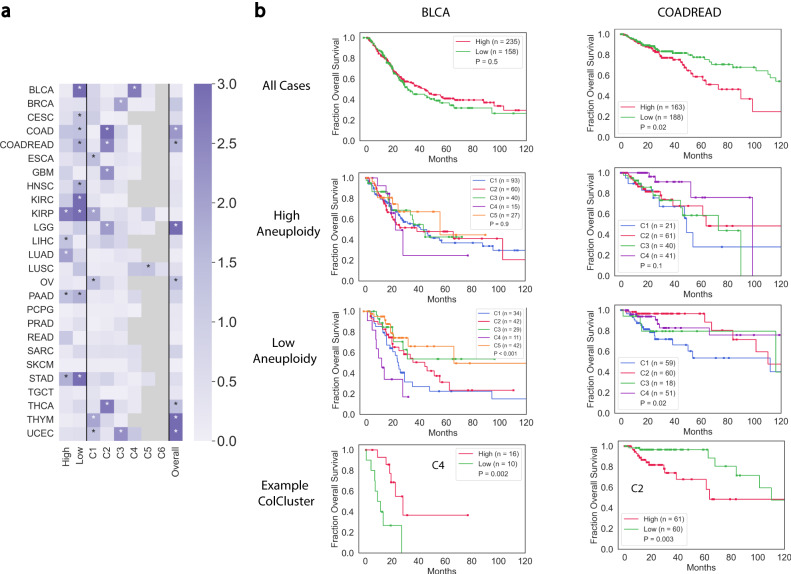
ColClusters combined with aneuploidy identify tumors associated with overall survival in collagen-defined environments. **a** Heat map of negative log p values from Kaplan–Meier analysis for all cases comparing patients with high and low aneuploid tumors. Clusters (high) is the −log10(p) of all the ColClusters for tumors with high aneuploidy while Clusters (low) indicates the *p* for the tumors with low aneuploidy. Overall is the *p* for all considered cases. *P* values were computed with the log-rank test. **b** Representative Kaplan–Meier curves showing the differences for high and low aneuploid tumors in ColClusters summarized in the heat map for two cancer types.

Aneuploidy is reported to have context-dependent impacts on tumors including unclear associations with overall survival^40^. The ColClusters associated with aneuploidy levels along with the SVM model identified relationships between aneuploidy and the collagen composition (Fig. 5). Evaluation of high and low aneuploid tumors in many ColClusters revealed associations with overall survival (Fig. 7), suggesting that collagen provides some context for patients’ outcomes with relatively high or low aneuploid levels .

For example, Bladder Urothelial Carcinoma (BLCA) tumors with high aneuploidy were separated by overall survival by collagen composition, while the low aneuploid BLCA tumors were not separated by overall survival. A large difference in overall survival between high and low aneuploid tumors was observed for BLCA-C4, the lowest overall survival BLCA ColCluster (Fig. 2). BLCA-C4 is marked by a combination of COL2A1, COL4A3, and COL11A2 among others. Similar observations were made for Liver Hepatocellular Carcinoma (LIHC), driven by the large difference in overall survival in LIHC-C3 between high and low aneuploid tumors. These observations identify contexts that distinguish high and low aneuploid tumors. Some collagen contexts did not have significant impacts on overall survival, maintaining a similar pattern compared to all tumors in the cancer type.

Other cancer types reveal different patterns of separation when combining aneuploidy and collagen composition. LUSC exemplifies how overall survival of high aneuploidy tumors is collagen composition dependent. High aneuploid tumors in LUSC-C4 have relatively lower risk while patients with high aneuploid tumors in LUSC-C5 have higher risk (Fig. 7). High aneuploid UCEC tumors have lower overall survival, however, UCEC-C4 strongly separates the high and low aneuploid tumors. All of the small number of low aneuploid tumors in UCEC-C4 remain alive (Fig. 7). These findings highlight how collagen composition influences tumors with high and low aneuploidy .

### Integrating the data

Considering the ColClusters with similar collagen composition as highlighted by being grouped into the same PanColClusters across cancer types (Fig. 1c), reveals new insights into these tumors. The range of molecular alterations and cell features in these groups highlights possible similar features to consider targeting across cancer types. For example, the high aneuploidy ColClusters, with relatively short OS, SARC-C4, STAD-C3, UCEC-C4, were grouped together in the Pan-Gyn, PanCan-C11 group, along with BRCA-C2 characterized by many copy number gains. Conversely, the longer OS STAD-C4 group mapped to the heterogeneous PanCan-C10 group with BLCA-C3, BLCA-C5, ESCA-C3, KIRP-C3, and OV-C2,C3; all with relatively lower levels of aneuploidy, marked by collagen type IX expression with lower fibrillar collagen expression. These findings suggest that classes of tumors originating from a range of tissues had high aneuploidy and similar collagen composition. A group of ColClusters in gastrointestinal (GI) tumors were enriched for tumors with lower levels of aneuploidy, high expression of fibrillar collagens including COL1A1, and yet also had relatively short overall survival, including COAD-C1, PAAD-C1, and STAD-C1.

We highlight a few ColClusters where combining the genetics, environment, and collagen composition clustering reveals new opportunities for therapeutic and biomarker development. STAD-C5 included a mixture of tumors with high and low aneuploidy classified together with similar collagen expression profiles. These tumors were enriched for Wnt Beta Catenin signaling hallmarks (Fig. 6). STAD-C5 had longer overall survival compared to the other STAD ColClusters.

BLCA-C1 and BLCA-C2 have similar expression of the fibrillar collagens and stroma fraction. BLCA-C2 is marked by COL17A1 expression and includes many squamous tumors. BLCA-C1 is enriched for EMT and angiogenesis hallmark gene sets while BLCA-C2 was enriched for 27 hallmark gene sets compared to 4 gene sets in BLCA-C1 as well as 5 gene sets with similar Qusage scores. BLCA-C5 is enriched for FGFR3 mutations and is highest for Notch hallmark gene sets. Notch may be a tumor suppressor pathway and is consistent with patients in BLCA-C5 having the longest overall survival. BLCA-C3 and BLCA-C4 are distinguished by a number of minor collagens and lower levels of fibrillar collagen expression. BLCA-C3 was enriched for bile acid metabolism, while BLCA-C4 was enriched for cell cycle regulation and had the shortest overall survival among the BLCA ColClusters. High aneuploidy tumors were significantly dispersed across many UCEC ColClusters. The high aneuploidy UCEC-C4 ColCluster is enriched for Notch signaling along with DNA repair and proliferation gene sets suggesting possibilities for therapeutic development in this class of tumor with a distinct combination of genetics, collagen composition, and tumor phenotypes.

## Discussion

In this study, we examined the impact of collagen expression to define tumor states. Clustering by collagen expression identified groups based on tissue specificity, cell states, immune environment, molecular alterations and overall survival. Collagen expression is strongly correlated with many molecular alterations including aneuploidy. Machine learning predicted these with high accuracy, particularly aneuploidy and chromosome copy numbers, based solely on collagen expression. Understanding the impact of genomic alterations on survival remains a major challenge for many cancer types. We found that combining collagen expression with molecular alterations improves predictions and tumor type definition. Features such as cell states are also critical for therapy response, and this study highlights the connections between the collagen content and specific cell states. Interestingly, ColClusters were marked by high COL1A1 and fibrillar collagen expression, or expression of minor collagens with relatively low COL1A1 expression. ColClusters defined by the expression of rare collagens improved associations with overall survival, molecular alterations, and were linked to specific pathways. Connections between collagen expression and molecular alterations helped identify tumors that are most impactful to patients. Many specific somatic mutations and aneuploidy are associated with either prolonged or diminished survival in various cancer types. Combining these molecular alterations with ColClusters defined by collagen expression improved associations with overall survival in TCGA (Fig. 7). Because of the large number of fascinating observations connecting collagens, molecular alterations and cell states, more detailed descriptions of some of the findings in each cancer type are summarized in the supplemental information.

Many collagens are known to be expressed in specific tissues. Classifying all the TCGA samples in a single dataset by collagen gene expression identified 15 tumor groups. These were often defined by tissue of origin and mapped very well to the published PanCancer groupings (Fig. 1). This is due in part to expression of many collagens that were confined to specific tumor types. For example, COL20A1 is highly expressed in neural systems and was also only expressed in neuronal tumors (Supplemental Fig. 3). Many minor collagens are only expressed in some cancer types and in only some subsets. Similarly, COL17A1 is expressed in both normal epithelial stomach cells and stomach adenocarcinoma. However, unlike COL20A1, COL17A1 is found in subsets of tumors in a number of cancers. Another possibility is that some collagens become expressed in tumors even though the normal tissue is distant from the tumor. For example, COL7A1 and COL10A1 are only expressed in very specific locations such as skin^49^ or developing cartilage^50^, respectively, and yet become dysregulated in a number of tumors including stomach^51^ and breast tumors^10^. These observations highlight how the combination of local specific tissue collagen expression and dysregulation of collagens in each tumor type defines unique tumor types and states. Understanding collagen and ECM composition helps interpret the features of tumors predicting therapy responses.

Fibroblasts, macrophages, cancer cells and other cell types all secrete collagens to create the complex tumor tissue structure. Because the ECM and collagen composition is the result of a complicated mixture of cells both secreting and remodeling, an ECM-collagen-based classifier may gain its power because it is the sum of the output of the tumor ecosystem, reflecting both cell composition and cell states. Many cells express and secrete the most abundant collagen, collagen type I. COL1A1 is present in every solid cancer type, but has >100 fold range in many instances, reflecting the variability of cell composition in heterogeneous tumors (Supplemental Fig. 3). Presence of relatively high levels of collagen type I have been associated with poor outcomes, including even GBM and LGG where COL1A1 is lower compared to most tumor types^52^ (Fig. 2) and (Supplemental Fig. 3). Tumors with high stroma fraction with expression of COL1A1 and related fibrillar collagens were often associated with shorter survival. Also notable is that ColClusters with higher stroma fraction and collagen type I, often C1 and C2 (Fig. 2) had distinct molecular alterations (Fig. 3 and Supplemental Fig. 17), including lower aneuploidy levels compared to other tumors in the same tissue in many cancer types (Fig. 5). Targeting stroma and related markers have become important to improve patient outcomes^53^. Stroma fraction has also been marked by Cancer Associated Fibroblasts (CAFs) in many tumors and associated with shorter survival^54^. Classifying tumors by collagens, representative of specific features of multiple cell types, is likely beneficial to predict the fate of disease progression.

High aneuploidy and ploidy levels are common to many cancer types, with a wide distribution across tumor specimens (Supplemental Fig. 10). A major complication is that these molecular alterations have a range of associations with overall survival in many cancer types (Fig. 7)^39^. The strongest association with molecular alterations across multiple cancers were between collagen expression and aneuploidy (Fig. 5). This observation may be because aneuploidy is more common relative to other somatic mutations in tumors in various cancer types. High and low aneuploidy levels were associated with specific ColClusters (Fig. 5). Combining aneuploidy with ColClusters refined interpretation of the impact of aneuploidy on overall survival (Fig. 7). In many cancer types aneuploidy was not associated with overall survival, and yet, aneuploidy combined with collagen composition identified tumor classes associated with survival that were not apparent when considering aneuploidy alone (Fig. 7). Combining aneuploidy with collagen composition defines tumor growth and treatment responses and could be further developed as diagnostic tool for patients.

Targeting the same pathway in multiple cancer types has been challenged by poor responses to many treatments across tissues, even when targeting the same molecular alteration^1^. This study suggests that the collagen environment contributes to the selection and survival of certain cancer cells defined by the same molecular alterations. There are other likely features in the collagen-based ECM that could further refine the tumor definition including post-translational modifications of collagens and remodeling of the matrix, often by proteolytic cleavage of collagens. Increased applications of scRNAseq and spatial transcriptome profiling hold the promise of increasing specificity to define tumors with even greater precision.

This study suggests that both highly expressed and dysregulated minor collagens mark multiple facets of tumors and could be useful biomarkers of the tumor ecosystem and disease progression. Attention should be paid to the collagen, and likely the full ECM, composition in pre-clinical in vitro and animal models to better represent the actual tumor microenvironment seen in patients that impact the functional consequence of molecular alterations, cell states, and probable responses to therapy. We envision that all manner of intervention including immunotherapy, targeted therapy, and chemotherapy can be better tailored to patients by considering the collagen and ECM milieu. In sum, the impact of the ECM and collagen has relationships with molecular alterations and infiltrating immune cells that could be considered to improve predictions of treatment outcomes. Taken together, these findings indicate that cancer cell state is associated with specific collagen-defined ECMs, implying that ECM state is a critical factor in properly targeting tumors.

## Methods

### Clustering and analysis

Only primary solid tumors were considered in this analysis. From a total set of 43 collagen genes, genes with significant expression (defined to be greater than 10 samples with an RSEM expression value of 200 or greater in the tumor type) were selected as features for clustering. Expression values were log2-transformed, and cancer cases were sub-typed using k-means clustering with Pearson’s correlation distance, for 3-6 clusters. Cluster number selection was informed by silhouette analysis and gap statistic comparison^18^. Colon (COAD) and rectal (READ) adenocarcinomas were clustered both separately and together as a combined colorectal adenocarcinoma tumor type (COADREAD). To characterize the molecular-level characteristics of each cluster, gene sets were selected from the Molecular Signatures Database (MsigDB), and clusters were compared to each other using quantitative set analysis for gene expression (QuSAGE)^46^. This analysis was supplemented with single-sample gene set enrichment analysis (ssGSEA)^55^.

### Support vector machine models

To assess the relationship between collagen expression and aneuploidy, we trained a linear support vector machine model for each TCGA tumor type with the scikit-learn v0.24.2 machine learning package for Python. Normalized collagen RSEM expression scores and stromal fraction were used as initial input features. Feature selection was performed by removing insignificantly-expressed collagens and lesser-contributing (as defined by low relative SVM weight) collagens. Labels (high and low) for each sample were generated by fitting each aneuploidy score distribution to a mixture of two Gaussian distributions. 5-fold cross-validated models were evaluated with area under the receiver-operator curve (AUROC) scores. The same pipeline was used to separately predict chromosome arm copy number gains and losses for copy number modifications with sufficient counts (>10 copy number modifications within the tumor type).

### Statistical analysis

The majority of the data processing and statistical analyses were performed in Python v3.11.0. Kaplan–Meier and Cox survival analysis, based on clinical data from the PanCancer Atlas was used to compare overall survival between clusters, using the Python lifelines v0.26.0 and R survival v3.2-7 packages respectively^56,57^. The log-rank test was performed on the resulting Kaplan–Meier survival models to assess differences in overall survival within tumors. Categorical variables were compared with Pearson’s Chi-squared test. Unless otherwise stated, all comparisons for continuous variables were performed with the two-sided Kolmogorov-Smirnov test. Where applicable, *, **, *** denote *p* values of <0.05, 0.01, and 0.001 respectively.

### Reporting summary

Further information on research design is available in the Nature Research Reporting Summary linked to this article.

## Supplementary information

Supplemental File

Supplementary Table 2

Supplementary Table 3

Reporting Summary

## Data Availability

Processed sequence and clinical data, including aneuploidy, stromal fraction, and mutation data from The Cancer Genome Atlas are available from NCI Genome Data Commons at https://gdc.cancer.gov/about-data/publications/pancanatlas(Supplemental Table 2). All the data generated in this study are available in the manuscript text and/or the supplementary information.

## Code availability

The underlying code for this study is not publicly available but is based on publically available software packages and may be made available to qualified researchers on reasonable request from the corresponding author. Kaplan–Meier and Cox survival analysis was performed with the Python lifelines v0.26.0 and R survival v3.2-7 packages respectively^56,57^. R (v4.0.1) was also used to perform ssGSEA (v10.0.0.1) and QuSAGE (v2.22.0). Graphs and heatmaps were generated using the Seaborn v0.11.1 data visualization library for Python. Circular heatmaps were generated with circlize v0.4.13 in R.
