# Supplementary material for: Tumor collagens predict genetic features and patient outcomes
Source: NPJ Genom Med. 2023 Jul 6;8:15. doi: 10.1038/s41525-023-00358-9 (PMC10326062; doi:10.1038/s41525-023-00358-9)

Supplementary Discussion: Cancer Type Summaries

For each cancer type, we present a brief synopsis of the major observations that could guide marker development and emphasize how the classification of tumors by collagen expression is associated with genetics, molecular and cellular phenotypes.

Bladder Urothelial Carcinoma (BLCA)

BLCA-C1 and BLCA-C2 have similar expression of the fibrillar collagens and stroma fraction. BLCA-C2 is marked by COL17A1 expression and includes many squamous tumors (**Supplemental Figure 14**). BLCA-C1 is enriched for EMT and angiogenesis hallmark gene sets while BLCA-C2 was enriched for 27 hallmark gene sets compared to 4 gene sets in BLCA-C1 with 5 gene sets with similar Qusage scores. BLCA-C5 is enriched for FGFR3 mutations and is highest for Notch hallmark gene sets (**Figure 3**). Notch may be a tumor suppressor pathway and is consistent with patients in BLCA-C5 having the longest overall survival. BLCA-C3 and BLCA-C4, are distinguished by a number of minor collagens and relatively lower levels of fibrillar collagen expression. BLCA-C3 was enriched for bile acid metabolism, while BLCA-C4 was enriched for cell cycle regulation and had the shortest overall survival among the BLCA ColClusters. 8p loss in all clusters except BLCA-C5 (**Figure 4**). Neuroendocrine tumors were grouped in BLCA-C4 and enriched in BLCA-C5. Papillary and non-papillary forms were biased across ColClusters (p=5e-10). Low aneuploidy BLCA tumors, but not high aneuploidy, in the ColClusters were associated with overall survival (**Figure 7**). Because BLCA includes a variety of known histologies, we tested enrichments of the reported histology and previously assigned mRNA based classifications and found strong enrichment in the ColClusters further linking collagen expression with known classifications and histologies (**Supplemental Figure 15**). Some ColClusters were strongly enriched for specific somatic mutations (**Figure 3**). P53 missense mutations were enriched for BLCA-C2 and C4. RB1 truncations were biased towards BLCA-C1, C2, and C4, being nearly completely absent in BLCA-C3 and C5. FGFR3 missense mutations were strongly enriched in BLCA-C5 (**Supplemental Figure 14**), with longer overall survival compared to the other ColClusters (**Supplemental Figure 4**).

Breast invasive carcinoma (BRCA)

BRCA-C3 was highest for fibrillar collagen expression while BRCA-C1 was enriched for a number of collagens. BRCA-C2 and BRCA-C5 were marked by COL2A1 expression, with expression of COL4A3/4A4, COL9A1/COL9A3 and COL11A1 discriminating BRCA-C2. BRCA-C4 also had relatively high COL9A1, COL9A3, and COL11A2 expression, but low COL2A1 expression (**Supplemental Figure 14**). BRCA ColClusters were not significantly associated with overall survival (Figure 2). This is likely because of the long survival times achieved by many patients. KMT2C truncation, PIK3CA missense, TP53 missense, and TP53 truncation variants were significantly biased across BRCA ColClusters. (**Figure 3**) TP53 was localized to BRCA-C2 and BRCA-C4 while PIK3CA was enriched in BRCA-C1 and C3. ER+ tumors were present in all 5 BRCA ColClusters, but triple negative tumors dominated BRCA-C4 while ER+/HER2+ tumors were prominent in BRCA-C3. These findings suggest that similar ECMs were somewhat independent of hormone and HER2+ status. A number of chromosome arms were enriched in ColClusters including 6p gain in BRCA-C2 and losses in 12q, 14q, and 15q, in BRCA-C2 and BRCA-C4.

16p gain was enriched in BRCA-C1, C3, and C5. Pathways to consider for targeting in specific collagen environments include DNA repair, E2F, and Myc in BRCA-C2 and BRCA-C4, consistent with higher proliferation in tumors with more chromosome loss. BRCA-C2 and BRCA-C4 were enriched for Myc and Rad21 amplification (**Figure 4**), along with proliferation hallmarks including E2F targets, hypoxia, Myc targets, PI3K AKT mTOR signaling, and Wnt beta Catenin (**Figure 6**). EMT was highest in BRCA-C3 which is marked by high COL10A1 expression. Notch hallmark gene set was highest in BRCA-C5.

Cervical squamous cell carcinoma and endocervical adenocarcinoma (CESC)

CESC ColClusters were marked by a high fibrillar collagen group, CESC-C1. CESC-C2 is marked by COL4A5/A6, COL7A1, COL16A1, COL17A1 and is enriched for squamous carcinomas. CESC-C3 was marked by collagen type IX and was associated with the longest overall survival, while CESC-C1 and C2 had similar overall survival curves. CESC-C1 and C3 were enriched for missense mutations in PIK3CA (**Figure 3**), which as the only identified biased significantly enriched somatic mutation. Low frequency amplifications in CCND1, EGFR, the FGF locus and TERT were enriched in CESC-C2 (**Figure 4**). A number of chromosome arm level CNAs were enriched in CESC-C2 compared to the other two CESC ColClusters. These include the infrequent 1p, 4q, 19p loss along with the more frequent 5p gain and 8p loss. 20p and 20q gain were enriched in CESC-C1. 18p gain was enriched in CESC-C3. CESC-C2 was enriched for 31 hallmark gene sets including DNA repair. CESC-C1 was enriched for Notch signaling and angiogenesis. CESC ColClusters had similar immunotype profiles with a mixture of "Wound Healing" and "IFN-γ" (**Figure 6**). CESC-C2 was enriched for a number of immune cell types including gamma delta T cells, neutrophils, and T helper cells (**Supplemental Figure 13**). Effector memory T (Tem) cells were enriched in CESC-C1. CESC ColClusters had similar distributions of aneuploidy (**Figure 5**).

Colon adenocarcinoma (COAD)

COAD ColClusters were associated with overall survival with COAD-C4 marked with the longest surviving patients (**Supplemental Figure 4**). COAD-C1 was marked by high expression of fibrillar collagens. COAD-C2 was marked by COL9A1. High expression of COL2A1 and COL9A3 defined COAD-C3. COL4A5/6 marked COAD-C4. MSIH tumors were enriched in COAD-C1. KRAS mutations were biased towards COAD-C2 and COAD-C3. APC truncations were enriched in COAD-C3. None of the evaluated gene level CNAs were biased in the COAD ColClusters. COAD-C2 was enriched for high aneuploid tumors (**Figure 5**) which also manifested in chromosome arm level CNAs enriched in COAD-C2 (**Figure 4**). Hallmark gene sets were highest in COAD-C1 including strong enrichment of EMT, Hedgehog Signaling, Hypoxia, and the inflammatory gene sets (**Figure 6**). The Wnt signaling hallmark was enriched in COAD-C3. Peroxisome and protein secretion were enriched in COAD-C4 (**Figure 6**). COAD-C2, C3, and C4 were enriched for the "Wound Healing" immunotype (**Figure 6**). COAD-C1 was distributed between "Wound Healing" and "IFN-γ". COAD-C1 was enriched for the majority of immune cell signatures tested (**Supplemental Figure 13**). COAD-C3 was enriched for activated CD8 T cells while COAD-C2 and C3 were not enriched for any immune cell signatures. The SVM predicted aneuploidy well with AUC=0.77 (**Figure 5**). A notable finding is that COAD tumors with similar collagen composition include both MSS and MSI tumors (**Figure 3**) (**Supplemental**

Figure 14). Larger cohorts can be evaluated by their collagen composition to further distinguish the MSS and MSI forms.

Colorectal adenocarcinoma (COADREAD)

Four COADREAD ColClusters were identified and were significantly associated with overall survival (**Figure 2**) and (**Supplemental Figure 4**). COADREAD-C1 and C2 were both defined by high fibrillar collagen expression with COADREAD-C2 showing a little bit lower average expression of each fibrillar collagen (**Supplemental Figure 14**). COL9A1 expression in COADREAD-C2 and COL9A2 expression in COADREAD-C1 also discriminated these two ColClusters. Relatively high COL2A1 expression defined COADREAD-C3. Relatively high COL4A5/6 expression defined COADREAD-C4. COL9A2, COL9A3, COL11A2, and COL28A1 were also relatively high in COADREAD-C4. Most MSI tumors were in COADREAD-C1 (**Supplemental Figure 14**) and (**Supplemental Figure 7**). KRAS missense mutations were mildly enriched in COADREAD-C2 and C4 (**Figure 3**). A number of low frequency somatic mutations showed mild biases across the ColCluster. No gene level CNAs were enriched in specific ColClusters (**Figure 4**). A number of COADREAD ColClusters were specifically enriched for chromosome arm level CNAs. In particular, COADREAD-C1 was not as enriched as other ColClusters for CNAs. Notably, even though COADREAD-C2 and C3 were very different in their collagen expression, they had similar chromosome arm level CNAs including 8p, 18p and 18q losses and 20q gains. 1p and 14q loss, 2p and 2q gain were specific for COADREAD-C3. SVM predicted chromosome arm level CNAs (**Figure 5**). COADREAD-C1 was enriched for the majority of hallmark gene sets by QuSAGE, including the inflammation related hallmarks (Allograft Rejection, Inflammatory Response, etc.) (**Figure 6**). COADREAD-C2, even with lower overall levels of fibrillar collagen, was enriched for EMT as well as Hedgehog Signaling. COADREAD-C3 was enriched for Wnt Beta Catenin Signaling. COADREAD-C4 was enriched for Oxidative Phosphorylation. No ColCluster was enriched for many of the proliferation related hallmarks. COADREAD-C2,C3, and C4 were enriched for the "Wound Healing" immunotype, while COADREAD-C1 included tumors with both "Wound Healing" and "IFNγ" (**Figure 6**). COADREAD-C1 was also enriched for multiple immune cell signatures by Qusage including Cytotoxic cells, Macrophages, and Neutrophils (**Supplemental Figure 13**). COADREAD-C2 was enriched for CD56dim cells, suggesting a more immunocompetent environment. COADREAD-C3 was enriched for Activated CD8 T cells and Effector Memory T cells. COADREAD-C4 was relatively low for immune cell signatures. Aneuploid tumors were enriched in all the ColClusters except COADREAD-C1 (**Figure 5**). Aneuploidy was associated with overall survival in COADREAD (**Figure 7**). Stratification by aneuploidy showed that tumors with low aneuploidy in the ColClusters were significantly associated with outcomes, but not tumors with high aneuploidy. However, collagen expression combined with SVM did not predict aneuploidy well with AUC=0.61 (**Supplemental Figure 12**). In sum, MSI tumors were localized to specific ColClusters and the relatively modest differences in collagen expression between COADREAD-C1 and C2 were associated with overall survival, distinct phenotypic states, and immunoenvironments. COADREAD-C3 and C4 were defined by expression of specific collagens.

Esophageal carcinoma (ESCA)

ESCA ColClusters were not significantly associated with overall survival (**Supplemental Figure 4**). ESCA was distinguished from other cancers in the PanCancer clustering by the expression of COL17A1, a squamous cell marker (**Figure 2**)[1]. Stroma fraction was very similar across all the ESCA tumors (**Figure 2**B). ESCA-C1 was defined by modest fibrillar collagen expression, notably COL5A1/2 with COL4A3/4 expression. ESCA-C2 was defined by COL4A5/6 expression. Collagen type IX was highly expressed in ESCA-C1, C2, and C3. ESCA-C3 was marked by a combination of low fibrillar collagen, high COL4A3/4, and high collagen type IX expression. ESCA-C4 was defined by the high expression of the fibrillar collagens and was enriched for macrophages and regulatory T cells, suggesting a more immunosuppressive environment. Dendritic cells were also enriched in ESCA-C4 (**Supplemental Figure 13**). ESCA-C2 and ESCA-C4 were lowest for cytotoxic T cell signatures. Relatively low frequency somatic mutations including KMT2D truncations in ESCA-C2, LRP1B in ESCA-C1, C2 and C3, and PREX2 missense in ESCA-C3 show significant bias in ESCA ColClusters (**Figure 3**). NF1 truncations were enriched in ESCA-C1. ESCA-C2 and ESCA-C4 were enriched for CNAs in a number of oncogenes and suppressors, but were not so different in chromosome arm level CNAs compared to the other ColClusters (**Figure 4**). ESCA-C4 was enriched for the most hallmark gene sets. ESCA-C3 was enriched for the KRAS signaling gene set (**Figure 6**). No differences in aneuploidy were observed across the ESCA ColClusters, but collagen expression did predict aneuploidy levels using SVM (**Figure 5**). Although aneuploidy was not associated with overall survival across all cases, high aneuploidy tumors in ESCA-C1 had significantly shorter overall survival (**Figure 7**).

Glioblastoma multiforme (GBM)

The four GBM ColClusters were not significantly associated with overall survival. GBM ColClusters were not as well-defined as ColClusters in other cancer types (**Supplemental Figure 1**), likely because of the smaller number of available tumors (**Supplemental Table 3**). GBM-C1 is the high fibrillar collagen expression ColCluster (**Supplemental Figure 14**). GBM IDH1 mutant tumors were grouped with LGG tumors in the PanCan collagen clustering, suggesting similar ECM environments, that were distinct from IDH1 wild-type tumors. This may be consistent with the known differences in GBM IDH1 mutant tumors which is now used to define a specific tumor type[2]. Some IDH1 WT GBM tumors were also included in PanColCluster-C12 with the IDH1 mutant tumors, in GBM-C3 and C4, suggesting possible similar microenviroments and treatment responses. GBM-C1 was enriched for multiple immune cell expression signatures including macrophages, mast cells, neutrophils, and T regs. The other GBM ColClusters were not as infiltrated with immune cells. All GBMs were in the "Lymphocyte Depleted" group (**Figure 6**). TP53 alterations were enriched in GBM-C2 and C3 (**Figure 3**). Specific GBM ColClusters were enriched for a number of arm level CNAs including 22q loss, 13q loss, 14q loss in GBM-C2. 9p loss along with gains in 19p, 19q, 20p and 20q were enriched in GBM-C4. GBM did not show significant aneuploidy score biases (**Figure 5**). Aneuploidy could be predicted from collagen expression with AUC=0.8, but this prediction may not be strong due to the modest number of high aneuploid tumors in the TCGA GBM cohort (**Figure 5**). SVM did predict chromosome arm level CNAs suggesting a connection between collagens and specific genetic alterations (**Figure 5A**).

Head and Neck squamous cell carcinoma (HNSC)

Three HNSC ColClusters were identified and associated with overall survival. HNSC-C1 had significantly, though only modestly higher stroma fraction (**Figure 2**B). HNSC-C3 was associated with longer overall survival. HNSC-C1 is the high fibrillar collagen expression ColCluster. HNSC-C2 has relatively low collagen expression, while HNSC-C3 tumors were enriched for COL4A3/4, collagen type IX, COL19A1, COL21A1, and COL23A1 (**Supplemental Figure 14**) and (**Supplemental Figure 3**). HNSC-C1 and C2 were enriched for P53 missense and truncation variants. Relatively low frequency NSD1 truncations were enriched in HNSC-C2 (**Figure 3**). CDNK2A truncations and FAT1 truncations were enriched in HNSC-C1 and HNSC-C2 and were largely absent from HNSC-C3. HNSC-C3 was enriched for PTEN loss. Copy number gains in EGFR, and losses in CDKN2A and CDKN2B, were enriched in HNSC-C1 and C2. HNSC-C1 and C2 were enriched for a similar pattern of chromosome arm level CNAs including losses in 3p, 4p, 4q, 8p, 9p, 18q and 21q along with gains in 7p and 8q, suggesting genetic similarity. HNSC-C3 was enriched for gains in 8p, 18q, 19p, and 19q and losses in 11q, and 16q suggesting a genetically distinct group of tumors. HNSC-C1 was strongly enriched for EMT, Angiogenesis, and Myogenesis, while HNSC-C2 and C3 were strongly negatively enriched for these hallmarks (**Figure 6**). Hedgehog Signaling was biased towards HNSC-C1. HNSC-C2 was enriched for Hypoxia, Glycolysis, MTORC Signaling, Myc Targets, Oxidative Phosphorylation, and P53 Signaling. HNSC-C3 was negatively enriched for many hallmarks but positively enriched for E2F targets, suggesting a distinct mechanism of proliferation compared to the tumors in the other ColClusters. HNSC tumors were largely in the IFNγ immunotype (**Figure 6**), with HNSC-C1 also including some tumors with "Wound Healing". HNSC-C1 appears to be more immunosuppressive with enrichment for signatures for Eosinophils, Macrophages, Neutrophils, and Regulatory T cells (**Supplemental Figure 13**). HNSC-C2 and C3 included more immunocompetent environments, in particular HNSC-C3 with enrichment of Activated CD 8 T cells, B Cells, and Cytotoxic cells. HNSC-C3 was enriched for tumors with lower levels of aneuploidy (**Figure 5**). HNSC-C1 and HNSC-C2 had relatively higher levels of aneuploid tumors compared to HNSC-C3 (**Figure 5**). The SVM predicted aneuploidy with moderate success (AUC=0.73). Stratification by aneuploidy revealed that ColClusters for tumors with low aneuploidy were significantly associated with overall survival, but ColClusters for tumors with high aneuploidy were not, having similar overall risk (**Figure 7**).

Kidney renal clear cell carcinoma (KIRC)

Three KIRC ColClusters were identified and significantly associated with overall survival with KIRC-C1 associated with the shortest and KIRC-C2 the longest overall survival (**Supplemental Figure 4**). KIRC-C1 is the high fibrillar collagen expression group and had much higher stroma fraction compared to the other Colclusters (**Figure 2**B). PBRM1 truncations were localized to KIRC-C1 and C2. This was the only somatic mutation showing bias in KIRC of the tested genes. Some KIRC-C3 tumors were in the Lymphocyte depleted immunogroup (**Figure 6**). KIRC-C1 was enriched for the most hallmark gene sets. KIRC-C3 was enriched for Oxidative Phosphorylation, along with DNA Repair, G2M Checkpoints and Apical Surface hallmark gene sets. Notch and Hedgehog Signaling gene sets were enriched in KIRC-C2 (**Figure 6**). KIRC-C3 was also lower in a number of immune cell signatures (**Supplemental Figure 13**). Neutrophil expression signatures were enriched in KIRC-C2 while a

number of immune cell signatures were enriched in KIRC-C1. KIRC-C2 has lower aneuploidy compared to the other KIRC ColClusters (**Figure 5**), and the SVM predicted aneuploidy well at AUROC=0.78 in KIRC (**Figure 5**). From the collagen composition, a number of chromosomes were predicted by SVM (**Figure 5A**).

Kidney renal papillary cell carcinoma (KIRP)

Six KIRP ColClusters were identified and were strongly associated with overall survival (**Supplemental Figure 4**). KIRP-C1 and C3 were highest for fibrillar collagen expression. KIRP-C2 was marked by high expression of COL2A1 and COL22A1. Collagen type IV expression was a key determinant of KIRP ColClusters. KIRP-C3 had high expression of COL4A5/6 while KIRP-C1 was highest for COL4A1/2 expression (**Supplemental Figure 3**). KIRP-C5 was marked by high expression of COL4A5/6. KIRP-C5 had low expression of both fibrillar and type IV collagens, but relatively high expression of collagen type IX. The only significantly biased gene mutation was Met missense variants, enriched in KIRP-C4 and C5 (**Figure 3**). Some common gene CNAs were observed including CDK6 and EGFR gains enriched in KIRP-C2, C4, C5, C6. CDKN2A, CDKN2B, and MTAP losses and CCND1 gains had similar patterns enriched in KIRP-C3. KIRP-C3 stands out as distinct when compared to the other KIRP ColClusters (**Figure 4**). KIRP-C3 was highest for regulatory T cells. KIRP-C4, the ColCluster with the smallest HR, was highest for Mast cells. Reactive Oxygen Species hallmark gene set was highest in KIRP-C1 and C2 and lowest in C4. KIRP-C2 was enriched for Oxidative Phosphorylation and Interferon Gamma Response gene sets while also relatively high in Adipogenesis and low in Angiogenesis. KIRP-C4 was enriched in Cholesterol Metabolism and IL2 Stat5 Signaling. KIRP ColClusters did not show biases for aneuploidy scores (**Figure 5**). These data identified KIRP-C3 as a group of tumors with very short overall survival, high in a type of fibrillar collagen expression that differs from the other KIRC ColClusters and has specific genetics. KIRP-C2, C4, C5, and C6 are tumors with distinctive genetics, immunoenvironments, and pathways that have longer overall survival through different mechanisms.

Brain Lower Grade Glioma (LGG)

Five LGG ColClusters were identified strongly associated with overall survival (**Supplemental Figure 4**). LGG-C1 and C2 had the highest Hazard Ratios (HR). Fibrillar collagen expression was highest in LGG-C2 (**Supplemental Figure 14**). Also of note is that a fraction of LGG-C2 tumors had similar collagen composition as GBM tumors (**Figure 2**E), with IDH1 wild-type and similar immunoenvironments (**Figure 6**). LGG-C1 was marked by a combination of expression of COL6A6, COL8A1, COL19A1, COL21A1, COL23A1, COL24A, and COL25A1. The overall mutation rates was similar across all the ColClusters. TP53 alterations were particularly enriched in LGG-C4, with mild enrichment in LGG-C2 and C3 (**Figure 3**). IDH1 missense alterations were enriched in LGG-C3, C4, and C5 with much lower levels in LGG-C1 and C2. Low frequency EGFR missense mutations were enriched in LGG-C1 and C2. ATRX truncations were enriched in LGG-C3 and C4. A number of genes were enriched in specific LGG ColClusters (**Figure 4**). MTAP and p16/CDKN2A copy number loss and EGFR copy number gains were enriched in LGG-C1 and C2. Lower frequency enrichments of SOX2 gains were in LGG-C2. Chromosome arm level CNAs also showed significant enrichments in specific ColClusters (**Figure 4**). 19q losses were strongly enriched

in LGG-C5 and mildly enriched in LGG-C1 and C3. Together, these observations connect molecular alterations to specific collagen compositions. LGG-C1 and C2, highest in collagen expression, and enriched for many known important molecular alterations had shorter overall survival compared to the other LGG ColClusters. LGG-C1 and C2 were each enriched for the largest number, 19, of hallmark gene sets, including both EMT and proliferation gene sets such as E2F targets (**Figure 6**). The other LGG ColClusters showed distinct enrichment patterns. LGG-C1 was enriched for Fatty Acid Metabolism, Myogenesis, Oxidative Phosphorylation, LGG-C3 was enriched for Adipogenesis, Reactive Oxygen Species, and Xenobiotic Metabolism gene sets. LGG-C3 and C4 had relatively lower levels of aneuploidy, and the SVM predicted aneuploidy with high accuracy (**Figure 5**). LGG-C2 was marked by relatively high COL1A1 expression, comparable to GBM tumors, though still much lower than the large majority of other cancer types (**Supplemental Figure 3**). The higher aneuploid tumors in LGG-C2 had much shorter overall survival (**Figure 7**).

Liver hepatocellular carcinoma (LIHC)

Three LIHC ColClusters were not significantly associated with overall survival (**Supplemental Figure 4**), but were quite distinct in collagen composition, molecular alterations, and phenotypes. LIHC-C1 had the highest expression of fibrillar collagens. LIHC-C2 showed high expression of COL2A1 and COL11A2 (**Supplemental Figure 14**). LIHC-C3 was defined by generally lower collagen expression and modestly higher expression of COL4A5/6, COL5A3 and COL7A1. For the evaluated genes, gene level CNAs were not significantly biased across LIHC ColClusters for the common CNAs including MYC and NOTCH2. Chromosome arm level CNAs were particularly enriched in LIHC-C2 (**Figure 4**). LIHC-C2 was enriched for a number of chromosome arm level copy gains including 19q, 20p, and 20q losses in 1p, 4q, 8p, 9p, and 16q. A number of specific chromosome arm level CNAs were predicted by SVM (**Figure 5**). CTNNB1 alterations were strongly enriched in LIHC-C3 (**Figure 3**). Each of the 3 LIHC ColClusters have distinct immunotypes (**Figure 6**). LIHC-C1 was high in "Inflammatory", LIHC-C2 was a mixture of "Inflammatory" and "Lymphocyte Depleted" and the majority of LIHC-C3 tumors were "Lymphocyte Depleted". LIHC ColClusters were enriched for 25, 9, and 16 hallmark gene sets, respectively. Wnt beta catenin signaling hallmark gene set was enriched in LIHC-C2 (**Figure 6**). Inflammatory and angiogenesis gene sets were enriched in LIHC-C1. DNA Repair and proliferation gene sets were enriched in LIHC-C2. Cholesterol metabolism, Oxidative phosphorylation, and Reactive Oxygen Species gene sets were enriched in LIHC-C3. These observations suggest that ECM defined LIHC groups have distinctive features to target. LIHC ColClusters did not show biases for aneuploidy scores (**Figure 5**). But the LIHC tumors with high aneuploidy scores were distinct in their differential association with overall survival while LIHC tumors with lower aneuploidy, even with distinct collagen composition had similar overall survival (**Figure 7**).

Lung adenocarcinoma (LUAD)

The four LUAD ColClusters were significantly associated with overall survival with LUAD-C1 having the shortest overall survival. Fibrillar collagen expression was highest in LUAD-C1 (**Supplemental Figure 4**). LUAD-C2 was marked by high COL4A3/4/5/6 and COL6A6 expression. LUAD-C3 was marked by COL25A1 expression and LUAD-C4 was marked by

COL2A1 and COL11A2 expression. P53 missense and truncation variants were enriched in LUAD-C1 and C4 with the fewest fraction of tumors with P53 alterations in LUAD-C3. LUAD-C3 was enriched for KRAS missense variants, although significant numbers of tumors with KRAS missense mutations were in each LUAD ColCluster. Lower frequency alterations including LRP1B missense mutations were enriched in LUAD-C1 and C4. Copy number gains in CDK4, EGFR, SOX2, TERC, and TERT were enriched in LUAD-C4. Gains in Myc were biased to LUAD-C3, which also was highest for the Myc Targets hallmark gene set (**Figure 6**). LUAD-C1 was enriched for the most hallmarks (**Figure 6**). LUAD-C2 was enriched for a number of inflammation gene sets including Interferon Alpha Response and Interferon Gamma Response as well as the P53 pathway. LUAD-C4 was enriched for the E2F targets, G2M checkpoints and DNA repair gene sets. LUAD-C3 was enriched for Xenobiotic Metabolism, Unfolded Protein Response, Oxidative Phosphorylation and Fatty Acid Metabolism. LUAD tumors have diverse immunoenvironments in the ColClusters (**Figure 6**). LUAD-C1 and C4 were enriched for "Wound Healing" and IFN-γ while LUAD-C2 and C3 were enriched for "Inflammatory" with some tumors enriched for "IFN-γ". Central and effector memory T cells were enriched in LUAD-C2 and C3 (**Supplemental Figure 13**). Regulatory T cells were enriched in LUAD-C1. LUAD-C2 was enriched for lower aneuploidy while LUAD-C4 was enriched for higher aneuploidy (**Figure 5**B). SVM predicted aneuploidy scores based on collagen expression with high accuracy (**Figure 5**C). These observations highlight collagen composition associated with the large range of molecular alterations and treatments responses in TCGA.

Lung squamous cell carcinoma (LUSC)

The six LUSC ColClusters were not associated with overall survival (**Supplemental Figure 4**). LUSC-C1,C2, and C3 had similar stroma fractions, differing from lower stroma fraction LUSC-C4 and C5 (**Figure 2**). LUSC-C1, C2, and C3 were all high in fibrillar collagen expression and discriminated by expression of COL4A3/4 (LUSC-C2), collagen type IX (LUSC-C2 and C3), COL19A1 (LUSC-C2) as well as COL21A1, COL22A1, and COL23A1 (**Supplemental Figure 14**). LUSC-C4 was marked by low fibrillar collagen expression, high COL4A5/6, and COL21A1. LUSC-C5 was marked by low fibrillar collagen expression, high expression of COL4A5/6, COL17A1, COL27A1, and COL28A1. LUSC ColClusters have biased distributions of low frequency somatic mutations in PTEN, PTPRB, PTPRT, and RB1 (**Figure 3**). Genes with high frequency CNAs in LUSC were enriched in all the ColClusters except LUSC-C4 (**Figure 4**). LUSC-C4 was enriched for losses in 22q and 19p. LUSC-C3 was enriched for losses in 18q while LUSC-C2 was enriched for losses in 14q. These CNAs define the genetic-ECM relationships for LUSC. No biases were observed for aneuploidy scores in LUSC. LUSC-C1 and C3 were each enriched for a large number of hallmarks (**Figure 6**). LUSC-C2, also relatively high in fibrillar collagen expression however, was enriched for only the Allograft Rejection gene set. Along with LUSC-C4, LUSC-C2 was enriched for B cells, Cytotoxic cells (**Supplemental Figure 13**). LUSC-C4 was also enriched for Macrophages. These observations suggest distinct immunoenvironments in each ColCluster. LUSC ColClusters showed no biases for aneuploidy scores, nor could collagen expression predict aneuploidy robustly in LUSC (**Figure 5**). High aneuploidy LUSC tumors had distinct relationships with overall survival in LUSC-C4 and C5 (**Figure 7**). In sum, the collagen composition defines distinct LUSC tumor types and relationships for candidate treatments.

Ovarian serous cystadenocarcinoma (OV)

Three OV ColClusters were identified that were not associated with overall survival (**Supplemental Figure 4**). Stroma fraction was similar between OV-C1 and C2, with OV-C3 being significantly lower (**Figure 2**B). OV-C1 tumors had higher fibrillar expression compared to the other OV ColClusters. OV-C3 was marked by high COL2A1, COL4A5/6 and COL9A3 expression. OV-C2 has relatively low collagen expression. No somatic variants evaluated showed significant enrichments in an OV ColCluster (**Figure 3**). Some chromosomes had strong biases in ColClusters marked by collagen environments. 2p gain was enriched in OV-C3. 12q gain and 9p loss were enriched in OV-C2 and C3. 8p loss was enriched in both OV-C1 and C3 (**Figure 4**). OV-C1 was enriched for 38 of the 50 hallmark gene sets (**Figure 6**). OV-C3 was the most enriched for EMT, Notch, Wnt Beta Catenin signaling, suggesting connections between these signaling pathways and the distinct ECM of these tumors. OV ColClusters showed no biases for aneuploidy scores, nor could collagen expression predict aneuploidy robustly (**Figure 5**). These findings suggest that even for a very heterogeneous copy number driven cancer type such as OV, distinct connections between the genetics and tumor ECM can be identified. Combining the collagen expression and molecular alterations does distinguish OV tumors to consider for treatment options.

Pancreatic adenocarcinoma (PAAD)

PAAD ColClusters were significantly associated with overall survival with PAAD-C4 distinct from the other three ColClusters with similar risk (**Figure 2**) and (**Supplemental Figure 4**). PAAD-C1 and C2 were both marked by high fibrillar collagen expression and distinguished by differences in COL4A3/4 expression along with differences in COL10A1. Some fibrillar associated collagens had distinct expression patterns between PADD-C1 and C2. COL11A1 had higher expression in PAAD-C2, while COL141 and COL15A1 had higher expression in PAAD-C1 (**Supplemental Figure 14**). PAAD-C3 was marked by high expression of COL9A2, COL9A3, and COL11A2. PAAD-C4 was marked by high expression of COL2A1, COL4A6, and COL25A1. Because PAAD-C1 includes the very high stroma fraction, and lower tumor cell fraction, these tumors were underrepresented for KRAS and TP53 variants as reported[3]. TP53 and KRAS variants were notably absent in the long surviving PAAD-C4 group (**Figure 3**). PAAD-C1 was notably not enriched for chromosome arm level CNAs (**Figure 4**). PAAD-C2 and C3 had similar patterns of chromosome level CNAs, mostly chromosome arm copy losses. On the other hand, PAAD-C4 had a number of chromosome level copy number gains. Many of the chromosome arm level CNAs were predicted from collagen expression by SVM (**Figure 5**). PAAD-C2 was enriched for the most hallmark gene sets (**Figure 6**B). Even though both PAAD-C2 and C2 had high expression of fibrillar collagens, only PAAD-C1 was enriched for the TGFb gene set. PAAD-C3 was enriched for Cholesterol Metabolism and Oxidative Phosphorylation. The one ColCluster with long overall survival, PAAD-C4, was negatively enriched for many hallmarks with just spermatogenesis positively enriched (**Figure 6**B). Aneuploidy scores are borderline significantly biased across PAAD ColClusters (**Figure 5**), but the SVM performed poorly to predict aneuploidy, perhaps for similar reasons of the only modest separation of the k-means defined ColClusters (**Supplemental Figure 1**). PAAD ColClusters were distributed across multiple PanColClusters reflecting the large range of collagen composition that was most similar to a variety of other tumor types. PAAD-C1 was mostly in PanColCluster-C3, LUAD with LUSC/THCA tumors.

PAAD-C2 and C3 were most enriched in PanColCluster-C2, Pan-GI. PAAD-C4 was distributed among multiple PanColClusters. In sum, pancreatic tumors are very heterogeneous, and yet specific links exist between collagen expression and combinations of chromosome level arm CNAs.

Pheochromocytoma and Paraganglioma (PCPG)

Four PCPG ColClusters were identified and were not associated with overall survival as the large majority of patients live a long time (**Figure 2** and **Supplemental Figure 4**). PCPG-C1 and C2 were enriched for fibrillar collagen expression with PCPG-C1 marked by the neuronal collagen, COL20A1 (**Supplemental Figure 14**). PCPG-C4 was marked by relatively low fibrillar collagen expression and high COL20A1 expression. PCPG-C3 was marked by a combination of low fibrillar collagen expression and high COL4A5/6 expression. Low frequency NF1 truncations were enriched in PCPG-C4 (**Figure 3**). Low frequency HRAS missense variants were enriched in PCPG-C3 and C4. None of the evaluated genes showed CNA enrichments (**Figure 4**). A few chromosome arm level CNAs, mostly copy number losses, were predicted from collagen expression by SVM, suggesting connections between the genetics and the tumor ecosystem. No one PCPG ColCluster was enriched for the large majority of hallmark gene sets (**Figure 6**). A few hallmark gene sets showed high enrichment in a PCPG ColCluster. Glycolysis, Hypoxia, and MTORC Signaling gene sets were enriched in PCPG-C1 (**Figure 6**). EMT and Cholesterol Metabolism gene sets were enriched in PCPG-C2. Pancreatic Beta Cells gene set was enriched in PCPG-C3. G2M checkpoint and KRAS Signaling Down gene sets were high in PCPG-C4. A number of gene sets were the lowest in PCPG-C4 compared to the other PCPG ColClusters including Adipogenesis, Angiogenesis, Hypoxia, Kras Signaling Up, and Protein Secretion. PCPG ColClusters did not have significant biases of aneuploidy scores or sufficient numbers to test by SVM (**Figure 5**). SVM predicted a number of specific chromosome arm level gains and losses by collagen composition (**Figure 5**A).

Prostate adenocarcinoma (PRAD)

Three PRAD ColClusters were identified. PRAD patients in the TCGA cohort live a long time and no association with overall survival was observed (**Figure 2** and **Supplemental Figure 4**). PRAD-C2 is the high fibrillar expression ColCluster even thought PRAD-C1 has higher stroma fraction (**Figure 2**B). PRAD-C1 is marked by expression of COL4A5/6, COL7A1, and COL9A1. PRAD-C3 is marked by expression of COL2A1 and COL9A2/3. No variants in the genes evaluated were significantly biased in a PRAD ColCluster (**Figure 3**). Some low frequency CNAs in a few genes were significantly enriched in specific PRAD ColClusters including MYC and RAD21 gains in PRAD-C2, PTEN losses in PRAD-C3 and AGO2 gains in PRAD-C2 and C3 (**Figure 4**). Each PRAD ColCluster had distinct enrichment for hallmarks. PRAD-C1 was enriched for Androgen Response and Interferon Alpha and Gamma gene sets (**Figure 6**). PRAD-C2 was enriched for Angiogenesis, E2F Targets, and Fatty Acid Metabolism gene sets. PRAD-C3 was enriched for DNA Repair, G2M Checkpoints, PI3K AKT MTOR Signaling, Protein Secretion, and Unfolded Protein Response. The three PRAD ColClusters have similar immunotypes with PRAD-C2 and C3 including some tumors with the "Wound Healing" immunotype not observed in PRAD-C1 (**Figure 6**). Both PRAD-C1 and C2 were enriched for neutrophils, while PRAD-C3 had relatively low levels of neutrophils (**Supplemental**

Figure 13). PRAD-C1 was enriched for B cells while no significant biases were observed for Cytoxic T cells. PRAD-C2 and C3 had higher aneuploidy scores than PRAD-C1 and the SVM predicted aneuploidy based on collagen expression with high accuracy (AUC=0.86) (**Figure 5**). Many chromosome arm level CNAs were enriched in specific PRAD ColClusters (**Figure 4**) including 8p loss in PRAD-C3, 8q gain in PRAD-C2, and 16q loss in PRAD-C2 and C3. Stratification by aneuploidy scores did not reveal significant association with overall survival (**Figure 7**). Many chromosome arm specific copy number differences were predicted well by SVM based on the collagen expression (**Figure 5**). These findings strongly suggest connections between molecular alterations and phenotypes with collagen composition in PRAD.

Rectum adenocarcinoma (READ)

Three READ ColClusters were identified and were not associated with overall survival (**Figure 2** and **Supplemental Figure 4**). READ-C1 is the high fibrillar collagen expression group (**Supplemental Figure 14**). READ-C2 has lower collagen expression and is marked by COL9A1 expression. READ-C3 is marked by COL4A5/6 and COL9A2 expression and has lower stroma fraction compared to READ-C1 and C2. APC truncations populated all the READ ColClusters, while READ-C3 was most enriched for KRAS missense variants (**Figure 3**). Relatively few READ-C1 tumors had KRAS mutations. No gene level CNAs were enriched in a READ ColCluster. READ-C2 was most enriched for a few chromosome arm level CNAs with enrichment for losses in 14q and gains in 13q, 16q, 16p, 20p and 20q (**Figure 4**). READ-C1 and C2 had similar enrichments that differed from READ-C3 including losses in 1p, 4q and 8p and gains in 7p and 8q. READ-C1 was enriched for the most hallmark gene sets including Angiogenesis, EMT, and the inflammatory hallmark gene sets (**Figure 6**). No hallmark gene sets were enriched in READ-C2. READ-C3 was enriched for Oxidative Phosphorylation and Protein Secretion gene sets. READ ColClusters were all enriched for the "Wound Healing" immunotype while READ-C1 also included tumors with the "IFN-γ" immunotype (**Figure 6**). READ-C1 was enriched for a number of immune cells consistent with an immunosuppressive environment including Macrophages and Regulatory T cells (**Supplemental Figure 13**). READ-C2 and C3 were not enriched for immune cell signatures. Aneuploidy scores were not biased across the READ ColClusters but the SVM predicted high aneuploidy at AUC=0.74 in READ (**Figure 5**). Stratification by aneuploidy did not reveal associations with overall survival in READ ColClusters (**Figure 7**). Note that these observations were based on a relatively small number of tumors which may reduce the power of enrichment tests and the definition of the READ ColClusters.

Sarcoma (SARC)

TCGA SARC represents a diverse group of tumors from multiple tissue site locations with distinct histologies that were strongly enriched into specific SARC ColClusters (**Supplemental Figure 16**). The four SARC ColClusters were borderline associated with overall survival (**Figure 2**) and (**Supplemental Figure 4**). SARC-C4 was associated with shorter overall survival. Both SARC-C1 and C2 had relatively high expression of a number of fibrillar collagens, but SARC-C2 had higher COL1A1 expression (**Supplemental Figure 3**). SARC-C2 had higher expression of COL7A1, COL8A1, COL10A1 and COL11A1. SARC-C3 was defined by relatively high expression of both COL4A1/2 and COL4A5/6. SARC-C4 was defined

by high expression of COL2A1, all three collagen type IX genes (COL9A1, COL9A2, COL9A3), COL11A2, COL20A1, COL23A1, and COL25A1. RB1 truncations and TP53 missense variants were specifically enriched in SARC-C3. No other variant enrichments for the genes evaluated were observed (**Figure 3**). Many gene level CNAs were significantly biased across the SARC ColClusters (**Figure 4**). Similar to RB1 truncations, RB1 copy number losses were also enriched in SARC-C3. MYC gains were enriched in SARC-C4. CCNE1 gains were enriched in SARC-C1. Chromosome arm level CNAs specific enrichments were prevalent across the SARC ColClusters (**Figure 4**). Most notably, 18q loss and 1p gain were enriched in SARC-C2. 10q loss was enriched in SARC-C3. SARC-C1 was defined by copy number gains in several chromosome arms including 17p, 18p, 19p and 19q. SARC ColCluster phenotypes were strongly associated with distinct phenotypes as indicated by QuSAGE enrichment of hallmark gene sets (**Figure 6**). SARC-C2 was enriched for the most hallmark gene sets with SARC-C1 also enriched for many hallmark gene sets. SARC-C4 was enriched for Notch Signaling, Unfolded Protein Response, and Wnt Beta Catenin Signaling hallmark gene sets. SARC tumors included a range of immunotypes with SARC-C4 enriched for "Wound Healing" and no "IF-γ". SARC-C1 and C2 have some tumors with "C6:TGF-β" and otherwise SARC-C1, C2, and C3 include a mixture of 4 other immunotypes (**Figure 6**). Evaluation of specific immune cell genets found that SARC-C3 was enriched for B cells (**Supplemental Figure 13**). SARC-C1 was enriched for a variety of immune cells including dendritic. Neutrophils and Tregs were enriched in both SARC-C1 and C2. SARC-C3 and C4 had relatively low expression of a number of immune cells including Neutrophils and Tregs. SARC-C4 was enriched for T helper cells. SARC-C3 had lower aneuploidy scores compared to the other 3 SARC ColClusters (**Figure 5**). The SVM moderately predicted SARC high aneuploid tumors (AUC=0.73). Many chromosome arm level CNAs, especially copy number losses, were predicted by SVM (**Figure 5**A). In sum, collagen composition helped define the tissue origin and evolution of the tumor behaviours.

Skin Cutaneous Melanoma (SKCM)

The four identified SKCM ColClusters were not associated with overall survival. We only evaluated the primary SKCM tumors because the TME and ECM would be expected to differ greatly in metastases and therefore observations are limited in SKCM because of the small cohort (**Supplemental Table 3**). SKCM ColClusters were weakly defined (**Supplemental Figure 1**). SKCM-C1 and C2 had relatively high fibrillar collagen expression (**Supplemental Figure 14**). SKCM-C3 was defined by COL2A1. SKCM-C4 was marked by generally low heterogeneous collagen expression. No somatic mutations in the genes tested, gene level nor chromosome arm levels CNAs tested were significantly biased across the SKCM ColClusters. Aneuploidy scores were not biased across the SKCM ColClusters and the SVM predicted high aneuploidy at only AUC=0.65 in SKCM (**Figure 5**). The SVM did predict specific chromosome arm level CNAs for a number of arm level gains and losses (**Figure 5**). No hallmark gene sets were significantly enriched compared to the other ColClusters for Hallmark gene sets (**Figure 6**). SKCM-C2 was notable for low enrichment of MTORC1 Signaling and Oxidative Phosphorylation hallmark gene sets. SKCM ColClusters included tumors from four immunotypes (**Figure 6**). Likely because of low numbers, QuSAGE did not identify enriched immune cell signatures in the SKCM ColClusters (**Supplemental Figure 13**). Stratification by aneuploidy scores did not reveal significant association with overall survival (**Figure 7**). Note that these observations were based on a relatively small

number of tumors which may reduce the power of enrichment tests and the definition of the SKCM ColClusters.

Stomach Adenocarcinoma (STAD)

STAD ColClusters were strongly associated with overall survival (**Supplemental Figure 4**). STAD-C3, enriched for aneuploid tumors and along with the high collagen STAD-C1, had the shortest overall survival. STAD-C1 had higher stroma fraction compared to the other 4 STAD ColClusters with similar stroma faction (**Figure 2**). STAD-C1 and STAD-C2 both had high expression of fibrillar collagens with some differences. STAD-C1 had high expression of COL3A1 and collagen type IV and STAD-C2 had higher expression of COL5A1/2 and COL11A1. STAD-C3, the high aneuploidy ColCluster, was marked by high COL2A1 expression. High COL9A3 and COL11A2 expression mark STAD-C5. STAD-C4, with high expression of COL11A2 and COL9A3, was enriched for APC truncations (**Figure 3**) and had the highest levels of the Wnt signaling gene set (**Figure 6**). ARID1A mutations were enriched in STAD-C1,C2, and C4, but not in the high aneuploidy STAD-C3 and C5 groups. MSI cases were largely grouped into STAD-C2, but some MSI tumors were spread to other ColClusters (**Supplemental Figure 14**). P53 missense variants were enriched in STAD-C3. STAD-C3 and STAD-C5 were enriched for many gene and arm level CNAs (**Figure 4**). No other significantly biased CNAs were enriched in the other ColClusters. Tumors with three different immunotypes were significantly populated in STAD-C1 (**Figure 6**). STAD-C2, C4, and C5 has similar distributions between "Wound Healing" and "IFN-γ", while the majority of STAD-C3 tumors were in "Wound Healing" (**Figure 6**). STAD-C1 had high expression levels of many of the immune cell signatures. STAD-C2 was highest for activated Dendritic Cells (aDC). STAD-C3 had the lowest levels of B cells and cytotoxic cells. STAD-C4 was highest for NK cells. STAD-C5 was enriched for Wnt Beta Catenin Signaling. Angiogenesis was highest in STAD-C1 and C2, which were the two high fibrillar and collagen type IV STAD ColClusters. STAD-C3 and STAD-C5 were enriched for aneuploid tumors and the SVM predicted aneuploidy in STAD tumors with high accuracy (**Figure 5**). Chromosome arm copy number losses were enriched by collagen expression and identified by SVM, while fewer chromosome arm copy number gains were identified by SVM (**Figure 5**A). Across all tumors, high and low aneuploidy were not associated with overall survival (**Figure 7**), but when evaluating tumors with high and low aneuploidy divided by ColClusters, significant differences in overall survival were observed. ColClusters distinguished the range of overall survival times observed across STAD tumors suggesting that collagen composition is a critical determinant. The big difference in STAD-C3 and C5 in overall survival even though they both have many high aneuploid tumors highlights this observation. In sum, STAD tumors have a range of collagen composition that distinguishes tumors by their molecular alterations and tumor states with a range of associations with overall survival. These findings suggest that the treatments used on these tumors were dependent on collagen composition and it is likely that future treatment options for STAD would also be dependent on collagen expression profiles.

Testicular Germ Cell Tumors (TGCT)

Four TGCT ColClusters were identified that were not associated with overall survival as the large majority of patients all had long overall survival (**Figure 2**) and (**Supplemental Figure 4**). TGCT-C4 was high in fibrillar collagen and yet has the

lowest stroma fraction compared to the other three TGCT ColClusters. TCGT-C3 had the second expression levels of fibrillar collagens. TGCT-C1 and C2 were marked by expression of COL6A6, COL17A1, COL22A1, COL23A1, and the neuronal specific collagen, COL20A1. The collagen type IV genes were key discriminators as COL4A5/6 was high in TGCT-C4 and TGCT-C1 but not TGCT-C2 and TGCT-C3. TGCT-C4 was enriched for AGO2, MYC, and RAD21 copy number gains (**Figure 4**). KRAS amplifications were high in each TGCT ColCluster except TGCT-C1. TCGT-C1 was enriched for KIT and KRAS missense mutations. A number of chromosome arm levels CNAs were enriched in specific TGCT ColClusters. 1q, 12q, and 22q gains were enriched in TGCT-C2, while 22q losses were enriched in TGCT-C4. TGCT-C3 and TGCT-C4 were enriched for 19 and 25 hallmark gene sets, respectively (**Figure 6**). TGCT-C1 was enriched for Allograft rejection, Interferon Alpha, Interferon Gamma and KRAS Signaling Up gene sets (**Figure 6**). TGCT-C1 and TGCT-C2 were negatively enriched for most of the gene sets. TGCT-C4 was enriched for the "Wound Healing" immunotype, while the other three TGCT ColClusters were enriched for "IFN-γ" immunotype. No biases in aneuploid tumors were observed across the TGCT ColClusters. TGCT-C1 and C2 were enriched for a number of immune cells while TGCT-C4 was not, except for Mast, regulatory T, and iDC cells. In total, these observations suggest that specific molecular alterations and phenotypes of TGCT tumors were associated with distinctive collagen defined tumors.

Thyroid carcinoma (THCA)

Four THCA ColClusters were identified that were modestly associated with overall survival with THCA-C1 and C3 being the lower overall survival groups (**Figure 2**). THCA ColClusters were clearly defined by stark differences in collagen expression. THCA-C4 has lower stroma fraction than the other three THCA ColClusters (**Figure 2**B). With similar stroma fraction as other ColClusters, THCA-C1 is still defined by higher fibrillar collagen expression compared to the other THCA ColClusters, including collagen types I and V along with COL10A1, COL11A1, COL12A1, COL22A1, and COL24A1 (**Supplemental Figure 14**). THCA-C2 had relatively low collagen expression, while THCA-C3 was marked by COL4A1/2, COL4A5/6, and COL9A3. THCA-C4 was defined by COL4A5/6, COL6A6, and COL9A1. BRAF missense mutations were enriched in THCA-C1, C2 and C3 (**Figure 3**). Only one THCA tumor in THCA-C3 had a BRAF mutation (**Supplemental Figure 14**). NRAS missense variants were enriched in THCA-C4 with a few tumors with NRAS missense in THCA-C3. Only one THCA tumor in THCA-C1 or C2 had a NRAS missense mutation. Low frequency EGFR amplifications were enriched in THCA-C3. Chromosome arm level CNAs were not frequent in THCA. 22q loss was enriched in THCA-C4. 12p, 12q, 5p, 5q, 7p, and 7q gains were enriched in THCA-C3. 1q gain was enriched in THCA-C1. THCA-C1 was enriched for the most hallmark gene sets (**Figure 6**). Notably, THCA-C1 and C2 had similar hallmark enrichment patterns, while THCA-C3 and C4 were enriched for similar hallmarks. THCA-C1 was strongly enriched for Angiogenesis. Both THCA-C1 and C2 were enriched for multiple inflammation related hallmark gene sets along with EMT and Cholesterol Metabolism. Fatty Acid Metabolism, Oxidative Phosphorylation, and mTORC signaling were enriched in THCA-C3 and C4. All THCA tumors were in the "C3:Inflammatory" immunotype (**Figure 6**). TH17 cells were enriched in THCA-C2, C3, and C4, not C1 (**Supplemental Figure 13**). Many other immune cell gene sets were much lower in THCA-C2, C3, and C4 while being enriched in THCA-C1 (**Supplemental**

Figure 13). Aneuploidy scores were not biased across the THCA ColClusters. The SVM however did predict aneuploidy with AUC=0.83 (**Figure 5**). SVM predicted the CNA for just 2 chromosome arm level gains and 3 losses. THCA-C2 showed strong separation between high and aneuploidy relative to overall survival (**Figure 7**). All together, these observations highlight the value of connecting molecular alterations and phenotypes in THCA tumors.

Thymoma (THYM)

Three THYM ColClusters were identified that were not associated with overall survival (**Figure 2**) and (**Supplemental Figure 4**). THYM-C2 was the high fibrillar collagen expression ColCluster with similar stroma fraction to THYM-C1 (**Figure 2**B). Even with lower stroma fraction, THYM-C3 also included high expression of some fibrillar collagens along with COL8A2 and COL28A1. THYM-C1 had relatively lower expression of collagens. THYM-C2 had higher overall mutation rates, but no gene with mutations was enriched in a ColCluster (**Figure 3**). Only very low frequency gene level CNAs were observed and localized to THYM-C2 (**Figure 4**). Interestingly, a number of chromosome arm level CNAs were localized to THYM-C2 (**Figure 4**). The modest genetic enrichments were complemented by strong phenotypic enrichments in the THYM ColClusters. THYM-C2 was enriched for inflammatory gene sets including Inflammatory Response and IL6 JAK STAT3 Signaling hallmark gene sets. Wnt Beta Catenin Signaling and TGFbeta were enriched in THYM-C3. No immunotypes were reported for THYM. Activated CD8 T cell expression signature was enriched in THYM-C1 (**Supplemental Figure 13**). B cell and neutrophil expression signatures were enriched in THYM-C2 while THYM-C3 was enriched for more immunosuppressive cells including macrophages and T regulatory cells. Aneuploidy scores were lower in THYM-C3 and the SVM predicted aneuploidy scores with high accuracy, but there were fewer than 10 high aneuploid cases in the THYM cohort (**Figure 5**). A number of chromosome arm level copy number gains and losses were predicted by collagen expression by SVM including 1q gain and 11p loss (**Figure 5**). Differences in aneuploidy were associated with high and low overall survival in THYM-C2 (**Figure 7**), though it may be a flaw in the data analysis as there were very few high aneuploidy tumors (**Supplemental Table 3**).

Uterine Corpus Endometrial Carcinoma (UCEC)

The four UCEC ColClusters were associated with overall survival (**Figure 2**). UCEC-C1 was the high fibrillar collagen expression ColCluster even with similar stroma fraction with all UCEC ColClusters except UCEC-C4. UCEC-C2 is the low collagen expression ColCluster. UCEC-C3 was defined by COL2A1 and COL21A1 expression. UCEC-C4 was defined by COL8A2, collagen type IX, COL19A1, COL22A1, COL23A1, and COL25A1 expression (**Supplemental Figure 14**). UCEC-C1,C2, and C3 have relatively high mutation rates compared to UCEC-C4 (**Figure 3**). PTEN truncations were enriched in UCEC-C1, C2, and C3, while P53 missense mutations were enriched in UCEC-C4. ARID1A truncations were enriched in UCEC-C1 and C3 and absent in UCEC-C4. Many gene and chromosome arm CNAs were enriched in UCEC-C4 (**Figure 4**), as this ColCluster has very high aneuploidy and polyploidy compared to the other UCEC ColClusters. SVM predicted high aneuploid tumors with AUC=0.74. The SVM predicted many chromosome level CNAs with high accuracy (**Figure 5**). UCEC-C4 had a distinct distribution of immunotypes with more "IFN-γ" while the other UCEC ColClusters had more "Wound

Healing" immunotypes (**Figure 6**). UCEC-C3 was enriched for Bile Acid Metabolism and Protein Secretion gene sets (**Figure 6**). The high aneuploid, shorter survival, UCEC-C4 was enriched for DNA Repair, E2F targets, G2M Checkpoints, Hedgehog Signaling, and Notch Signaling gene sets. Some high aneuploid tumors were in other UCEC-C1 and C3. Strong differences in high and low aneuploidy were linked to overall survival in UCEC-C1 and C3 (**Figure 7**). In UCEC-C4, the few low aneuploid tumors were too few for significant low p-value, but none showed any death. In sum, molecular alterations, overall survival and collagen composition were all associated together in UCEC defining the tumors responding well to current treatments, even high and low aneuploid tumors.

References

1. Jones, V. A., Patel, P. M., Gibson, F. T., Cordova, A. & Amber, K. T. The Role of Collagen XVII in Cancer: Squamous Cell Carcinoma and Beyond. *Front. oncology* **10**, 352, DOI: 10.3389/fonc.2020.00352 (2020).

2. Louis, D. N. *et al.* The 2021 WHO Classification of Tumors of the Central Nervous System: a summary. *Neuro-Oncology* **23**, 1231–1251, DOI: 10.1093/neuonc/noab106 (2021).

3. Raphael, B. J. *et al.* Integrated Genomic Characterization of Pancreatic Ductal Adenocarcinoma. *Cancer cell* **32**, 185–203.e13, DOI: 10.1016/j.ccell.2017.07.007 (2017).

4. Ricard-Blum, S. The Collagen Family. *Cold Spring Harb. Perspectives Biol.* **3**, a004978, DOI: 10.1101/cshperspect.a004978 (2011).

List of Supplemental Tables

Supplemental Table 1. Summary of the major collagen families.

Supplemental Table 2. Number of tumors in each cancer type with available data for each data type.

Supplemental Table 3. List of TCGA IDs and cluster assignments.

Supplemental Table 4. Univariate cox proportional hazards analysis of the ColClusters, stage, and stroma fraction.

Supplemental Table 5. multivariate cox proportional hazards analysis of the ColClusters, stage, and stroma fraction.

Supplemental Table 6. Counts for each immunotypes in each colcluster and P-values of chi-squared test evaluating biased distribution of immunotypes defined by Thorsson *et al.*[?] across the ColClusters in each cancer type.

Supplemental Table 1. Summary of the major collagen families. Descriptions of each collagen family have been reviewed[12].

Structural Family	Gene	Putative Role	References
Fibril forming	COL1A1	Fiber collagen	(2)
	COL1A2	Fiber collagen	(2)
	COL2A1	Fiber collagen	(3)
	COL3A1	Fiber collagen	(4)
	COL5A1, COL5A2	Promotes Type I fibers	(5)
	COL5A3	Negative regulator of Type I fibers	
	COL11A1	Promotes Type I fibers	(6, 7)
	COL11A2	Promotes Type I fibers	(8)
	COL14A1	Fibril surface; Negative regulator of Type I fibers	(9)
	COL24A1	Type I fibrillogenesis regulator	
	COL27A1		(10)
FACIT	COL9A1		(8)
	COL9A2		(8)
	COL9A3		(8)
	COL12A1		(11, 12)
	COL15A1	Banded Fibril linker	(13)
	COL19A1	Basement membrane zones	
	COL20A1		
	COL21A1		
	COL22A1	Basement membrane zones	
Network			
	COL4A1	Basement	(14)
	COL4A2	Basement	(14)
	COL8A1	Basement	
	COL10A1	Chondrocyte matrix deposition	(15)
COL6	COL6A1	Basement membrane/interstitial matrix	(16)
	COL6A2, COL6A3	Basement membrane/interstitial matrix	(16)
	COL7A1	Dermoepidermal Anchoring fibril	(17)
	COL26A1		
	COL28A1		
Membrane	COL13A1	Not known function	(18)
	COL17A1	Dermoepidermal anchoring complex	(19)
	COL23A1	Not known function	
	COL25A1	Linked with amyloid formation	(20)
Multiplexins	COL18A1		(13)

Supplemental Table 2. **List of available data for each cancer type. See Excel File**

Supplemental Table 3. **Assignments of ColClusters, PanColClusters, and aneuploidy for each sample. See Excel File**

Supplemental Table 4. Univariate Cox proportional hazards analysis of ColClusters, stromal fraction, and staging in each cancer type.

Characteristic	HR	0.05 CI	0.95 CI	p
BLCA-C1	1.00			
BLCA-C2	0.96	0.66	1.38	8.1E-01
BLCA-C3	0.78	0.50	1.21	2.7E-01
BLCA-C4	1.62	0.90	2.88	1.1E-01
BLCA-C5	0.55	0.33	0.94	3.0E-02
BLCA.Stromal.Fraction	1.93	0.98	3.79	5.7E-02
BLCA.pStageII	2104467.97	0.00	inf	9.9E-01
BLCA.pStageIII	3255994.00	0.00	inf	9.9E-01
BLCA.pStageIV	5861811.23	0.00	inf	9.9E-01
BRCA-C1	1.00			
BRCA-C2	0.64	0.25	1.63	3.5E-01
BRCA-C3	1.11	0.74	1.68	6.1E-01
BRCA-C4	1.04	0.59	1.84	8.9E-01
BRCA-C5	1.07	0.65	1.75	7.9E-01
BRCA.Stromal.Fraction	0.83	0.34	2.05	6.9E-01
BRCA.pStageII	1.60	0.91	2.80	1.0E-01
BRCA.pStageIII	3.11	1.73	5.60	1.5E-04
BRCA.pStageIV	9.39	4.54	19.40	1.5E-09
CESC-C1	1.00			
CESC-C2	0.99	0.56	1.72	9.6E-01
CESC-C3	0.47	0.25	0.87	1.6E-02
CESC.Stromal.Fraction	0.34	0.07	1.50	1.5E-01
CESC.cStageII	0.91	0.46	1.81	8.0E-01
CESC.cStageIII	1.32	0.63	2.76	4.6E-01
CESC.cStageIV	4.83	2.58	9.05	8.5E-07
COAD-C1	1.00			
COAD-C2	0.85	0.48	1.52	5.8E-01
COAD-C3	0.79	0.34	1.86	5.9E-01
COAD-C4	0.35	0.17	0.75	6.9E-03
COAD.Stromal.Fraction	3.09	0.78	12.17	1.1E-01

Supplemental Table 4. **Univariate Cox proportional hazards analysis of ColClusters, stromal fraction, and staging in each cancer type.**

Characteristic	HR	0.05 CI	0.95 CI	p
COAD.pStageII	1.78	0.52	6.12	3.6E-01
COAD.pStageIII	3.51	1.04	11.81	4.3E-02
COAD.pStageIV	9.47	2.78	32.20	3.2E-04
COADREAD-C1	1.00			
COADREAD-C2	0.60	0.35	1.05	7.4E-02
COADREAD-C3	0.89	0.47	1.66	7.1E-01
COADREAD-C4	0.31	0.16	0.62	9.2E-04
COADREAD.Stromal.Fraction	2.33	0.64	8.50	2.0E-01
COADREAD.pStageII	1.04	0.38	2.84	9.3E-01
COADREAD.pStageIII	2.54	0.98	6.59	5.5E-02
COADREAD.pStageIV	5.52	2.07	14.73	6.5E-04
ESCA-C1	1.00			
ESCA-C2	0.85	0.38	1.90	6.9E-01
ESCA-C3	0.73	0.39	1.38	3.4E-01
ESCA-C4	0.92	0.48	1.78	8.1E-01
ESCA.Stromal.Fraction	0.75	0.17	3.26	7.1E-01
ESCA.pStageII	1.81	0.68	4.79	2.3E-01
ESCA.pStageIII	4.26	1.57	11.55	4.4E-03
ESCA.pStageIV	9.58	2.89	31.71	2.2E-04
GBM-C1	1.00			
GBM-C2	0.97	0.51	1.85	9.4E-01
GBM-C3	0.83	0.51	1.37	4.7E-01
GBM-C4	0.77	0.47	1.26	3.0E-01
GBM.Stromal.Fraction	1.75	0.53	5.78	3.6E-01
HNSC-C1	1.00			
HNSC-C2	1.07	0.81	1.42	6.2E-01
HNSC-C3	0.39	0.20	0.74	4.2E-03
HNSC.Stromal.Fraction	0.89	0.39	2.03	7.8E-01
HNSC.cStageII	1.01	0.45	2.27	9.7E-01
HNSC.cStageIII	1.19	0.53	2.64	6.7E-01

Supplemental Table 4. Univariate Cox proportional hazards analysis of ColClusters, stromal fraction, and staging in each cancer type.

Characteristic	HR	0.05 CI	0.95 CI	p
HNSC.cStageIV	1.11	0.52	2.39	7.8E-01
KIRC-C1	1.00			
KIRC-C2	0.42	0.31	0.58	9.5E-08
KIRC-C3	0.64	0.36	1.15	1.4E-01
KIRC.Stromal.Fraction	3.00	0.67	13.46	1.5E-01
KIRC.pStageII	1.17	0.61	2.24	6.3E-01
KIRC.pStageIII	2.83	1.86	4.28	9.9E-07
KIRC.pStageIV	6.46	4.35	9.58	2.1E-20
KIRP-C1	1.00			
KIRP-C2	0.31	0.12	0.77	1.2E-02
KIRP-C3	2.58	1.01	6.57	4.7E-02
KIRP-C4	0.22	0.07	0.68	8.4E-03
KIRP-C5	0.34	0.12	0.95	3.9E-02
KIRP-C6	0.32	0.11	0.98	4.7E-02
KIRP.Stromal.Fraction	2.16	0.34	13.90	4.2E-01
KIRP.pStageII	1.43	0.40	5.16	5.8E-01
KIRP.pStageIII	4.61	2.11	10.05	1.2E-04
KIRP.pStageIV	15.45	6.21	38.45	4.0E-09
LGG-C1	1.00			
LGG-C2	0.99	0.59	1.67	9.8E-01
LGG-C3	0.50	0.28	0.89	1.9E-02
LGG-C4	0.55	0.31	0.99	4.6E-02
LGG-C5	0.42	0.21	0.83	1.3E-02
LGG.Stromal.Fraction	1.10	0.44	2.77	8.4E-01
LIHC-C1	1.00			
LIHC-C2	1.53	0.95	2.46	8.0E-02
LIHC-C3	1.13	0.77	1.66	5.4E-01
LIHC.Stromal.Fraction	0.89	0.36	2.23	8.1E-01
LIHC.pStageII	1.44	0.89	2.34	1.4E-01
LIHC.pStageIII	2.53	1.65	3.88	2.1E-05

Supplemental Table 4. **Univariate Cox proportional hazards analysis of ColClusters, stromal fraction, and staging in each cancer type.**

Characteristic	HR	0.05 CI	0.95 CI	p
LIHC.pStageIV	5.07	1.81	14.22	2.0E-03
LUAD-C1	1.00			
LUAD-C2	0.52	0.36	0.77	9.4E-04
LUAD-C3	0.58	0.38	0.87	7.8E-03
LUAD-C4	0.67	0.38	1.17	1.6E-01
LUAD.Stromal.Fraction	0.44	0.19	1.01	5.2E-02
LUAD.pStageII	2.29	1.59	3.30	9.5E-06
LUAD.pStageIII	3.51	2.40	5.13	9.2E-11
LUAD.pStageIV	3.93	2.26	6.83	1.2E-06
LUSC-C1	1.00			
LUSC-C2	0.93	0.56	1.55	7.9E-01
LUSC-C3	0.94	0.61	1.45	7.8E-01
LUSC-C4	0.94	0.60	1.46	7.8E-01
LUSC-C5	0.54	0.33	0.86	1.1E-02
LUSC-C6	0.78	0.52	1.18	2.4E-01
LUSC.Stromal.Fraction	1.36	0.62	3.00	4.4E-01
LUSC.pStageII	1.34	0.97	1.87	7.6E-02
LUSC.pStageIII	1.89	1.31	2.73	7.3E-04
LUSC.pStageIV	3.33	1.34	8.23	9.4E-03
OV-C1	1.00			
OV-C2	1.10	0.76	1.60	6.1E-01
OV-C3	0.88	0.58	1.34	5.6E-01
OV.Stromal.Fraction	0.71	0.15	3.44	6.7E-01
OV.cStageII	0.66	0.26	1.63	3.7E-01
OV.cStageIII	0.86	0.54	1.37	5.2E-01
PAAD-C1	1.00			
PAAD-C2	1.79	1.07	3.01	2.8E-02
PAAD-C3	1.76	0.92	3.36	8.9E-02
PAAD-C4	0.29	0.08	0.97	4.5E-02
PAAD.Stromal.Fraction	4.00	1.58	10.08	3.3E-03

Supplemental Table 4. **Univariate Cox proportional hazards analysis of ColClusters, stromal fraction, and staging in each cancer type.**

Characteristic	HR	0.05 CI	0.95 CI	p
PAAD.pStageII	2.39	1.10	5.22	2.8E-02
PAAD.pStageIII	1.26	0.15	10.36	8.3E-01
PAAD.pStageIV	1.89	0.39	9.16	4.3E-01
PCPG-C1	1.00			
PCPG-C2	0.99	0.09	10.98	1.0E+00
PCPG-C3	1.35	0.12	14.97	8.1E-01
PCPG-C4	0.00	0.00	inf	1.0E+00
PCPG.Stromal.Fraction	0.00	0.00	1.06	5.1E-02
PRAD-C1	1.00			
PRAD-C2	93553705.17	0.00	inf	1.0E+00
PRAD-C3	97429197.02	0.00	inf	1.0E+00
PRAD.Stromal.Fraction	0.11	0.00	5.42	2.7E-01
READ-C1	1.00			
READ-C2	0.38	0.11	1.34	1.3E-01
READ-C3	0.58	0.17	2.01	3.9E-01
READ.Stromal.Fraction	0.34	0.01	11.43	5.5E-01
READ.pStageII	0.08	0.01	1.02	5.2E-02
READ.pStageIII	0.91	0.18	4.62	9.1E-01
READ.pStageIV	0.88	0.12	6.28	9.0E-01
SARC-C1	1.00			
SARC-C2	1.18	0.71	1.98	5.2E-01
SARC-C3	0.80	0.46	1.39	4.3E-01
SARC-C4	1.85	0.95	3.61	7.3E-02
SARC.Stromal.Fraction	0.68	0.24	1.97	4.8E-01
SKCM-C1	1.00			
SKCM-C2	1.03	0.36	3.00	9.5E-01
SKCM-C3	0.84	0.30	2.39	7.5E-01
SKCM-C4	0.75	0.28	2.00	5.6E-01
SKCM.Stromal.Fraction	0.11	0.01	0.82	3.1E-02
SKCM.pStageII	42155345.08	0.00	inf	1.0E+00

Supplemental Table 4. Univariate Cox proportional hazards analysis of ColClusters, stromal fraction, and staging in each cancer type.

Characteristic	HR	0.05 CI	0.95 CI	p
SKCM.pStageIII	58206685.50	0.00	inf	1.0E+00
SKCM.pStageIV	375314586.48	0.00	inf	1.0E+00
STAD-C1	1.00			
STAD-C2	0.62	0.42	0.92	1.7E-02
STAD-C3	1.20	0.69	2.10	5.2E-01
STAD-C4	0.40	0.25	0.66	3.1E-04
STAD-C5	0.44	0.24	0.81	7.8E-03
STAD.Stromal.Fraction	2.11	0.93	4.80	7.4E-02
STAD.pStageII	1.36	0.72	2.58	3.5E-01
STAD.pStageIII	2.23	1.24	4.02	7.4E-03
STAD.pStageIV	3.61	1.83	7.09	2.0E-04
TGCT-C1	1.00			
TGCT-C2	0.00	0.00	inf	1.0E+00
TGCT-C3	0.00	0.00	inf	1.0E+00
TGCT-C4	1.44	0.13	15.83	7.7E-01
TGCT.Stromal.Fraction	10.09	0.05	2259.07	4.0E-01
TGCT.pStageII	0.00	0.00	inf	1.0E+00
TGCT.pStageIII	3.19	0.29	35.26	3.4E-01
THCA-C1	1.00			
THCA-C2	0.37	0.11	1.27	1.1E-01
THCA-C3	1.82	0.52	6.30	3.5E-01
THCA-C4	0.17	0.02	1.36	9.5E-02
THCA.Stromal.Fraction	3.07	0.12	79.83	5.0E-01
THCA.pStageII	5.31	0.74	37.95	9.6E-02
THCA.pStageIII	10.07	2.09	48.61	4.0E-03
THCA.pStageIV	19.23	3.69	100.21	4.5E-04
THYM-C1	1.00			
THYM-C2	1.00	0.19	5.31	1.0E+00
THYM-C3	0.24	0.03	2.03	1.9E-01
THYM.Stromal.Fraction	1.18	0.08	18.21	9.1E-01

Supplemental Table 4. Univariate Cox proportional hazards analysis of ColClusters, stromal fraction, and staging in each cancer type.

Characteristic	HR	0.05 CI	0.95 CI	p
UCEC-C1	1.00			
UCEC-C2	0.91	0.43	1.91	8.0E-01
UCEC-C3	0.60	0.26	1.37	2.3E-01
UCEC-C4	2.01	1.00	4.04	5.1E-02
UCEC.Stromal.Fraction	0.51	0.08	3.12	4.7E-01
UCEC.cStageII	1.27	0.38	4.22	7.0E-01
UCEC.cStageIII	2.50	1.30	4.79	6.0E-03
UCEC.cStageIV	7.00	3.42	14.35	1.1E-07

Supplemental Table 5. **Multivariate Cox proportional hazards analysis of ColClusters, stromal fraction, and staging in each cancer type, relative to ColCluster 1**

Characteristic	HR	0.05 CI	0.95 CI	p
BLCA-C1	1.00			
BLCA-C2	1.13	0.78	1.64	5.18E-01
BLCA-C3	1.11	0.66	1.87	6.89E-01
BLCA-C4	2.23	1.21	4.10	9.81E-03
BLCA-C5	0.95	0.50	1.80	8.78E-01
BLCA:Stromal.Fraction	1.71	0.75	3.91	2.06E-01
BLCA:pStageII	1999925.90	0.00	inf	9.94E-01
BLCA:pStageIII	3176029.62	0.00	inf	9.94E-01
BLCA:pStageIV	5630694.42	0.00	inf	9.94E-01
BRCA-C1	1.00			
BRCA-C2	0.81	0.31	2.11	6.64E-01
BRCA-C3	1.10	0.69	1.77	6.80E-01
BRCA-C4	1.36	0.73	2.54	3.32E-01
BRCA-C5	0.95	0.54	1.69	8.72E-01
BRCA:Stromal.Fraction	0.82	0.30	2.25	7.01E-01
BRCA:pStageII	1.41	0.79	2.50	2.42E-01
BRCA:pStageIII	2.98	1.64	5.41	3.36E-04
BRCA:pStageIV	8.67	4.10	18.33	1.59E-08
CESC-C1	1.00			
CESC-C2	1.28	0.71	2.28	4.09E-01
CESC-C3	0.55	0.29	1.05	6.97E-02
CESC:Stromal.Fraction	0.35	0.08	1.55	1.67E-01
CESC:cStageII	0.85	0.42	1.72	6.53E-01
CESC:cStageIII	1.16	0.55	2.47	6.99E-01
CESC:cStageIV	3.82	1.94	7.53	1.06E-04
COAD-C1	1.00			
COAD-C2	0.99	0.52	1.85	9.63E-01
COAD-C3	1.16	0.46	2.91	7.58E-01
COAD-C4	0.41	0.18	0.93	3.31E-02
COAD:Stromal.Fraction	2.22	0.44	11.24	3.37E-01

Supplemental Table 5. **Multivariate Cox proportional hazards analysis of ColClusters, stromal fraction, and staging in each cancer type, relative to ColCluster 1**

Characteristic	HR	0.05 CI	0.95 CI	p
COAD:pStageII	1.50	0.43	5.19	5.23E-01
COAD:pStageIII	3.02	0.90	10.21	7.46E-02
COAD:pStageIV	7.56	2.19	26.07	1.35E-03
COADREAD-C1	1.00			
COADREAD-C2	0.39	0.21	0.72	2.98E-03
COADREAD-C3	0.58	0.29	1.16	1.22E-01
COADREAD-C4	0.22	0.10	0.48	1.14E-04
COADREAD:Stromal.Fraction	0.79	0.19	3.31	7.48E-01
COADREAD:pStageII	0.93	0.34	2.52	8.80E-01
COADREAD:pStageIII	2.21	0.85	5.77	1.04E-01
COADREAD:pStageIV	6.04	2.18	16.73	5.36E-04
ESCA-C1	1.00			
ESCA-C2	1.27	0.49	3.28	6.18E-01
ESCA-C3	0.98	0.43	2.25	9.71E-01
ESCA-C4	1.35	0.65	2.81	4.21E-01
ESCA:Stromal.Fraction	0.29	0.05	1.59	1.56E-01
ESCA:pStageII	1.97	0.70	5.54	2.01E-01
ESCA:pStageIII	4.98	1.62	15.29	5.05E-03
ESCA:pStageIV	9.62	2.64	35.04	5.95E-04
GBM-C1	1.00			
GBM-C2	1.08	0.54	2.17	8.35E-01
GBM-C3	1.09	0.60	1.96	7.78E-01
GBM-C4	1.00	0.53	1.88	9.91E-01
GBM:Stromal.Fraction	1.82	0.40	8.30	4.41E-01
HNSC-C1	1.00			
HNSC-C2	1.09	0.81	1.47	5.69E-01
HNSC-C3	0.40	0.20	0.76	5.85E-03
HNSC:Stromal.Fraction	0.80	0.33	1.94	6.28E-01
HNSC:cStageII	0.93	0.41	2.10	8.67E-01
HNSC:cStageIII	1.13	0.50	2.54	7.66E-01

Supplemental Table 5. **Multivariate Cox proportional hazards analysis of ColClusters, stromal fraction, and staging in each cancer type, relative to ColCluster 1**

Characteristic	HR	0.05 CI	0.95 CI	p
HNSC:cStageIV	1.10	0.51	2.37	8.15E-01
KIRC-C1	1.00			
KIRC-C2	0.69	0.41	1.16	1.57E-01
KIRC-C3	1.24	0.52	2.98	6.30E-01
KIRC:Stromal.Fraction	1.33	0.23	7.84	7.50E-01
KIRC:pStageII	0.87	0.33	2.33	7.86E-01
KIRC:pStageIII	2.32	1.27	4.25	6.40E-03
KIRC:pStageIV	6.50	3.72	11.36	5.23E-11
KIRP-C1	1.00			
KIRP-C2	0.68	0.21	2.17	5.11E-01
KIRP-C3	4.11	1.47	11.54	7.21E-03
KIRP-C4	0.62	0.15	2.54	5.03E-01
KIRP-C5	1.28	0.32	5.11	7.27E-01
KIRP-C6	1.06	0.23	4.88	9.43E-01
KIRP:Stromal.Fraction	3.99	0.36	44.24	2.60E-01
KIRP:pStageII	1.62	0.43	6.14	4.80E-01
KIRP:pStageIII	4.40	1.75	11.08	1.66E-03
KIRP:pStageIV	16.32	5.14	51.82	2.17E-06
LGG-C1	1.00			
LGG-C2	0.97	0.57	1.65	8.99E-01
LGG-C3	0.48	0.26	0.87	1.50E-02
LGG-C4	0.51	0.28	0.94	3.01E-02
LGG-C5	0.38	0.19	0.78	8.83E-03
LGG:Stromal.Fraction	0.62	0.23	1.64	3.36E-01
LIHC-C1	1.00			
LIHC-C2	1.80	1.06	3.06	2.85E-02
LIHC-C3	1.41	0.86	2.30	1.77E-01
LIHC:Stromal.Fraction	1.56	0.50	4.85	4.38E-01
LIHC:pStageII	1.42	0.87	2.32	1.59E-01
LIHC:pStageIII	2.35	1.51	3.65	1.47E-04

Supplemental Table 5. **Multivariate Cox proportional hazards analysis of ColClusters, stromal fraction, and staging in each cancer type, relative to ColCluster 1**

Characteristic	HR	0.05 CI	0.95 CI	p
LIHC:pStageIV	5.55	1.96	15.69	1.24E-03
LUAD-C1	1.00			
LUAD-C2	0.53	0.35	0.81	3.21E-03
LUAD-C3	0.47	0.30	0.74	1.05E-03
LUAD-C4	0.58	0.32	1.06	7.47E-02
LUAD:Stromal.Fraction	0.18	0.07	0.46	2.89E-04
LUAD:pStageII	2.38	1.63	3.49	8.63E-06
LUAD:pStageIII	3.65	2.44	5.47	2.78E-10
LUAD:pStageIV	3.01	1.67	5.40	2.31E-04
LUSC-C1	1.00			
LUSC-C2	1.01	0.60	1.70	9.83E-01
LUSC-C3	0.92	0.59	1.44	7.16E-01
LUSC-C4	0.90	0.56	1.44	6.63E-01
LUSC-C5	0.58	0.35	0.95	2.97E-02
LUSC-C6	0.90	0.58	1.38	6.21E-01
LUSC:Stromal.Fraction	1.16	0.50	2.70	7.34E-01
LUSC:pStageII	1.44	1.03	2.01	3.40E-02
LUSC:pStageIII	1.94	1.33	2.84	5.54E-04
LUSC:pStageIV	3.02	1.20	7.59	1.87E-02
OV-C1	1.00			
OV-C2	1.09	0.64	1.86	7.57E-01
OV-C3	0.74	0.41	1.32	3.02E-01
OV:Stromal.Fraction	0.74	0.13	4.14	7.32E-01
OV:cStageII	0.40	0.12	1.33	1.36E-01
OV:cStageIII	0.43	0.23	0.78	5.19E-03
PAAD-C1	1.00			
PAAD-C2	1.55	0.86	2.80	1.44E-01
PAAD-C3	2.05	1.00	4.19	4.85E-02
PAAD-C4	0.25	0.06	1.17	7.79E-02
PAAD:Stromal.Fraction	2.52	0.90	7.07	7.92E-02

Supplemental Table 5. **Multivariate Cox proportional hazards analysis of ColClusters, stromal fraction, and staging in each cancer type, relative to ColCluster 1**

Characteristic	HR	0.05 CI	0.95 CI	p
PAAD:pStageII	1.16	0.50	2.69	7.33E-01
PAAD:pStageIII	1.07	0.12	9.10	9.54E-01
PAAD:pStageIV	0.80	0.16	4.02	7.84E-01
PCPG-C1	1.00			
PCPG-C2	0.69	0.06	8.09	7.64E-01
PCPG-C3	0.68	0.05	8.81	7.65E-01
PCPG-C4	0.00	0.00	inf	9.99E-01
PCPG:Stromal.Fraction	0.00	0.00	0.36	3.10E-02
PRAD-C1	1.00			
PRAD-C2	62828010.65	0.00	inf	9.98E-01
PRAD-C3	87156442.02	0.00	inf	9.98E-01
PRAD:Stromal.Fraction	0.18	0.00	11.54	4.23E-01
READ-C1	1.00			
READ-C2	0.18	0.04	0.85	3.02E-02
READ-C3	0.10	0.02	0.60	1.17E-02
READ:Stromal.Fraction	0.06	0.00	3.01	1.60E-01
READ:pStageII	0.02	0.00	0.49	1.64E-02
READ:pStageIII	0.35	0.05	2.26	2.70E-01
READ:pStageIV	0.61	0.08	4.69	6.38E-01
SARC-C1	1.00			
SARC-C2	1.20	0.70	2.03	5.09E-01
SARC-C3	0.69	0.37	1.29	2.49E-01
SARC-C4	1.56	0.74	3.26	2.40E-01
SARC:Stromal.Fraction	0.53	0.16	1.74	2.94E-01
SKCM-C1	1.00			
SKCM-C2	1.82	0.57	5.80	3.13E-01
SKCM-C3	0.63	0.18	2.21	4.68E-01
SKCM-C4	0.53	0.16	1.71	2.90E-01
SKCM:Stromal.Fraction	0.05	0.01	0.53	1.26E-02
SKCM:pStageII	65786343.53	0.00	inf	9.98E-01

Supplemental Table 5. **Multivariate Cox proportional hazards analysis of ColClusters, stromal fraction, and staging in each cancer type, relative to ColCluster 1**

Characteristic	HR	0.05 CI	0.95 CI	p
SKCM:pStageIII	106743440.91	0.00	inf	9.98E-01
SKCM:pStageIV	582550244.12	0.00	inf	9.98E-01
STAD-C1	1.00			
STAD-C2	0.73	0.47	1.11	1.43E-01
STAD-C3	1.43	0.80	2.54	2.28E-01
STAD-C4	0.43	0.25	0.74	2.35E-03
STAD-C5	0.56	0.29	1.06	7.59E-02
STAD:Stromal.Fraction	1.10	0.45	2.69	8.33E-01
STAD:pStageII	1.18	0.59	2.38	6.33E-01
STAD:pStageIII	1.92	1.00	3.68	4.95E-02
STAD:pStageIV	3.19	1.53	6.62	1.88E-03
TGCT-C1	1.00			
TGCT-C2	0.00	0.00	inf	9.99E-01
TGCT-C3	0.00	0.00	inf	9.99E-01
TGCT-C4	66.74	1.31	3398.14	3.62E-02
TGCT:Stromal.Fraction	6084340.94	5.54	6680069834193.79	2.77E-02
TGCT:pStageII	0.00	0.00	inf	9.99E-01
TGCT:pStageIII	56.85	0.48	6669.99	9.65E-02
THCA-C1	1.00			
THCA-C2	0.51	0.12	2.06	3.42E-01
THCA-C3	2.72	0.42	17.77	2.96E-01
THCA-C4	0.26	0.03	2.25	2.22E-01
THCA:Stromal.Fraction	2.69	0.09	82.67	5.71E-01
THCA:pStageII	4.57	0.61	34.18	1.39E-01
THCA:pStageIII	6.25	1.08	36.30	4.12E-02
THCA:pStageIV	23.02	3.84	137.94	5.97E-04
THYM-C1	1.00			
THYM-C2	0.86	0.16	4.64	8.60E-01
THYM-C3	0.22	0.02	2.03	1.82E-01
THYM:Stromal.Fraction	0.59	0.03	11.53	7.25E-01

Supplemental Table 5. Multivariate Cox proportional hazards analysis of ColClusters, stromal fraction, and staging in each cancer type, relative to ColCluster 1

Characteristic	HR	0.05 CI	0.95 CI	p
UCEC-C1	1.00			
UCEC-C2	0.84	0.36	1.93	6.75E-01
UCEC-C3	0.51	0.21	1.22	1.27E-01
UCEC-C4	1.28	0.60	2.71	5.27E-01
UCEC:Stromal.Fraction	0.23	0.04	1.45	1.17E-01
UCEC:cStageII	1.24	0.36	4.30	7.38E-01
UCEC:cStageIII	2.71	1.34	5.50	5.67E-03
UCEC:cStageIV	8.32	3.73	18.56	2.27E-07

Supplemental Table 6. **Counts for each immunotypes in each colcluster and P-values of chi-squared test evaluating biased distribution of immunotypes defined by Thorsson *et al.*[?] across the ColClusters in each cancer type.**

ColCluster	C1	C2	C3	C4	C5	C6	NA	p
BLCA-C1	70	51	4	3	0	1	2	6E-15
BLCA-C2	29	69	0	2	0	0	2	
BLCA-C3	33	21	10	8	0	0	2	
BLCA-C4	11	8	0	4	0	0	3	
BLCA-C5	28	13	6	19	0	2	2	
BRCA-C1	82	76	86	9	0	10	0	2E-15
BRCA-C2	17	36	1	5	0	0	0	
BRCA-C3	159	143	65	41	0	25	0	
BRCA-C4	38	64	9	9	0	2	0	
BRCA-C5	69	66	27	27	0	3	0	
CESC-C1	38	68	0	2	0	0	1	0.03
CESC-C2	21	66	0	0	0	0	0	
CESC-C3	17	76	0	4	0	0	2	
COAD-C1	48	29	2	1	0	3	0	5E-06
COAD-C2	77	12	0	2	0	0	0	
COAD-C3	26	1	0	0	0	0	0	
COAD-C4	59	4	2	5	0	0	0	
COADREAD-C1	39	33	3	3	0	3	0	7E-11
COADREAD-C2	104	17	1	1	0	1	0	
COADREAD-C3	56	2	1	0	0	0	0	
COADREAD-C4	84	4	2	4	0	0	0	
ESCA-C1	18	14	4	1	0	1	2	0.02
ESCA-C2	6	14	0	1	0	0	0	
ESCA-C3	13	20	3	3	0	0	6	
ESCA-C4	26	37	0	1	0	1	0	
GBM-C1	2	0	0	40	0	0	0	0.29
GBM-C2	0	0	0	18	0	0	0	
GBM-C3	0	0	0	39	1	0	0	
GBM-C4	0	0	0	41	0	0	0	
HNSC-C1	81	162	1	1	0	2	1	0.01

Supplemental Table 6. **Counts for each immunotypes in each colcluster and P-values of chi-squared test evaluating biased distribution of immunotypes defined by Thorsson *et al.*[?] across the ColClusters in each cancer type.**

ColCluster	C1	C2	C3	C4	C5	C6	NA	p
HNSC-C2	39	168	0	1	0	0	0	
HNSC-C3	8	43	1	0	0	1	0	
KIRC-C1	5	11	135	8	0	11	0	2E-16
KIRC-C2	0	6	281	9	0	2	0	
KIRC-C3	2	3	25	9	3	0	0	
KIRP-C1	0	2	36	13	0	2	0	3E-05
KIRP-C2	0	1	44	22	0	0	1	
KIRP-C3	2	1	9	2	1	0	3	
KIRP-C4	1	0	44	8	0	0	1	
KIRP-C5	0	0	33	9	0	0	0	
KIRP-C6	0	0	35	12	1	0	1	
LGG-C1	0	0	2	20	52	0	0	3E-24
LGG-C2	0	0	8	68	22	1	0	
LGG-C3	0	0	0	21	118	0	0	
LGG-C4	0	0	0	15	87	0	0	
LGG-C5	0	0	0	21	67	0	0	
LIHC-C1	12	23	80	44	0	1	5	1E-07
LIHC-C2	7	7	17	21	0	0	0	
LIHC-C3	3	14	37	94	0	0	1	
LUAD-C1	52	71	32	8	0	22	29	2E-20
LUAD-C2	6	39	93	8	0	4	5	
LUAD-C3	17	17	49	3	0	0	12	
LUAD-C4	6	17	3	1	0	2	7	
LUSC-C1	93	50	1	4	0	4	0	1E-06
LUSC-C2	17	22	1	0	0	1	0	
LUSC-C3	37	28	0	0	0	3	1	
LUSC-C4	15	34	6	1	0	4	0	
LUSC-C5	49	23	0	2	0	1	0	
LUSC-C6	56	25	0	0	0	1	0	
OV-C1	23	73	2	19	0	0	0	0.02

Supplemental Table 6. **Counts for each immunotypes in each colcluster and P-values of chi-squared test evaluating biased distribution of immunotypes defined by Thorsson *et al.*[?] across the ColClusters in each cancer type.**

ColCluster	C1	C2	C3	C4	C5	C6	NA	p
OV-C2	6	44	0	27	0	0	0	
OV-C3	13	31	1	12	0	0	0	
PAAD-C1	10	3	23	0	0	4	9	9E-14
PAAD-C2	36	23	10	0	0	13	1	
PAAD-C3	9	6	5	1	0	4	4	
PAAD-C4	2	0	2	0	0	0	12	
PCPG-C1	0	1	27	3	0	0	0	0.0006
PCPG-C2	0	0	38	17	1	2	0	
PCPG-C3	0	0	23	18	0	0	0	
PCPG-C4	0	0	18	23	4	0	0	
PRAD-C1	0	2	64	5	0	0	34	6E-05
PRAD-C2	20	8	148	25	0	0	25	
PRAD-C3	13	8	88	13	0	0	28	
READ-C1	16	7	1	0	0	1	0	0.05
READ-C2	33	2	1	0	0	0	0	
READ-C3	24	1	1	0	0	0	0	
SARC-C1	14	15	15	15	0	11	10	0.0006
SARC-C2	21	12	5	20	0	8	15	
SARC-C3	17	11	17	20	0	1	3	
SARC-C4	12	0	5	3	0	0	4	
SKCM-C1	15	5	5	3	0	1	0	0.51
SKCM-C2	11	5	2	3	0	1	0	
SKCM-C3	5	5	3	3	0	0	0	
SKCM-C4	10	12	4	10	0	0	0	
STAD-C1	20	33	27	0	0	2	2	2E-13
STAD-C2	39	102	2	5	0	3	6	
STAD-C3	21	8	1	0	0	0	3	
STAD-C4	29	42	4	2	0	2	7	
STAD-C5	18	24	2	2	0	0	3	
TGCT-C1	0	33	0	0	0	0	0	3E-10

Supplemental Table 6. Counts for each immunotypes in each colcluster and P-values of chi-squared test evaluating biased distribution of immunotypes defined by Thorsson *et al.*[?] across the ColClusters in each cancer type.

ColCluster	C1	C2	C3	C4	C5	C6	NA	p
TGCT-C2	2	34	0	0	0	0	0	
TGCT-C3	5	18	0	0	0	0	0	
TGCT-C4	35	18	2	1	0	0	0	
THCA-C1	1	5	102	3	0	3	0	0.008
THCA-C2	1	6	182	7	0	0	0	
THCA-C3	0	1	53	8	0	0	0	
THCA-C4	0	1	117	4	0	0	0	
THYM-C1	0	0	0	0	0	0	53	1
THYM-C2	0	0	0	0	0	0	17	
THYM-C3	0	0	0	0	0	0	50	
UCEC-C1	64	25	9	2	0	0	1	9E-05
UCEC-C2	47	30	14	3	0	0	0	
UCEC-C3	47	28	18	4	0	0	0	
UCEC-C4	19	37	1	4	0	0	0	

Supplemental Figures

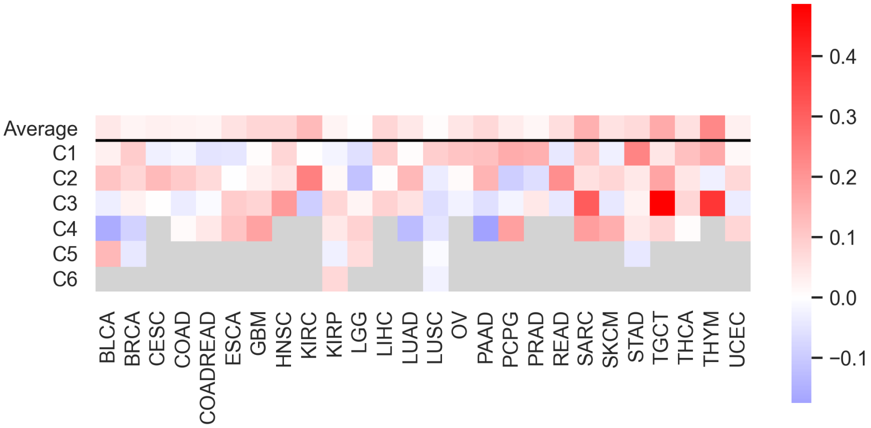

Supplemental Figure 1. Mean silhouette scores for each ColCluster.

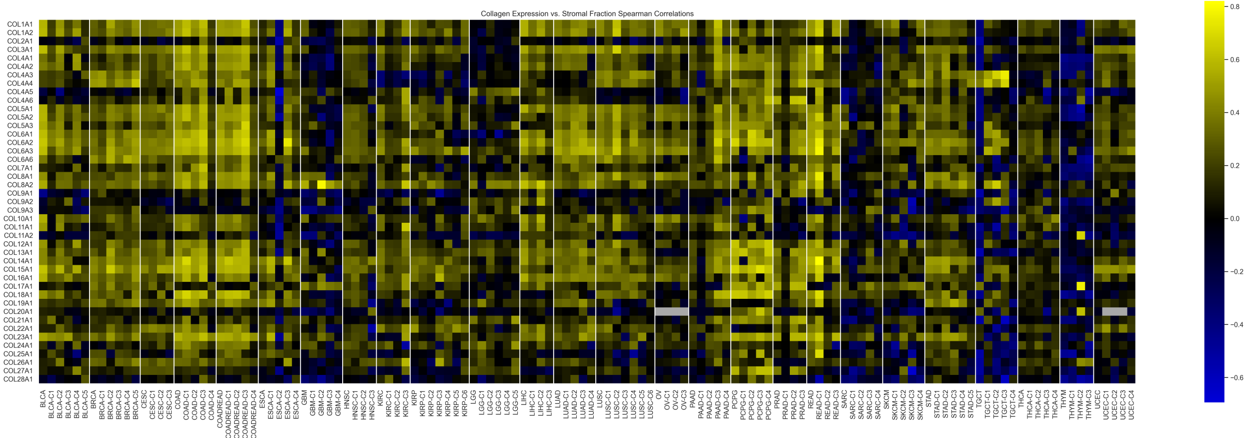

Supplemental Figure 2. Spearman correlation between stroma fraction and collagen expression in each tumor type and in each ColCluster. The fibrillar collagens and COL4A1/COL4A2 correlate in many cancer types. While non-fibrillar modulating collagens with high dynamic range of expression such as COL7A1, collagen type IX and collagens. Correlation with stroma was poor in the brain and kidney cancers which are low fibrillar collagen and high collagen type IV environments, respectively. For a number of collagens and cancer types, correlation in ColClusters differed from the overall correlation suggesting stroma and collagen expression were distinct.

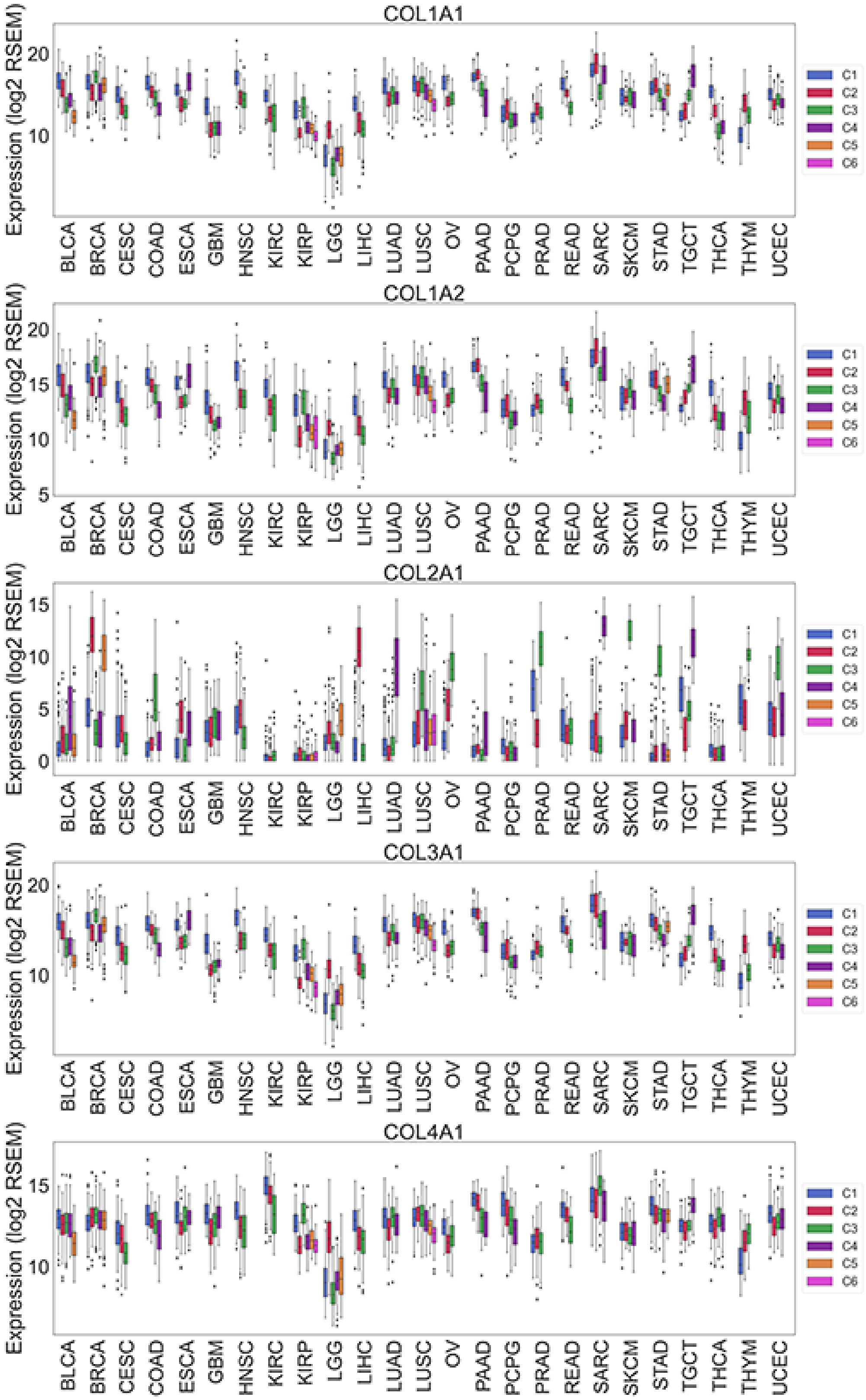

Supplemental Figure 3. Distribution of RSEM expression scores for each collagen gene in each cancer type in each ColCluster. Center line represents median and whiskers delineate interquartile range.

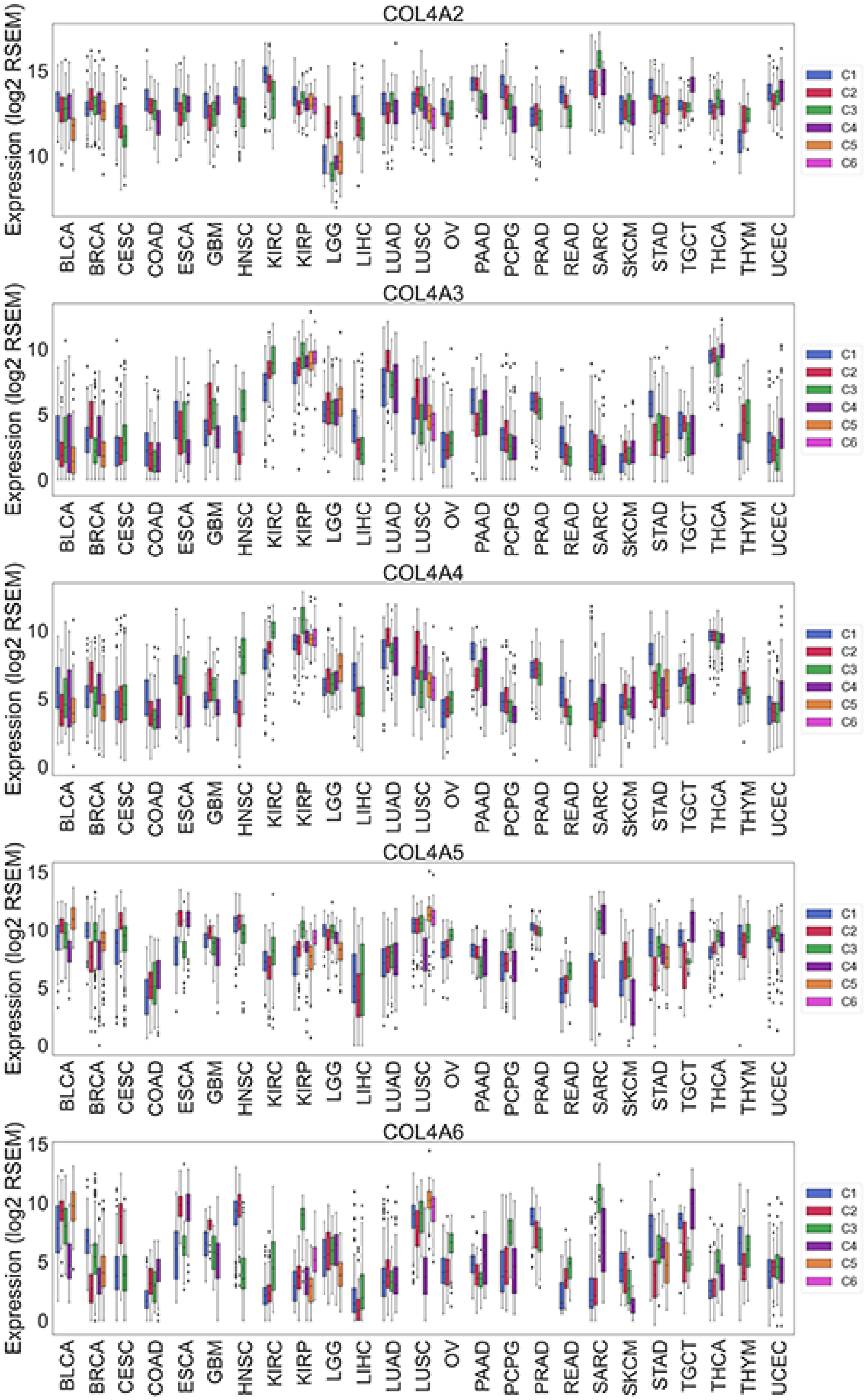

Supplemental Figure 3. Distribution of RSEM expression scores for each collagen gene in each cancer type in each ColCluster. Center line represents median and whiskers delineate interquartile range.

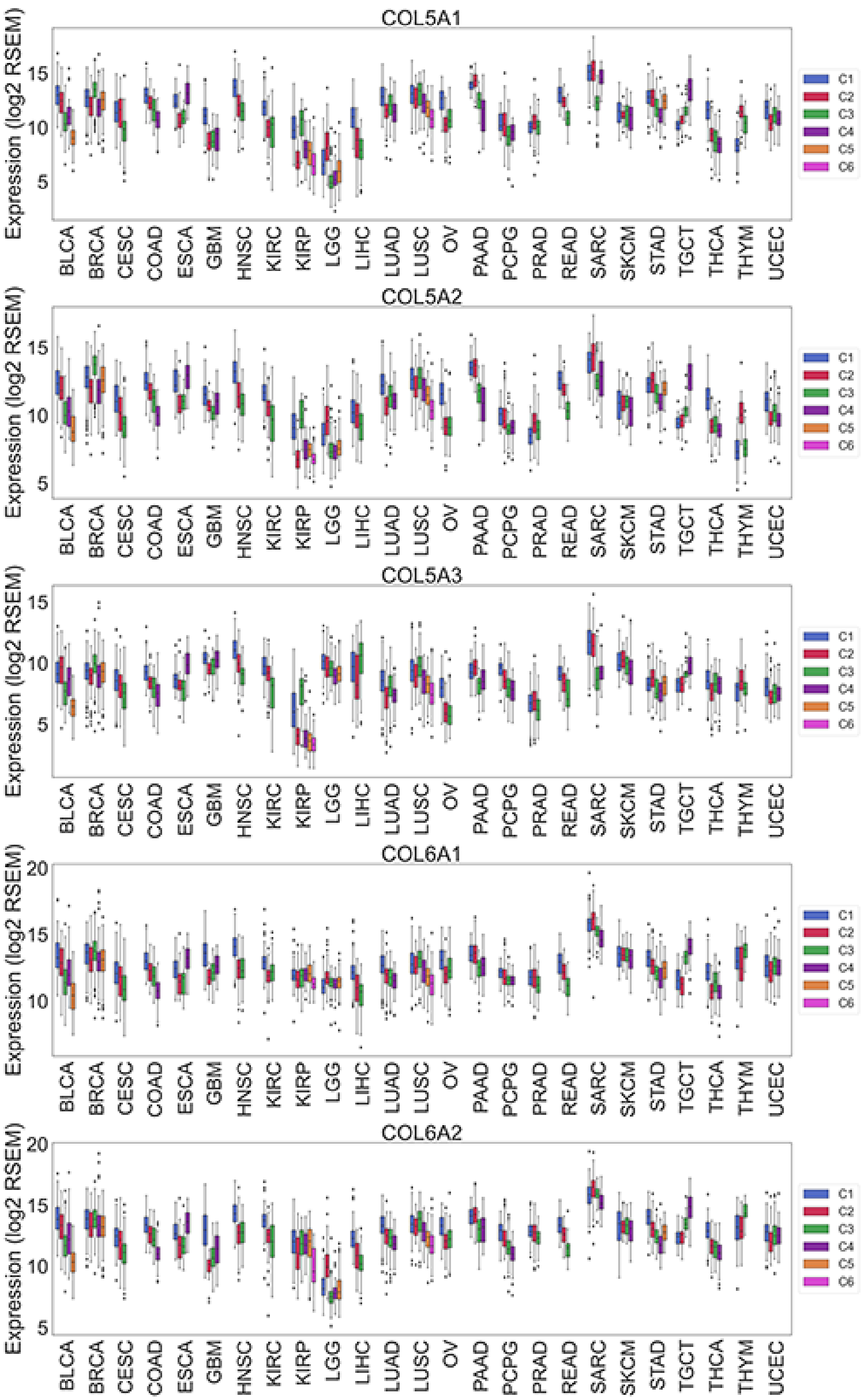

Supplemental Figure 3. Distribution of RSEM expression scores for each collagen gene in each cancer type in each ColCluster. Center line represents median and whiskers delineate interquartile range.

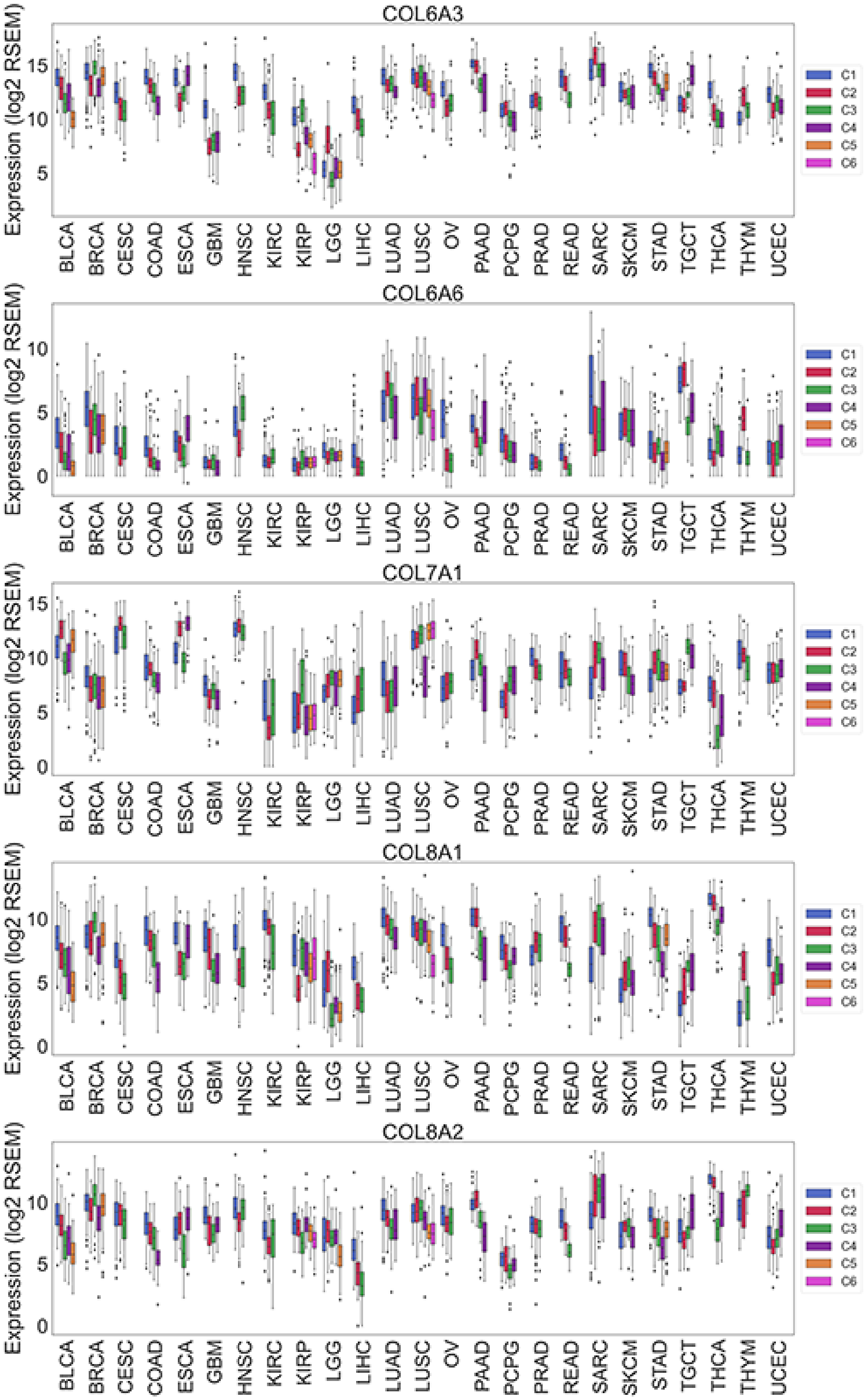

Supplemental Figure 3. Distribution of RSEM expression scores for each collagen gene in each cancer type in each ColCluster. Center line represents median and whiskers delineate interquartile range.

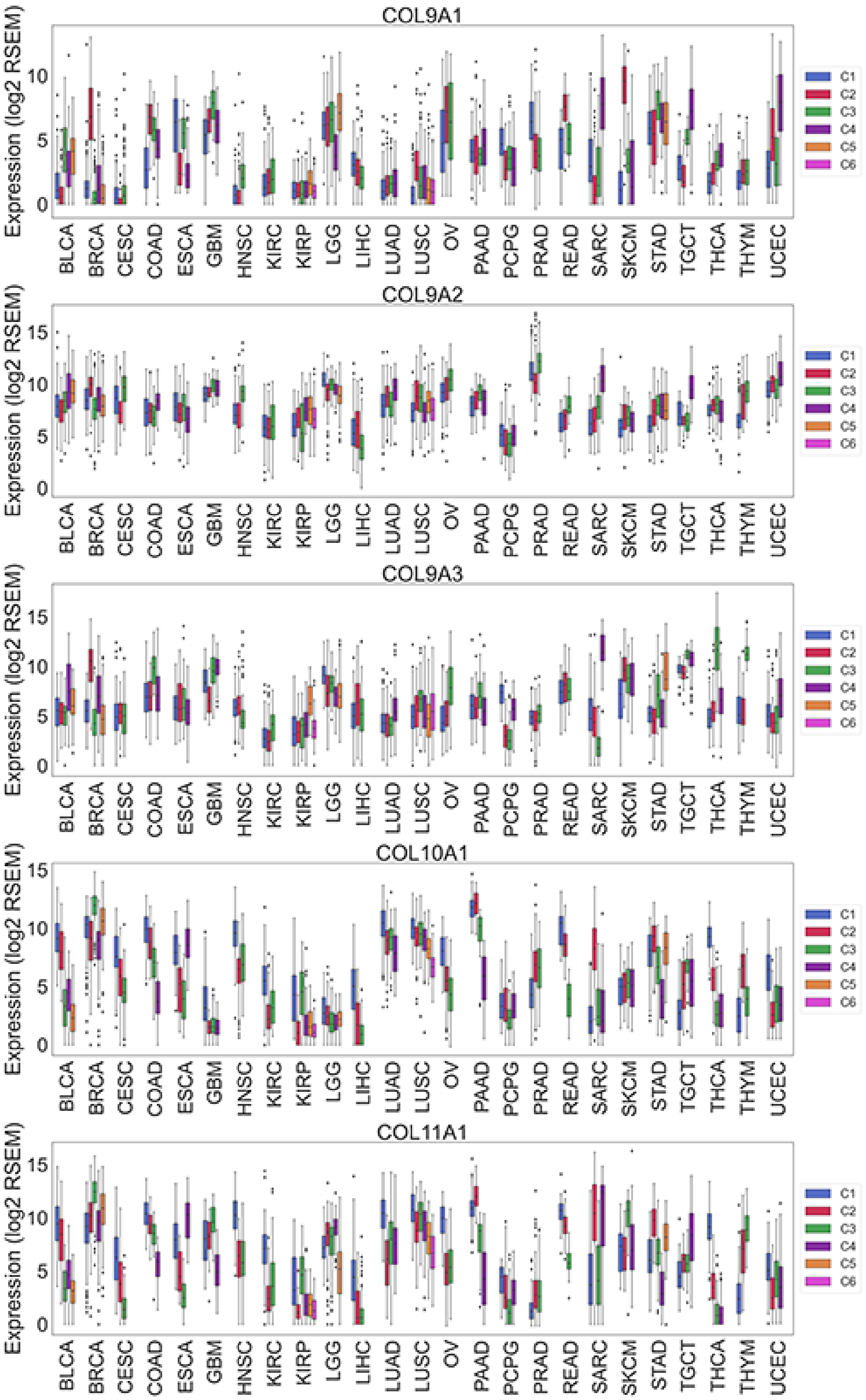

Supplemental Figure 3. Distribution of RSEM expression scores for each collagen gene in each cancer type in each ColCluster. Center line represents median and whiskers delineate interquartile range.

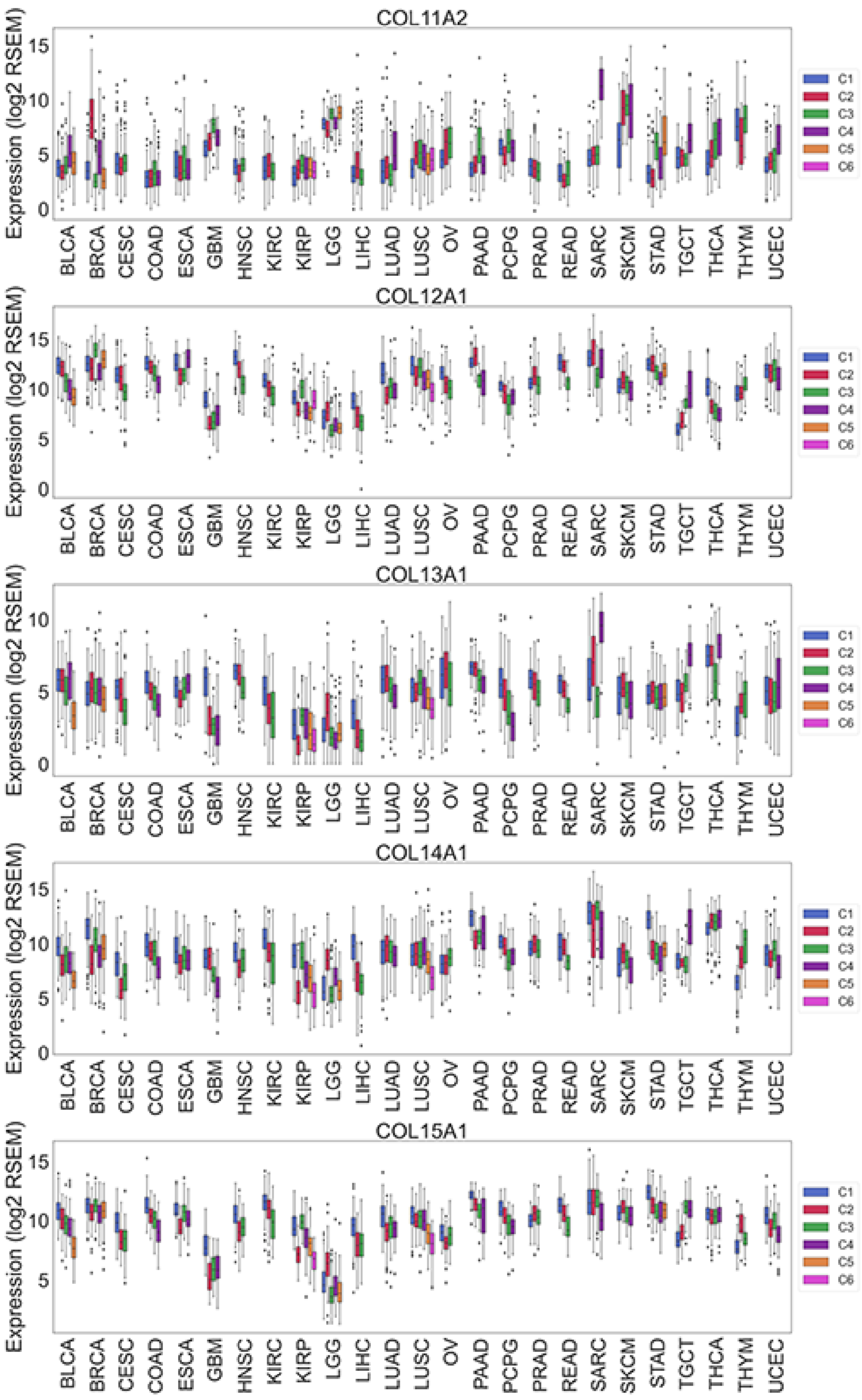

Supplemental Figure 3. Distribution of RSEM expression scores for each collagen gene in each cancer type in each ColCluster. Center line represents median and whiskers delineate interquartile range.

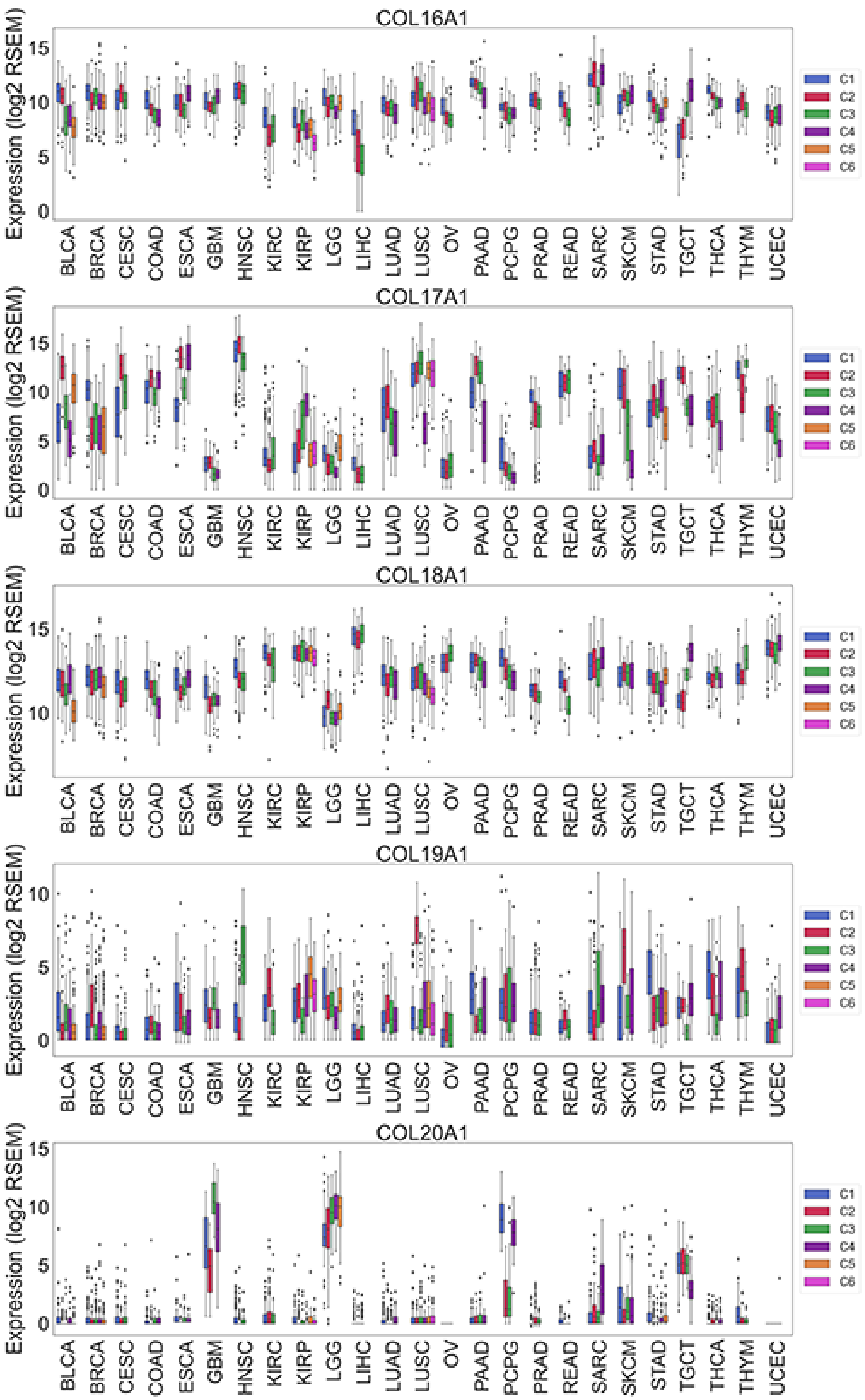

Supplemental Figure 3. Distribution of RSEM expression scores for each collagen gene in each cancer type in each ColCluster. Center line represents median and whiskers delineate interquartile range.

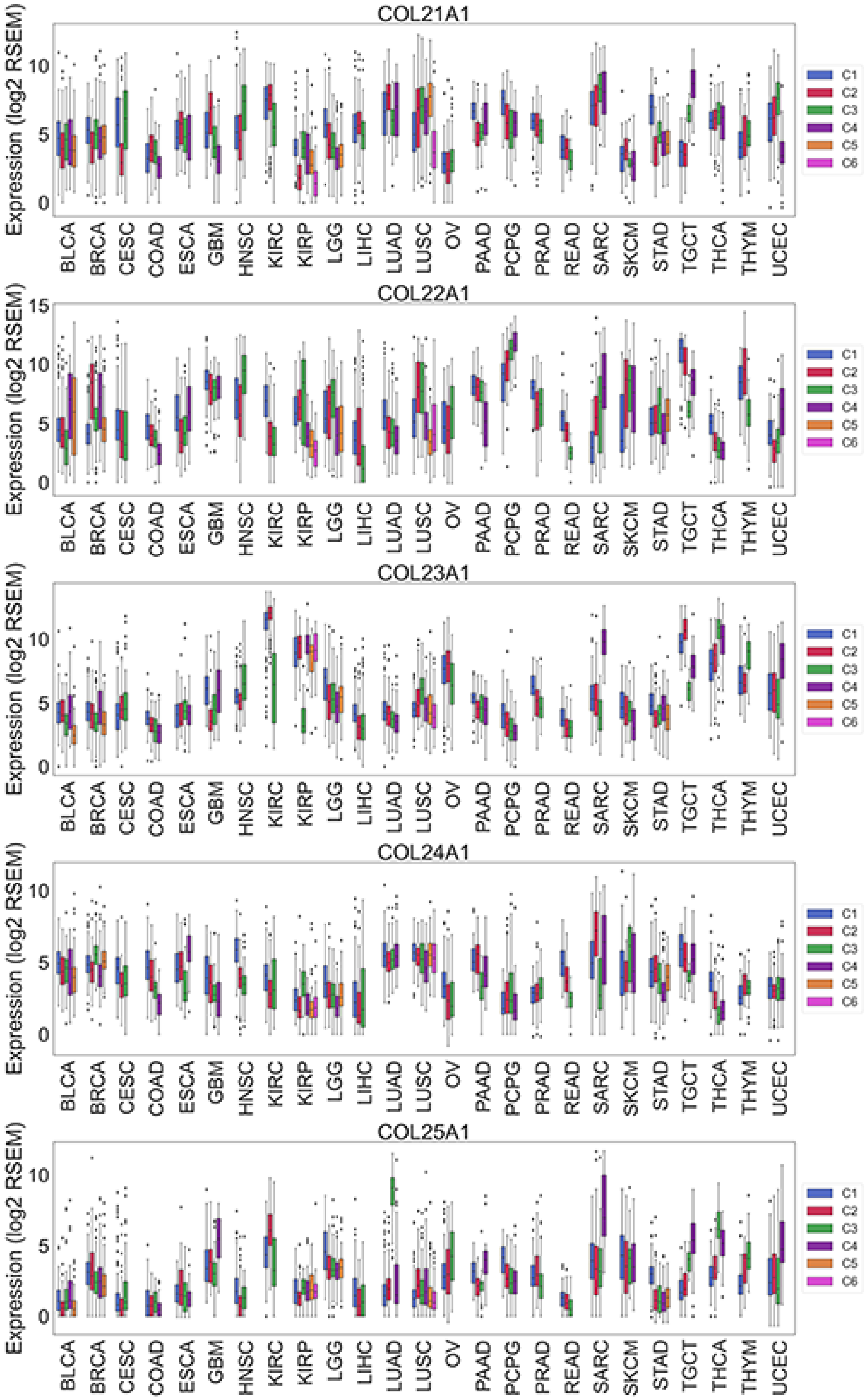

Supplemental Figure 3. Distribution of RSEM expression scores for each collagen gene in each cancer type in each ColCluster. Center line represents median and whiskers delineate interquartile range.

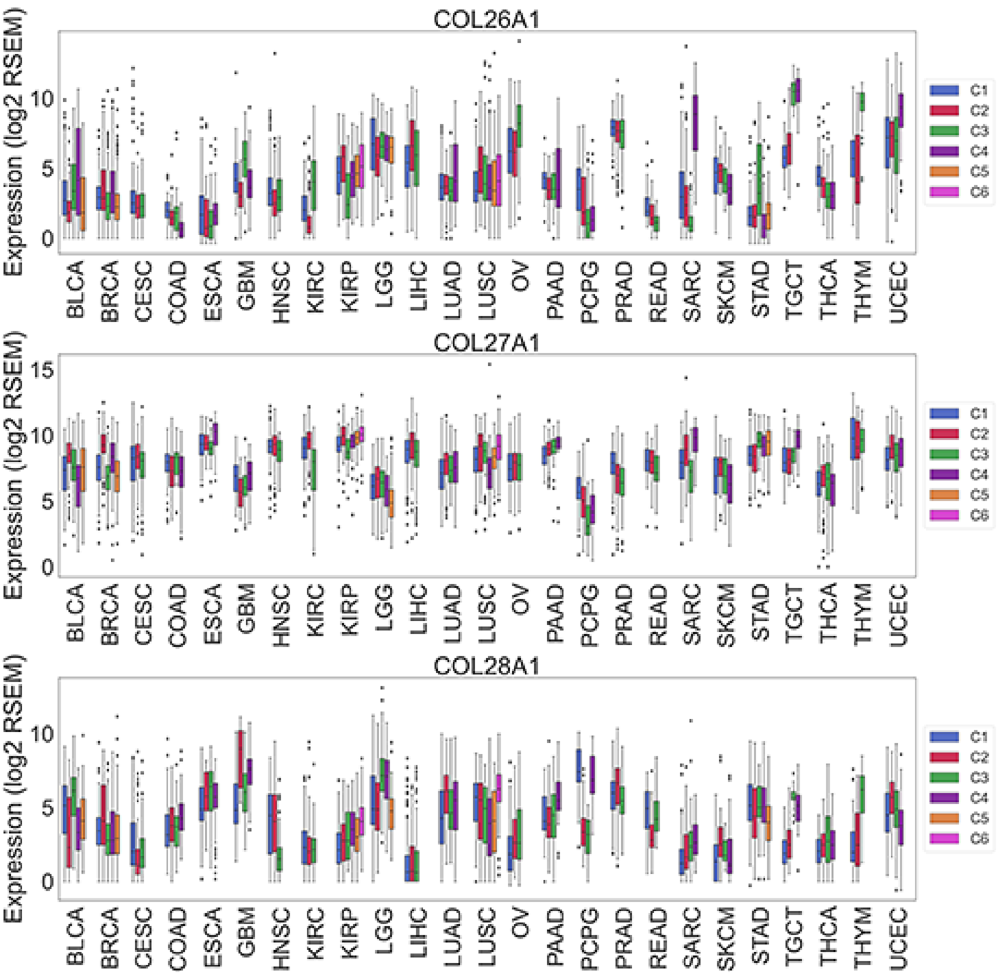

Supplemental Figure 3. Distribution of RSEM expression scores for each collagen gene in each cancer type in each ColCluster. Center line represents median and whiskers delineate interquartile range.

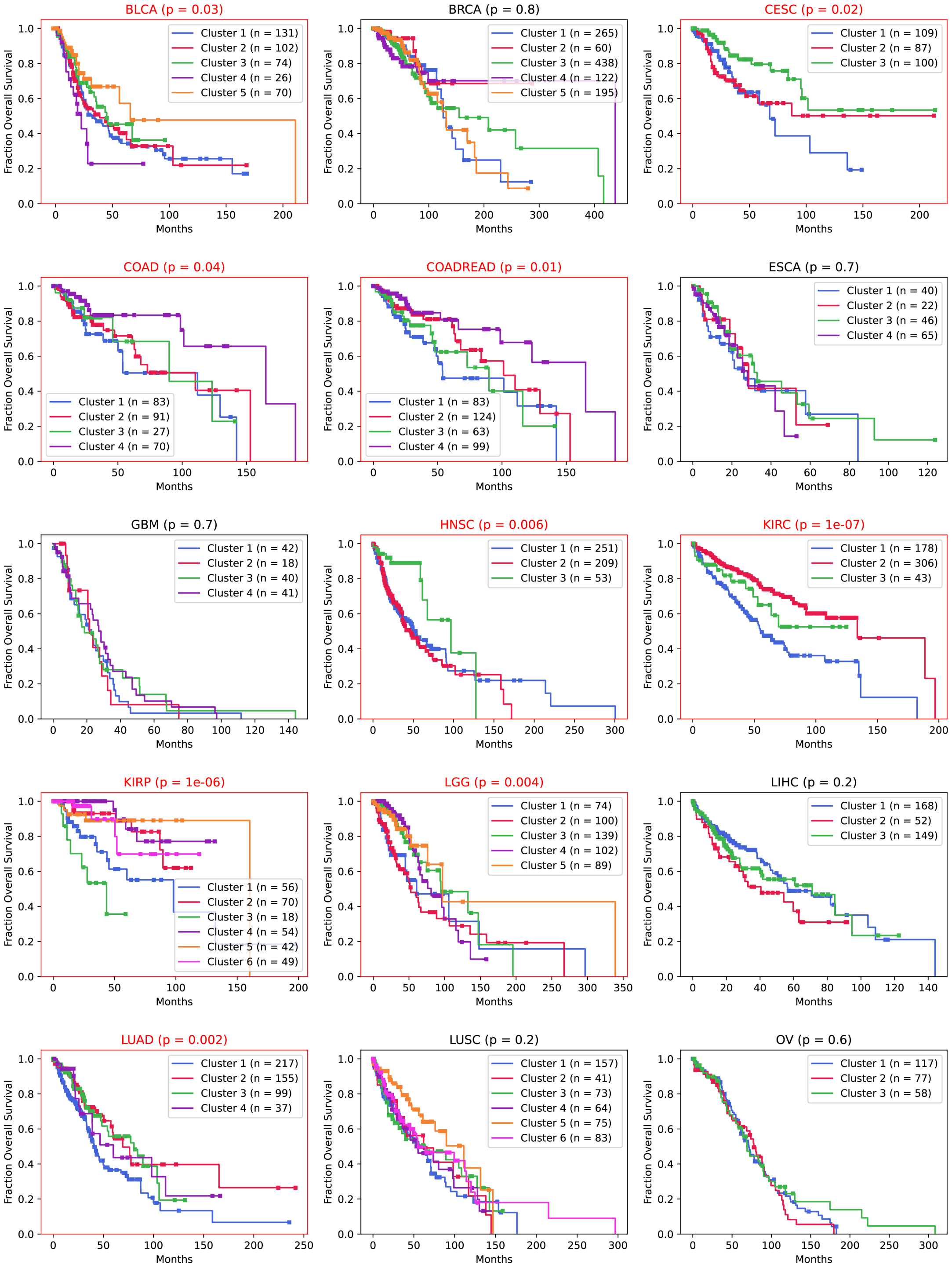

Supplemental Figure 4. Kaplan-Meier Curves for each tumor type for the ColClusters.

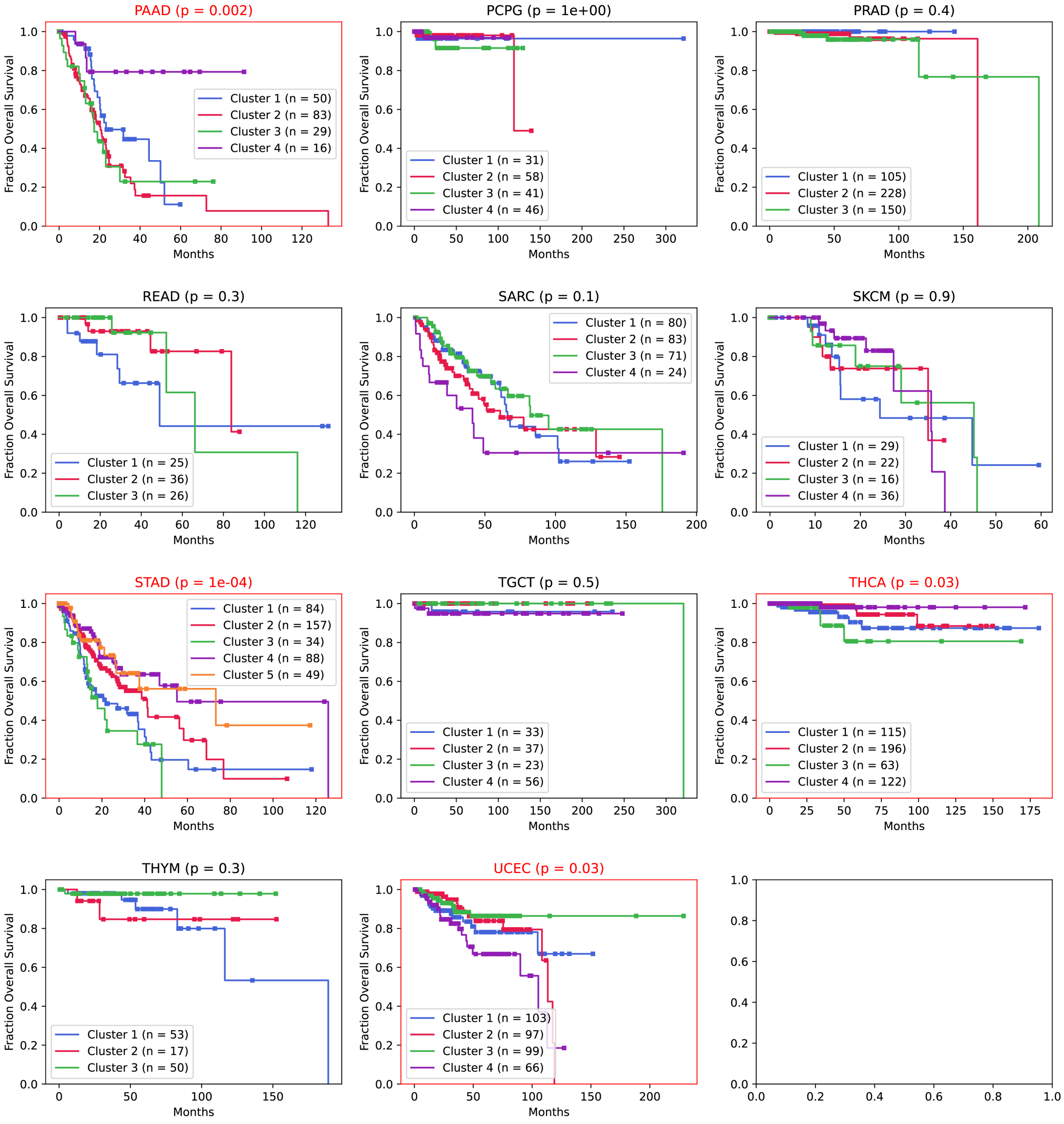

Supplemental Figure 4. Kaplan-Meier Curves for each tumor type for the ColClusters.

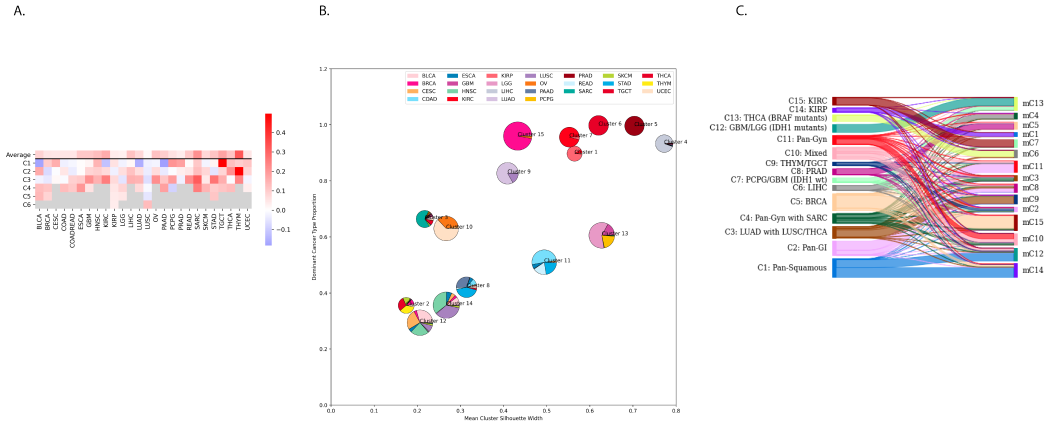

Supplemental Figure 5. Comparison of Matrisome vs. Collagen gene sets in classifying TCGA solid tumors PanCancer. (A) Mean cluster silhouette scores for each matrisome-defined tumor-type-specific cluster. (B) PanCan matrisome cluster robustness (mean cluster silhouette score) vs. composition (dominant cancer type proportion). Each cluster is plotted as a pie chart with slices indicating cluster tumor type composition. (C) Sankey diagram showing correspondence between the ColClusters (left) and matrisome clusters (right).

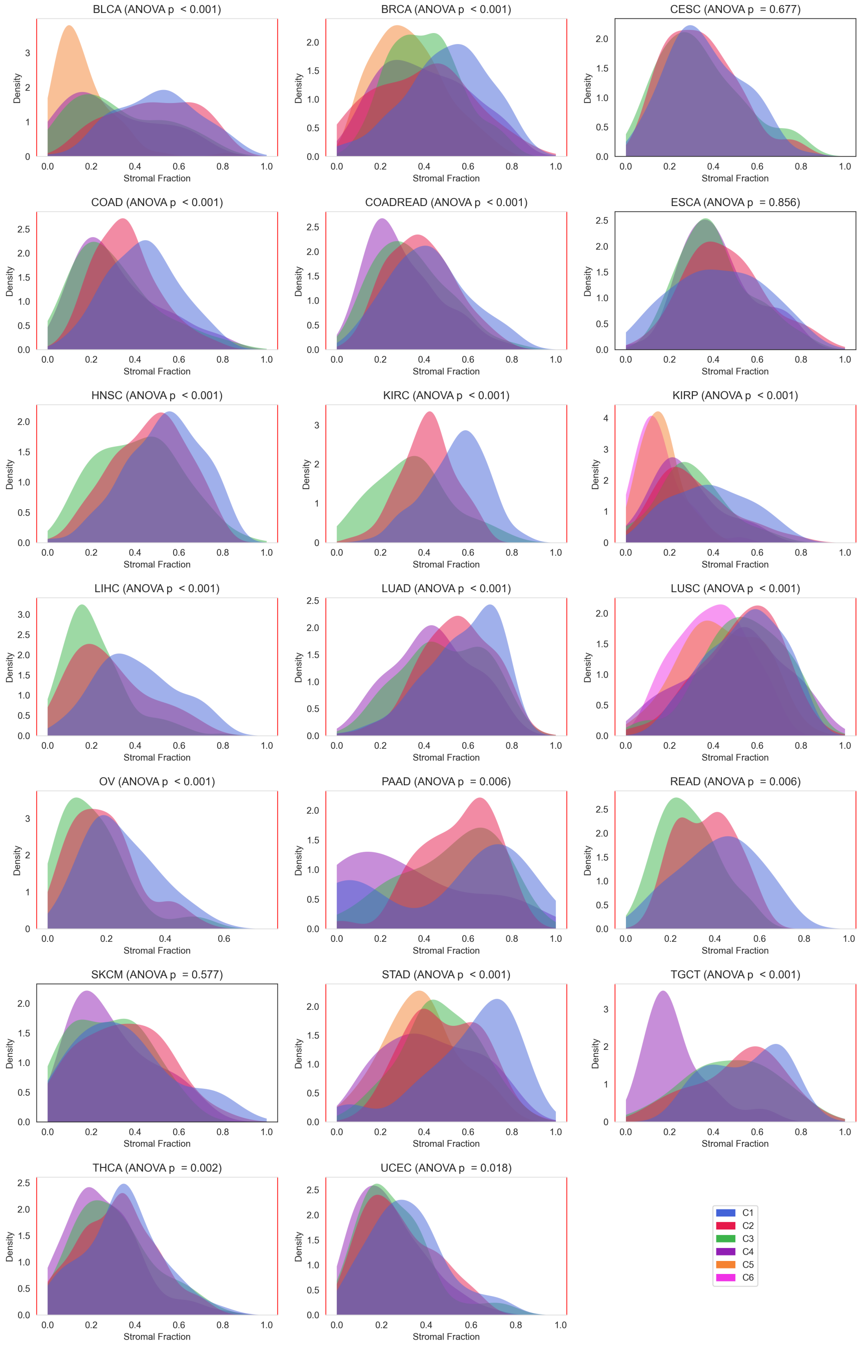

Supplemental Figure 6. Distributions of stroma fraction in each ColCluster.

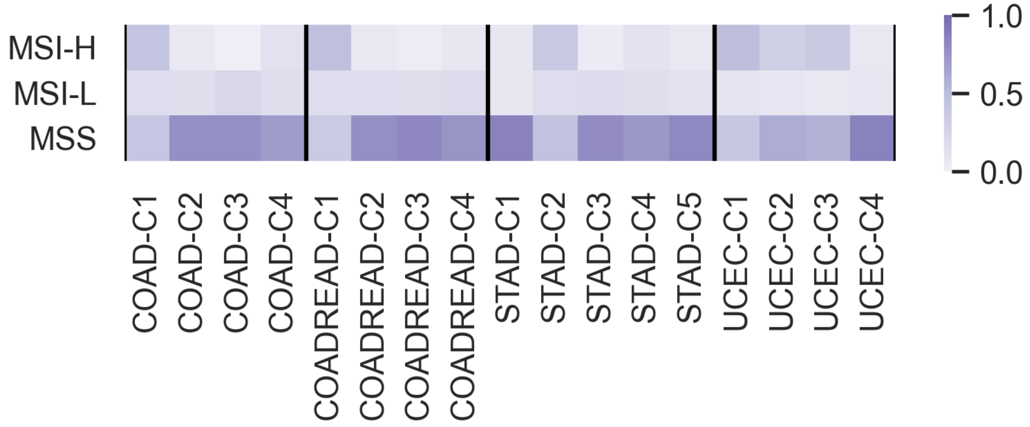

Supplemental Figure 7. Distributions of MSIH and MSS tumors in each ColCluster for the relevant cancer types. Fraction of the tumor in each ColCluster is shown.

Supplemental Figure 8. Gene Number Alterations at the gene level were enriched in specific ColClusters. (A) Fraction of tumors in each ColCluster with gene copy number amplifications (orange) or deletions (green). (B) Gene copy number changes that were significantly biased across the ColClusters as determined by Chi-squared tests. Heat map indicates negative log p values.

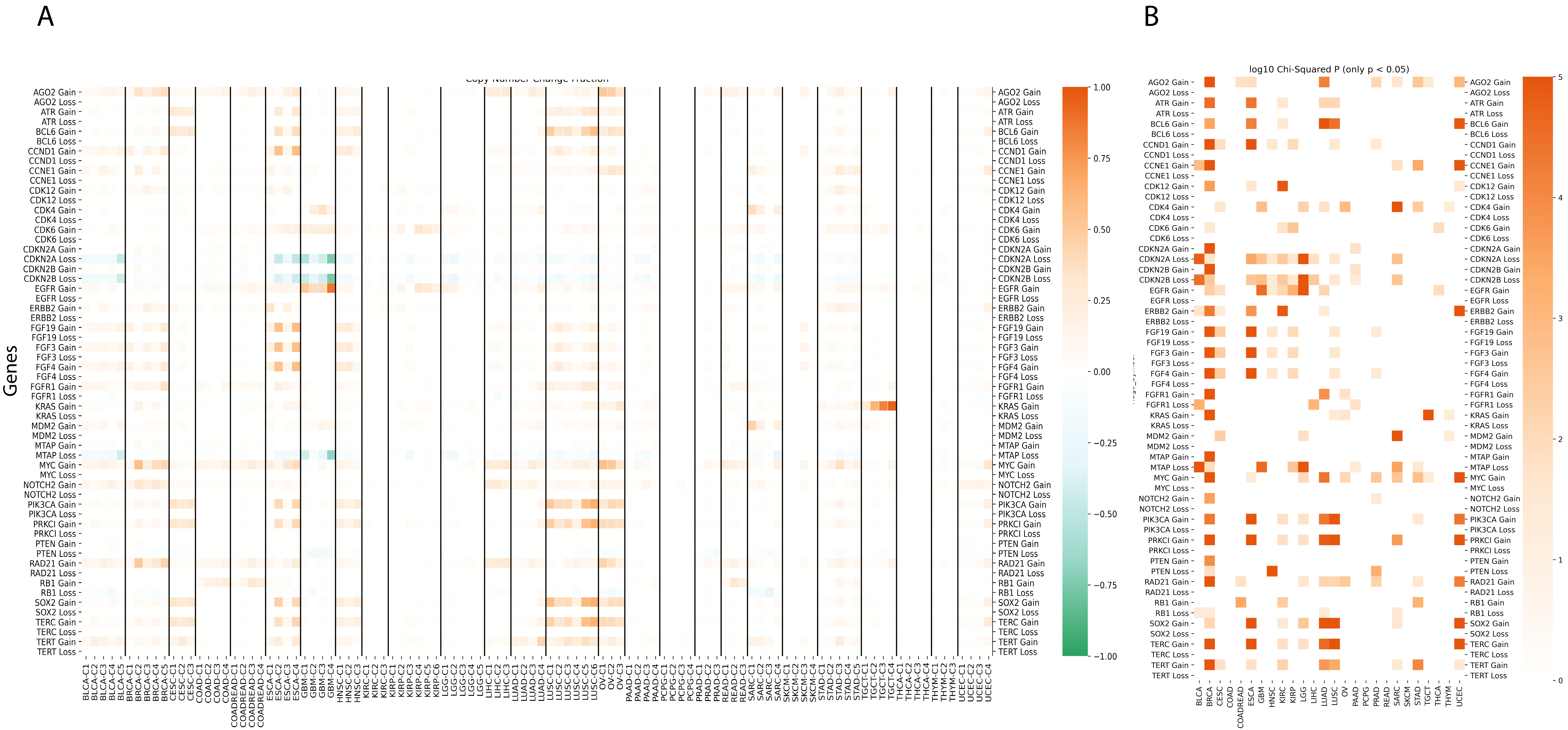
A
Copy Number Change Fraction
Genes
B
log10 Chi-Squared P (only p < 0.05)

Supplemental Figure 9. Copy Number Alterations at the chromosome arm level were enriched in specific ColClusters. (A) Fraction of tumors in each ColCluster with chromosome arm amplification (orange) or deletion (green). (B) Chromosome arm copy number changes that were significantly biased across the ColClusters as determined by Chi-squared tests. Heat map indicates negative log p values.

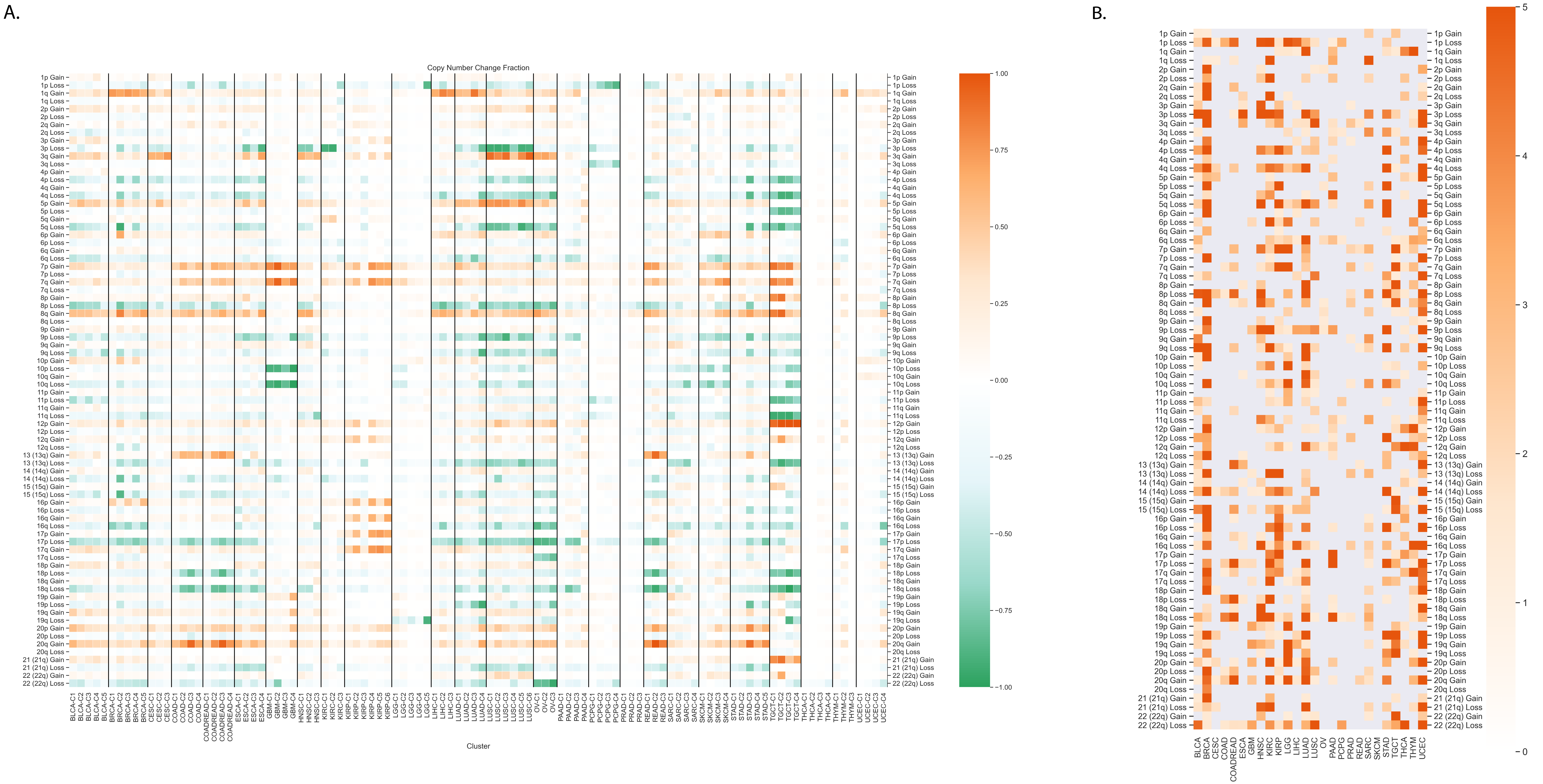
A.
Copy Number Change Fraction
Cluster
1.00
0.75
0.50
0.25
0.00
−0.25
−0.50
−0.75
−1.00
B.
BLCA
BRCA
CESC
COAD
COADREAD
ESCA
GBM
HNSC
KIRC
KIRP
LGG
LIHC
LUAD
LUSC
OV
PAAD
PCPG
PRAD
READ
SARC
SKCM
STAD
TGCT
THCA
THYM
UCEC
5
4
3
2
1
0

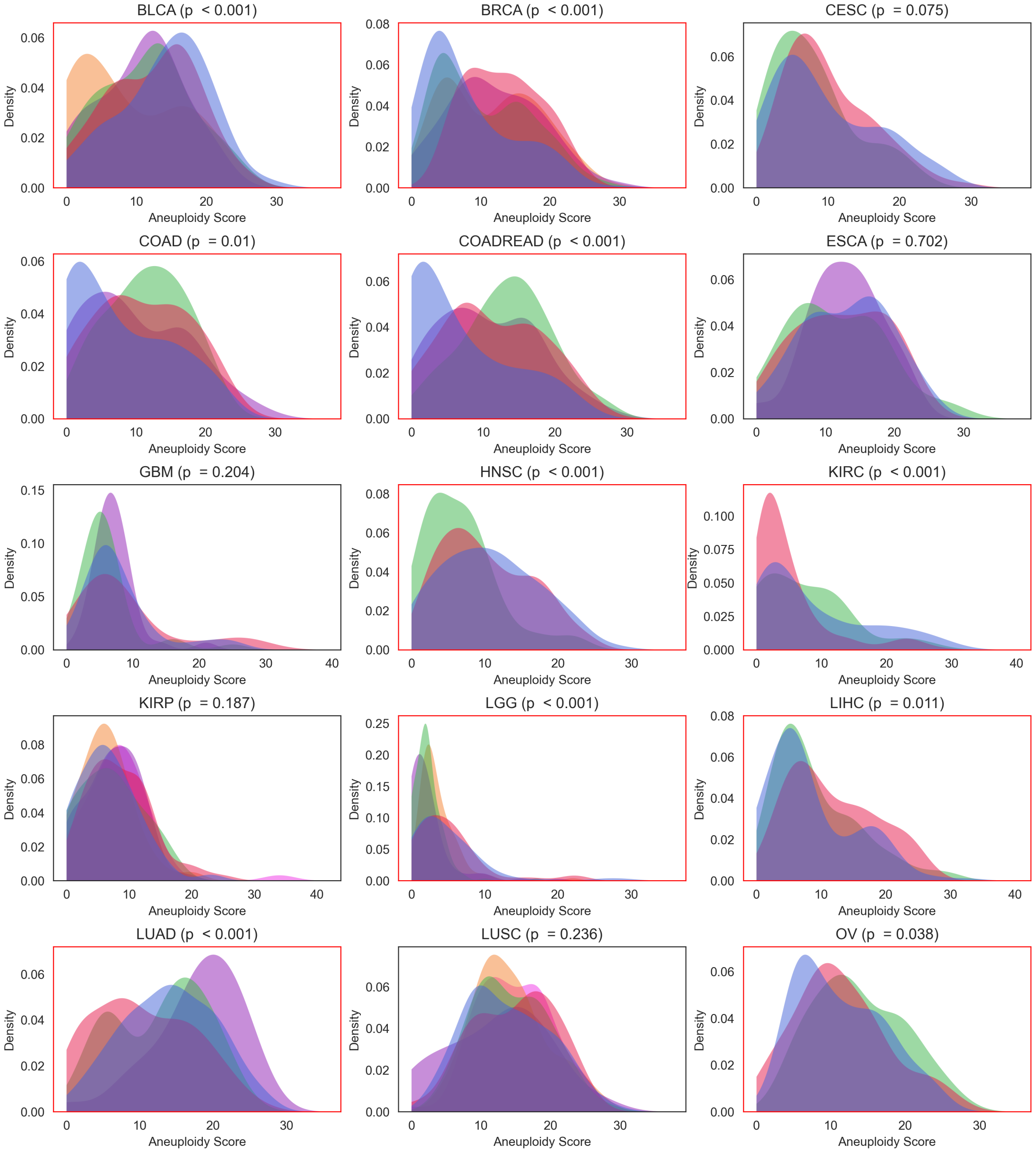

Supplemental Figure 10. Distribution of aneuploidy scores in each ColCluster.

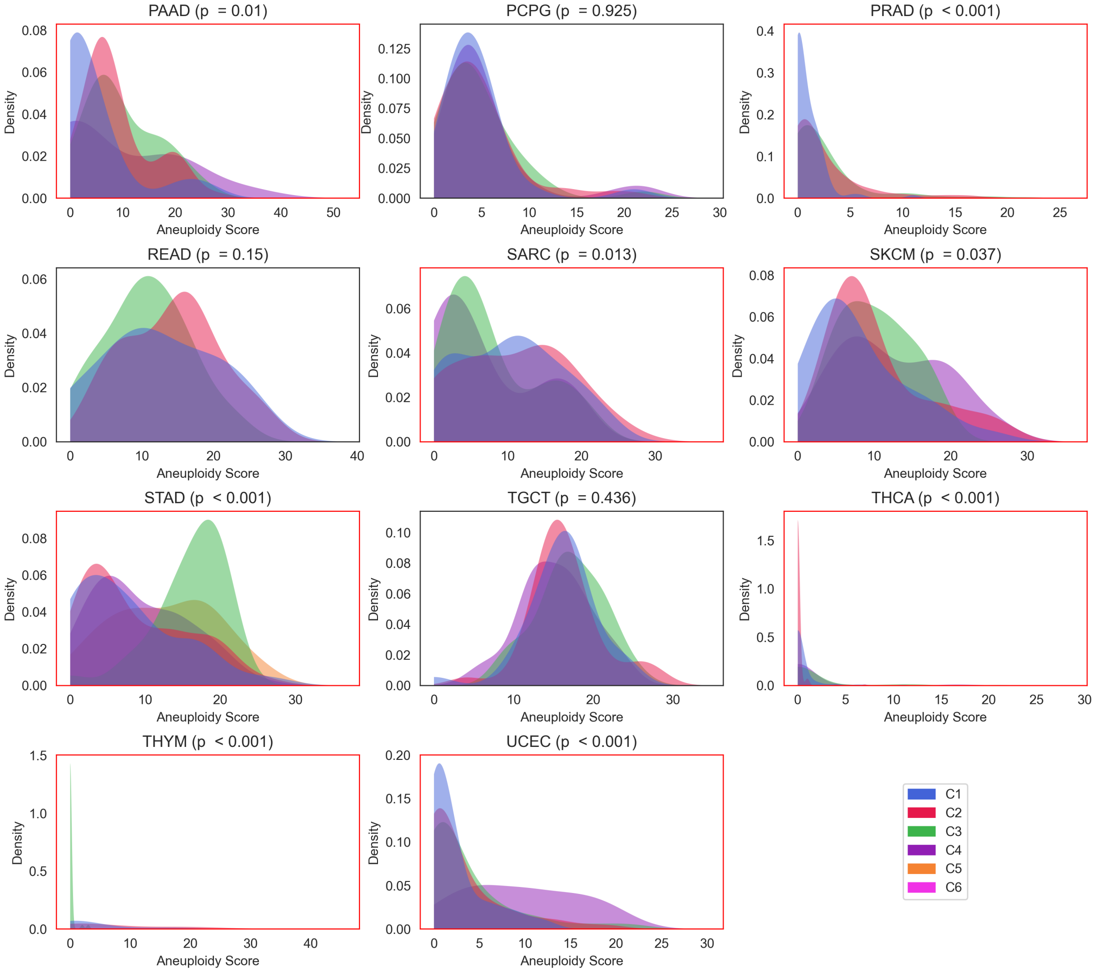

Supplemental Figure 10. Distribution of aneuploidy scores in each ColCluster.

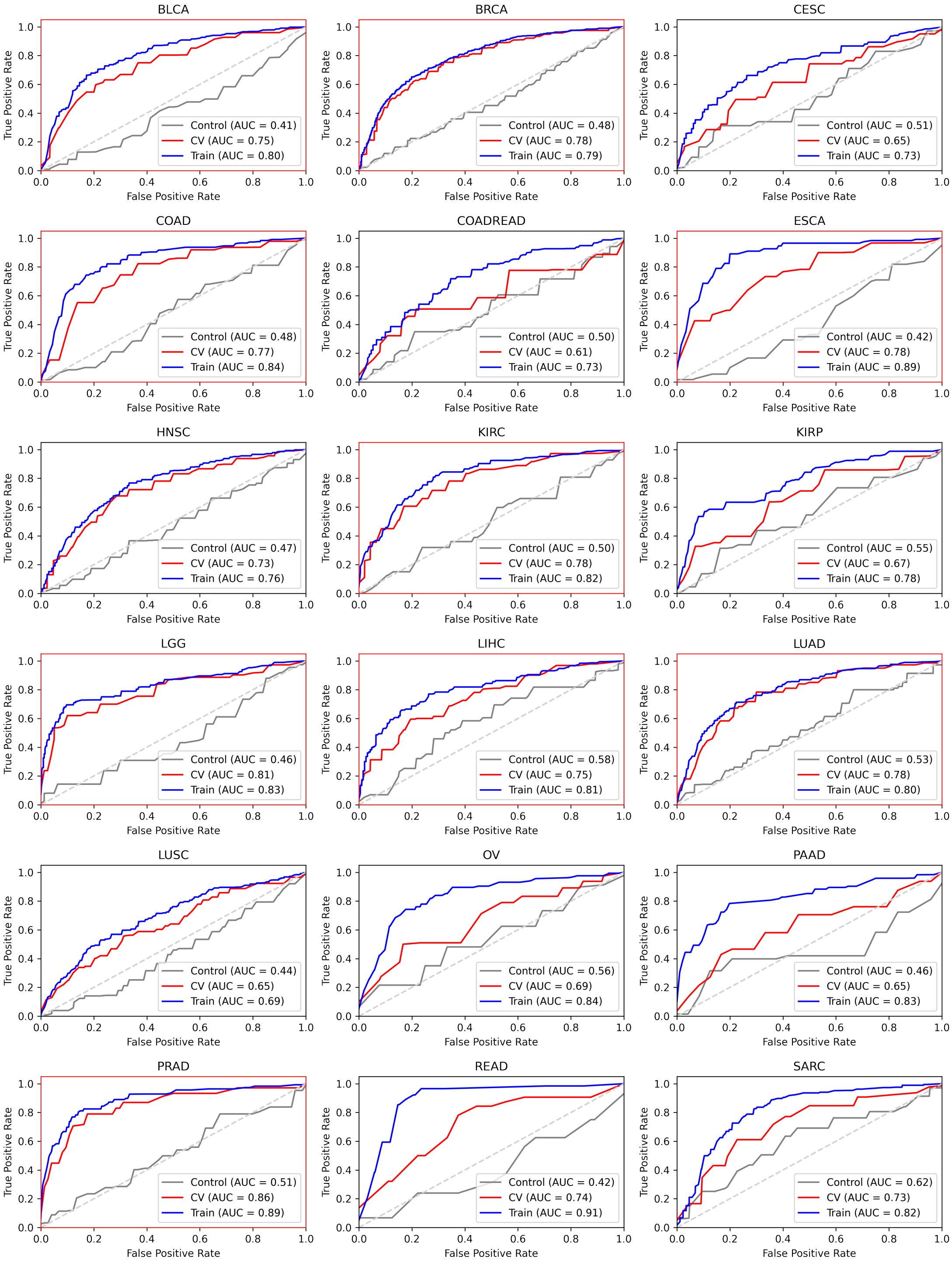

Supplemental Figure 11. Representative reciever-operator curves (ROC) of SVM predictions of aneuploidy from collagen RNA expression in TCGA in each cancer sub-type.

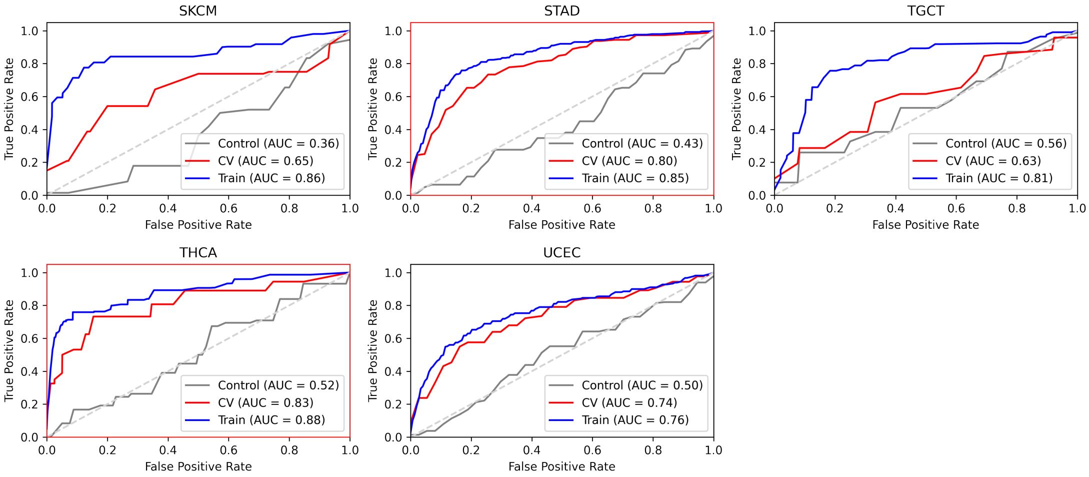

Supplemental Figure 11. Representative reciever-operator curves (ROC) of SVM predictions of aneuploidy from collagen RNA expression in TCGA in each cancer sub-type.

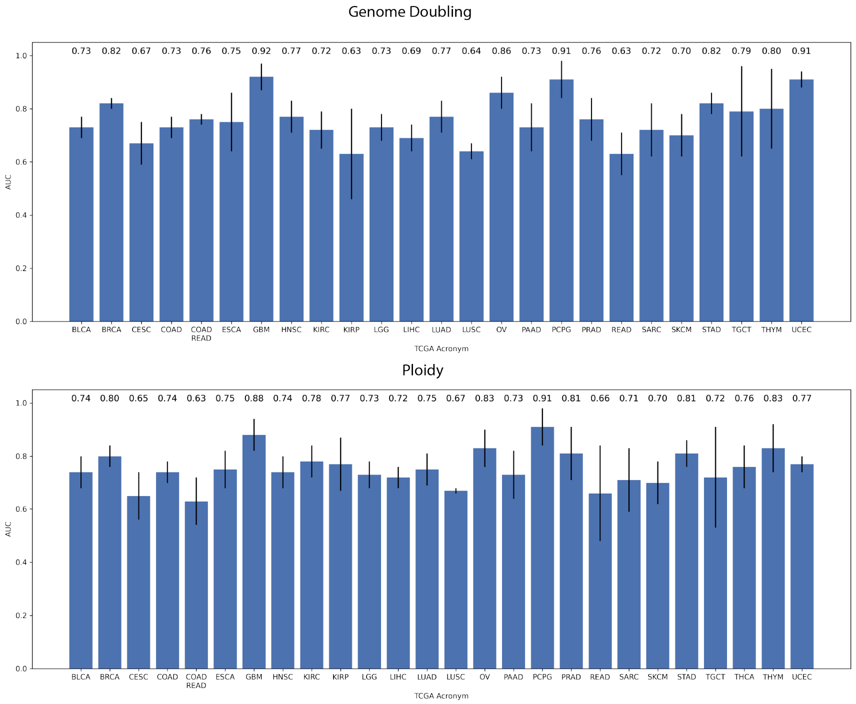

Supplemental Figure 12. Summary of mean AUC scores from genome doubling and ploidy SVM model cross-validation for each tumor type. Error bars represent the standard deviation of the cross-validation folds.

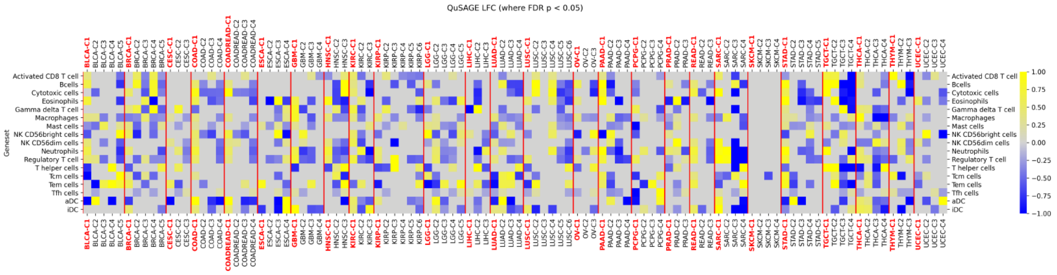

Supplemental Figure 13. QuSAGE analysis of immune cell expression enrichment in each ColCluster. Abbreviations: NK, Natural Killer; Tcm, Central Memory T cells; Tem, Effector Memory T cells; Tfh, T Follicular Helper cells; aDC, Activated Dendritic cells; iDC, Interstitial Dendritic cells.

figures/SummaryHeatMaps_All.png

Supplemental Figure 14. Summary heat maps for each cancer type showing collagen expression, genetic features, and the 50 hallmark gene sets as determined by ssGSEA in each tumor in each ColCluster. See file for Figure S12.

Supplemental Figure S14

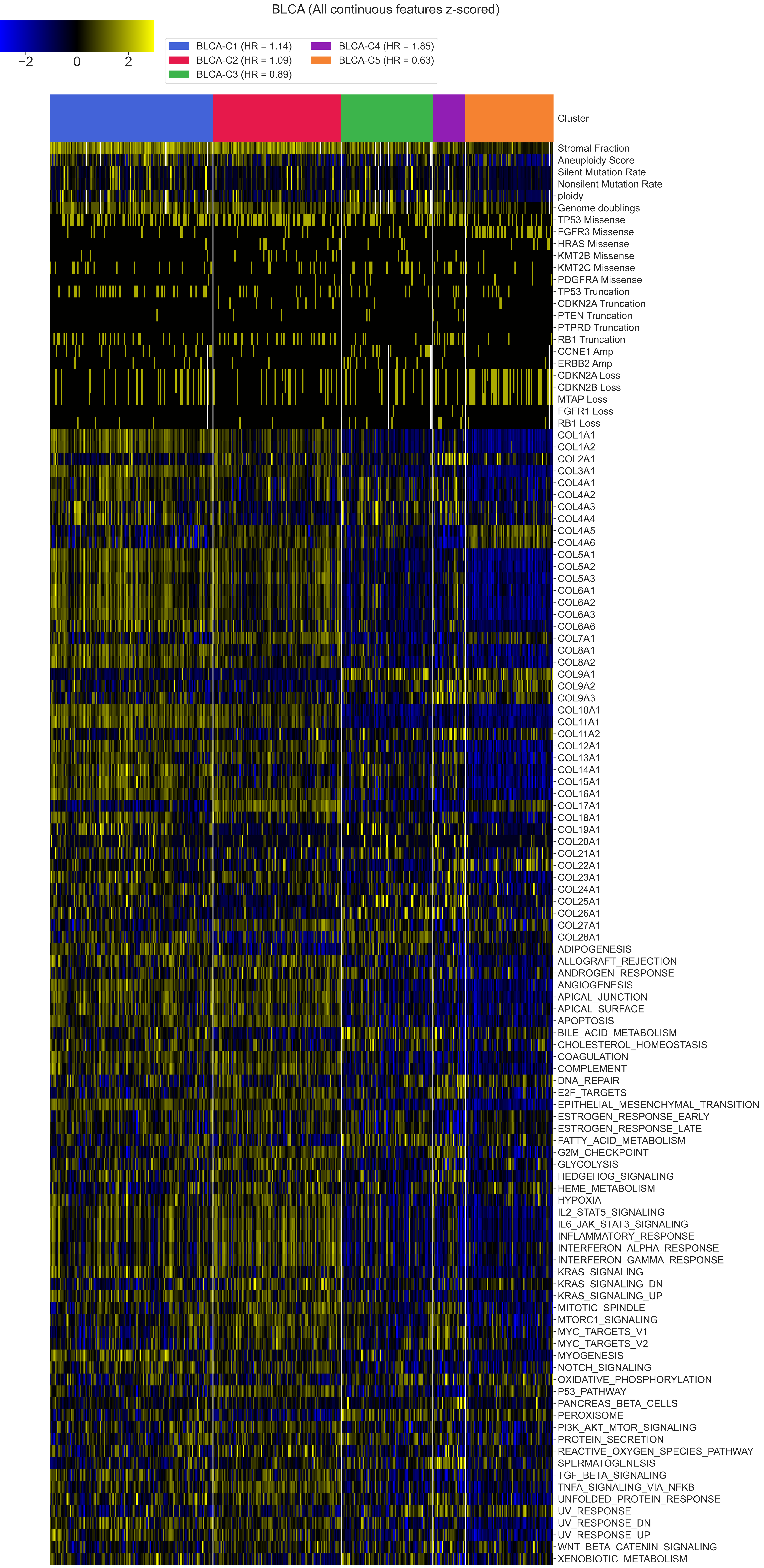

Supplemental Figure S14

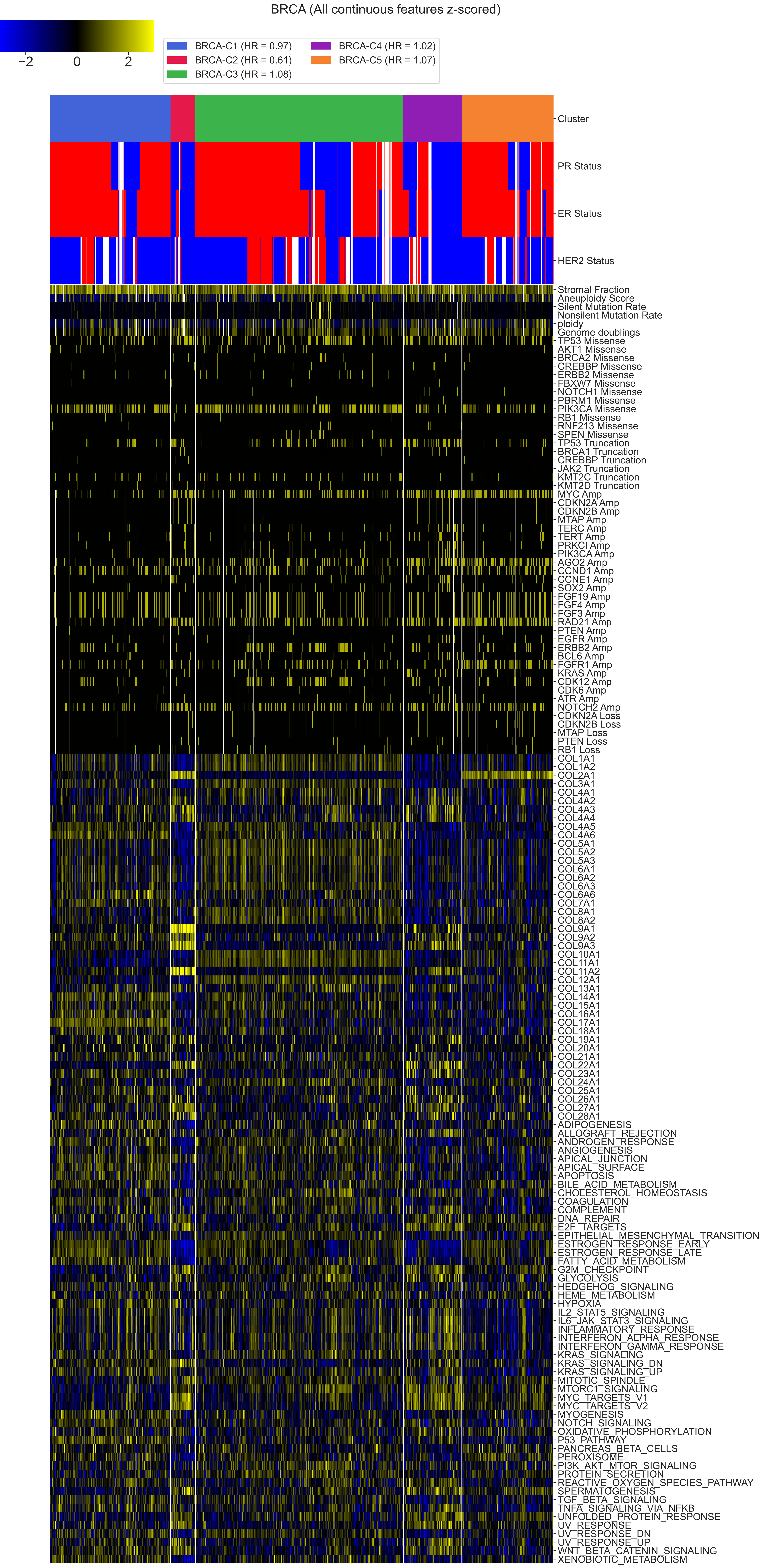

Supplemental Figure S14

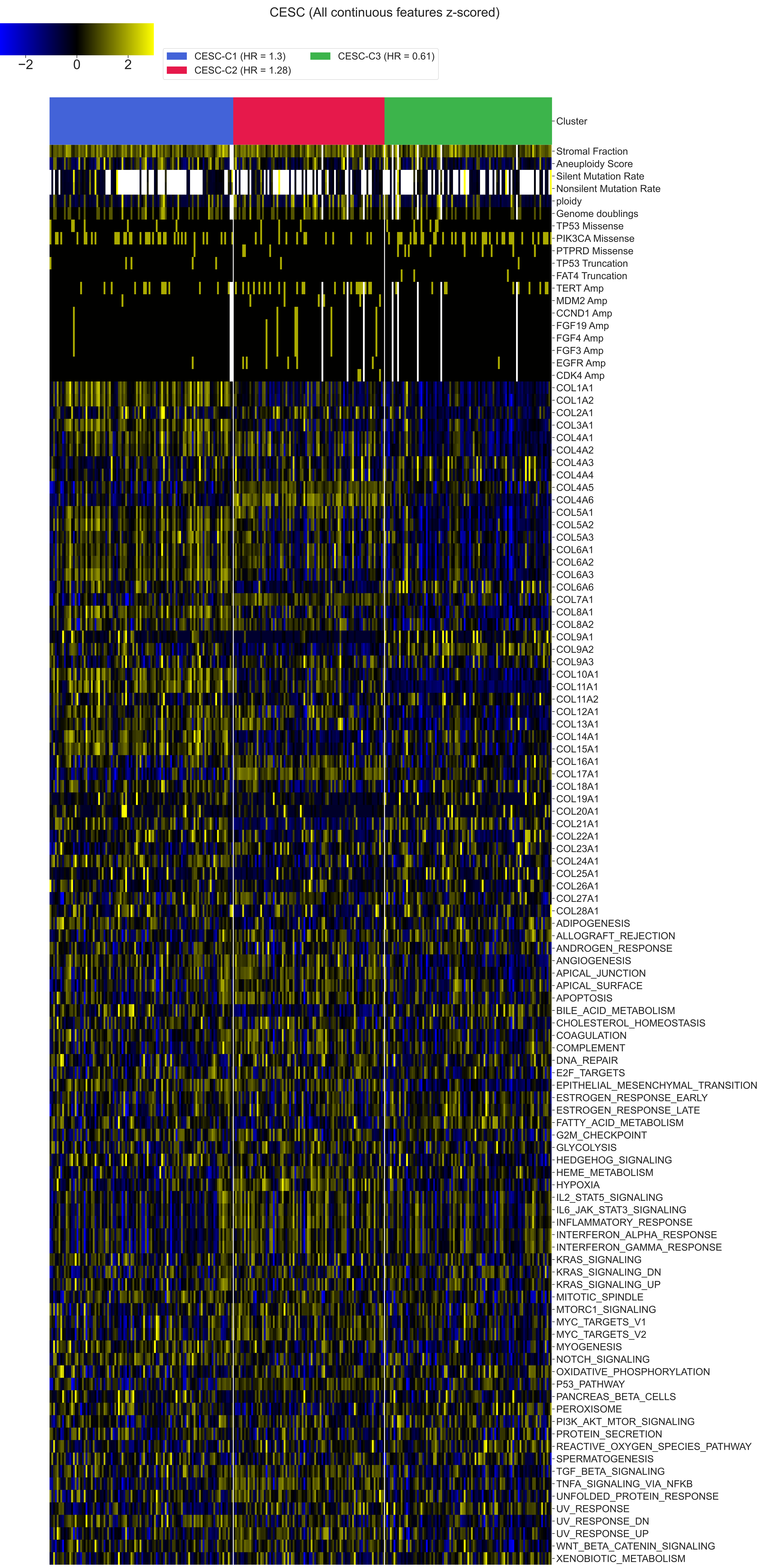

Supplemental Figure S14

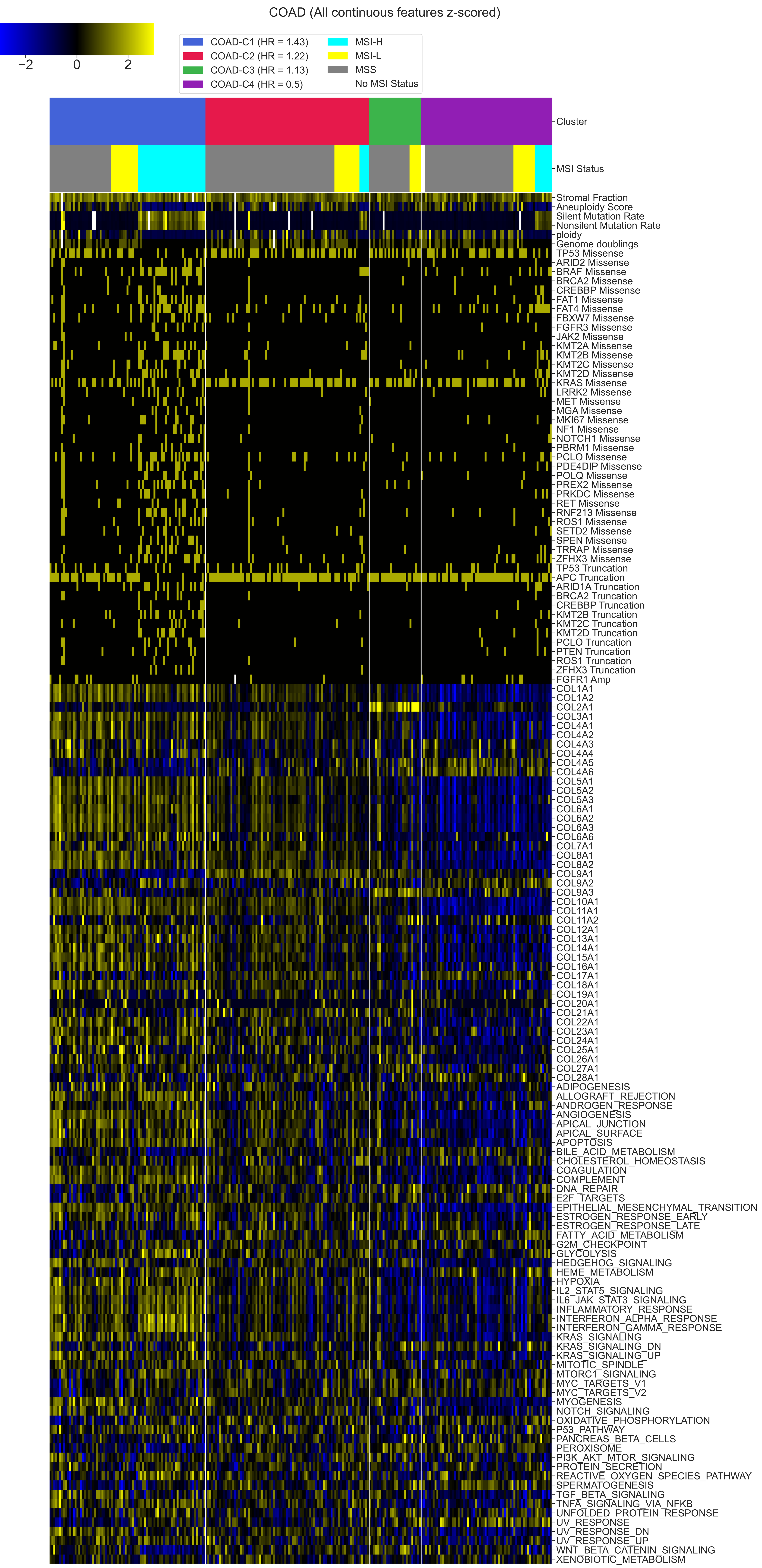

Supplemental Figure S14

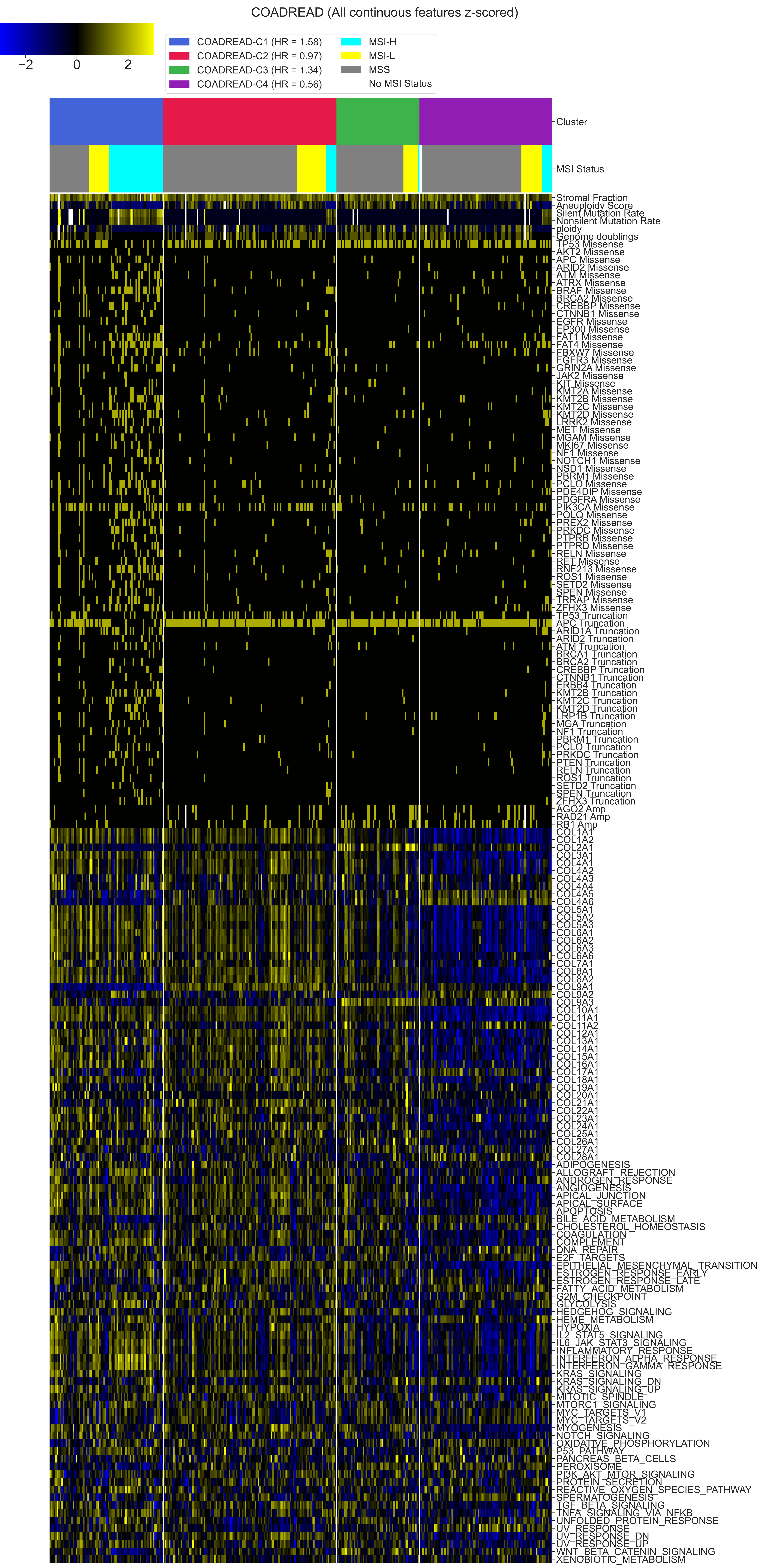

Supplemental Figure S14

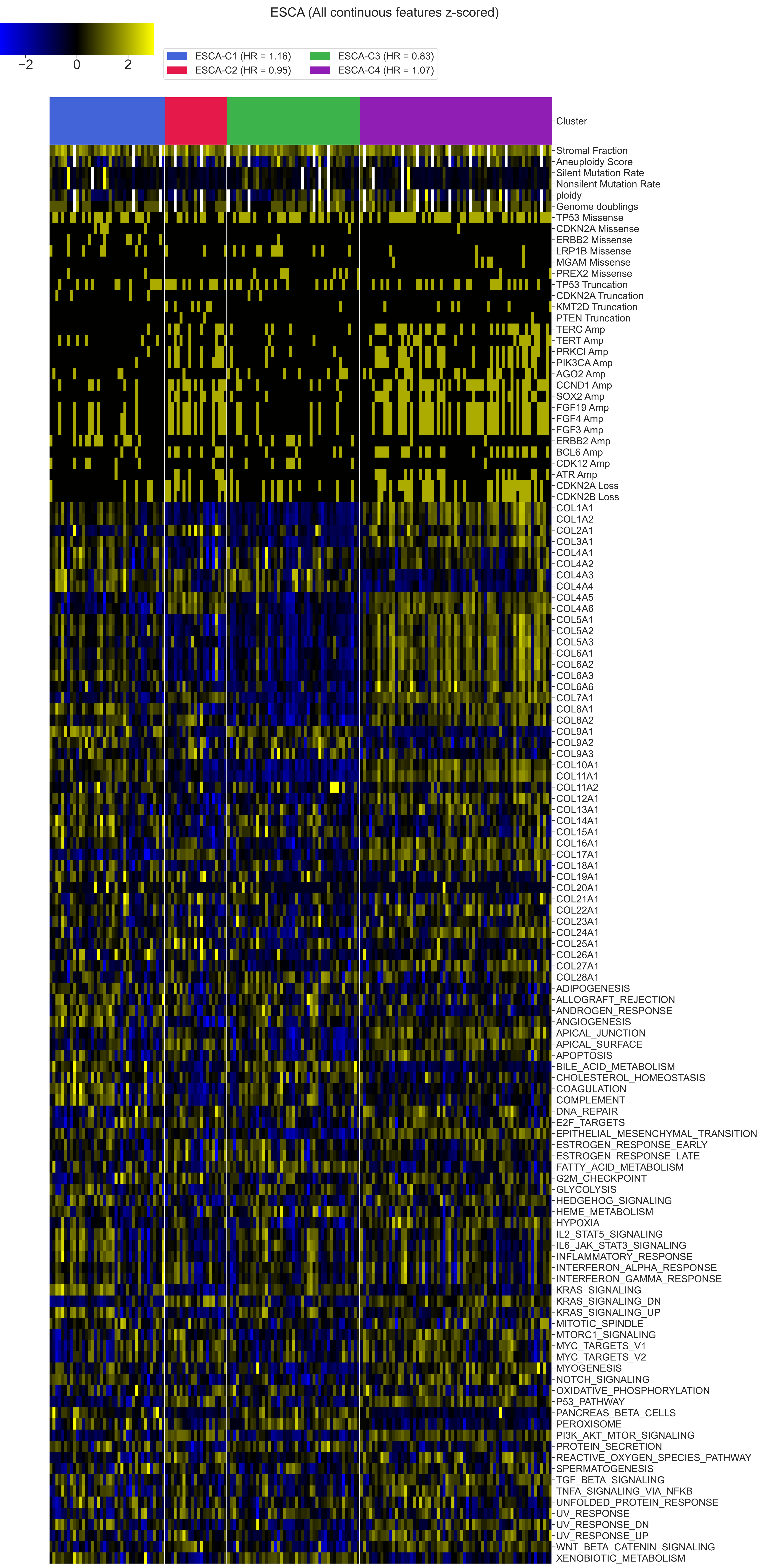

Supplemental Figure S14

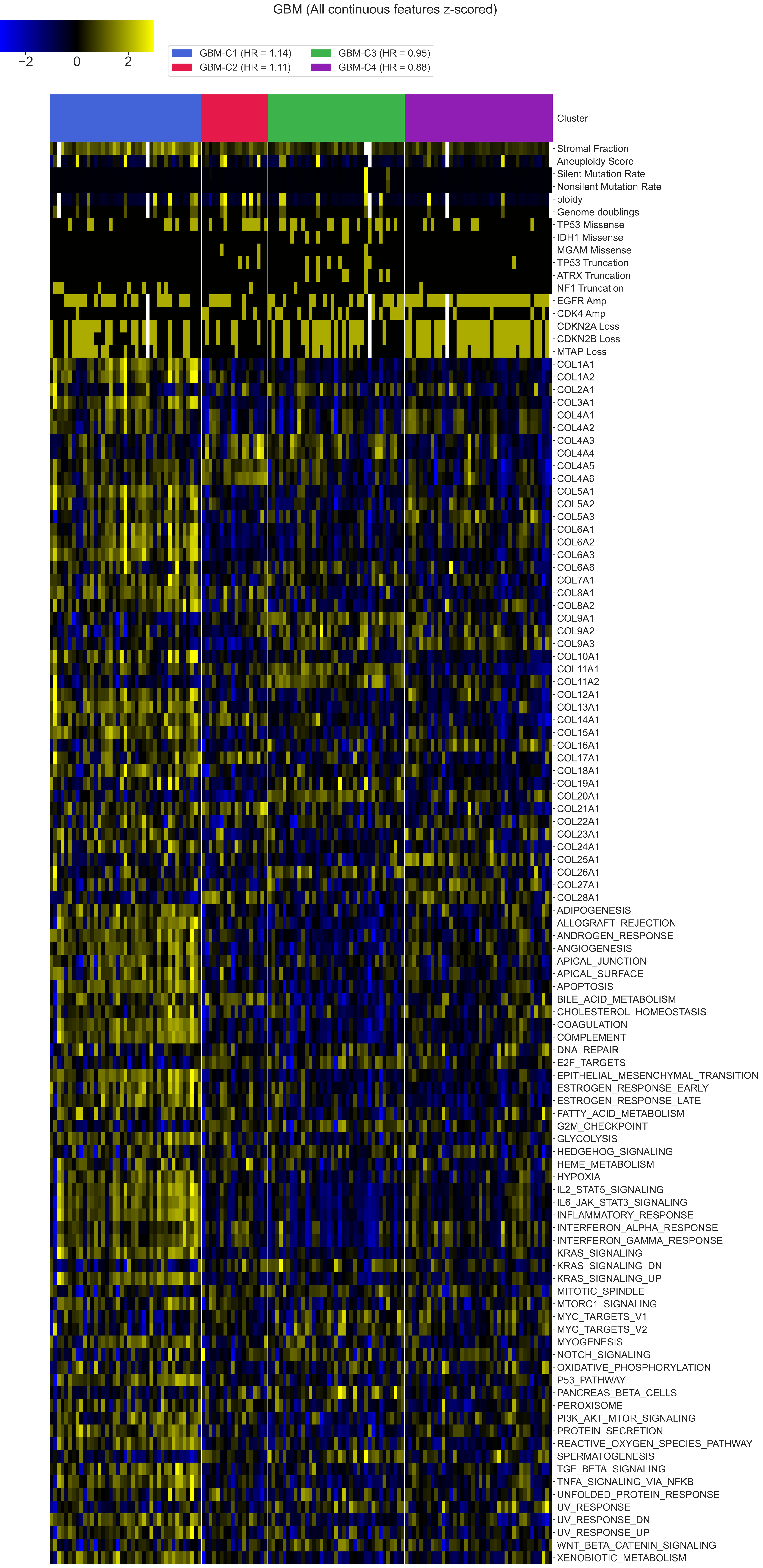

Supplemental Figure S14

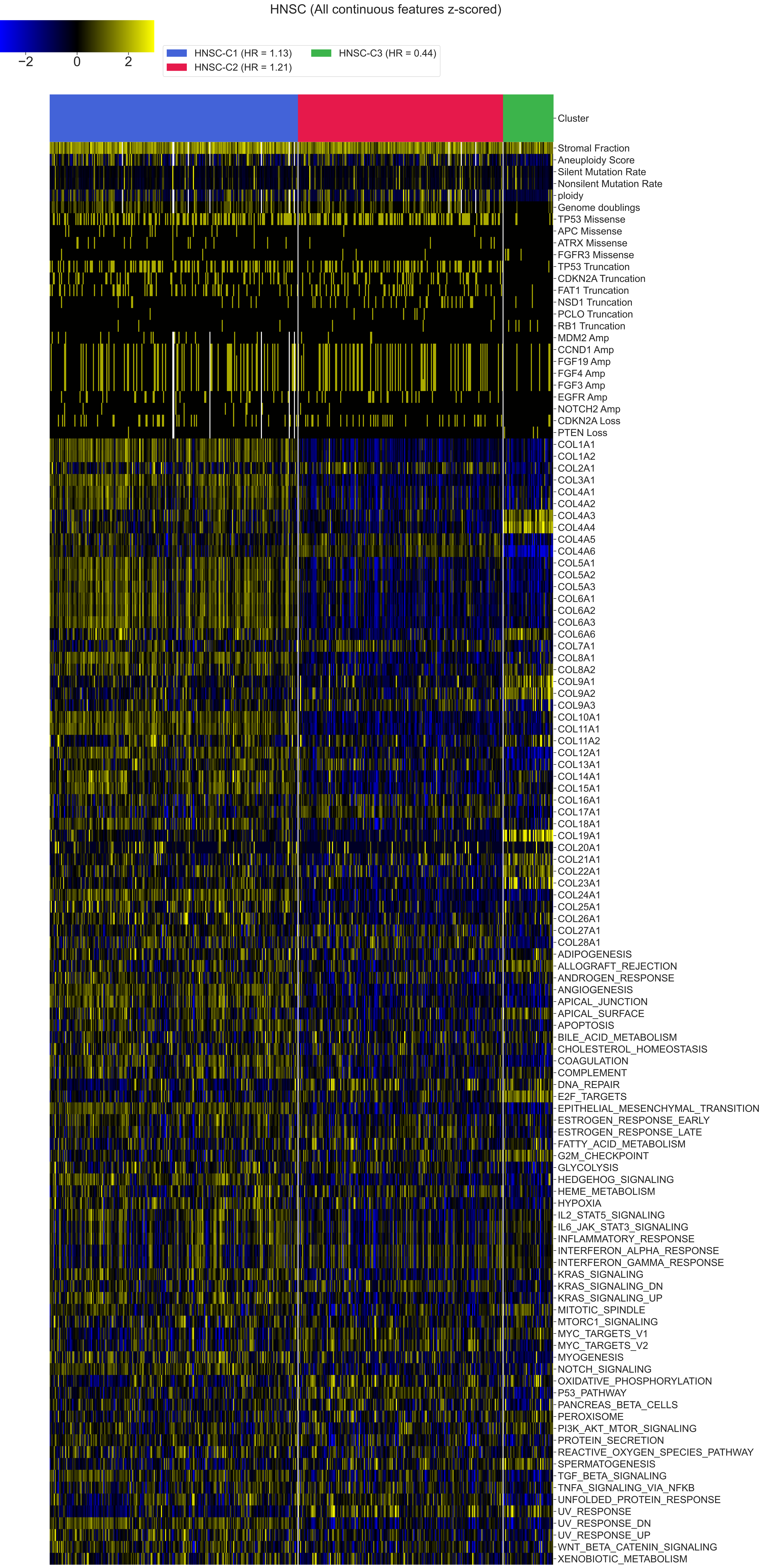

Supplemental Figure S14

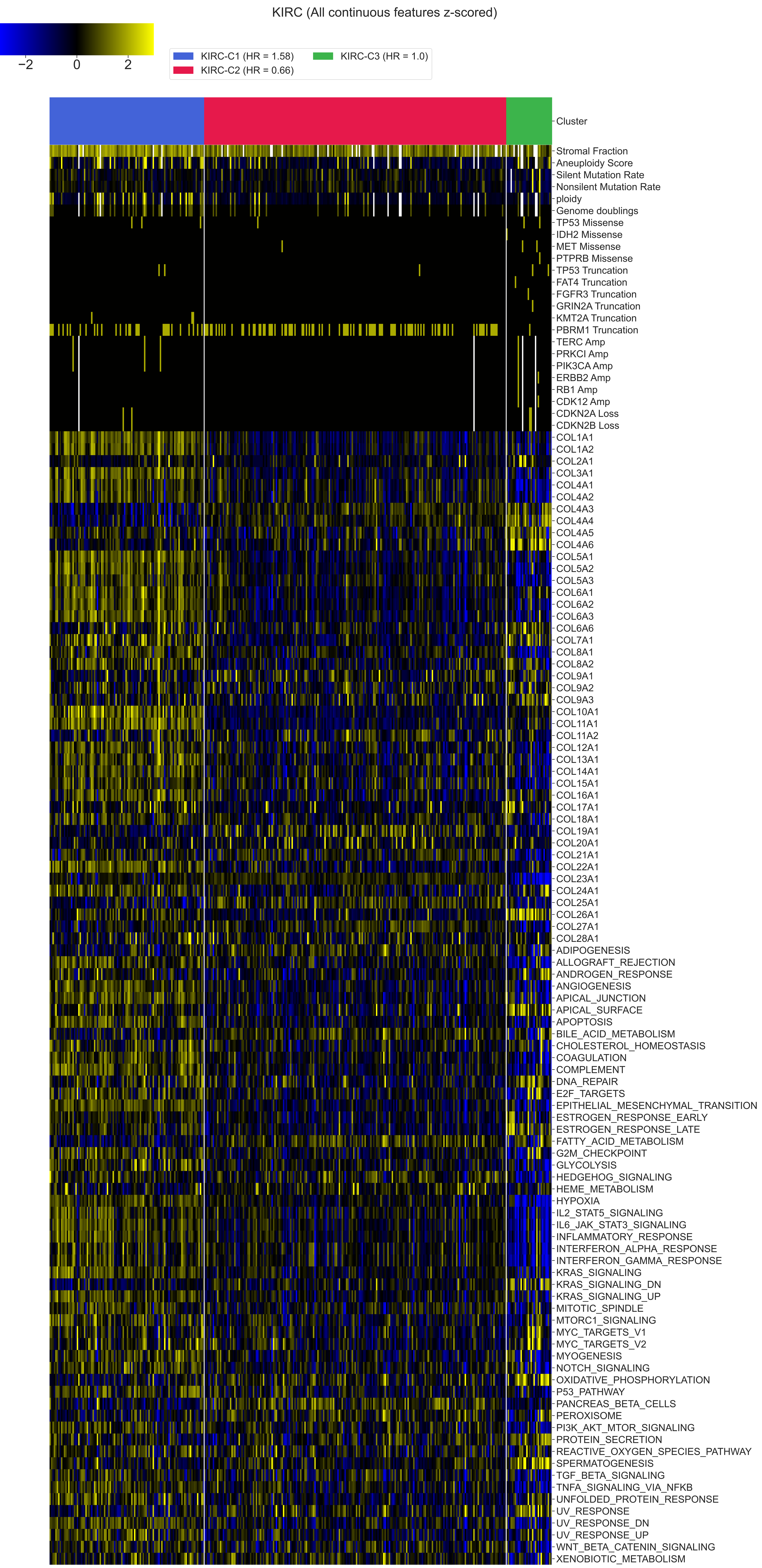

Supplemental Figure S14

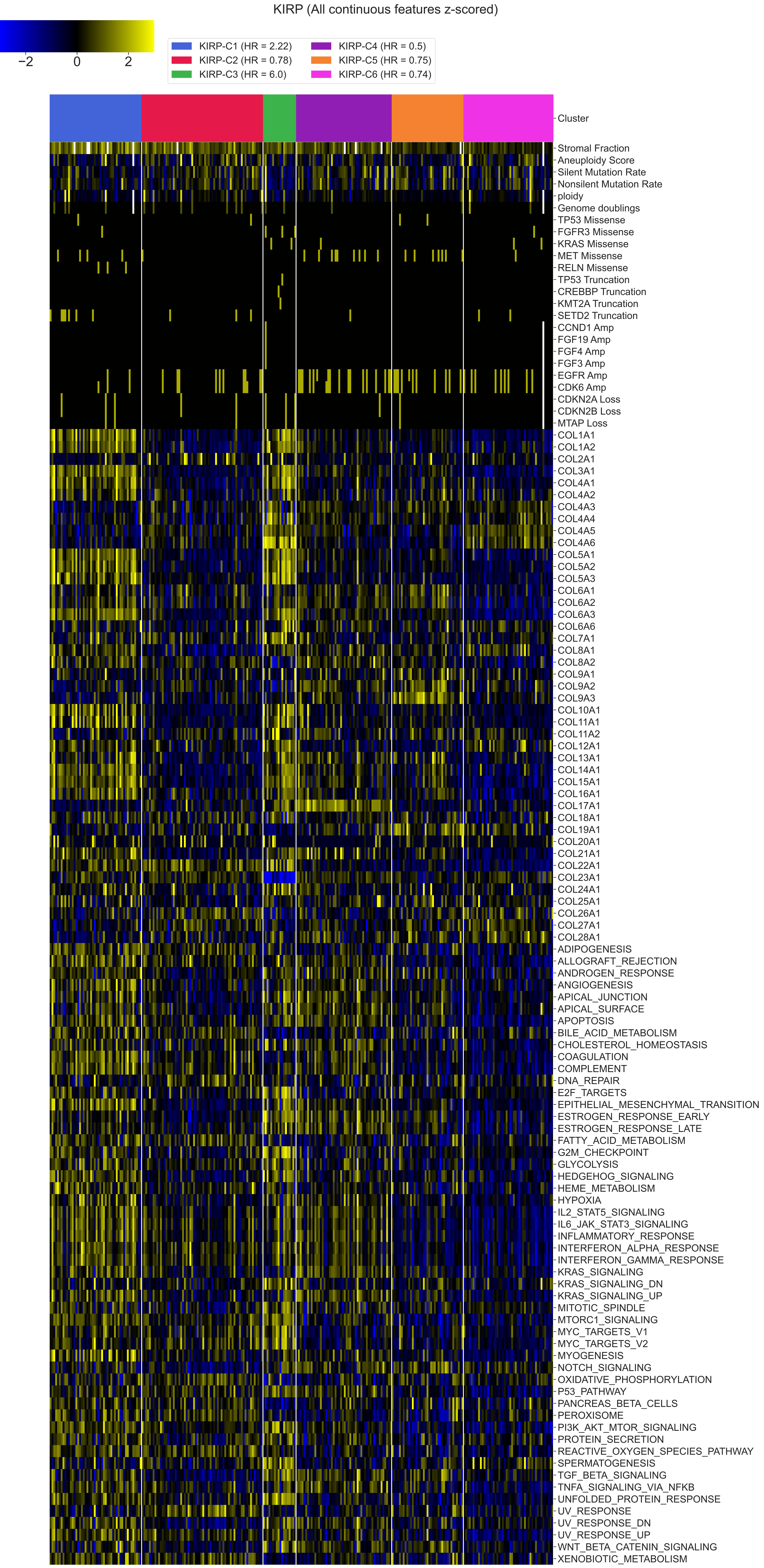

Supplemental Figure S14

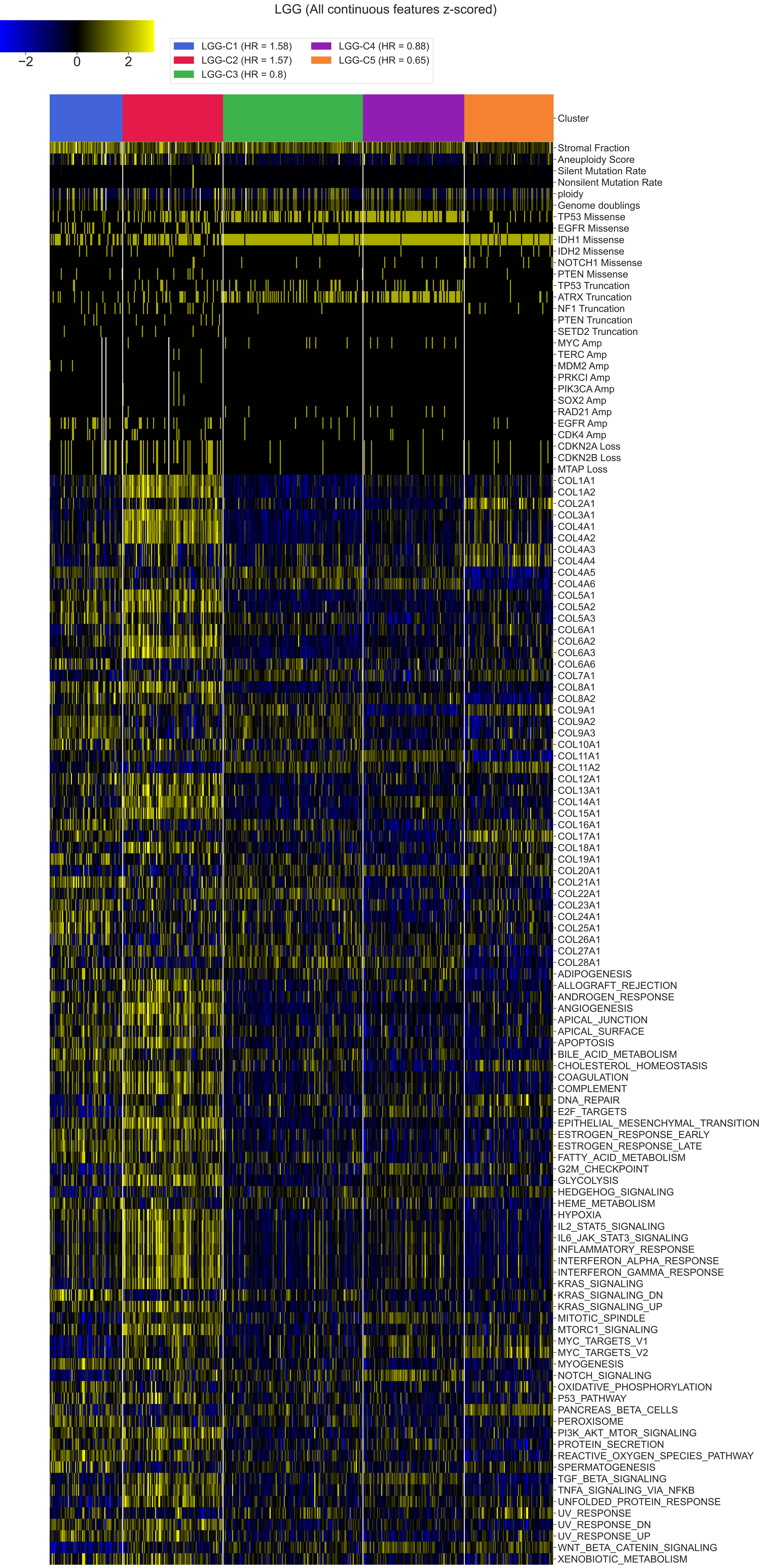

Supplemental Figure S14

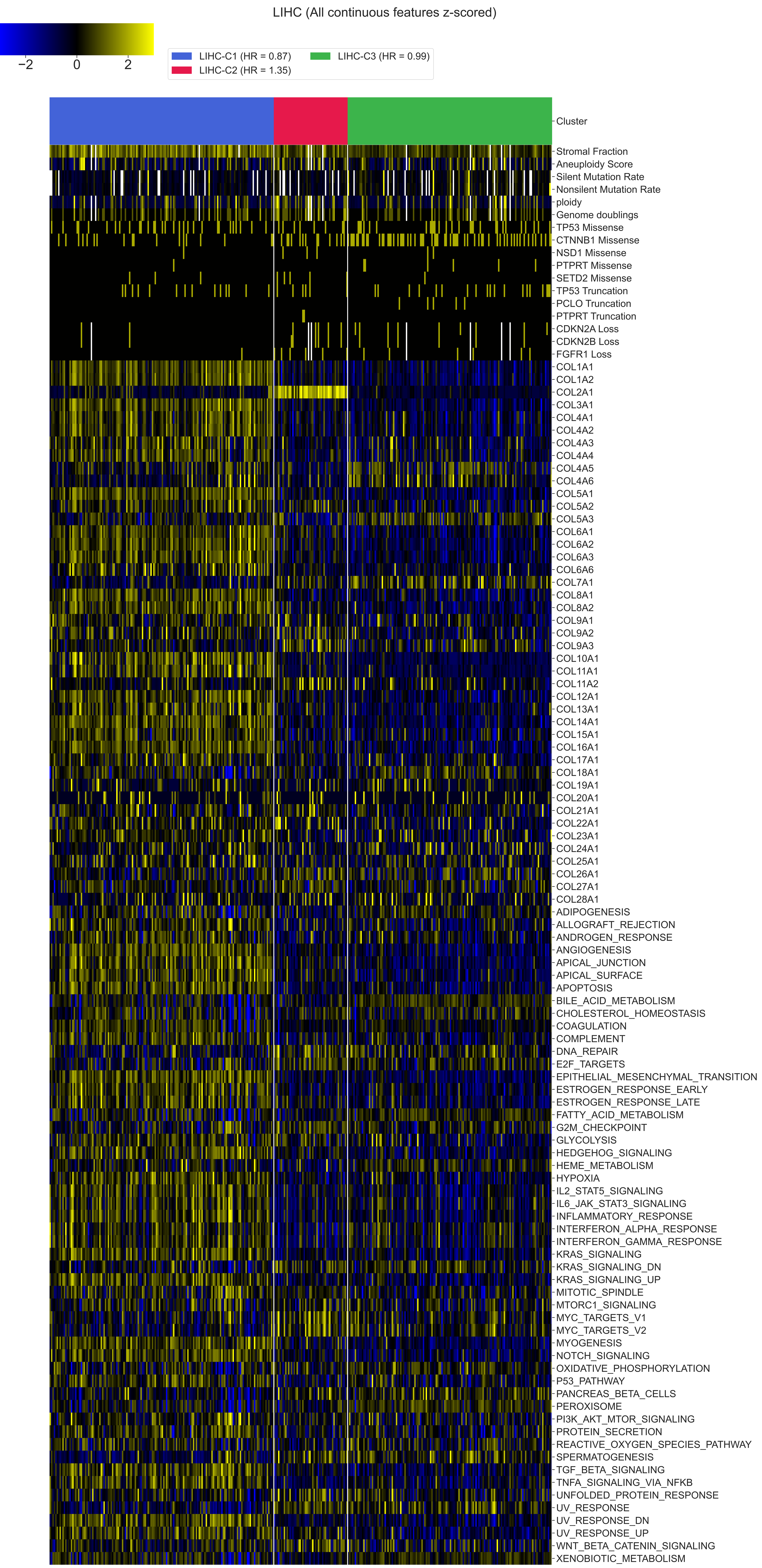

Supplemental Figure S14

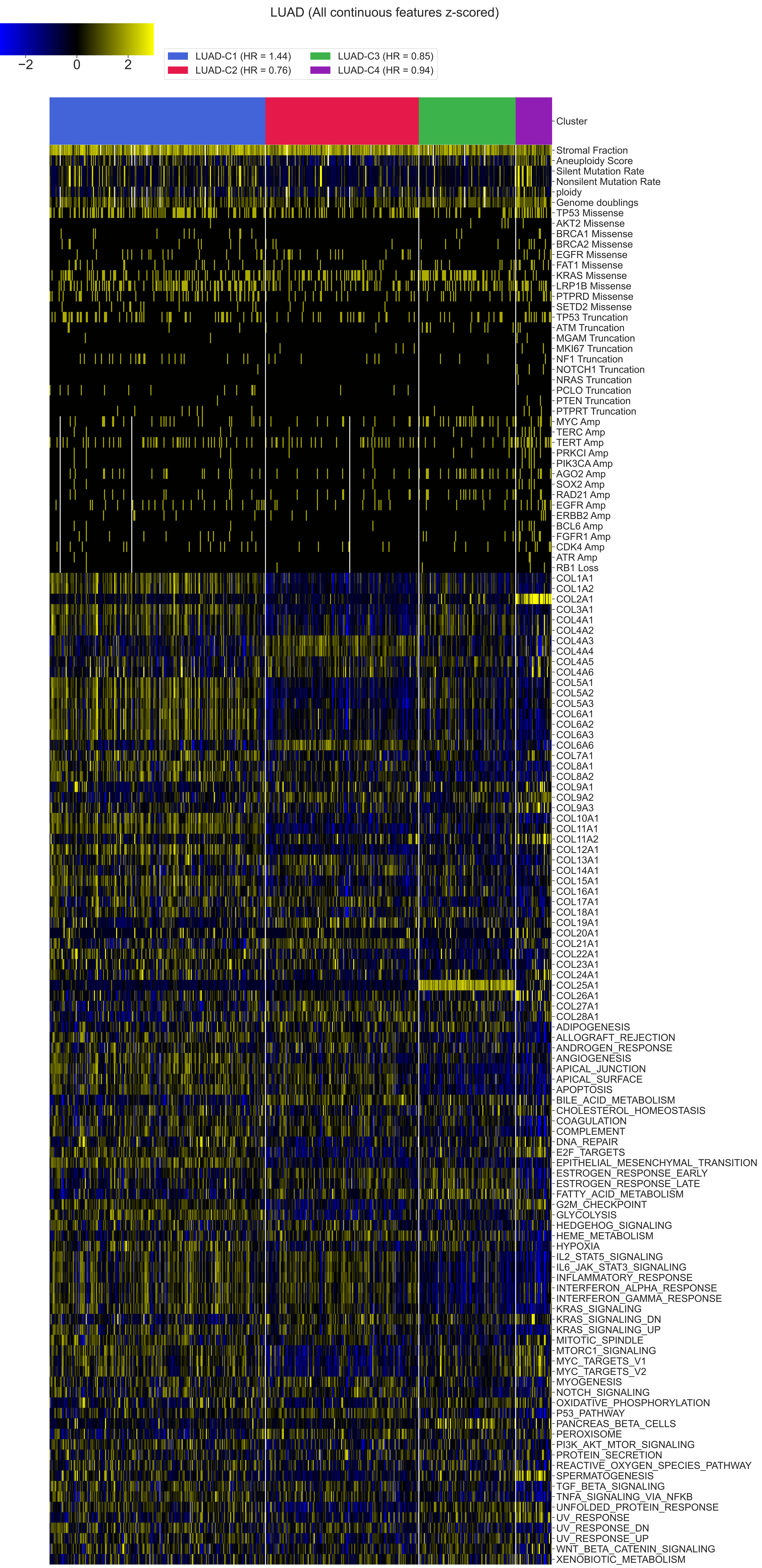

Supplemental Figure S14

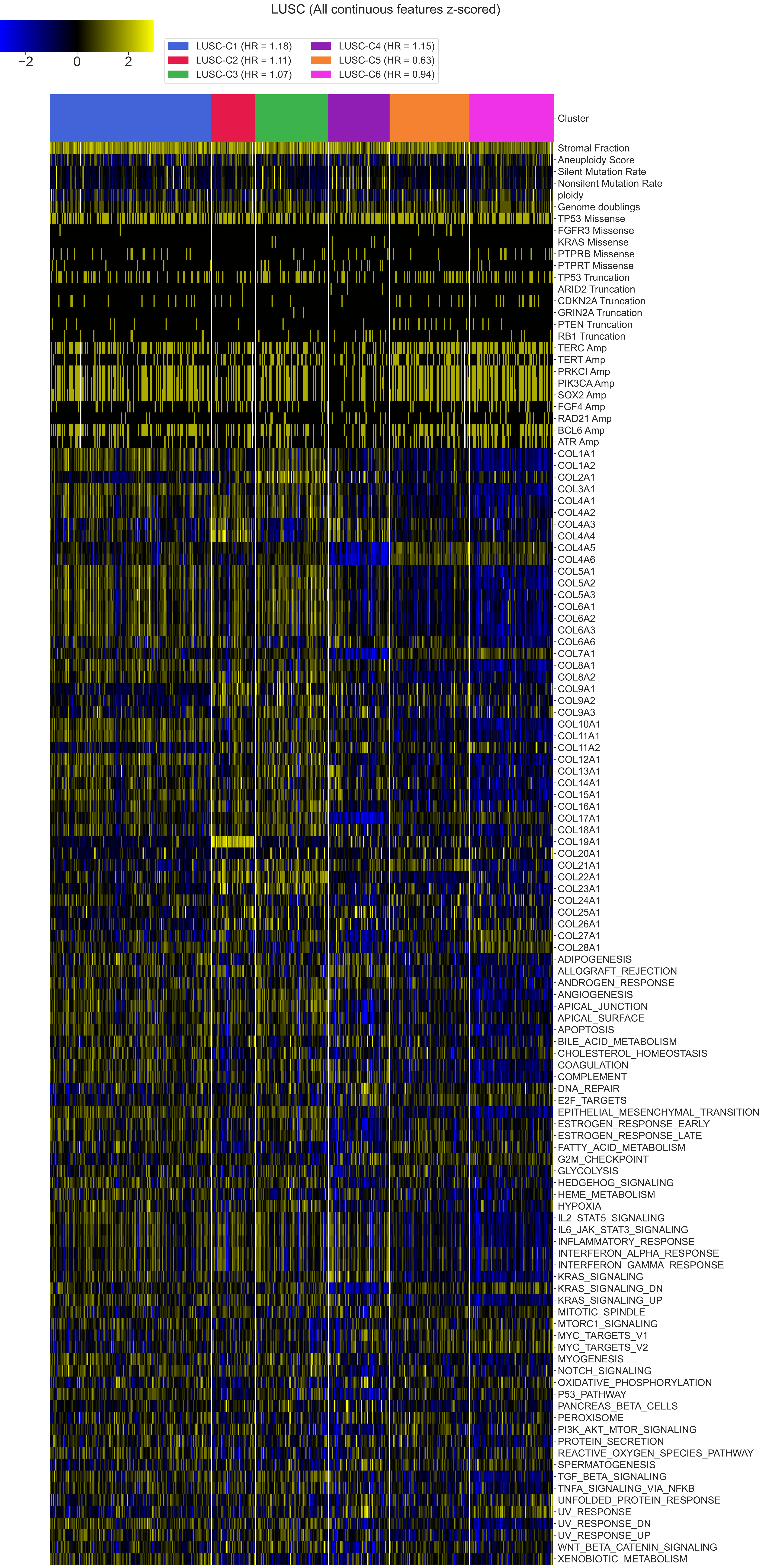

Supplemental Figure S14

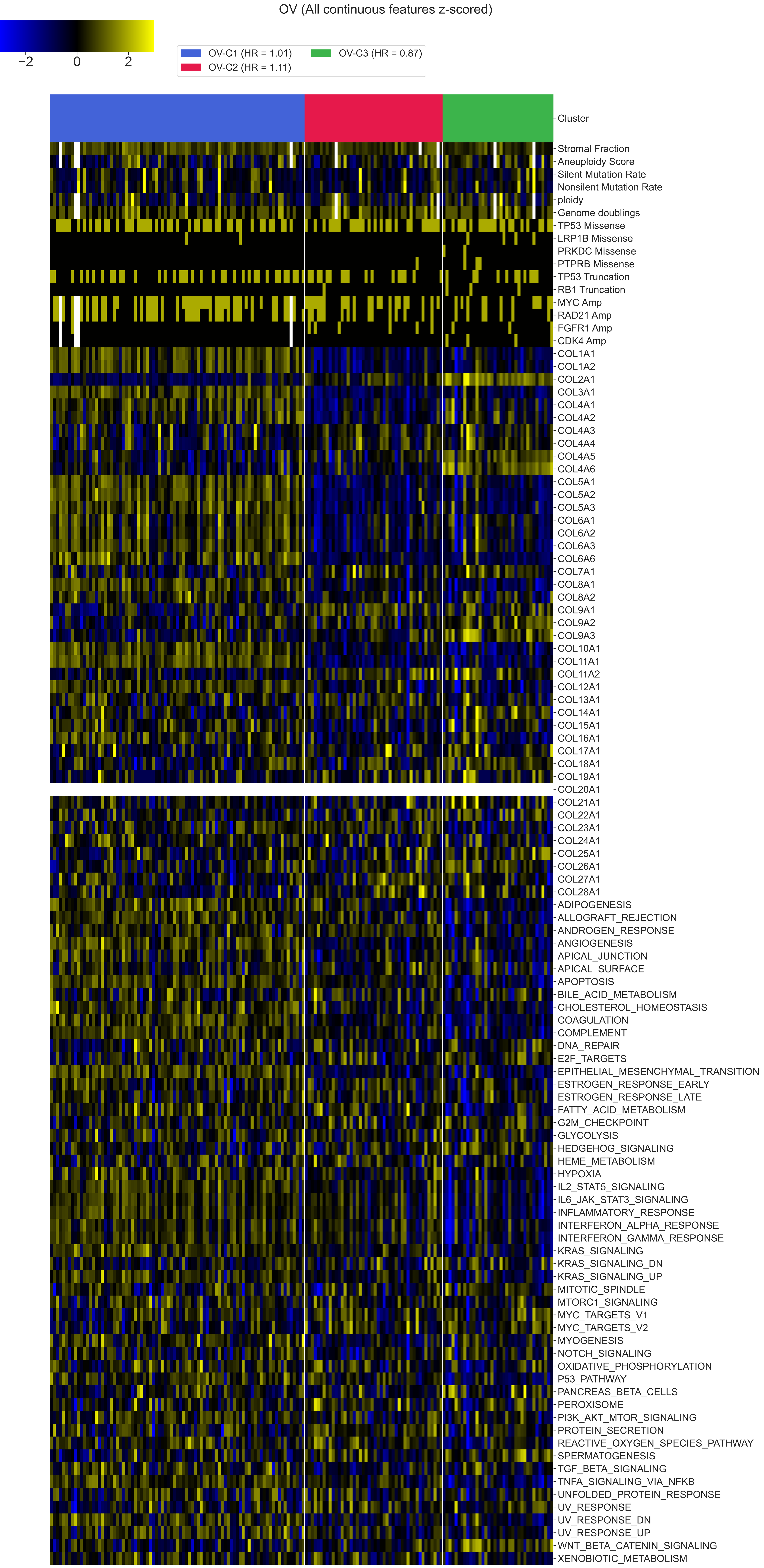

Supplemental Figure S14

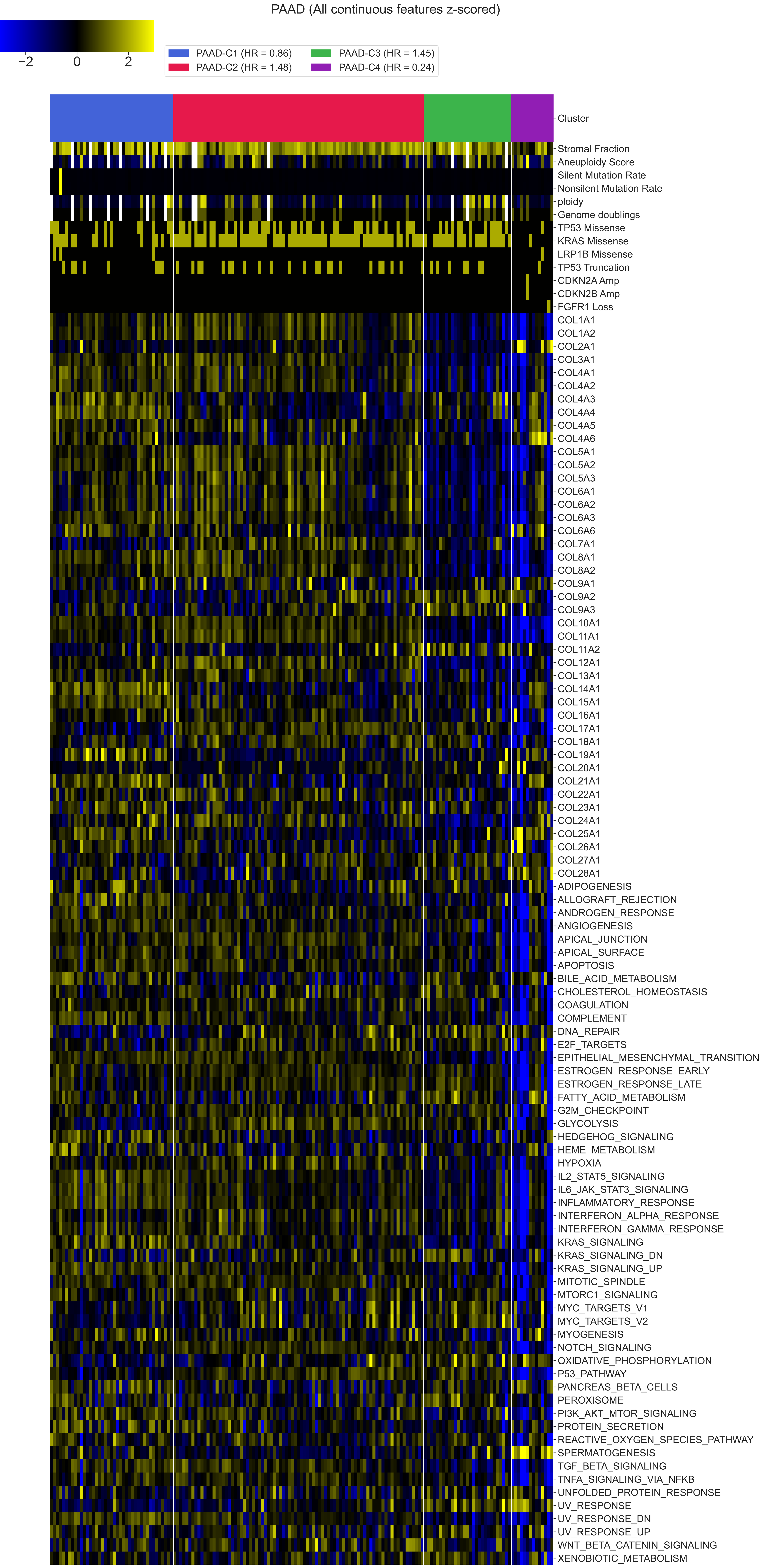

Supplemental Figure S14

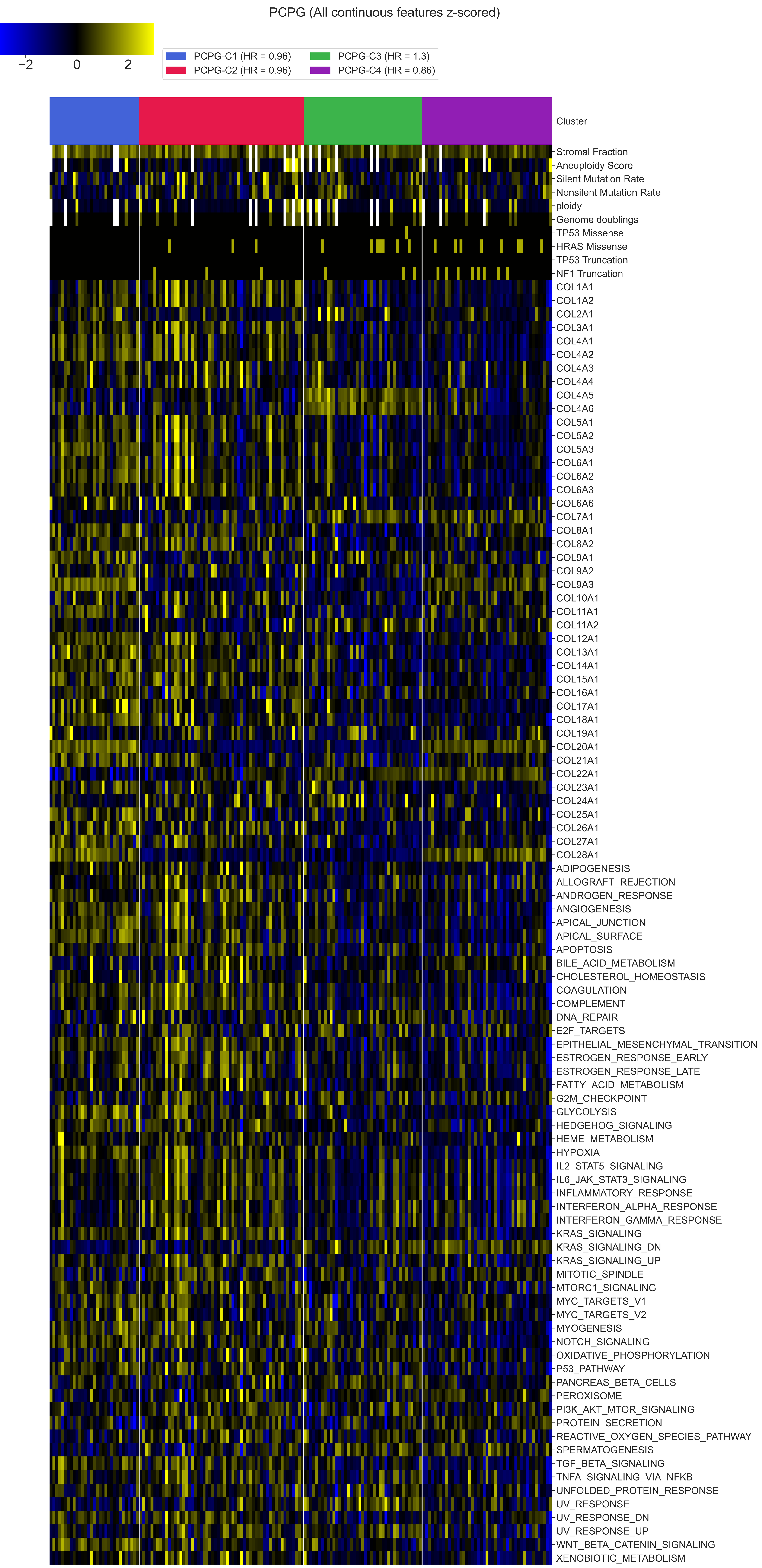

Supplemental Figure S14

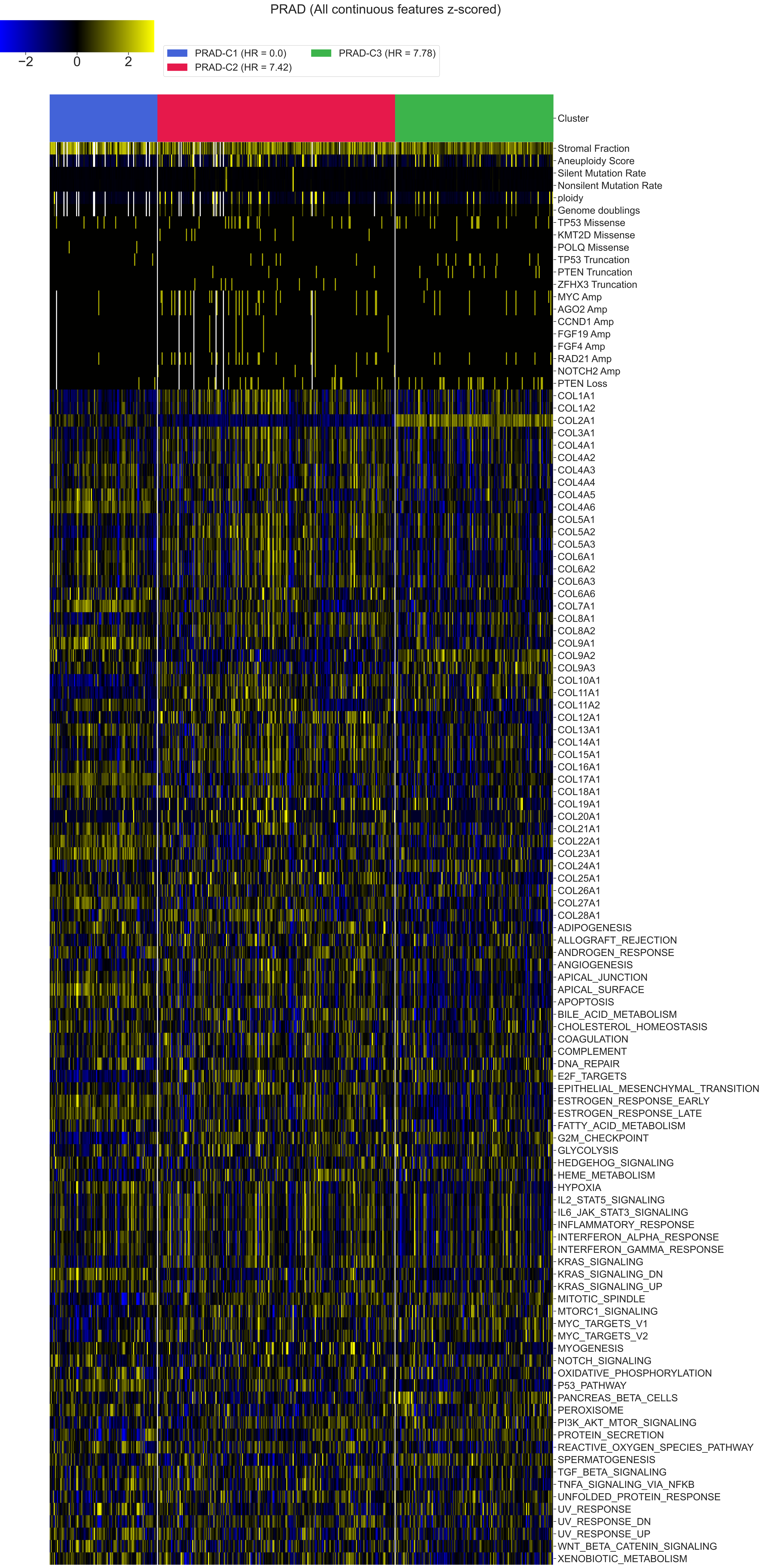

Supplemental Figure S14

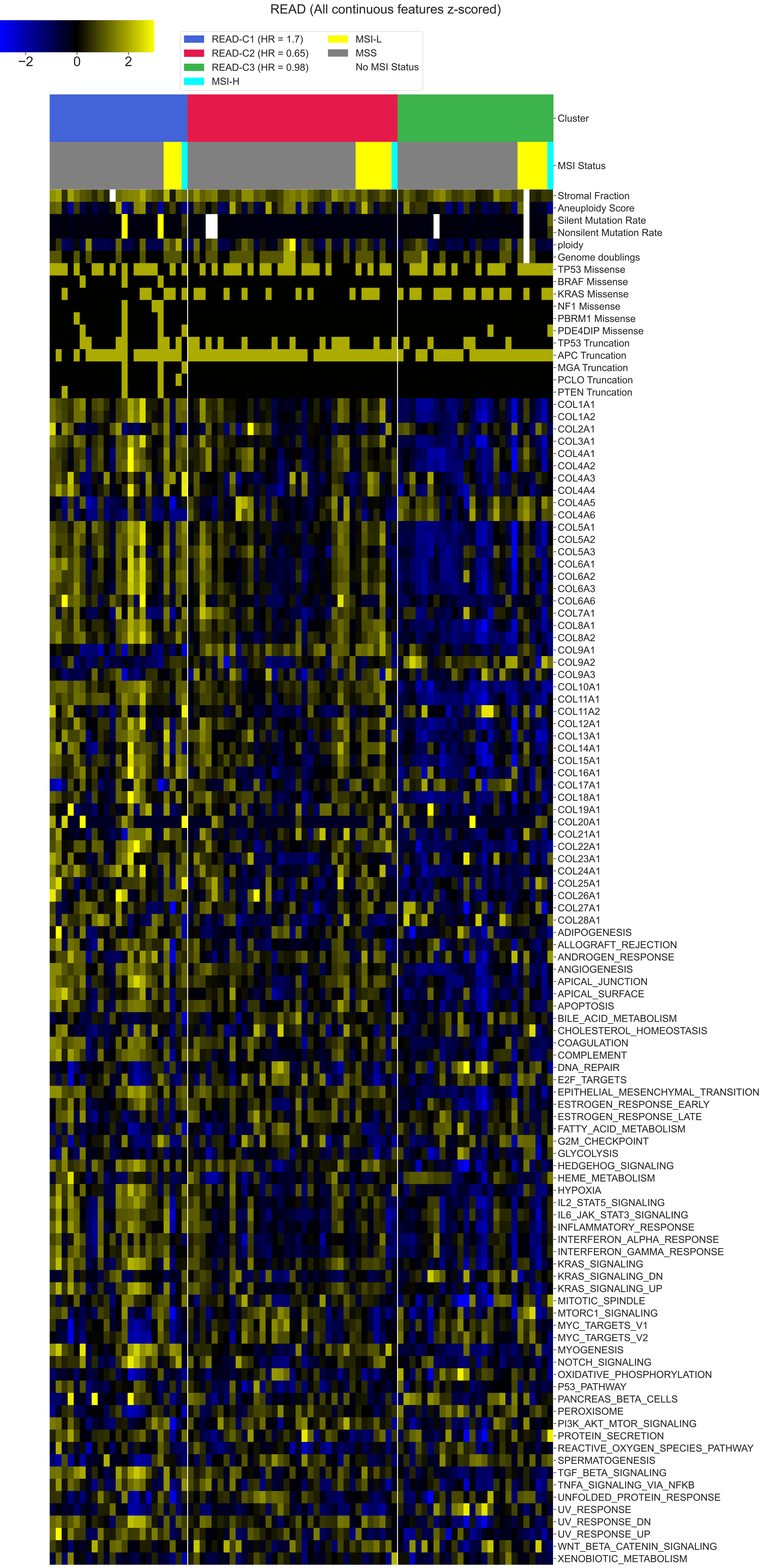

Supplemental Figure S14

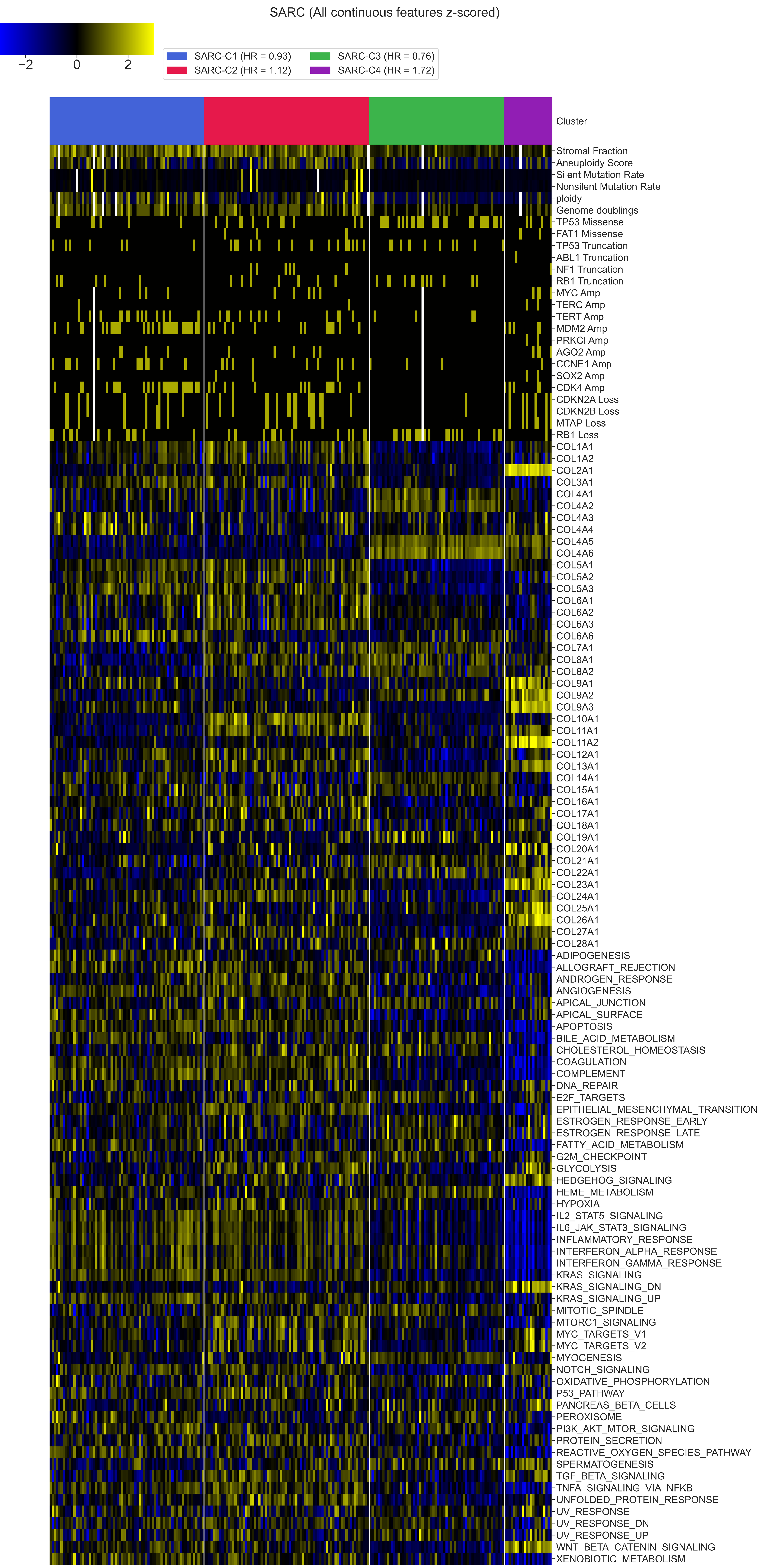

Supplemental Figure S14

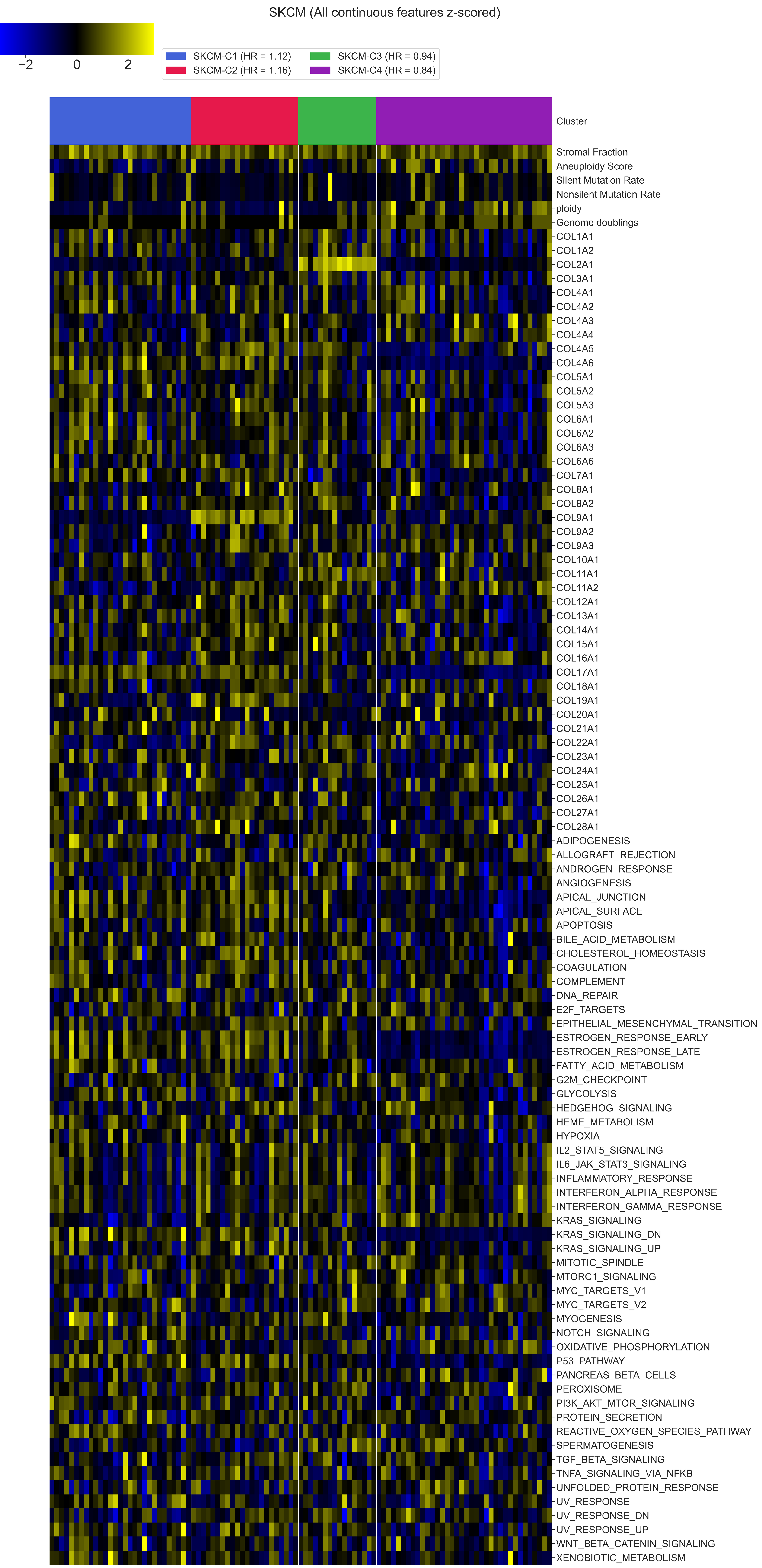

Supplemental Figure S14

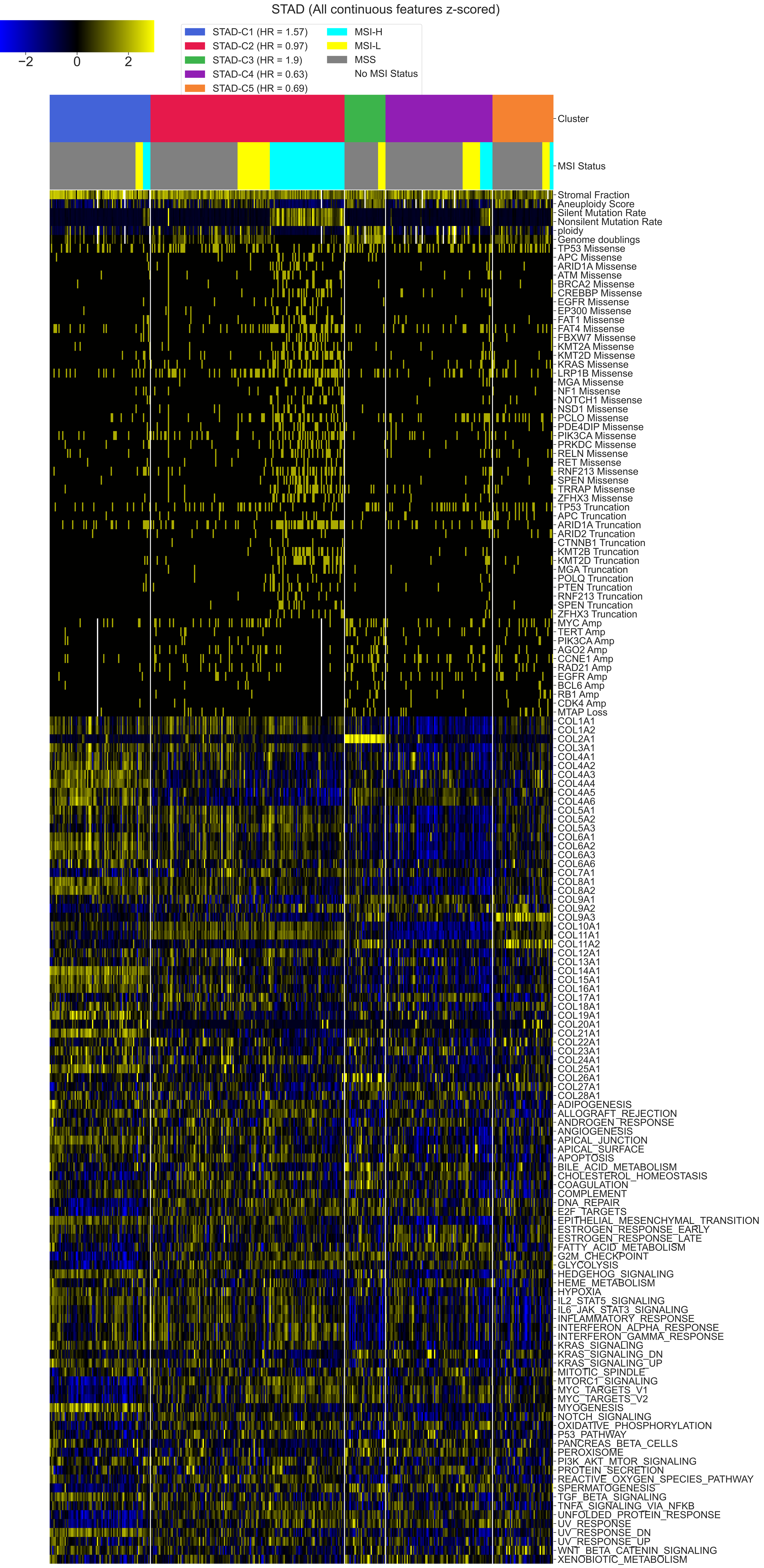

Supplemental Figure S14

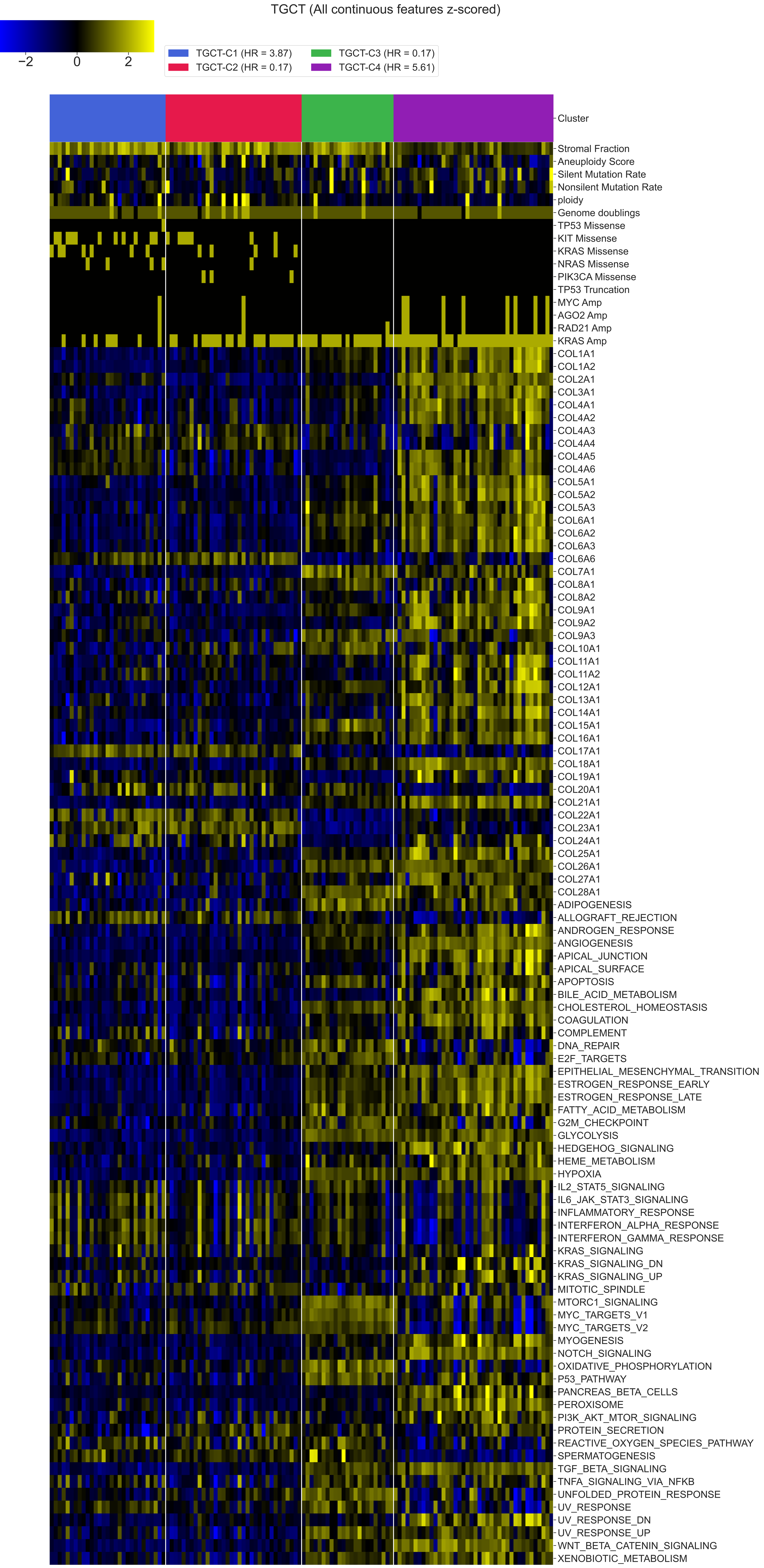

Supplemental Figure S14

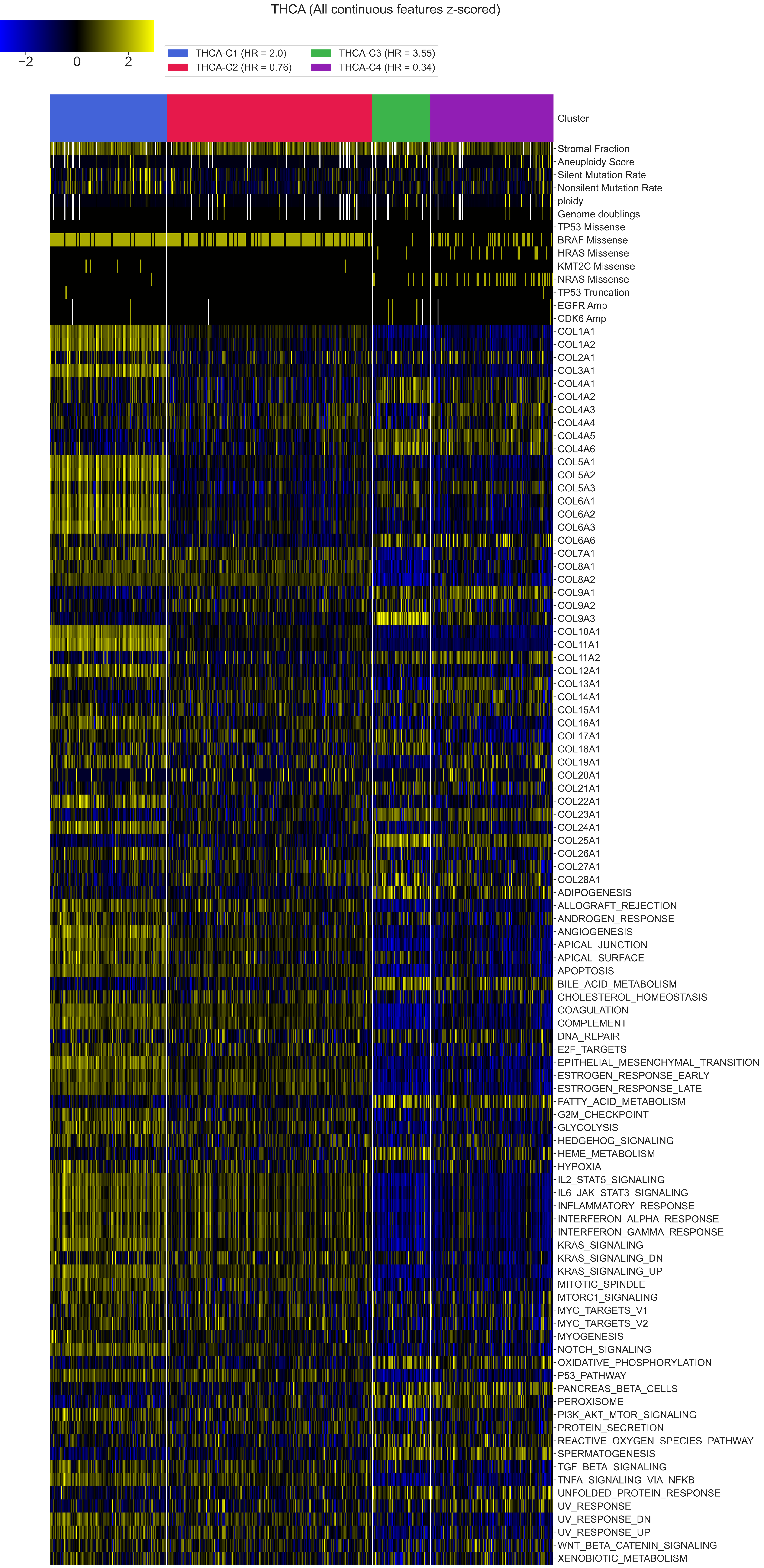

Supplemental Figure S14

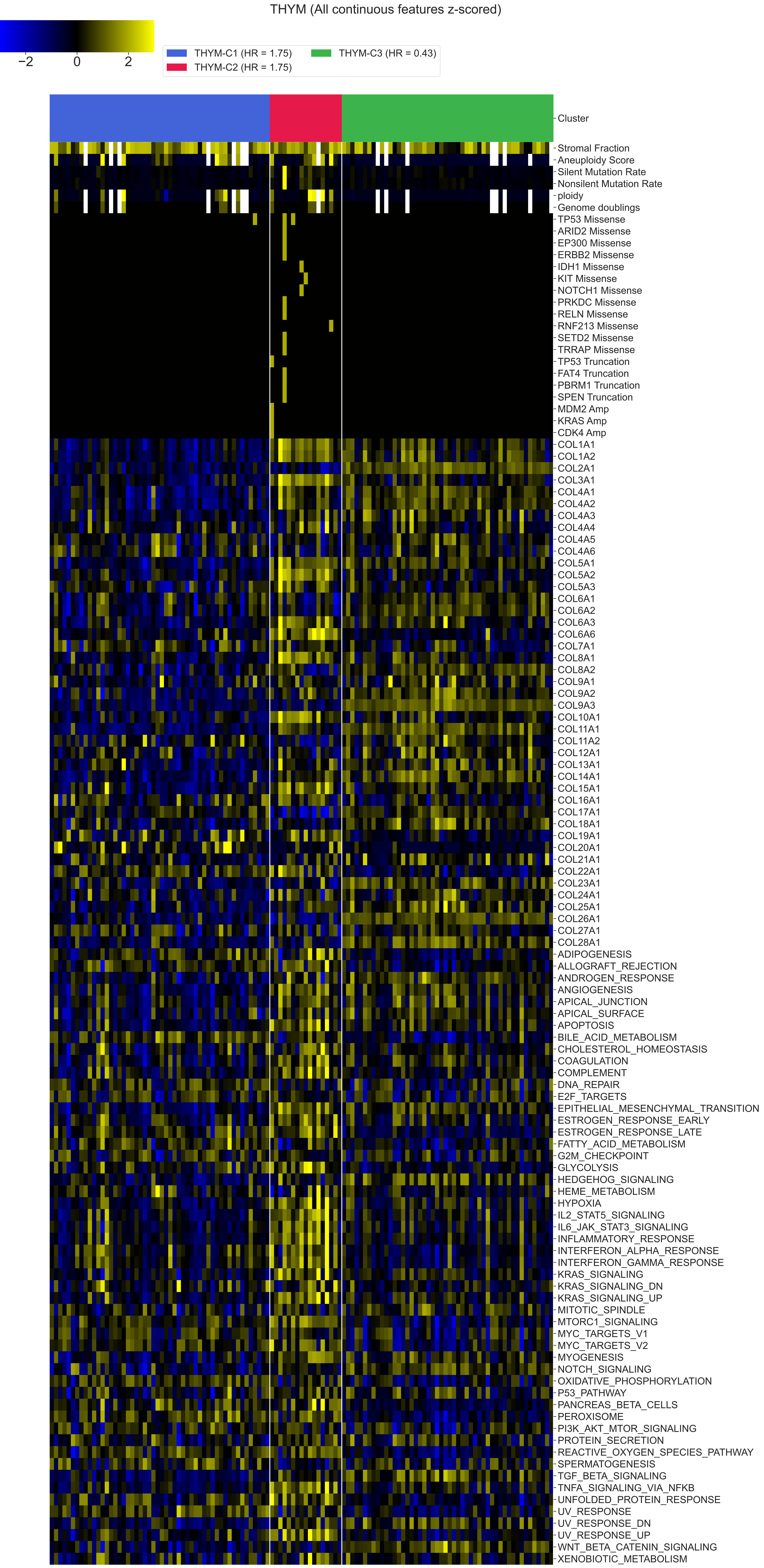

Supplemental Figure S14

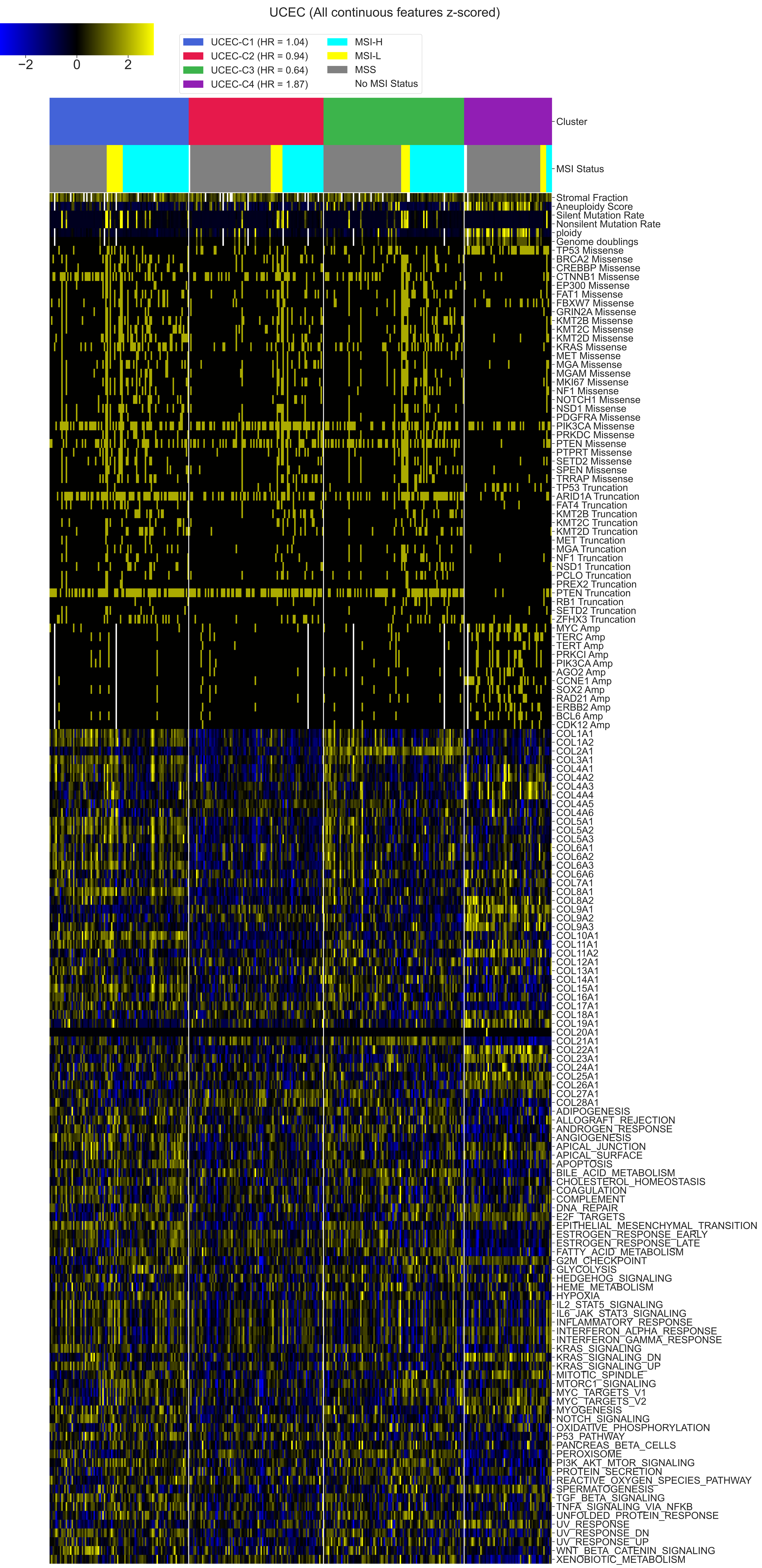

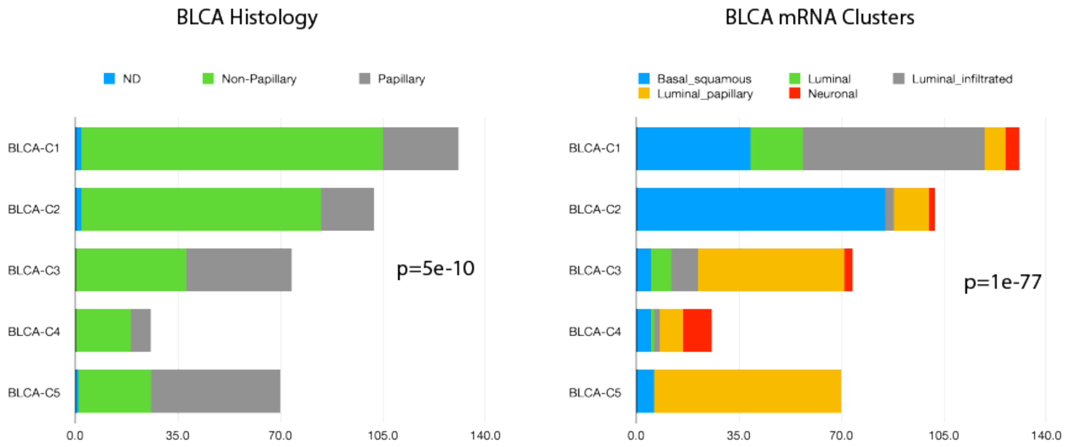

Supplemental Figure 15. Enrichment of histology and mRNA clusters in BLCA. P-values determined by chi-squared test.

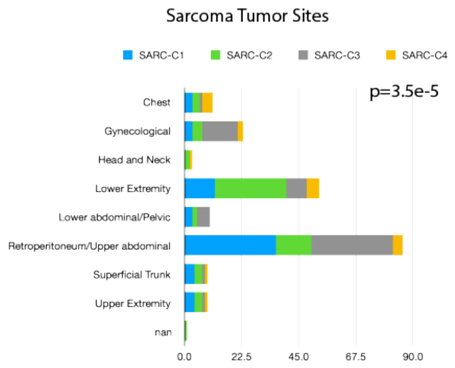

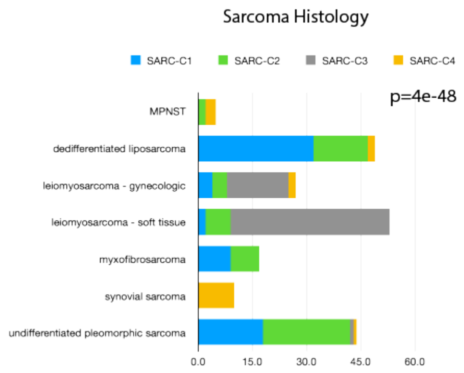

Supplemental Figure 16. Enrichment of tumor sites and histology in sarcomas. P-values determined by chi-squared test.

figures/Supp_circles.png

Supplemental Figure 17. Circle diagrams of molecular alterations and collagen expression in each ColCluster for each cancer type. ColClusters with relatively high and low general collagen expression are shown. See file for Figure S15.

Supplemental Figure S17

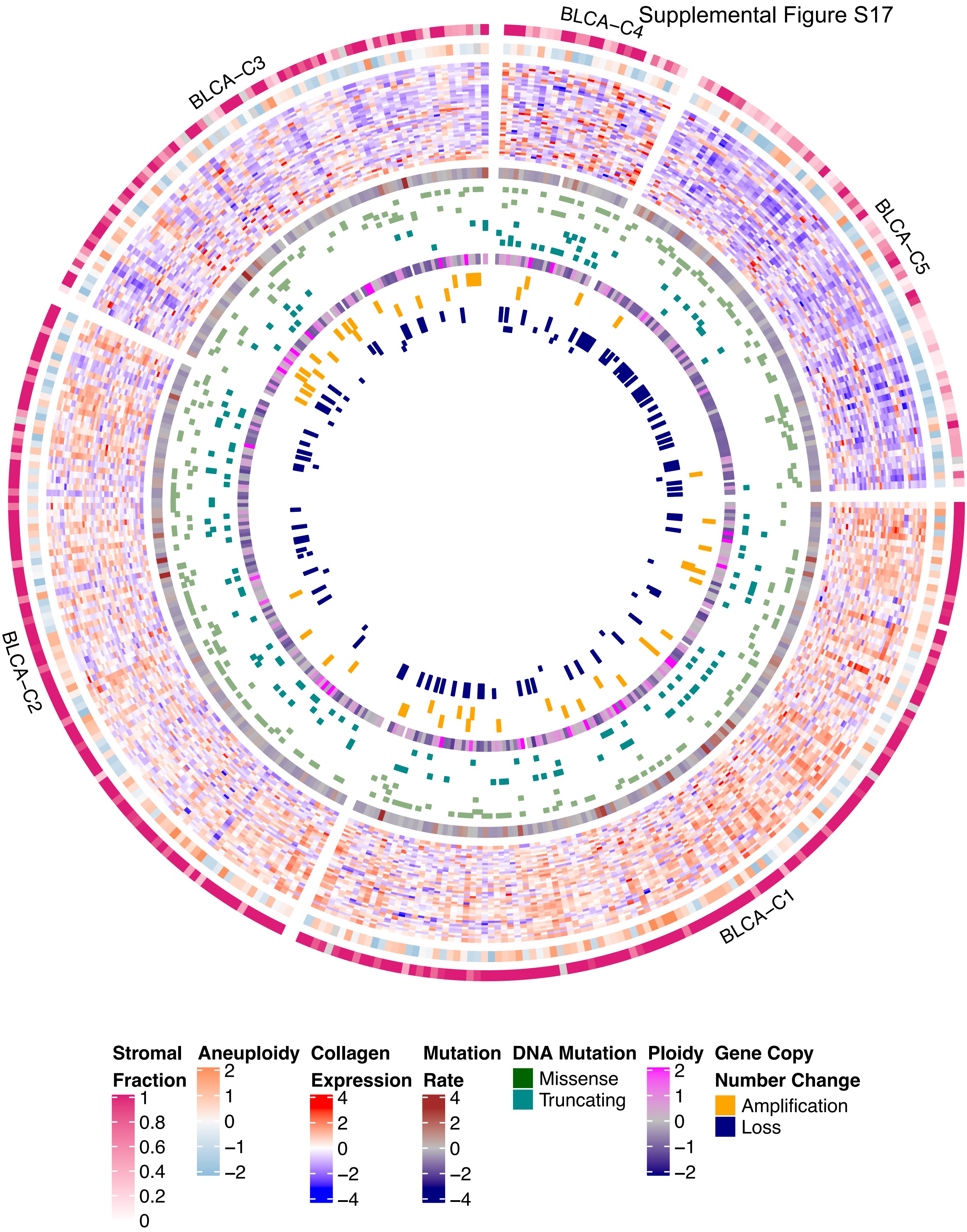

Supplemental Figure S17

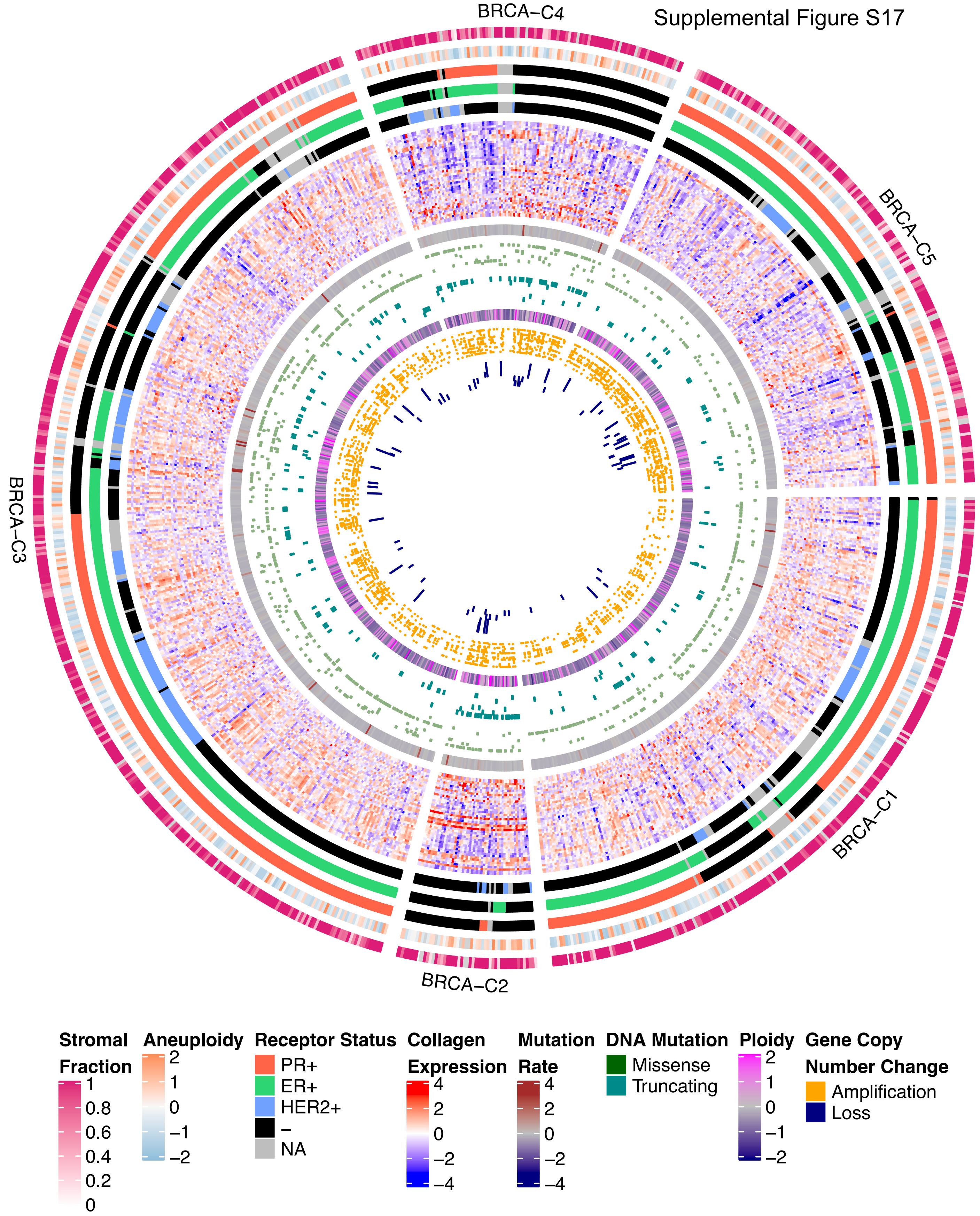

Supplemental Figure S17

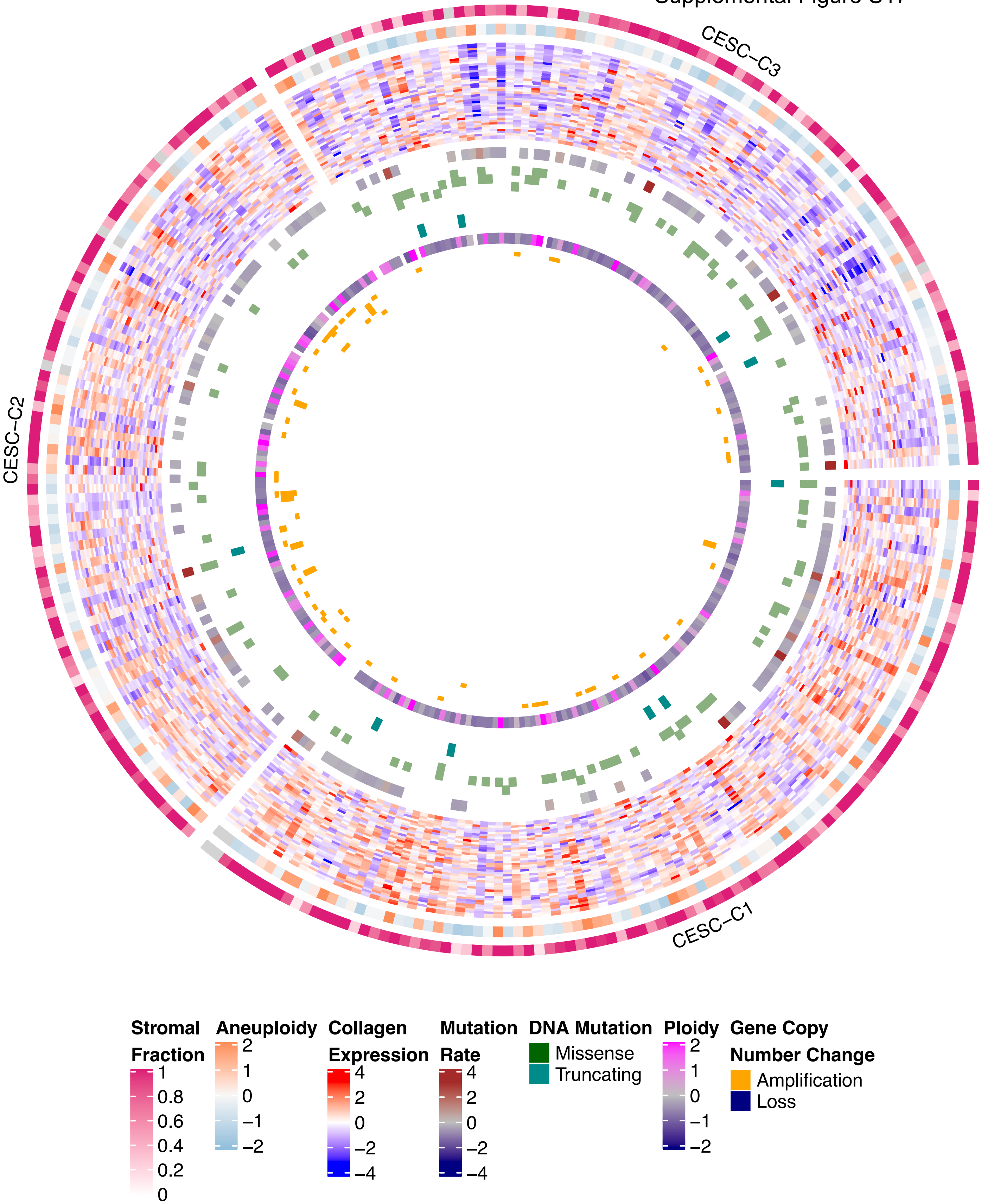

Supplemental Figure S17

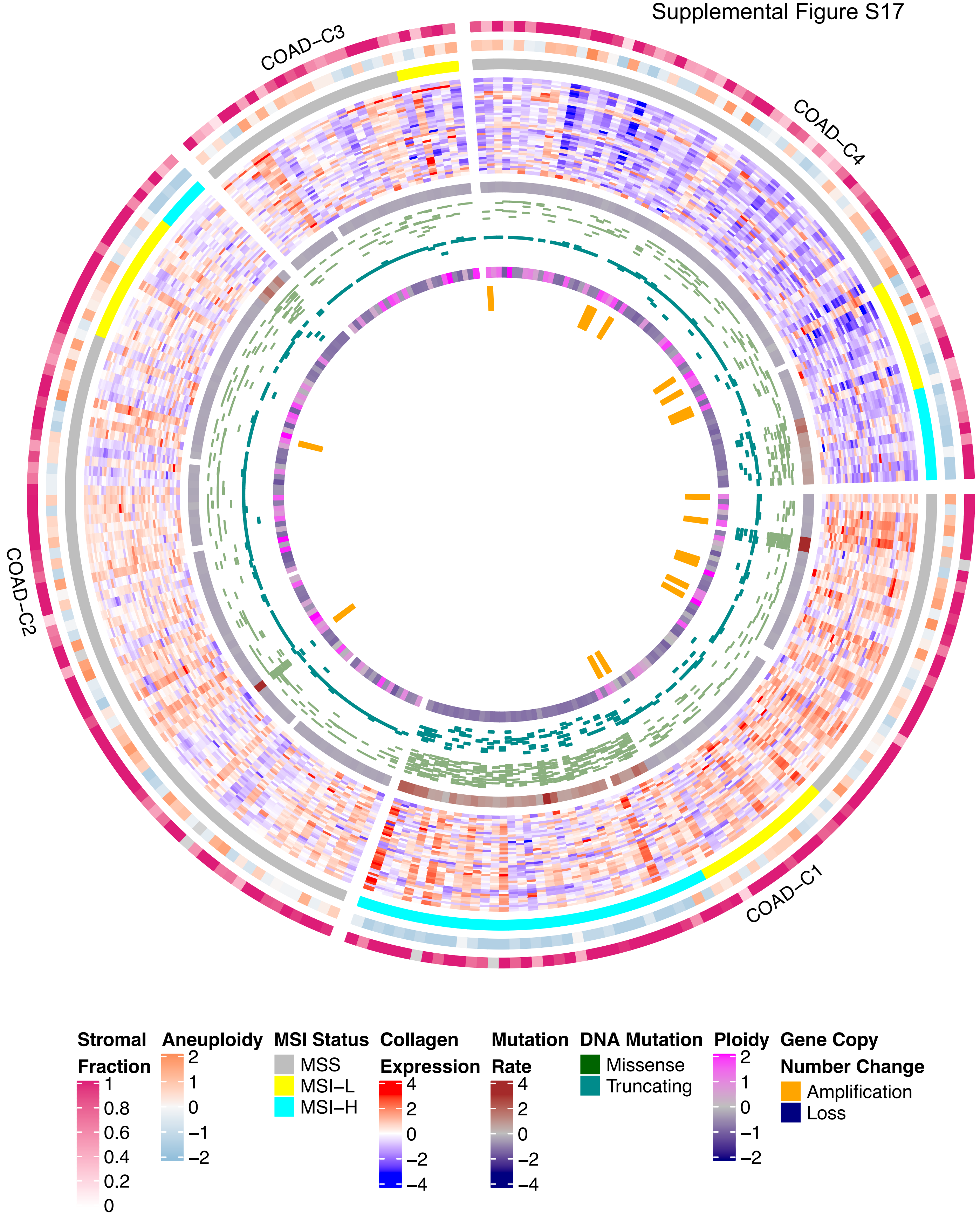

Supplemental Figure S17

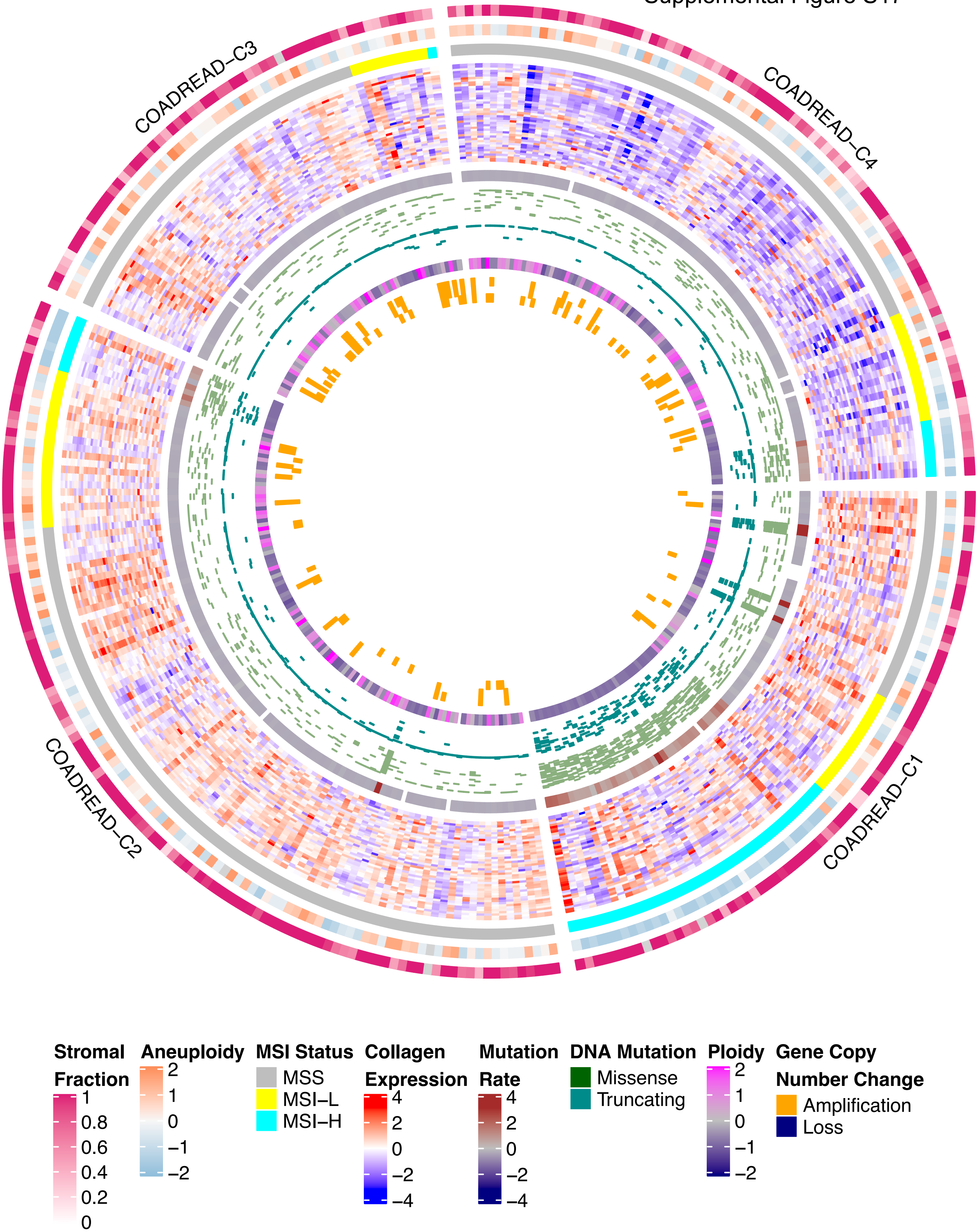

Supplemental Figure S17

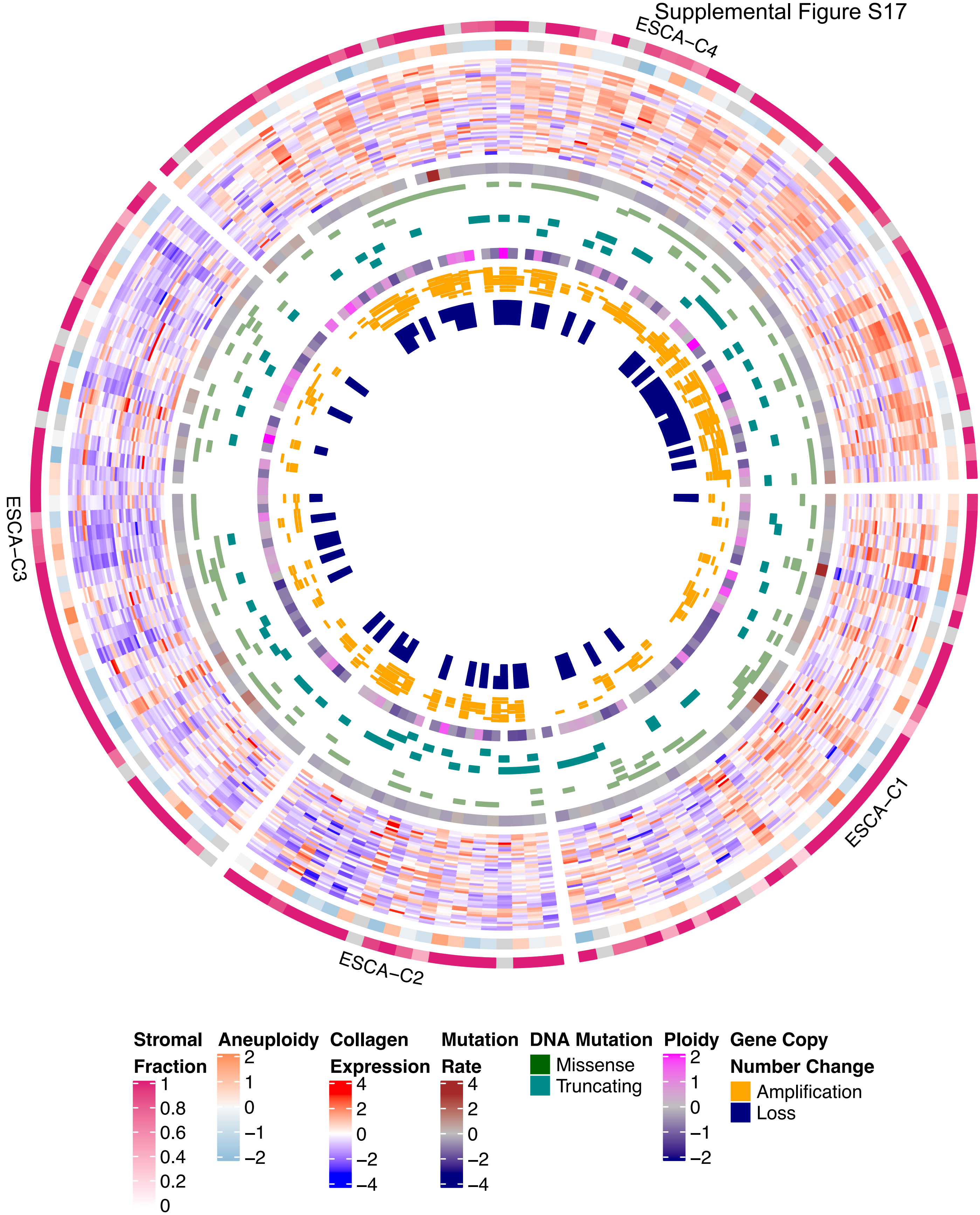

Supplemental Figure S17

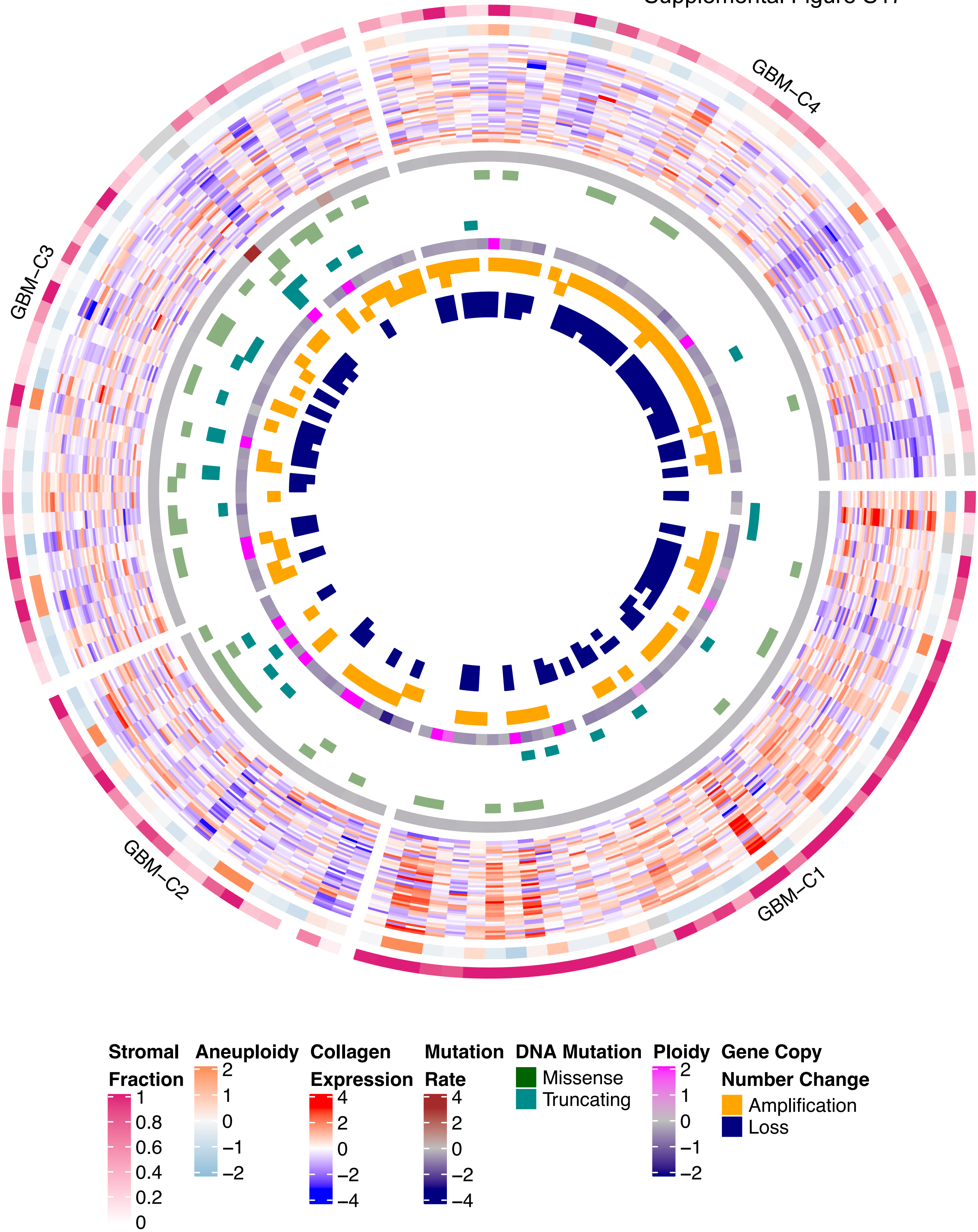

Supplemental Figure S17

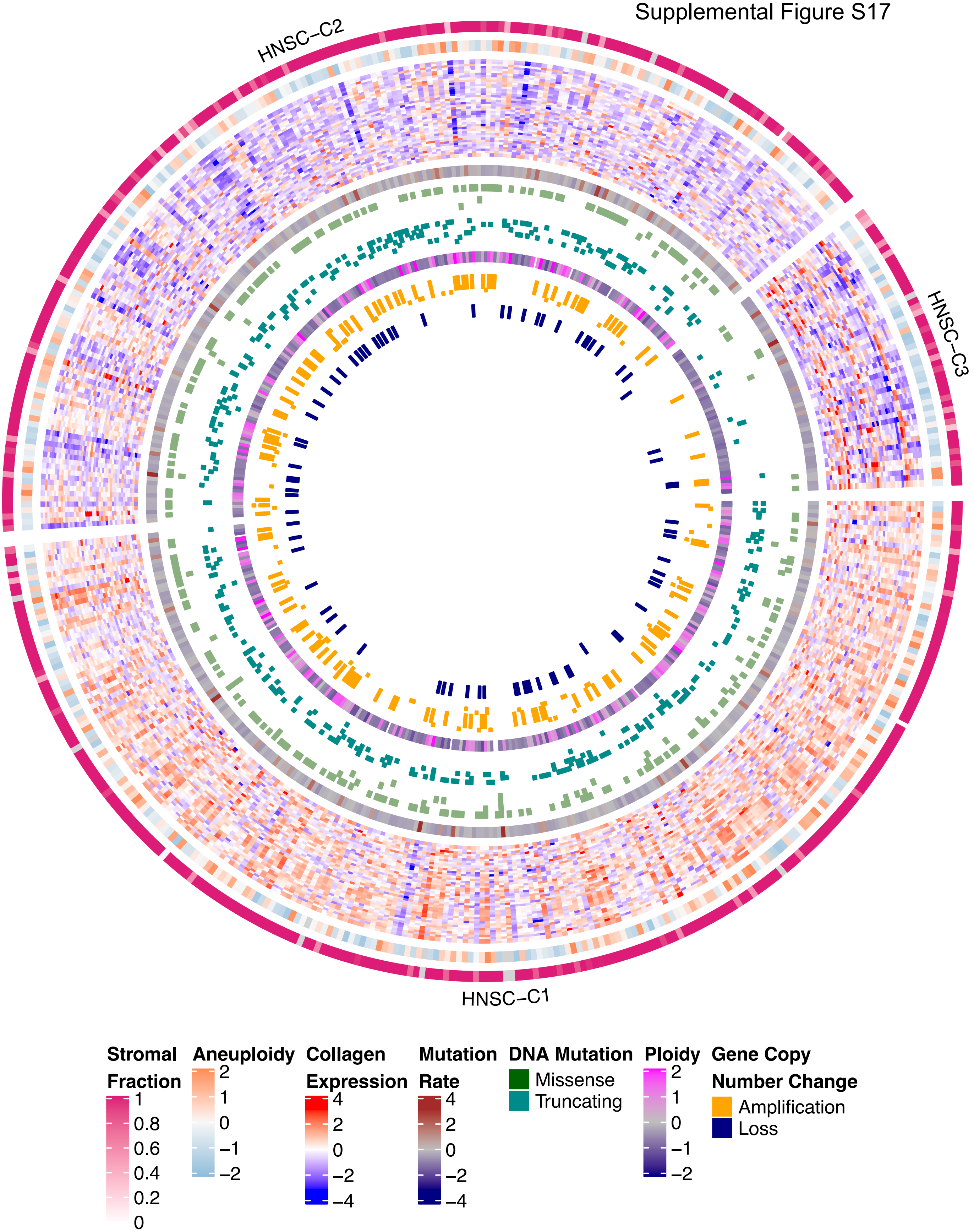

Supplemental Figure S17

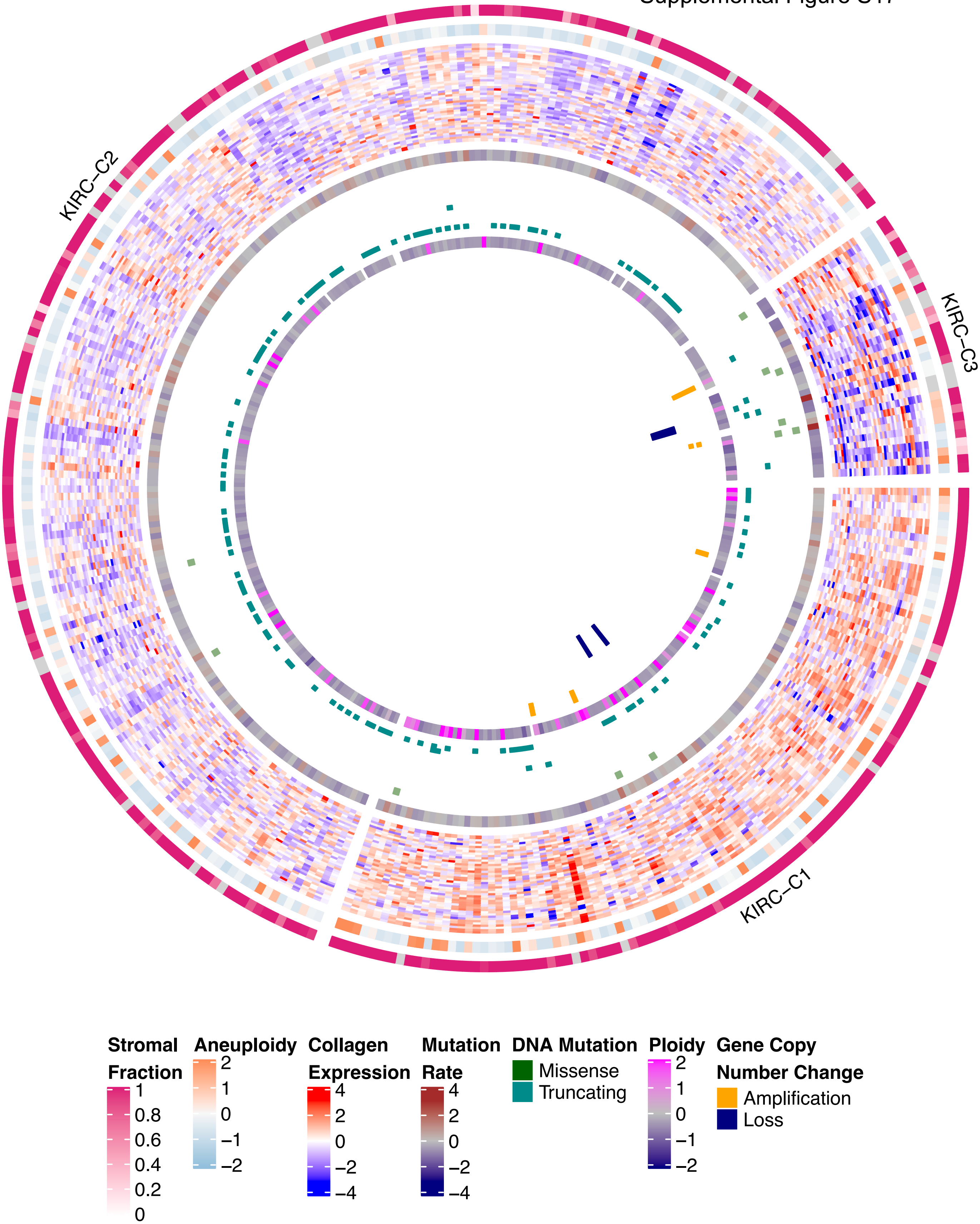

Supplemental Figure S17

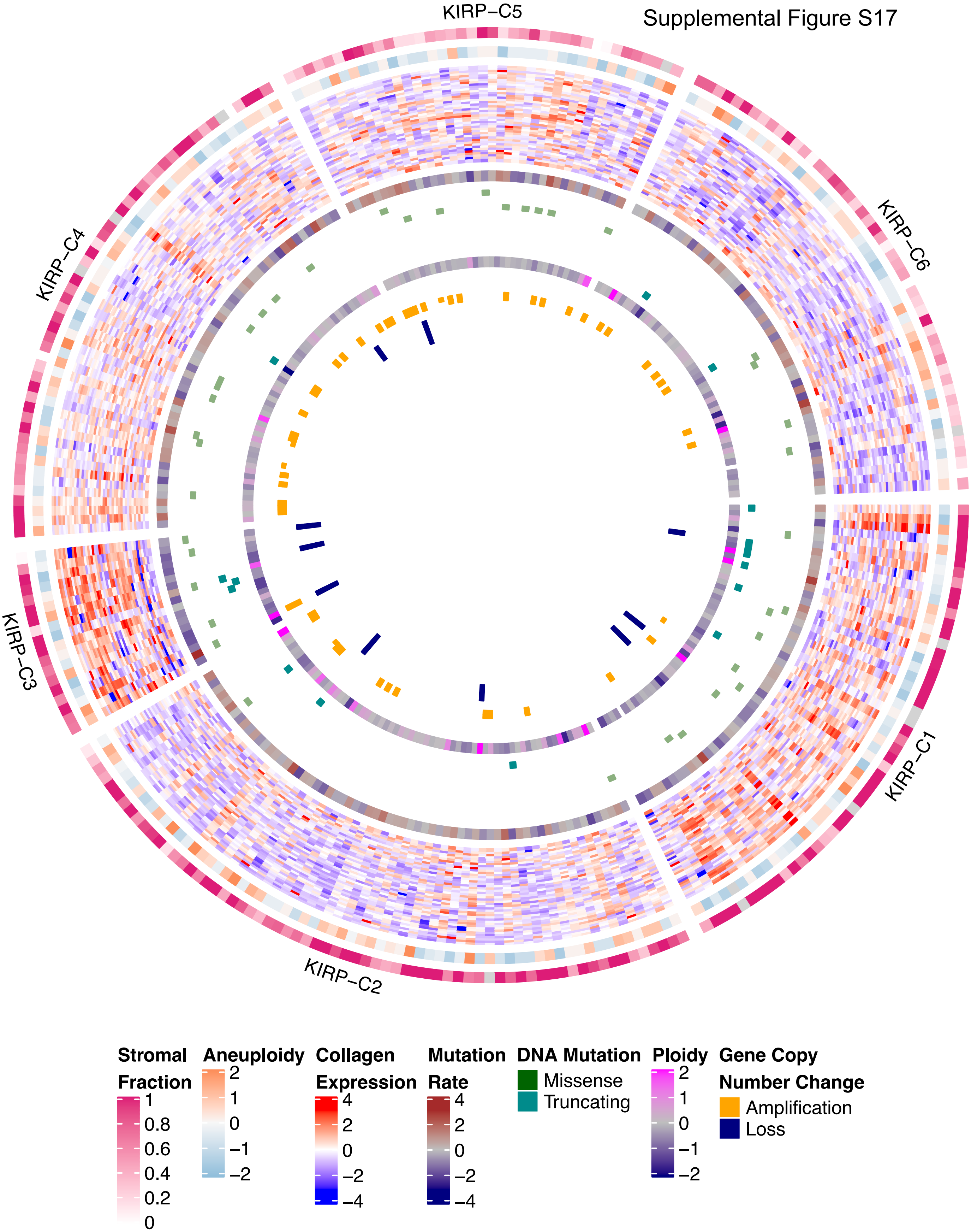

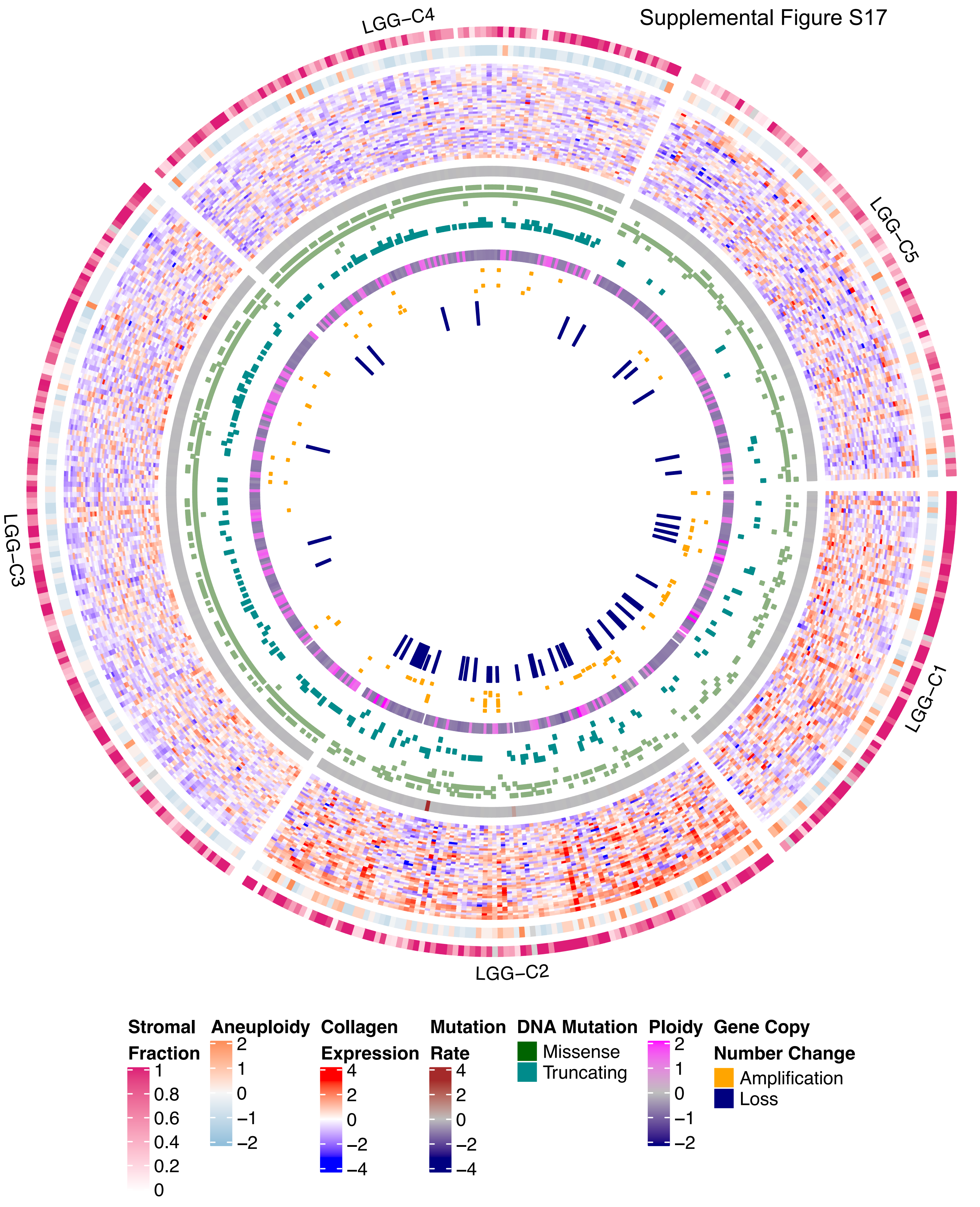

Supplemental Figure S17
LGG−C4
LGG−C5
LGG−C3
LGG−C1
LGG−C2
Stromal Fraction
1
0.8
0.6
0.4
0.2
0
Aneuploidy
2
1
0
−1
−2
Collagen Expression
4
2
0
−2
−4
Mutation Rate
4
2
0
−2
−4
DNA Mutation
Missense
Truncating
Ploidy
2
1
0
−1
−2
Gene Copy Number Change
Amplification
Loss

Supplemental Figure S17

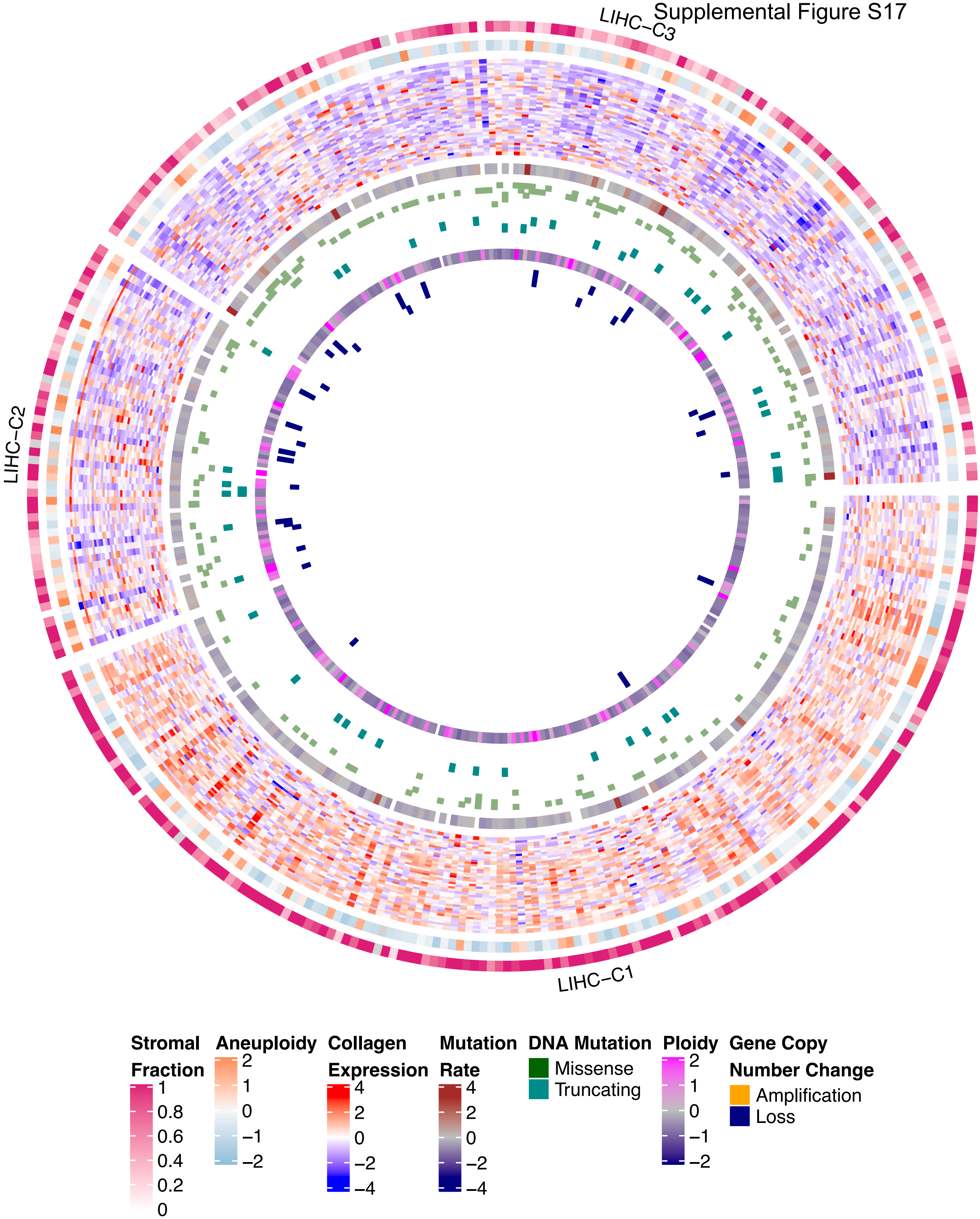

Supplemental Figure S17

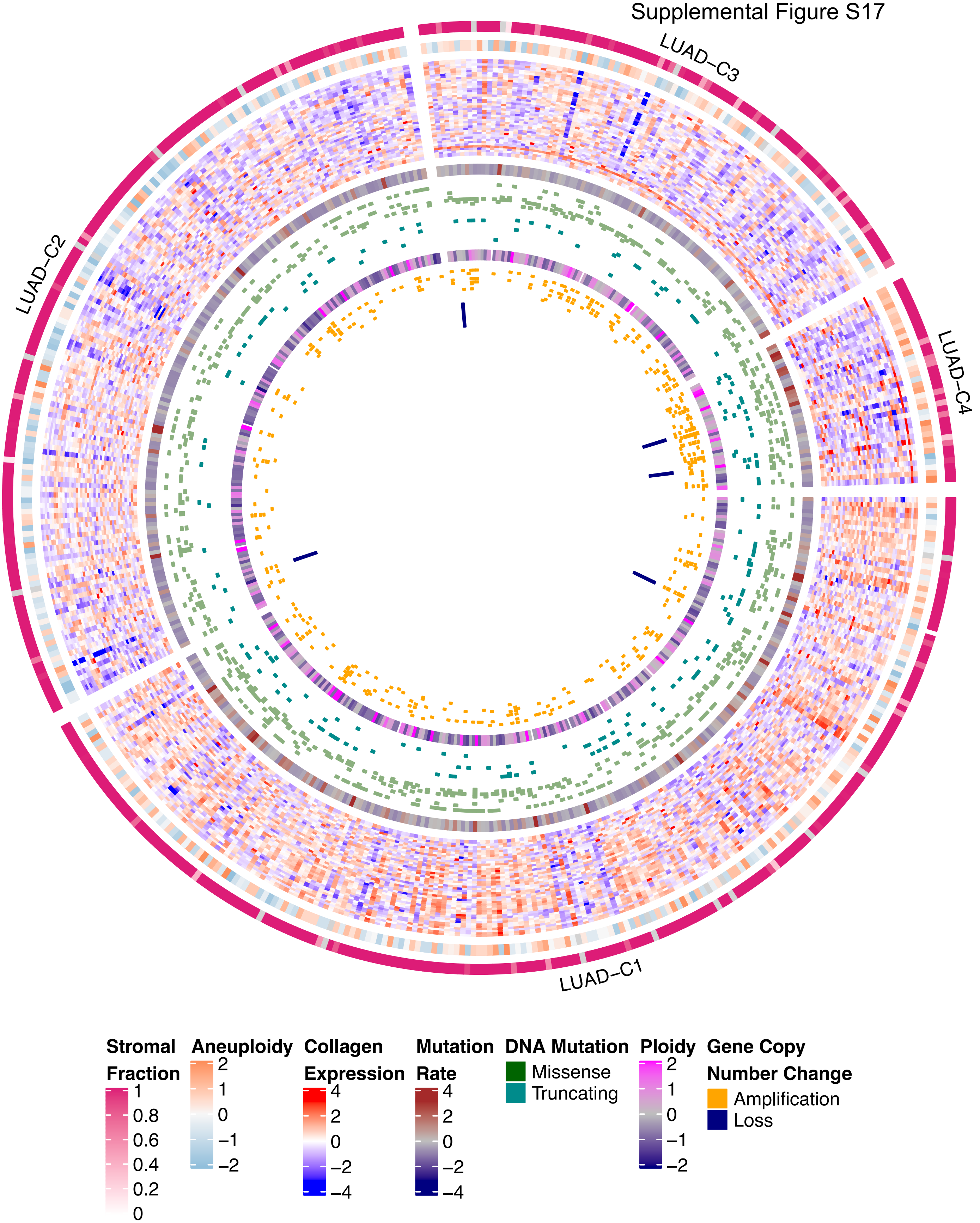

Supplemental Figure S17

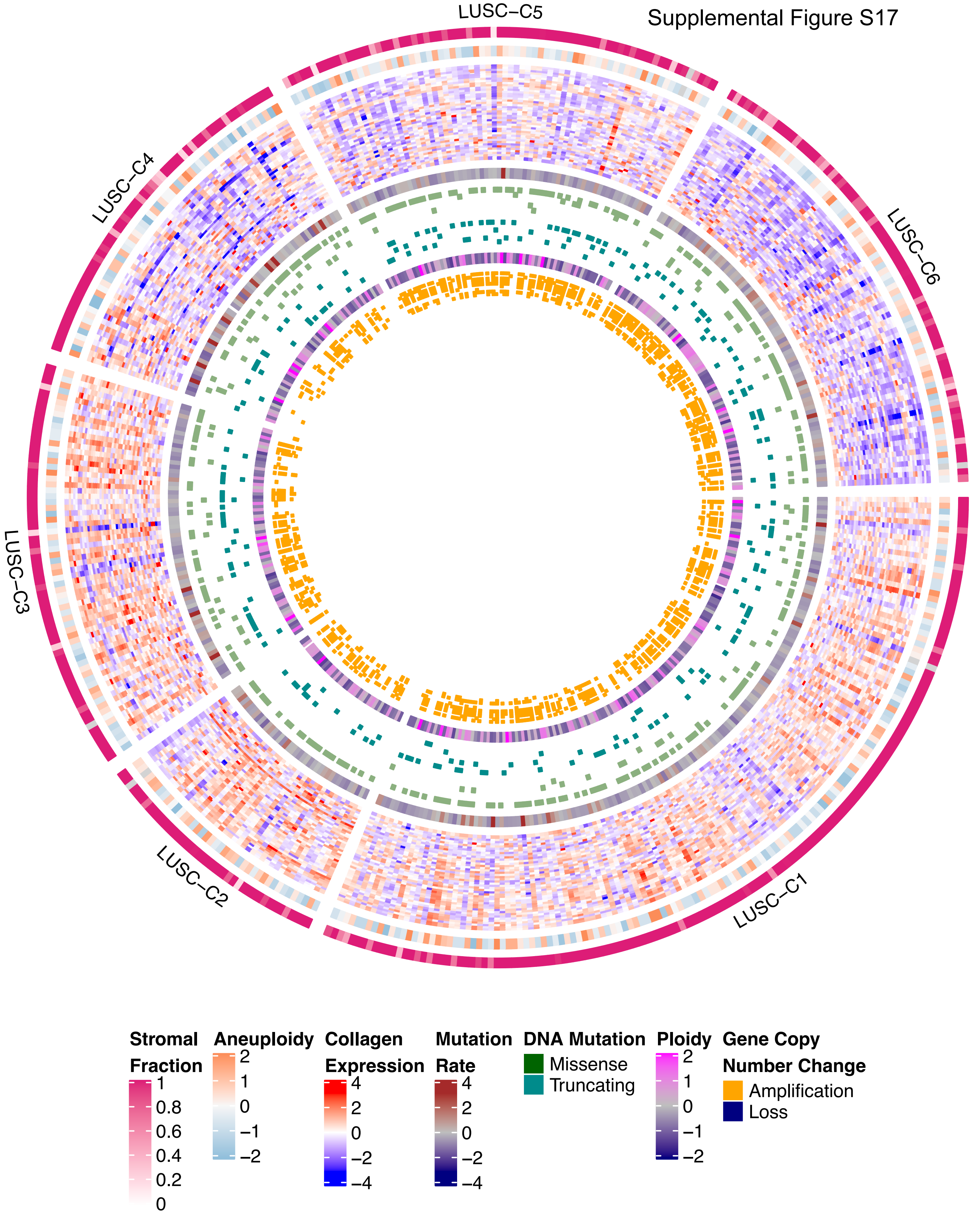

Supplemental Figure S17

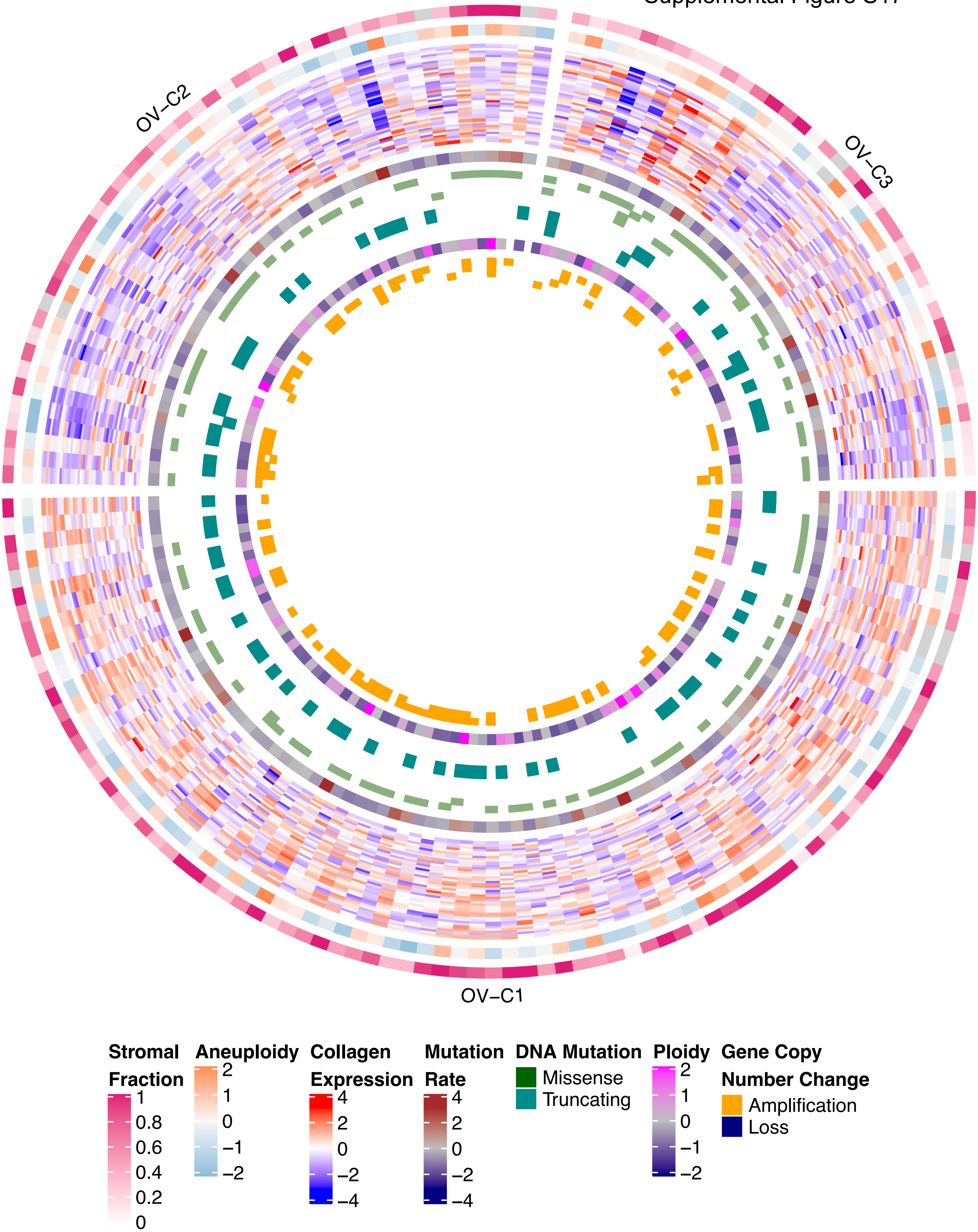

Supplemental Figure S17

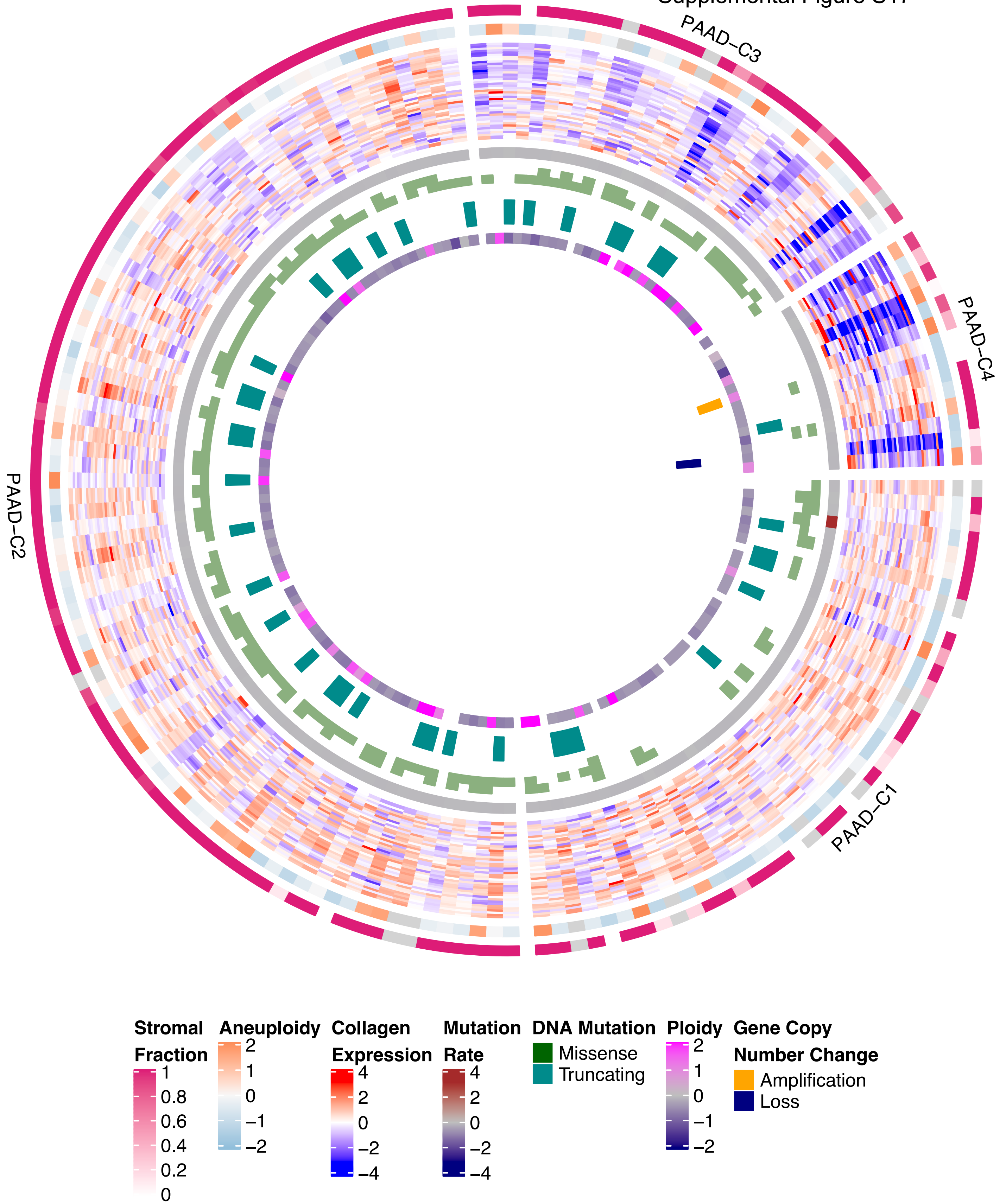

Supplemental Figure S17

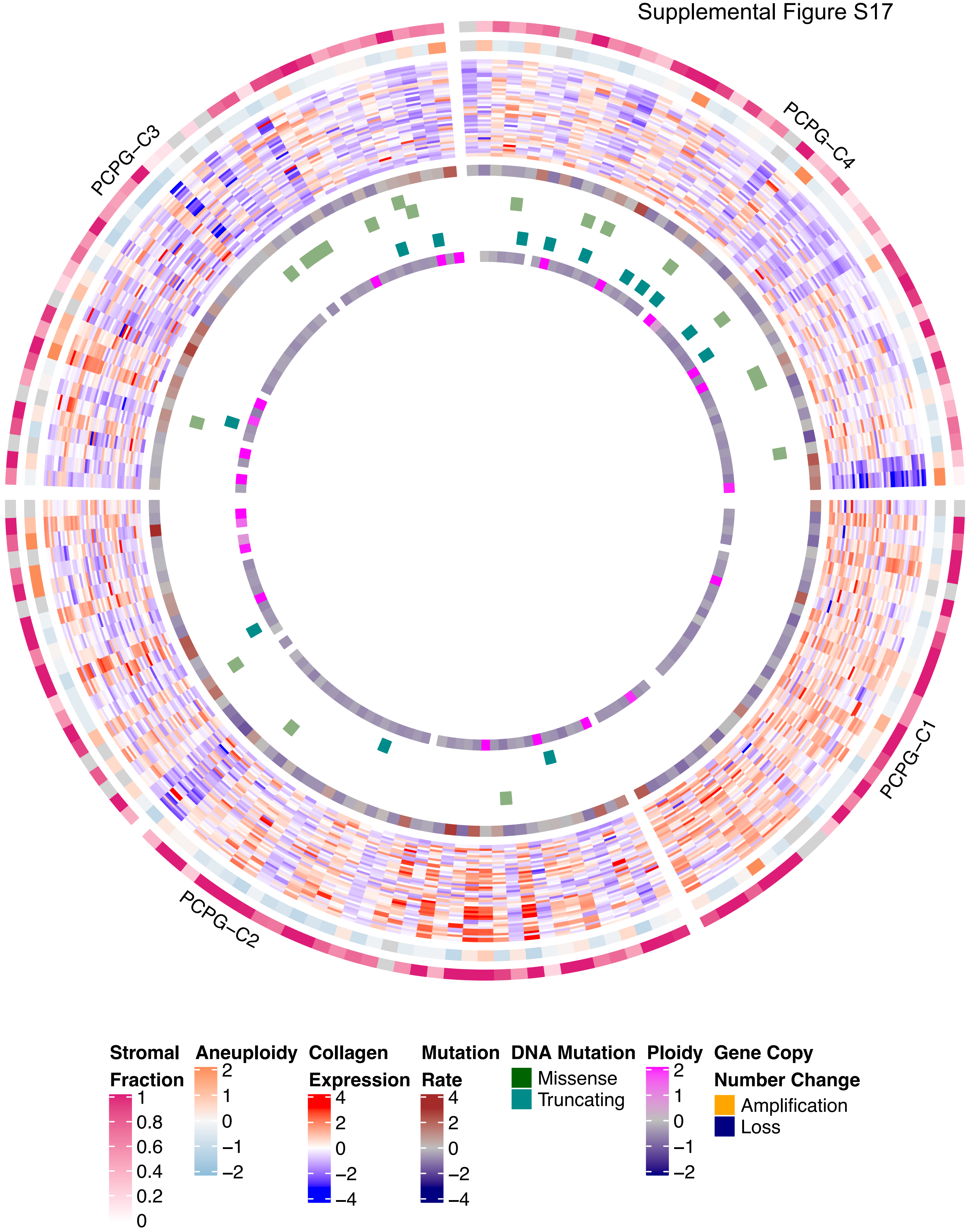

Supplemental Figure S17

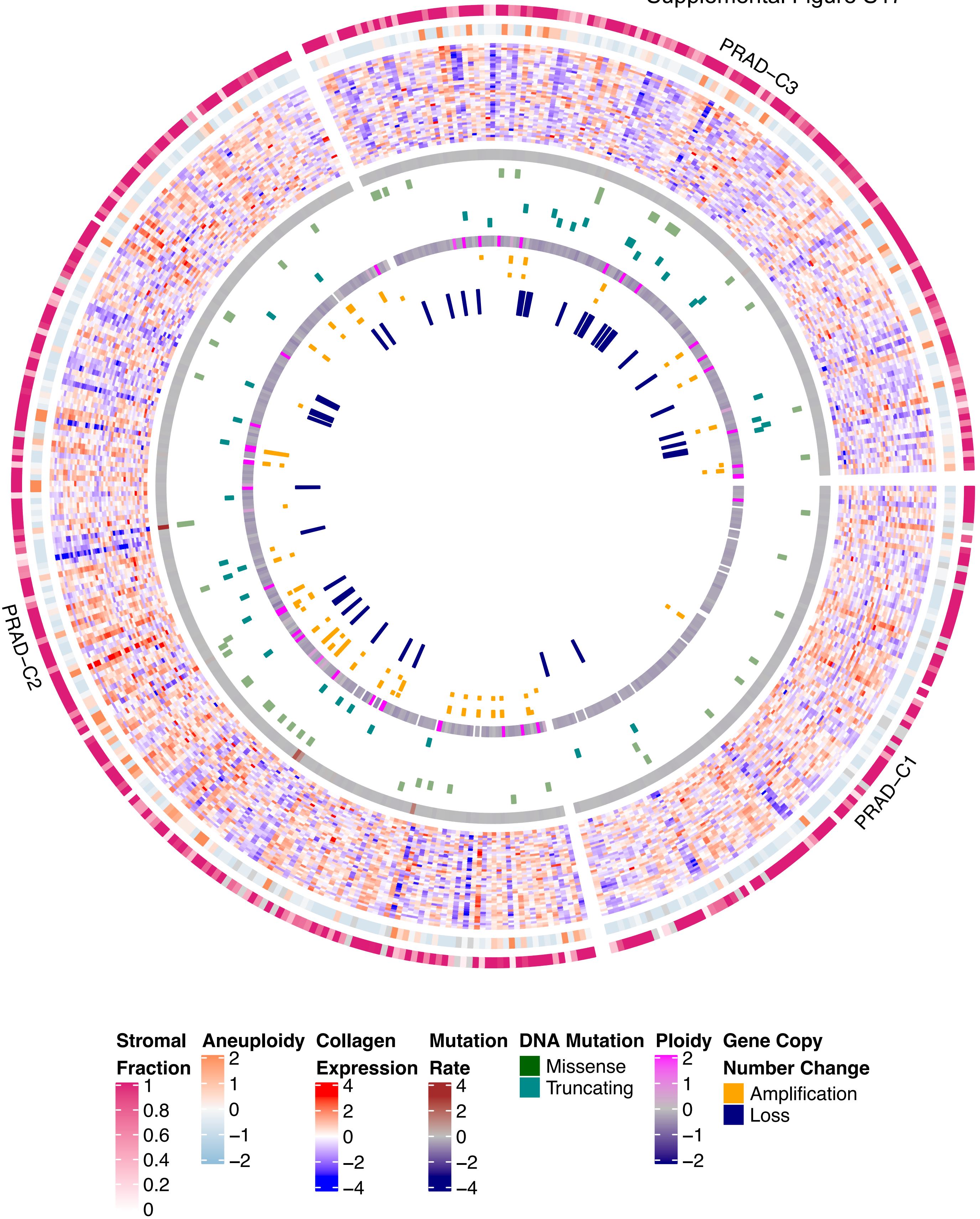

Supplemental Figure S17

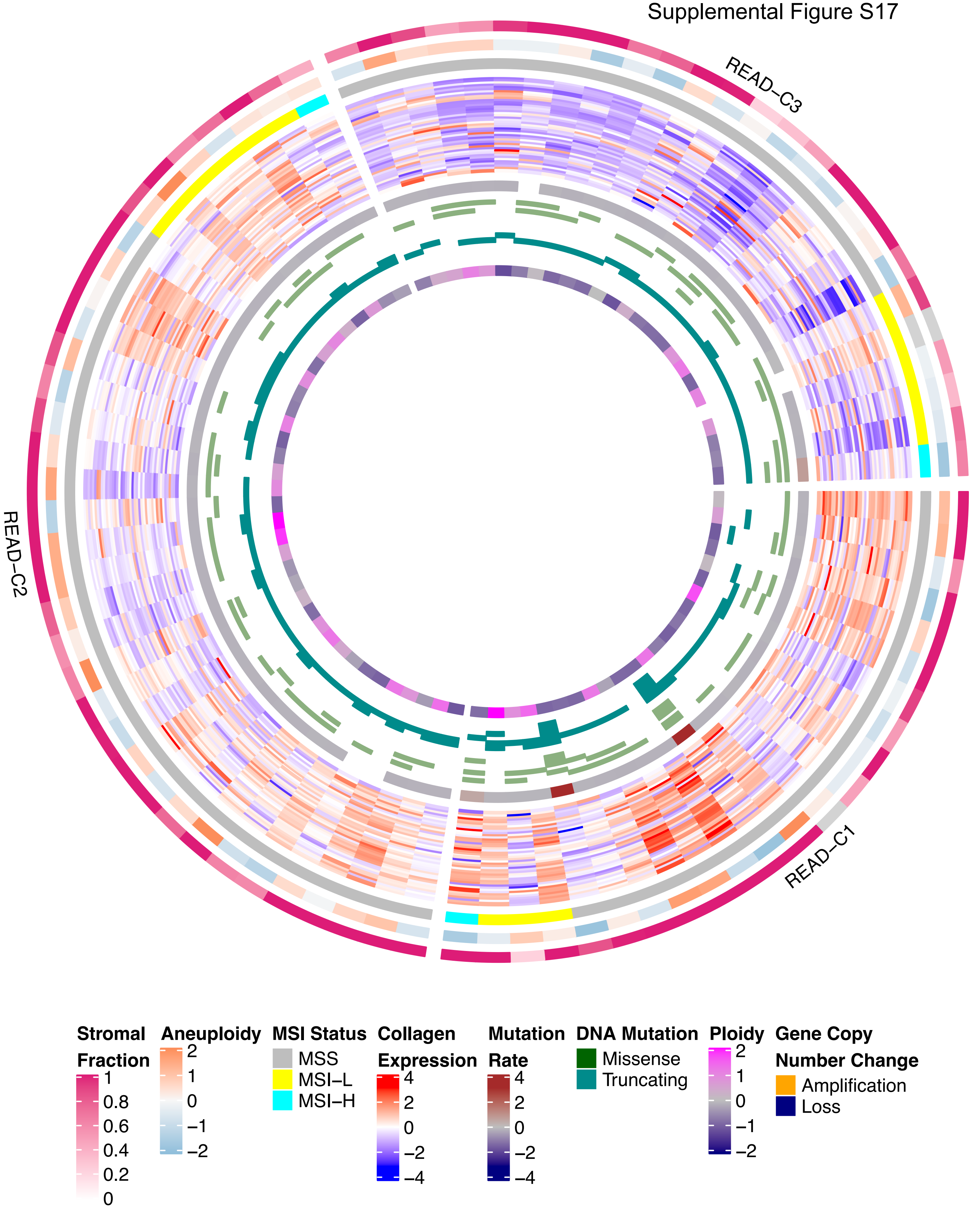

Supplemental Figure S17

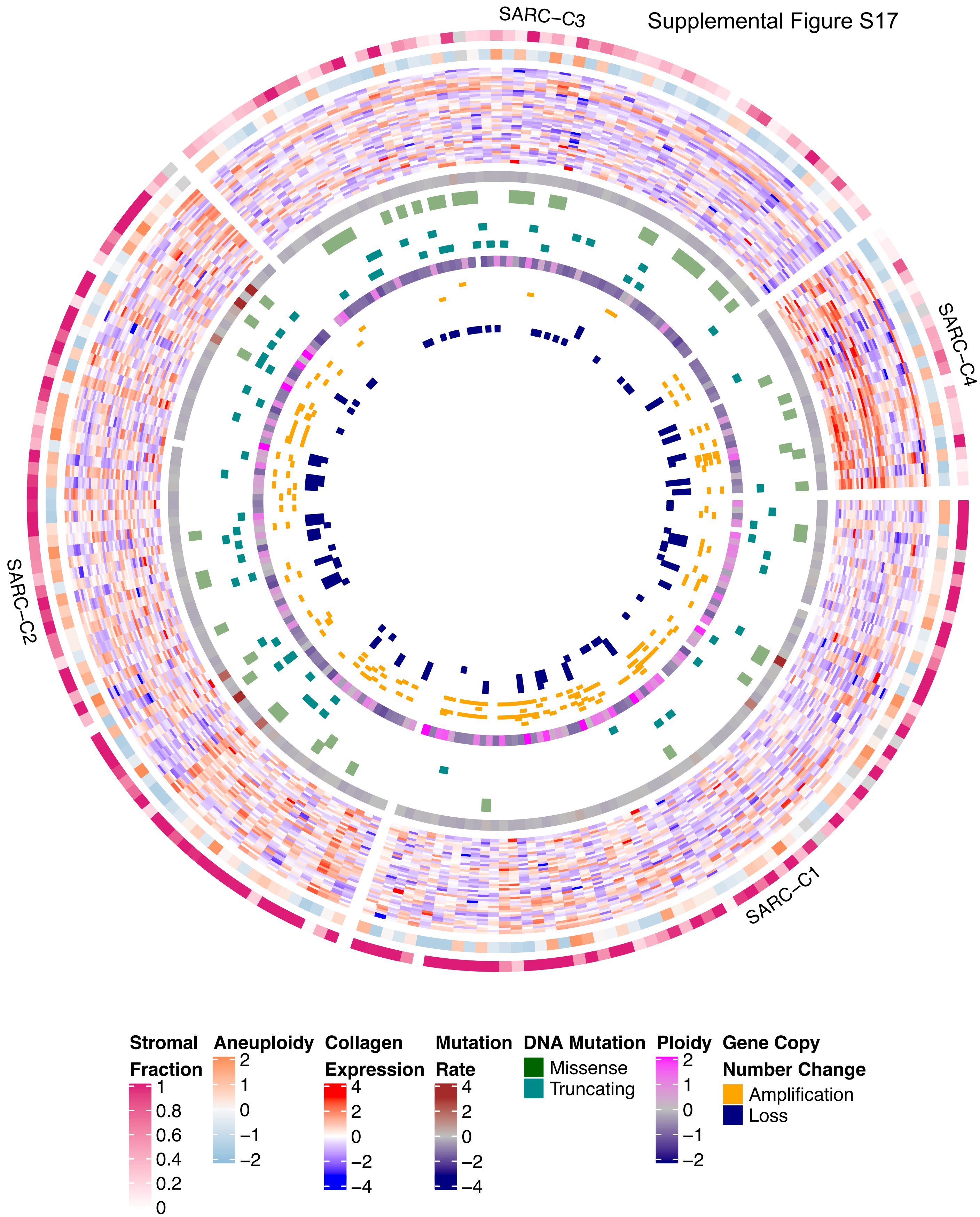

Supplemental Figure S17

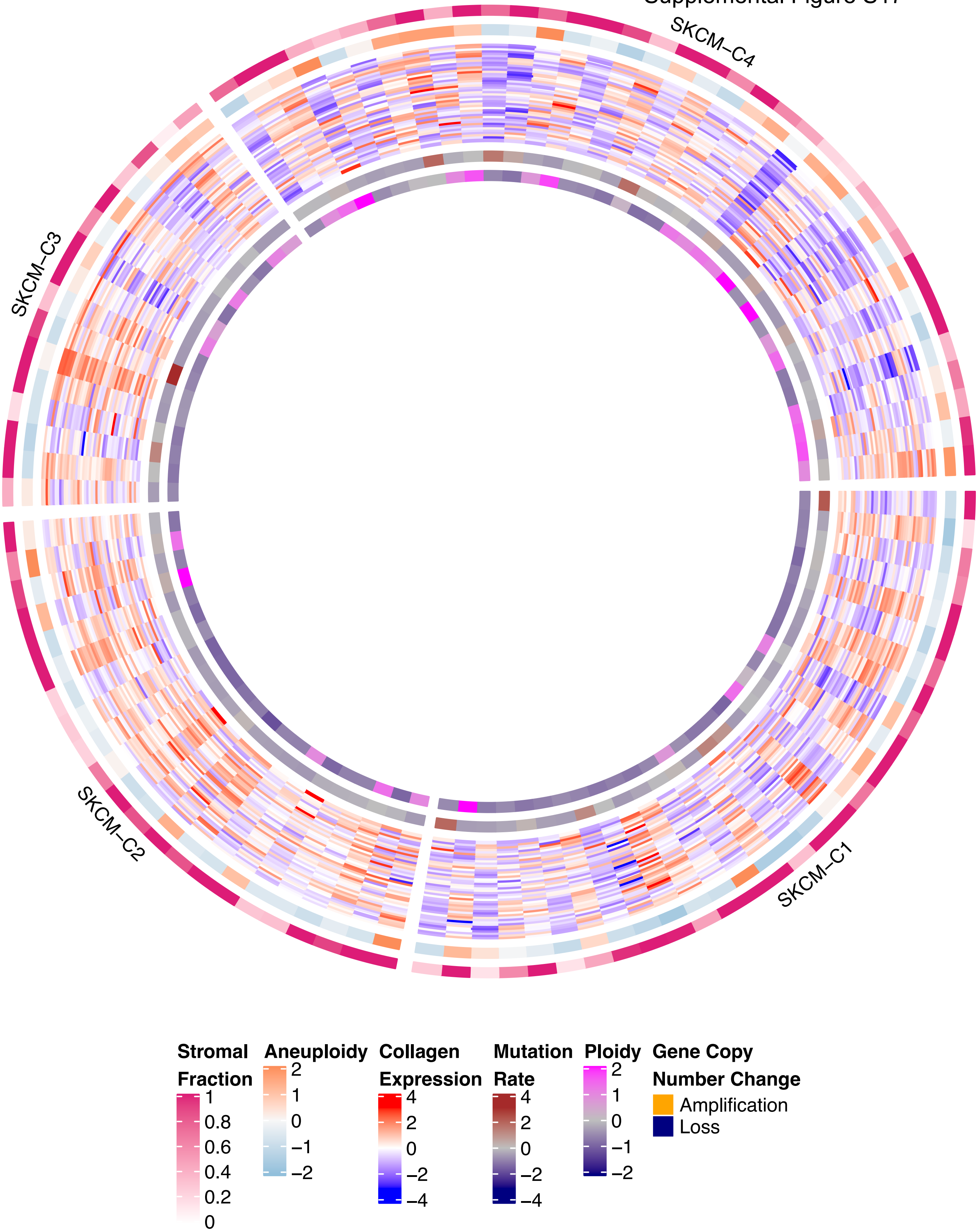

Supplemental Figure S17

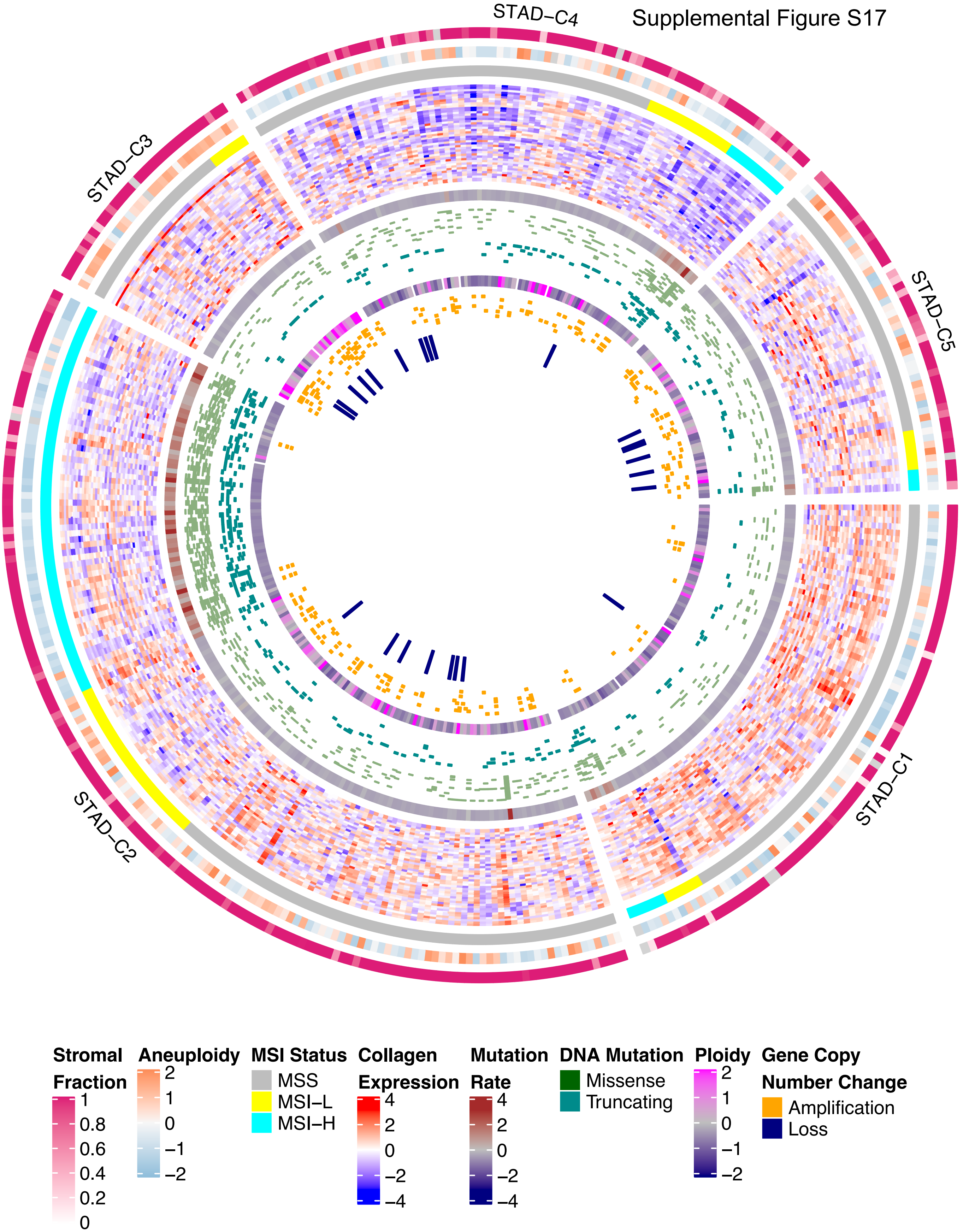

Supplemental Figure S17

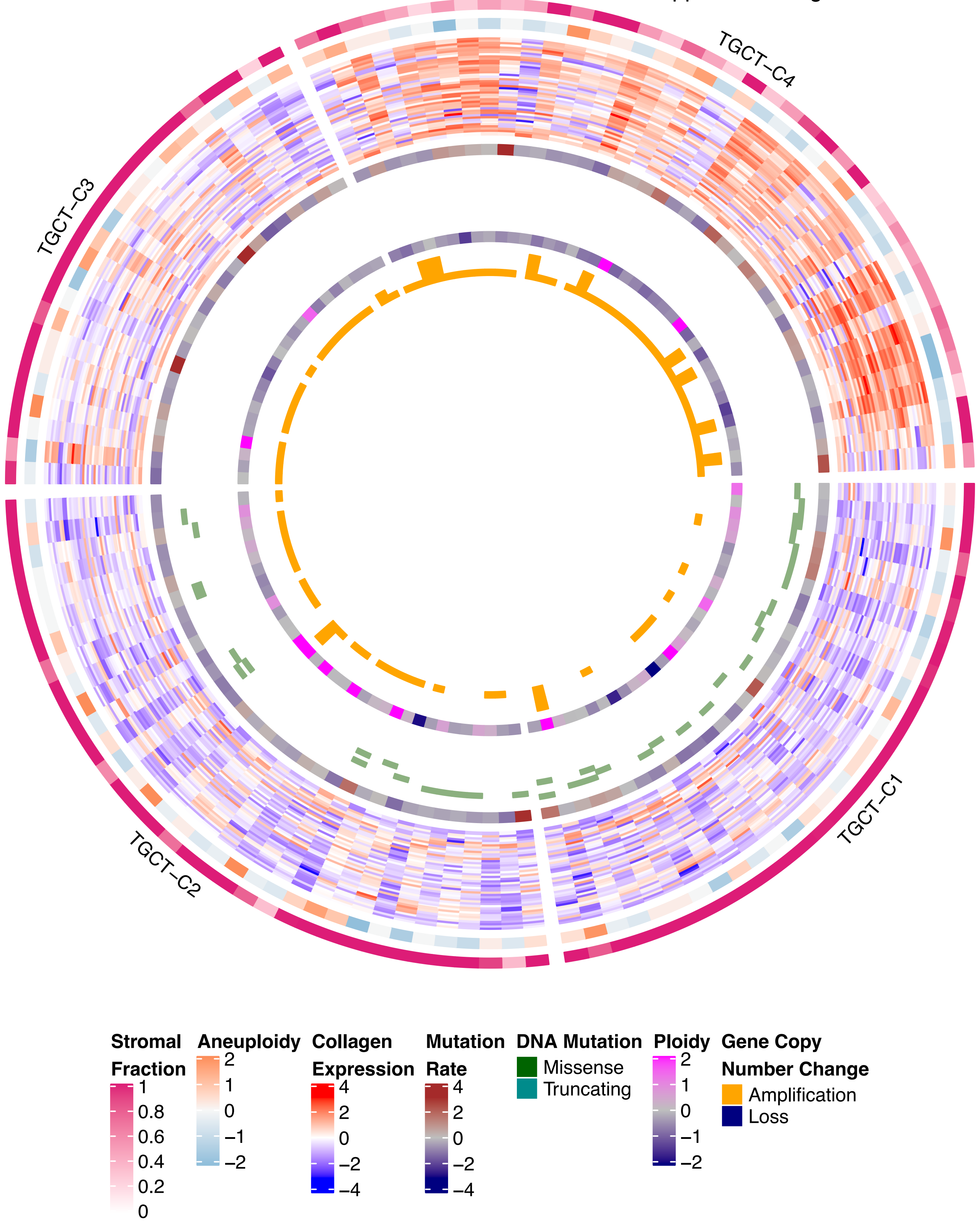

Supplemental Figure S17

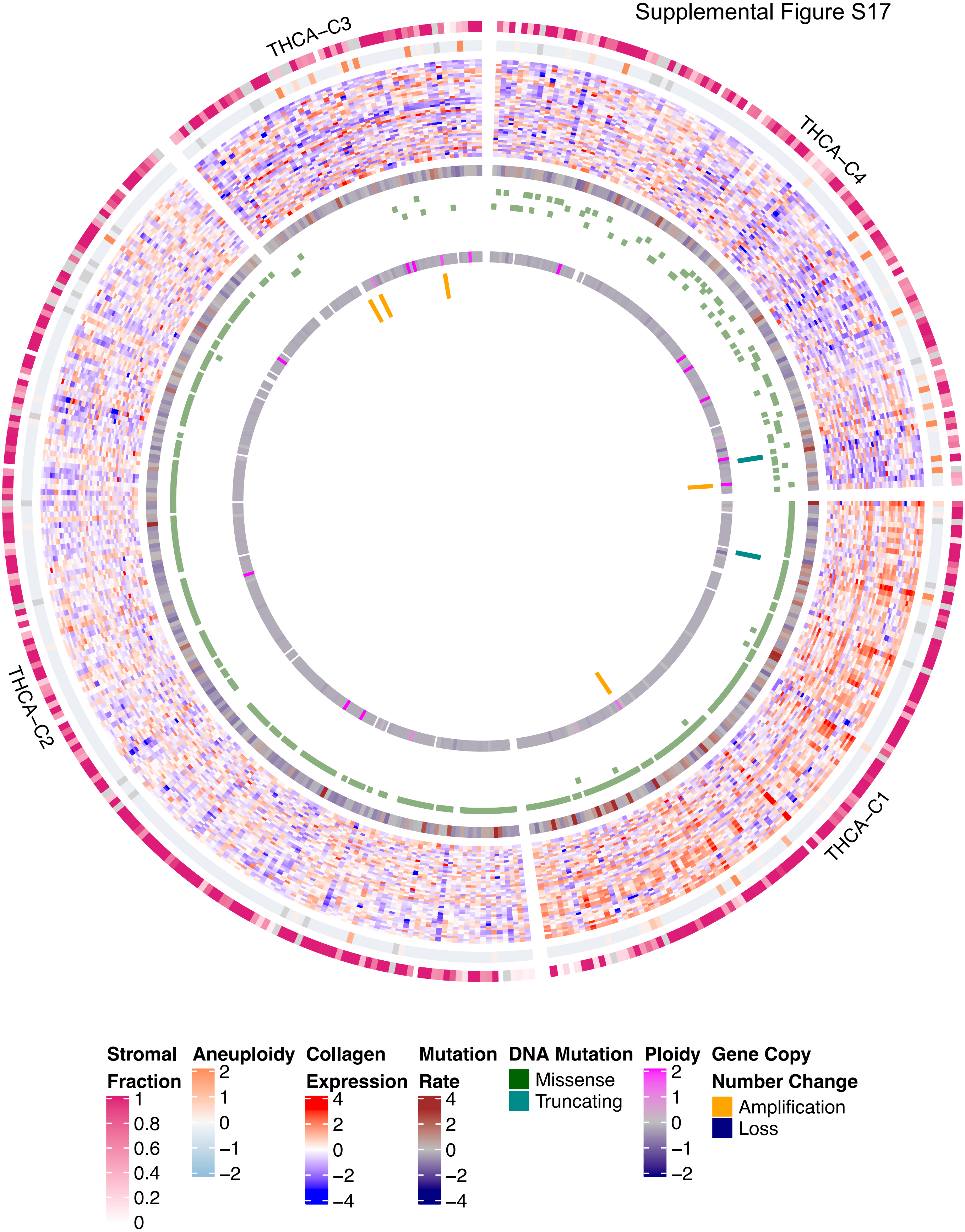

Supplemental Figure S17

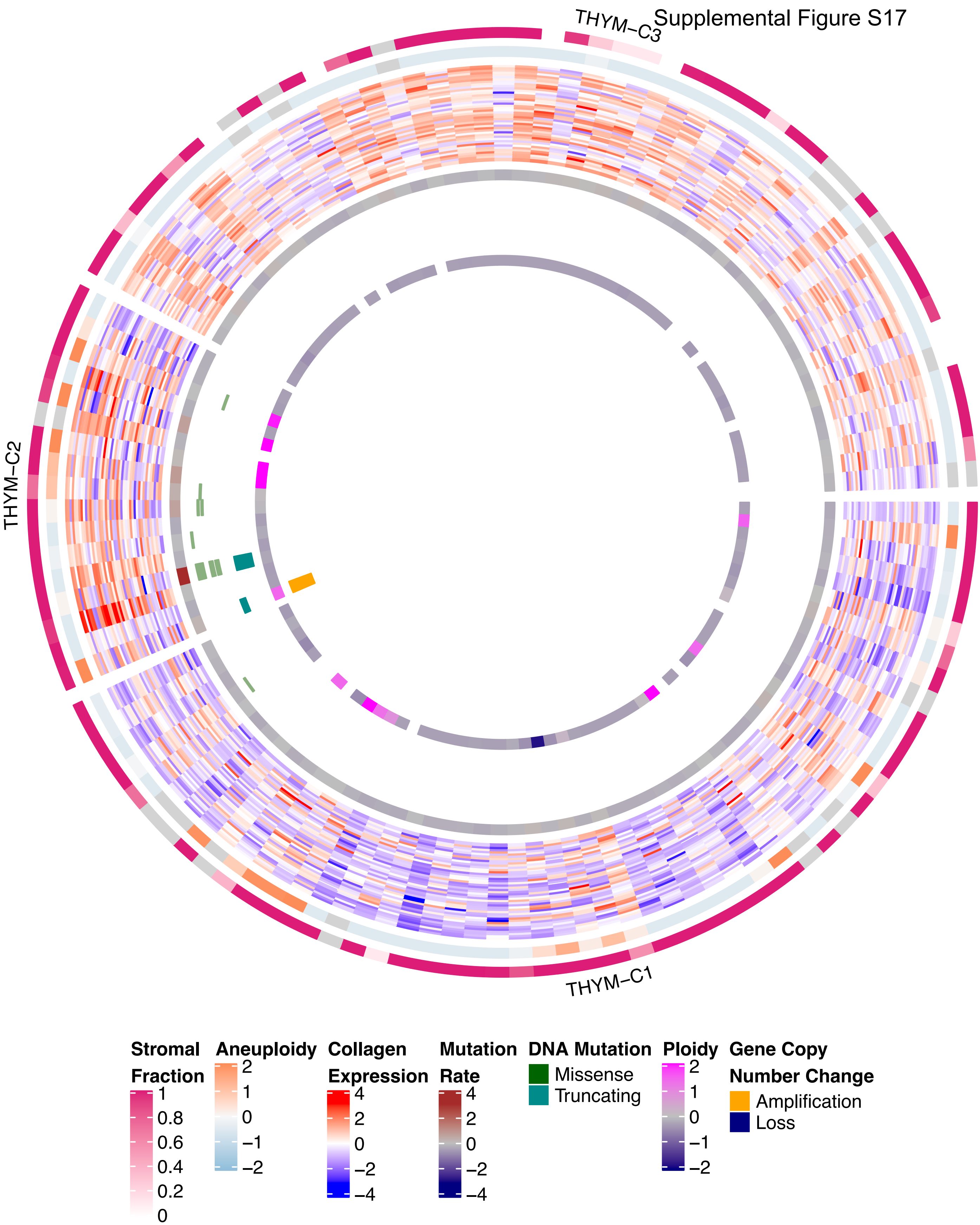

Supplemental Figure S17

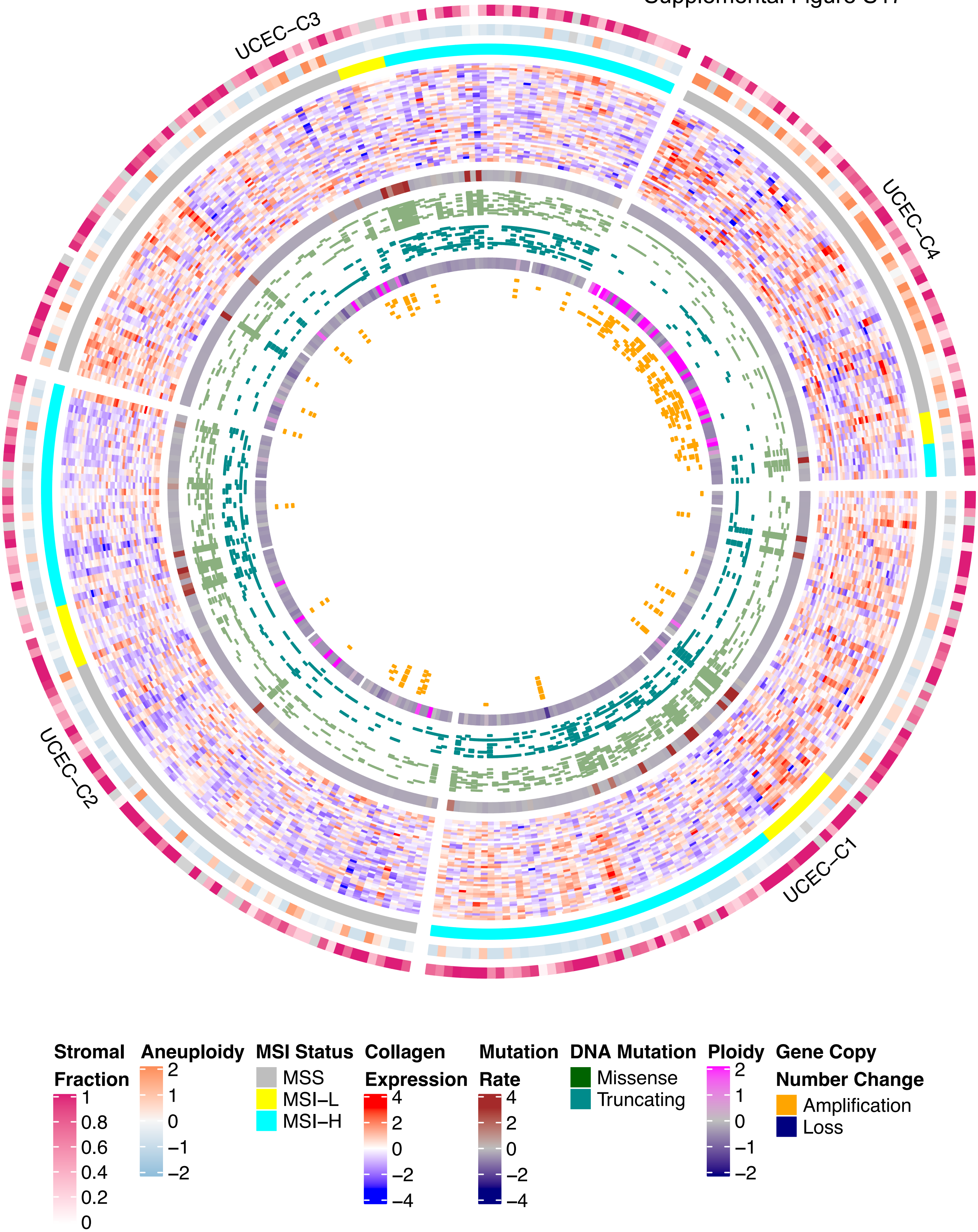

Supplement: Supplementary file 1 — Supplemental File [file 41525_2023_358_MOESM1_ESM.pdf]
